# TCA Cycle and Its Relationship with Clavulanic Acid Production: A Further Interpretation by Using a Reduced Genome-Scale Metabolic Model of *Streptomyces clavuligerus*

**DOI:** 10.3390/bioengineering8080103

**Published:** 2021-07-22

**Authors:** Howard Ramirez-Malule, Víctor A. López-Agudelo, David Gómez-Ríos, Silvia Ochoa, Rigoberto Ríos-Estepa, Stefan Junne, Peter Neubauer

**Affiliations:** 1 Escuela de Ingeniería Química, Universidad del Valle, Cali 25360, Colombia; valonso.lopez@udea.edu.co; 2 Grupo de Investigación en Simulación, Diseño, Control y Optimización de Procesos (SIDCOP), Departamento de Ingeniería Química, Universidad de Antioquia UdeA, Medellín 050010, Colombia; dandres.gomez@udea.edu.co (D.G.-R.); silvia.ochoa@udea.edu.co (S.O.); 3 Escuela de Biociencias, Universidad Nacional de Colombia sede Medellín, Medellín 050010, Colombia; rriose@unal.edu.co; 4 Chair of Bioprocess Engineering, Institute of Biotechnology, Technische Universität Berlin, D-13355 Berlin, Germany; stefan.junne@tu-berlin.de (S.J.); peter.neubauer@tu-berlin.de (P.N.)

**Keywords:** *Streptomyces clavuligerus*, clavulanic acid, fed-batch cultivation, TCA cycle intermediate, flux balance analysis, single-use bioreactor, shadow prices

## Abstract

*Streptomyces clavuligerus* (*S. clavuligerus*) has been widely studied for its ability to produce clavulanic acid (CA), a potent inhibitor of β-lactamase enzymes. In this study, *S. clavuligerus* cultivated in 2D rocking bioreactor in fed-batch operation produced CA at comparable rates to those observed in stirred tank bioreactors. A reduced model of *S. clavuligerus* metabolism was constructed by using a bottom-up approach and validated using experimental data. The reduced model was implemented for in silico studies of the metabolic scenarios arisen during the cultivations. Constraint-based analysis confirmed the interrelations between succinate, oxaloacetate, malate, pyruvate, and acetate accumulations at high CA synthesis rates in submerged cultures of *S. clavuligerus*. Further analysis using shadow prices provided a first view of the metabolites positive and negatively associated with the scenarios of low and high CA production.

## 1. Introduction

**Figure 1:**
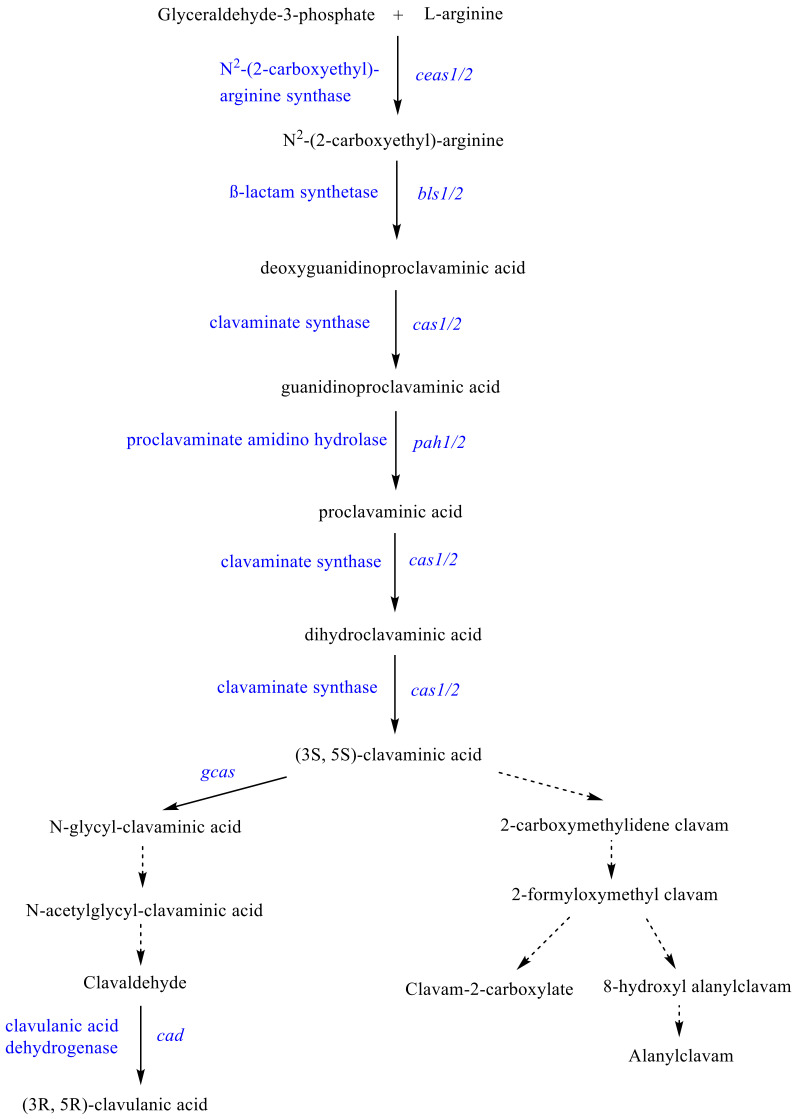
Schematic pathway of clavam biosynthetic pathway in *S. clavuligerus*.

*Streptomyces clavuligerus* has been widely studied for its ability to produce clavulanic acid (CA), a potent inhibitor of β-lactamase enzymes [1,2,3,4]. In this sense, many important achievements have been made regarding nutrients, environmental and operational conditions on CA production as previously reviewed by Ser et al. [5]. Although recent studies have contributed to a deeper understanding of *S. clavuligerus* metabolism [6,7,8] as a basis for strain engineering, system biology, and downstream processing (see recent review by López-Agudelo et al. [9]), the interaction of byproduct accumulation and product synthesis is not understood yet. Nonetheless, several *S. clavuligerus* strains have been engineered, yielding up to 6.7 g L^−1^ of CA in the supernatant [10,11]. Nevertheless, some steps in the clavam pathway (Figure 1), wherein CA is synthesized, remain unclear or even unknown, which hinders a detailed understanding of CA regulation and production [12,13,14,15,16,17,18].

In addition, a metabolic relationship between the tricarboxylic acid (TCA) cycle intermediates and antibiotic production has been observed in *Streptomycetes* sp. [19]. This relationship has been experimentally validated for the case of CA biosynthesis in *S. clavuligerus* cultivated in fed-batch and continuous modes [20,21]. Some experimental studies suggest that CA production is favored during fed-batch operation under phosphate limitation [22,23]. The differences in CA titers observed in fed-batch cultivations with defined media, along with the identification of the role of TCA cycle intermediates on the synthesis of CA in *S. clavuligerus* might help to identify metabolic targets susceptible to genetic modification for rational strain engineering.

Classical approaches for studying the nutritional, genetic, and environmental perturbation effects on metabolic rates include the use of constraint-based models (CBM) and genome-scale metabolic models (GEM) [24,25]. These models contain the stoichiometric information of the cell-specific metabolism, Boolean relationships between genes, proteins, and reactions, as well as nutritional constraints. GEMs work under the optimization of a cellular objective (usually the maximization of the biomass growth rate) with the aim of predicting carbon flux distributions [26]. Five GEMs have been published and used as tools for assessing the complexity of *S. clavuligerus* metabolism in silico, especially in connection with antibiotics production (CA and cephamycin C) [20,21,27,28,29]. The previous stoichiometric metabolic models for *S. clavuligerus* were not reconstructed de novo from the organism-specific reference sequences; instead, rather new biochemical information was added to the first GEM without changing its initial structure [9,21]. Only recently, an automated “bottom-up” reconstruction of the *S. clavuligerus* metabolism based on a reference genome sequence for the strain (refseq GCA_001693675.1) was used as a model draft. The initial draft was systematically curated, validated, and used for describing metabolic events during CA production in fed-batch cultures [21].

In summary, the “top-down” approach used in the first reconstructions of the *S. clavuligerus* metabolism used the genome annotation as a starting point for reconstructing the initial metabolic network [30]. Nevertheless, this strategy tends to generate large and complex networks with many gaps, blocked reactions, and non-connected metabolites in poorly characterized metabolic pathways [31]. On the contrary, “bottom-up” reconstruction uses the biochemical and organism-specific information as a starting point leading to higher quality manually-curated metabolic networks [31].

Application of GEMs in *S. clavuligerus* studies has focused on the interplay between the antibiotics’ biosynthesis in the cephalosporin and clavam pathway and the Central Carbon Metabolism (CCM). The complexity and quality of GEMs are increasing with the availability of improved high-quality sequences, and hence, better gene annotations. Nevertheless, the mathematical solution of the optimization problem under pseudo-steady state assumption frequently shows a large number of reactions with a zero flux. The majority of analyses are focused on the central metabolism and specific pathways activated under certain conditions. Reduced models have emerged recently as alternatives to GEMs which describe the metabolism with a lower number of reactions while preserving the pathways of interest. Reduced networks also facilitate the inclusion of kinetic equations, which was infeasible for a whole GEM. Some systematic and algorithmic approaches have been proposed to reduce a GEM into a small model while maintaining the quality of the original model. Reduced GEMs for the organisms *Pseudomonas putida*, *Escherichia coli,* and for human metabolism (Recon 2 and Recon 3D) have been recently constructed using a “bottom-up” strategy. They have been used for integrating high throughput data and exploiting demanding computational algorithms for parameter optimization in large-scale kinetic models [32,33,34,35]. Therefore, a consistent model representation of the physiology of *S. clavuligerus* might also be reached by a reduced model retaining the predictive capabilities of the GEM [34].

Some previous reports have been connected to the CA biosynthesis with the availability of precursors and metabolic flux distribution [20,28,36,37]. Additionally, kinetic studies have proven that CA is susceptible to hydrolysis in aqueous solutions [38,39,40]. Recently, a robustified experimental design for ^13^C-Metabolic Flux Analysis was conducted in *S. clavuligerus* [41]. However, metabolic bottlenecks for increasing the CA biosynthesis rate remain to be identified. In this respect, the high flexibility, lower complexity, and small size of reduced GEMs are helpful in identifying bottlenecks between the CCM and secondary metabolite production that might be masked in the large-size models. Additionally, critical parameters that could improve CA production can be investigated by the analysis of metabolic fluxes, constraint-based modeling approaches, and the interpretation of shadow prices, resulting from the solution of the Flux Balance Analysis (FBA) problem [25,42].

In this study, experimental data of fed-batch bioreactor cultivations of *S. clavuligerus* cultivated under low shear stress conditions (non-stirred) are integrated with constraint-based modeling using a reduced, curated, and validated model of the microorganism to understand further the role of central carbon precursors in the synthesis of CA.

## 2. Materials and Methods

### 2.1. Strain and Cultivations Conditions

*S. clavuligerus* DSM 41,826 was used for all cultivations. Periodically, mycelium stock cultures were reactivated and stored at −80 °C in cryotubes with glycerol solution (16.7% *v*/*v*). The media for inoculum, production, and the feed have been published previously [12,20,43]. One point two microliters of glycerol stock were transferred to 50 mL of seed medium in 250 mL UltraYield^©^ shake flasks which were sealed with air-permeable AirOtop membranes (both from Thomson Instrument Company, Oceanside, CA, USA). The seed cultures were grown in a rotary shaker incubator for 26 h at 200 rpm and 28 °C. For the second preculture, 10 mL of cultivated seed broth was transferred to 90 mL of bioreactor batch medium in a 500 mL shake flask. The flasks were covered with AirOtop seals, and cells were grown for 20 h at the same condition as the seed medium. The preculture was inoculated at 10% *v*/*v* into the bioreactor batch medium. The feed composition was as follows: glycerol 120 g L^−1^, (NH_4_)_2_SO_4_ 8 g L^−1^, and K_2_HPO_4_ 2 g L^−1^.

Fed-batch cultivations were conducted by duplicate in a 20 L single-use 2D rocking-motion bioreactor CELL-tainer^®^ CT 20 (Cell-tainer Biotech BV, Winterswijk, The Netherlands) with a working volume of 1 L during the batch phase as described in [21,43]. After 36 h, feeding was started at a constant rate of 0.01 L h^−1^ for 72 h up to 1.8 L as the final volume. Cultivations were controlled at 28 °C, 0.6 vvm, and a pH-value of 6.8. Samples of 2 mL were taken at 12 h intervals for dry cell weight (DCW) determination. Samples were centrifuged at 15,000 rpm and 4 °C for 10 min and dried overnight at 75 °C up to constant weight. The supernatants samples were analyzed via HPLC-DAD and HPLC-RID for CA and intermediates quantifications, respectively, as described previously [44,45].

### 2.2. Bottom-Up Reconstruction of a Reduced Genome-Scale Metabolic Model of S. clavuligerus

The GEM of *S. clavuligerus* (iDG1237), recently reported by Gómez-Ríos et al. [21], was used as a bottom-up draft reconstruction. The high-quality GEM model iDG1237 is the most recent reconstruction of *S. clavuligerus* metabolism, which includes 1518 metabolites, 2177 reactions, and 1237 genes and represented accurately experimentally observed phenotypes during CA secretion. The bottom-up strategy consisted of a systematic process of manual and semi-automated curation [31]. Initially, the main metabolic pathways of the iDG1237 GEM were identified and used for the generation of a first draft of the reduced stoichiometric matrix. This draft contained the reactions associated with CCM, such as glycolysis/gluconeogenesis, pentoses phosphate pathway, TCA cycle, amino acid metabolism, urea cycle, and clavam pathway. The reactions not associated with those subsystems were not considered in the reduced draft model. Subsequently, the zero-flux reactions and dead-end metabolites in the network were identified and eliminated or curated by filling the gaps with reactions taken from the BiGG Models database [46,47] and the reduced model of *E. coli* [33]. Likewise, lumped reactions of biomass precursors of *E. coli* that also exist in *S. clavuligerus* were included [48]. The production of biomass precursors was checked after reduction. The biomass precursors that could not be produced in the reduced model (named in this study as sclav_red) were identified, and the reactions required for their production to maintain the consistency of the model were included. In the case of those reactions not reported in the *S. clavuligerus* genome, the corresponding precursors were eliminated from the reduced biomass reaction. The carbon contribution of those reactions to biomass formation was not statistically significant. The stated steps were applied iteratively until a reduced metabolic network of *S. clavuligerus* with high topological consistency was obtained.

Additionally, thermodynamically infeasible cycles (TICs) were checked and corrected [49,50,51]. TICs are formed by cyclic reactions carrying fluxes without exchanging energetic metabolites, such as ATP, and therefore violate the second law of thermodynamics. The TICs identification and correction was carried out by adapting the methodology described by López-Agudelo et al. [52] and Gómez-Ríos et al. [21] (https://github.com/viclopezag/UR-TICS, last accessed 4 July 2021). Once identified, the TICs formed by erroneous directionality assignments were corrected by directionality restriction based on a change in the Gibbs free energy obtained from the eQuilibrator database [53]. Similarly, transport reactions were revised to change the directionality of those only associated with the import and export of metabolites. All the modifications applied to the model draft are listed in the Supplementary Table S1.

### 2.3. Validation of the Sclav_Red Model Using Experimental Data

The sclav_red model validation was performed by comparing the predicted fluxes at a pseudo-steady state against experimental data of *S. clavuligerus* in continuous cultures [20]. Here, FBA simulations were performed by maximizing the specific growth rate under the minimization of the Manhattan norm of fluxes by using the “optimizeCbModel” function along with the “minNorm” option, both included in the COBRA toolbox [54]. The experimental specific growth rate in continuous cultures was compared with the model’s predictions. No hard constraints were imposed on the optimization. Only soft constraints were used so that the experimental exchange fluxes were in the range defined by the upper and lower bounds (File S2). Exchange fluxes of glycerol, O_2_, CO_2_, and CA were constrained according to the experimental data and were used as lower (Glycerol and O_2_) or upper (CA and CO_2_) bounds constraints. The bounds were systematically explored by Flux Variability Analysis (FVA) to avoid unbounded fluxes for the nutrients. Mean square error (*MSE*) between the model outputs from FBA and experimental fluxes in continuous cultures at different dilution rates [20] was computed to evaluate the quality of predictions.

### 2.4. FBA and Shadow Prices as Tools for Sensitivity Analysis of Metabolic Networks

FBA has been used very often to study the metabolic flux distribution of an entire metabolic network [55,56,57,58]. Under the assumption of a pseudo-steady state, the net flux of production and consumption of a metabolite is assumed to be zero. In order to simulate the present experimental data of this paper, a two-step optimization approach was used. First, the maximization of CA production was defined as an objective function under the assumption that during phosphate limitation, CA production is achieved; thus, configuring the FBA primal problem. Second, the minimization of the Manhattan norm of the absolute value of all the intracellular fluxes was done by using as a constraint the maximum CA value obtained in the first step optimization. This FBA optimization was implemented in the cobra toolbox function “optimizeCbModel” [54]. Additional to the vector of fluxes, the vector of shadow prices per metabolite was calculated with the solution of the optimization problem. Shadow prices were interpreted as the sensitivity of FBA to flux imbalances obtained during the solution of the dual problem of linear optimization. The shadow prices vector relates the change in the objective function of the primal problem (i.e., CA maximization) when the flux of one of the intracellular metabolites increase or decrease, resulting in a deviation from the steady state; that is the sensitivity of FBA to flux imbalances obtained during the solution of the dual problem of the linear optimization. Reznik et al., 2013 showed the importance of shadow prices in the analysis of metabolic networks. For instance, a negative value of the shadow price for a metabolite implies that additional outflow of this metabolite would increase the value of the objective function showing that the metabolite is actually a limiting compound for the objective [42]. The biological interpretation of shadow prices in metabolic modeling has been summarized as follows: (i) negative values of shadow prices are obtained for those metabolites whose flux is limiting the objective; (ii) zero value implies that the objective function is not sensitive to that metabolite; and (iii) positive values of shadow prices are obtained for those metabolites with sufficient intracellular flux for reaching the objective.

Further details on shadow prices utilization in metabolic modeling are available (see references [25,54]). The experimental growth rate was set as a hard constraint in the optimization problem with the aim to get a better representation of the metabolic scenarios, while the measured uptake and secretion rates (when available) were set as lower (O_2_, glycerol, succinate, oxaloacetate, and malate) and upper (CO_2_, pyruvate and acetate) bounds, respectively. The IBM CPLEX Studio Optimizer v12.10.0 was used for the solution of the optimization problems. It is worth mentioning that the sign of shadow prices calculated by CPLEX solver was negative (−λ), and it was considered for the further biological interpretation of shadow prices results.

Furthermore, the solution space for the fluxes’ distributions of two different metabolic states of *S. clavuligerus* was explored by using FVA and flux sampling using the sclav_red metabolic model. The model was constrained with uptake and secretion rates corresponding to 36 h (batch stage) and 48 h (12 h after starting the fed-batch stage) of cultivation. The model predictions were contrasted with experimental exchange fluxes for the assayed metabolites and nutrients using the squared error (*SE*) as indicated in Equation (1) for a given metabolite *i*. *MSE* was calculated as indicated in Equation (2) to assess the deviation of model predictions and experimental fluxes for a given metabolic scenario.

(1) $$SE_{i}={\left(X_{i}-{\hat{X}}_{i}\right)}^{2}$$

(2) $$MSE=\frac{1}{p}\overset{p}{\underset{i=1}{\sum }}{SE}_{i}$$

where p is the number of experimental fluxes considered in the scenario, X is the experimental flux value, and $\hat{X}$ is the model-predicted flux value.

In addition, the feasible flux range was determined for each reaction via FVA. Alternatively to the unique solution provided by the FBA problem, the coordinate hit-and-run with rounding algorithm (CHRR) [59] was used for sampling the solution space for the explored cultivation conditions (batch and fed-batch stages). The following CHRR parameters were set: the sampling density, nStepsPerPoint = 1848, and the number of samples, nPointsReturned = 5000. A Kruskal—Wallis test was used to assess whether flux samples generated using either the batch (36 h) or fed-batch (48 h) constraints stemmed from the same statistical distribution [60]. A principal component analysis (PCA) was applied to metabolite shadow prices for the identification of those metabolites that contributes to the changes in flux distributions or phenotypes between the culture conditions under study.

All simulations were carried out in RAVEN 2.0 [61] and COBRA Toolbox v3.0 [54] under MATLAB 2020b (see Supplementary File S3).

## 3. Results and Discussion

### 3.1. Fed-Batch Cultivation of S. clavuligerus

**Figure 2:**
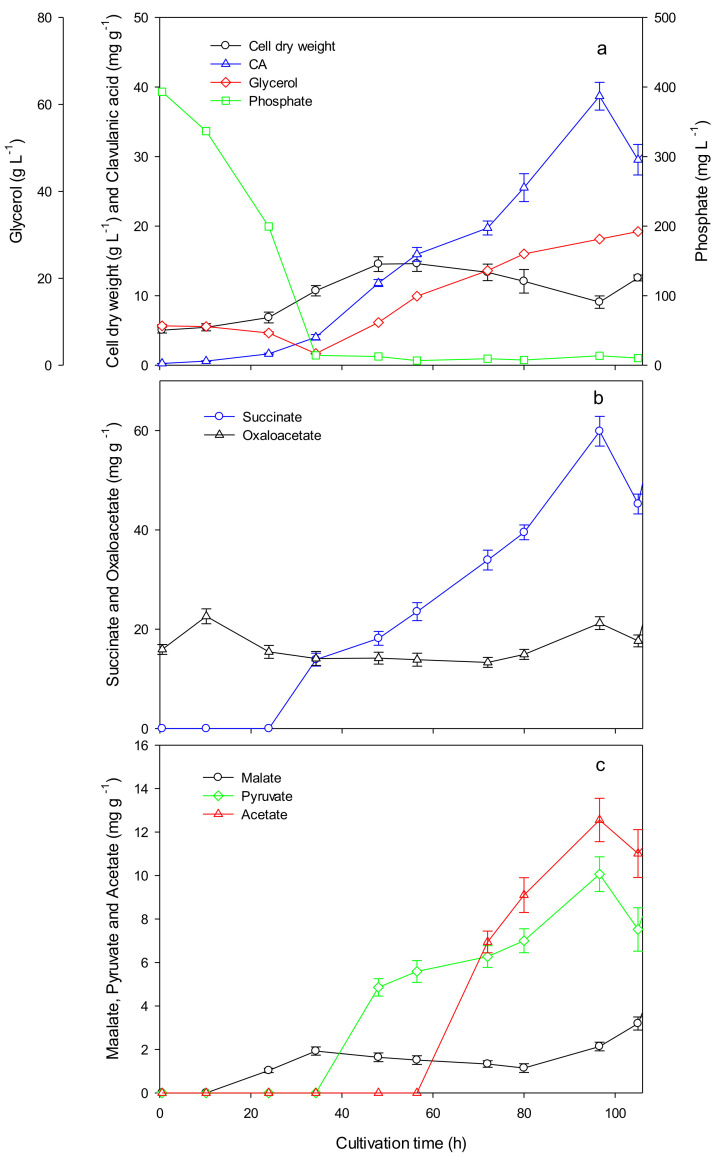
Fed-batch cultivation of *S. clavuligerus*. (**a**) Cell dry weight (circle), phosphate (square), glycerol (diamond), and clavulanic acid (triangle) as a function of cultivation time. (**b**) Succinate (circle) and oxaloacetate (triangle) as a function of cultivation time. (**c**) Malate (circle), pyruvate (diamond), and acetate (triangle) as a function of cultivation time.

Figure 2a displays the profiles of biomass as DCW, glycerol, phosphate, and CA concentrations during fed-batch cultivations of *S. clavuligerus* in a 2D rocking bioreactor starting at 5% and finalizing at 9% filling volume. The batch phase lasted for the first 36 h of cultivation; then, the feeding started at constant feed rate until the 108 h. The maximum biomass concentration was attained at 52 h with 14.6 g DCW L^−1^. Afterward, a clear decrease in biomass concentration was observed due to the phosphate limitation and dilution due to the feed rate. CA was detectable during the complete phosphate-limited stage up to a maximum concentration of 370.9 ± 14.1 mg L^−1^ (Figure 2a). TCA intermediates succinate, pyruvate, and acetate were accumulated during CA synthesis, while oxaloacetate and malate were rather constant during the fed-batch cultivation (Figure 2b,c). A comparable metabolic scenario (acetate, succinate, oxaloacetate, and malate accumulation during CA synthesis) has already been reported by Ramirez-Malule and co-workers in continuous cultures with low CA titers [20]; however, the accumulation levels of CA and the TCA cycle intermediates was almost 10-fold higher during fed-batch operation in comparison with the reported results for continuous mode. The higher extracellular concentration of those metabolites is linked to the higher biomass, more active energetic metabolism, and specialized metabolites production [21].

Previously, a difference in the metabolic performance of *S. clavuligerus* cells has been observed when cultivating at different shear conditions and hence, intracellular oxygen uptake flux. The 2D rocking bioreactor is characterized by enhanced gas mass transfer due to large film surface and combination of vertical and horizontal motion leading to wave formation and gas suction into the liquid phase with lower shear stress than stirred tank bioreactors [62]. CA productivity in *S. clavuligerus* is intimately related to intracellular oxygen availability since the biosynthetic pathway requires molecular oxygen in those reactions catalyzed by the clavaminate synthase. The metabolic scenario observed in this work resembles that observed in a stirred tank bioreactor with comparable CA productivity, suggesting that intracellular oxygen availability was likely the same [21,43]. In this case, the large headspace and low volume favored the oxygen uptake and hence, CA secretion, compared with cultivations at similar conditions in 2D rocking bioreactor but higher (5-fold) operating volume.

The observed succinate accumulation was reported as a result of increased activity of nitrogen metabolism and hence, with an existing phosphate limitation, due to CA production. Succinate is released as a byproduct in the reaction steps of CA biosynthesis catalyzed by the clavaminate synthase, an α-ketoglutarate iron-dependent oxygenase (see Figure 1) [63,64,65]. Such activation of specialized metabolism in *S. clavuligerus* occurs under phosphate limitation probably as part of an energy-regulating mechanism that controls ATP generation and ATP saving according to the anabolism requirements [21].

Pyruvate and acetate accumulations during CA production (Figure 2c) were associated with the requirements of the CCM and CA biosynthesis, respectively [20,21]. Oxaloacetate and malate were rather constant during fed-batch operation with smooth variations linked to phosphate and limited availability of amino acids, which affect growth and energetic metabolism [21]. All those accumulations supported the higher activity of the TCA cycle and energetic metabolism expected during the CA biosynthesis. Bushell and co-workers [37] reported that feeding amino acids from the oxaloacetate family improved the CA yield more than 10-fold compared with non-supplemented chemostat cultivations, thus resuming a bottleneck that otherwise occurs. Previously, a reaction mechanism for the N-acetyl-glycyl-clavaminic acid formation involving acetate by an ATP-grasp enzyme was proposed [12]. This hypothesis is supported by the consistent acetate accumulations in chemostat and fed-batch cultures (this work). Furthermore, acetylated compounds, such as N-acetyl-glycyl-clavaminic acid and N-acetyl-clavaminic acid, have been suggested as intermediates in the clavam pathway [66,67]. The experimental results of this work and those reported by us previously [20,21,43] suggest a clear interrelation between the TCA cycle fluxes, the availability of intermediates along with pyruvate and acetate accumulation, and CA biosynthesis.

### 3.2. Construction of a Reduced Model of S. clavuligerus Metabolism

Reduced metabolic models are condensed representations of an organism’s biochemical network. The mathematical analysis of the biochemical network allows studying in silico the cellular phenotypes observed in vivo. This reduced representation leads to a reduction in computational time of complex simulations without loss of fidelity. Additionally, it is expected that the reduction of a high-quality GEM may generate a reduced model with equivalent accuracy. The first draft of the reduced model included the reactions of CCM and numerous gaps, single connected and dead-end metabolites as a consequence of insufficient connection between reactions in the network. Thus, 317 reactions were added, including the lumped reactions summarizing some minor metabolic pathways. The added reactions and lumped reactions (identified as LMPD) are detailed in Supplementary Table S1. Forty-two reactions were modified to fix the single-connected or dead-end condition of some metabolites. Finally, the directionality of 20 reactions was modified during the thermodynamic curation. The reduced metabolic network encompassed 652 reactions, 1552 metabolites, and 1284 genes. The validation results for the sclav_red model against pseudo-steady state experimental data [20] are presented in Table 1. The sclav_red model (-mat format) is available in the Supplementary Material (File S1).

The *MSE* for the reduced model of *S. clavuligerus* metabolism was lower than for previously reported GEMs. A complete performance evaluation of *S. clavuligerus* GEMs was made by Gómez-Ríos et al. [21]. Although the iDG1237 model has the lowest *MSE* reported for a *S. clavuligerus* metabolic model [21], the sclav_red model exhibited a *MSE* in the same order of magnitude, which means that both models have a similar performance in the prediction of this specific experimental scenario. Indeed, it was expected that the parent GEM would have a lower error since it is the most updated GEM for the organism. The sclav_red constituted a condensed representation of the metabolic capabilities of *S. clavuligerus*, with a special focus on the CCM and CA biosynthesis. This model constitutes a more ‘computationally’ efficient metabolic network compared to the large-scale iDG1237. This network could be applied for exploring metabolic phenotypes using constraint-based methods that require high computational power, such as flux sampling [68], thermodynamically constrained stoichiometric models that use energy balance as a constraint [69], or large-scale kinetic modeling [32,70].

### 3.3. Flux Distributions in S. clavuligerus at Two Different Metabolic States during Fed-Batch Cultivations

A constraint-based analysis was conducted at the two different metabolic states expected during the batch and fed-batch stages of the cultivations, under the assumption of quasi-steady-state conditions between two consecutive data points. The curated and reduced reconstruction of *S. clavuligerus* metabolism sclav_red was used in all the simulations.

The analysis of the two stages of cultivation aimed to explore the metabolic phenotypes that occurred during the two different operation modes by means of FVA, FBA (Table S2), and flux sampling. The pseudo-state condition was assumed since the measured growth rate was rather constant (an elongation of exponential growth phase) in both time steps. Table 2 displays the metabolic constraints used for the in silico studies and the comparison between the experimental and the simulated flux rates. Notice that in those metabolites with *SE* = 0, the exchange flux assumed the lower bound value constraining the search space, while in some cases, the metabolites assumed values in the range defined by the upper and lower bounds. According to the results of *MSE* calculations, the reduced metabolic model was suitable for representing the glycerol assimilation, CA production, succinate, oxaloacetate, malate, and acetate secretion fluxes during both batch and fed-batch stages. However, the specific O_2_ consumption and specific CO_2_ production rates showed the highest discrepancies in both operation modes. Likewise, the sclav_red model provided a better representation of the metabolic scenario of the fed-batch (*MSE* = 0.86) stage than the batch stage (*MSE* = 1.7).

**Figure 3:**
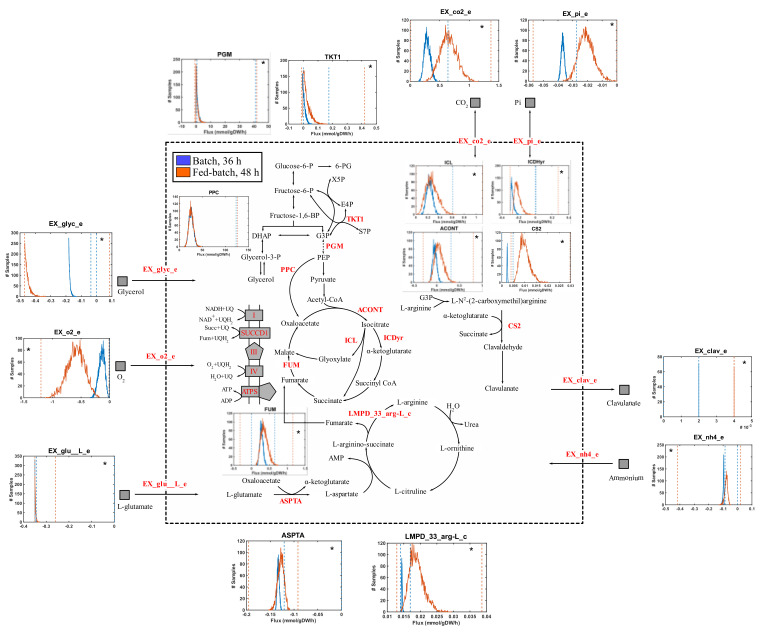
Summary of intracellular and exchange flux distributions in the *S. clavuligerus* reduced metabolic network for Table 2. Distributions indicated with blue, and orange represent the metabolic scenarios in batch (36 h) and fed-batch mode (48 h), respectively. Dotted lines represent flux ranges of FVA. The significantly different distributions (*p* < 0.001, Kruskal—Wallis test) have been marked with an asterisk (*). The reaction names with the prefix EX_ corresponds to the exchange reactions of consumed or produced metabolites by *S. clavuligerus*. glu__L: L-glutamate, o2: Oxygen, glyc: glycerol, co2: CO_2_, pi: phosphate, clav: clavulanic acid, nh4: NH_4+_. For a better discrimination of flux sampling distributions, please refer to Figure S1.

Figure 3 shows the predicted intracellular flux distributions of *S. clavuligerus* during cultivation in the 2D rocking single-use bioreactor at 36 and 48 h. The results showed that the high CA production during the fed-batch stage could be associated with the probability of increased flux through the transketolase (TKT1) enzyme, which favors the production of glyceraldehyde−3 phosphate (G3P) from the pentose phosphate pathway and glycolysis. Availability of G3P favors the CA biosynthesis as this metabolite is included in the synthesis of *N*^2^-(2-carboxyethyl)-arginine, the first intermediate of the clavam pathway produced via *N*^2^-(2-carboxyethyl)-arginine synthase (CEAS) (Supplementary File S2). Similarly, the clavaminate synthase (CS2) was also activated by this increased flux in the clavam pathway towards clavaminic acid, a point of bifurcation for the biosynthesis of CA and clavam 5S compounds. One of the main observed phenotypes in our experimental data evidenced the link between TCA intermediates, such as succinate, oxaloacetate, and malate, and CA production (Figure 2). The in silico analyses suggested that the accumulation of succinate, malate, and oxaloacetate was associated with a high activation of the glyoxylate shunt via isocitrate lyase (ICL). Additionally, the anaplerotic reaction catalyzed by the phosphoenolpyruvate carboxylase (PPC) acted as the main contributor of oxaloacetate, which along with glutamate, generates arginine, the C5 precursor for CA production. Thus, the simulations showed higher probabilities for fluxes through aspartate transaminase (ASPTA) and a lumped reaction summarizing the steps of arginine production (LMPD_33_arg-L_c, Figure 3). The obtained flux distributions support the hypothesis that an increased flux of G3P favors its condensation with arginine (via CEAS), promoting a higher CA synthesis rate as a consequence of higher activity in the glycolysis, pentose phosphate shunt, and anaplerotic reactions, such as ICL and PPC.

**Figure 4:**
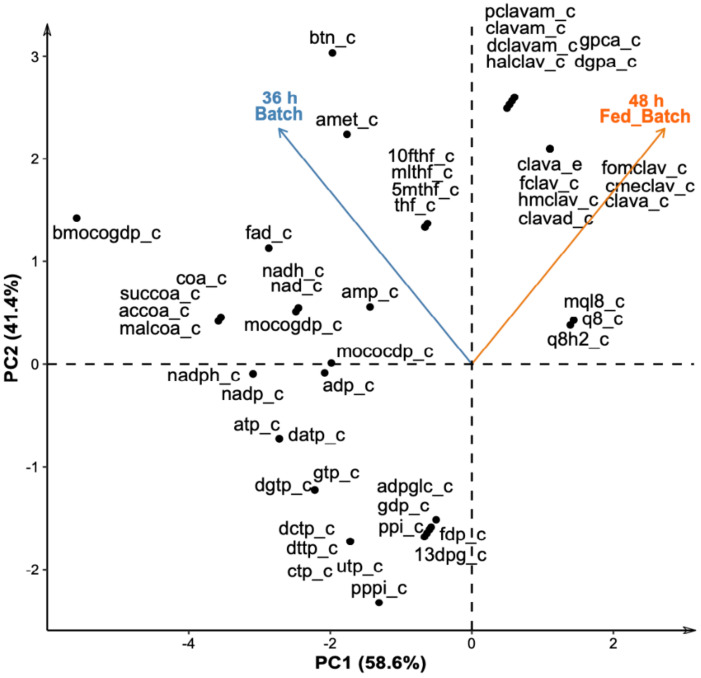
PCA of shadow price changes per metabolite in batch and fed-batch operation mode of *S. clavuligerus* cultivation. PC1: Principal component 1, PC2, Principal component 2.

The shadow prices were used to analyze the effect of accumulation or depletion of metabolites in the CA production through the deviation of steady-state. As in the case of flux distributions, the shadow prices were computed for each metabolite in the metabolic network for both stages, batch and fed-batch (Table S2). Therefore, the size of the data matrix was 1552 metabolites × 2 datasets (batch and fed-batch). This matrix contained the shadow prices of the 1552 metabolites in both conditions. Although dimensionality reduction techniques such PCA are not usually done for two sets of conditions, we decided to apply this analysis for a clear visualization of the most important metabolites contributing to the objective function for each experimental condition, as shown in Figure 4.

Blue vectors in opposite directions indicate that shadow price profiles in the metabolic network differ under batch and fed-batch operations. Therefore, the sclav_red model predicted a different use of the metabolites for CA production during the batch and fed-batch operation modes. The 70 metabolites with the highest changes in the shadow prices are represented in Figure 4. Co-factors and energetic metabolites, such as tetrahydrofolate, biotin, S-adenosyl-methionine, NAD^+^, NADP^+^, ATP, GTP, and CoA, were predicted to have high changes in shadow prices values. As an example, the shadow price values of biotin (btn[c]) were 3.0 and 0.28 for batch and fed-batch modes, respectively. Both positive values indicate an important role of biotin on CA biosynthesis since biotin is a key cofactor that maintains metabolic homeostasis. It is essential for the correct operation (carboxylation) of fatty acids, the TCA cycle, and amino acids metabolism [71,72]. Given the connections between the TCA, amino acid metabolism, and CA biosynthesis, biotin might have an indirect influence on CA production.

Phosphate limitation required for activation of CA biosynthesis affects ATP availability. The negative shadow prices for ADP/ATP and NADP^+^/NADPH in both conditions (Figure 4) suggest that CA biosynthesis coexisted with the reduction in ATP generation, and conversely, the high synthesis rate of those cofactors was connected with low or no CA production. It has been reported that one of the biological roles of antibiotics synthesis, apart from defense, is to adjust the ATP synthesis/consumption in phosphate scarcity conditions [73]. Similarly, the positive shadow prices of metabolites of the clavam pathway imply that high CA synthesis rates also favor the production of metabolites beyond the clavaminic acid bifurcation, i.e., the 5-S clavams compounds.

Table 3 lists some relevant metabolites that belong to the central metabolism with their respective shadow price value. Like the observed in the flux distribution and the experimental data, metabolic intermediates of TCA cycle, such as succinate, oxaloacetate, malate, pyruvate, citrate, and fumarate, had positive shadow prices during the phase of CA production, i.e., fed-batch stage under phosphate limitation. This suggests that these metabolites positively influence CA biosynthesis, and therefore, the intracellular accumulation of some TCA cycle intermediates would improve CA synthesis. Conversely, the accumulation of intermediates of the pentose phosphate pathway negatively influences the CA production since those metabolites are associated with nucleotide synthesis and anabolism for biomass production but not secondary metabolism. As in the case of TCA intermediates, shadow prices of amino acids related to the synthesis of the C-5 precursor of CA, such as glutamine, arginine, asparagine, aspartate, and glutamate, indicated a positive influence on CA biosynthesis.

## 4. Conclusions

In this work a reduced model of *S. clavuligerus* metabolism was successfully constructed and used as an analysis tool for the study of metabolic scenarios characterized by high CA production. The reduced reconstruction used a bottom-up approach starting from a high-quality genome-scale model and retained the predictive capacity of the large model while requiring less computational power for reaching a feasible solution. This is the first reduced genome-scale model of *S. clavuligerus* developed and validated using experimental data. A reduced network of *S. clavuligerus* would make possible a further construction of a kinetic genome-scale network, something infeasible for a large-scale metabolic model.

The metabolic scenario observed for the cultivation explored in this work resembles the biomass and CA productivity obtained in stirred tank bioreactors but without the shear stress effects associated with the axial-impeller equipment. The high volumetric gas transfer rate of the 2D rocking bioreactor favored the metabolic fluxes towards the clavams pathway under phosphate-limited conditions. The in silico analyses using the reduced model confirmed the interrelation between the accumulation of acetate, pyruvate and the TCA cycle intermediates (succinate, oxaloacetate, and malate) and CA production. The shadow prices provided the first view of those metabolites negatively and positively associated with the scenarios of low and high production, the first attained during the early stages of cultivation and the latter under phosphate limitation.

## Figures and Tables

**Table 1:** Comparison of rate predictions of sclav_red with experimental data in continuous cultures of *S. clavuligerus* at different dilution rates (D) obtained by Ramirez-Malule et al. [20].

Reaction	D = 0.045			D = 0.035			D = 0.050		
	FBA	Exp.	*SE*	FBA	Exp.	*SE*	FBA	Exp.	*SE*
Growth (h^−1^)	0.039	0.045	0	0.034	0.035	0	0.044	0.050	0
O_2_	−0.375	−1.665	1.662	−0.322	−1.621	1.686	−0.511	−1.848	1.787
Glycerol	−0.695	−1.110	0.172	−0.639	−0.968	0.108	−0.728	−0.728	0
CO_2_	0.067	0.067	0	0.023	0.023	0	0.253	0.253	0
Clavulanate	0.017	0.398	0.145	0.015	0.357	0.117	0.020	0.435	0.172
Phosphate	0.000	N/A	N/A	0.000	N/A	N/A	0.000	N/A	N/A
Glutamate	−0.350	N/A	N/A	−0.310	N/A	N/A	−0.400	N/A	N/A
*MSE*	0.396			0.382			0.392		

Flux units in (mmol.(gDCW *h)^−1^). FBA: flux balance analysis data for the sclav_red model, Exp: experimental data from [20], N/A: no experimental data available.

**Table 2:** Comparison between experimental and simulated rates in batch and fed-batch cultivations of *S. clavuligerus*.

Reaction	Batch (36 h)			Fed-Batch (48 h)		
	FBA	Exp.	*SE*	FBA	Exp.	*SE*
Growth (h^−1^)	0.042	0.042 ± 0.004	0	0.031	0.031 ± 0.003	0
O_2_	−0.512	−1.350	0.702	−0.776	−1.2	0.180
Glycerol	−0.182	−0.182	0	−0.457	−0.47	0
CO_2_	0.639	1.640	1.002	0.610	1.4	0.623
Clavulanate	0.002	0.002	0	0.004	0.004	0
Succinate	0.014	0.014	0	0.257	0.007	0.063
Oxaloacetate	0.004	0.004	0	0	0.003	0
Malate	0	0.001	0	0	0	0
Pyruvate	0	0	0	0.038	0.005	0.001
Acetate	0	0	0	0	0	0
*MSE*			1.700			0.860

Flux units in (mmol.(gDCW *h)^−1^). FBA: flux balance analysis data, Exp: experimental data. All fluxes in the table were experimentally quantified and soft constrained for the in silico studies. Growth rate was set as a hard constraint.

**Table 3:** Shadow price values of some metabolite intermediates of the central carbon metabolism in *S. clavuligerus*.

Metabolites	Shadow Prices (CPLEX)		
	Batch (36 h)	Fed-Batch (48 h)	Pathway
α-ketoglutarate	0	0.05	Glycolysis and TCA cycle
Pyruvate	0	0.02	
Citrate	0	0.05	
Isocitrate	0	0.05	
Fumarate	0	0.02	
Acetate	0	0.02	
Succinate	0	0.02	
Oxaloacetate	0	0.02	
Malate	0	0.02	
Erythrose 4 phosphate	0	−0.40	Pentose phosphate pathway
Fructose 6 phosphate	0	−0.38	
Xylulose 5 phosphate	0	−0.39	
Sedoheptulose 7-phosphate	0	−0.37	
L-glutamate	0.5	0.05	Amino acids synthesis
L-glutamin	0.5	0.05	
L-arginine	1.5	0.05	
L-asparagin	0.5	0.02	
L-aspartate	0.5	0.02	

## Data Availability

Not applicable.

## Supplementary Materials

The following are available online at https://www.mdpi.com/article/10.3390/bioengineering8080103/s1, File S1: reduced model of *S. clavuligerus* in mat format, File S2: Plot of 64 fluxes distributions during two metabolic states in batch and fed-batch operations, File S3: Supplementary scripts. Figure S1: intracellular and extracellular flux sampling distributions representing metabolic scenarios in batch (36 h) and fed-batch (48 h) culture mode of *S. clavuligerus*. Table S1: List of modifications to the curated sclav_red model, Table S2: FBA, FVA, and Shadow Prices obtained for the two simulated metabolic states during batch and fed batch cultivation. Text S1: Explorative gene knockouts to improve the CA production.
