# Supplementary material for: TCA Cycle and Its Relationship with Clavulanic Acid Production: A Further Interpretation by Using a Reduced Genome-Scale Metabolic Model of Streptomyces clavuligerus
Source: Bioengineering (Basel). 2021 Jul 22;8(8):103. doi: 10.3390/bioengineering8080103 (PMC8389198; doi:10.3390/bioengineering8080103)
Supplement: Supplementary file 1 [file bioengineering-08-00103-s001.zip › bioengineering-1226454-Text S1_final.pdf]

bioengineering

MDPI

Article

TCA Cycle and Its Relationship with Clavulanic Acid Production: A Further Interpretation by Using a Reduced Genome-Scale Metabolic Model of *Streptomyces clavuligerus*

Howard Ramirez-Malule [1,*], Víctor A. López-Agudelo [1], David Gómez-Ríos [2], Silvia Ochoa [2], Rigoberto Ríos-Estepa [3], Stefan Junne [4] and Peter Neubauer [4]

1 Escuela de Ingeniería Química, Universidad del Valle, Cali 25360, Colombia; valonso.lopez@udea.edu.co
2 Grupo de Investigación en Simulación, Diseño, Control y Optimización de Procesos (SIDCOP), Departamento de Ingeniería Química, Universidad de Antioquia UdeA, Medellín 050010, Colombia; dandres.gomez@udea.edu.co (D.G.-R.); silvia.ochoa@udea.edu.co (S.O.)
3 Escuela de Biociencias, Universidad Nacional de Colombia sede Medellín, Medellín 050010, Colombia; rriose@unal.edu.co
4 Chair of Bioprocess Engineering, Institute of Biotechnology, Technische Universität Berlin, D-13355 Berlin, Germany; stefan.junne@tu-berlin.de (S.J.); peter.neubauer@tu-berlin.de (P.N.)
* Correspondence: howard.ramirez@correounivalle.edu.co

Supplementary Text 1

Although the *MSE* for the model iDG1237 is lower than the model sclav_red, both *MSE* are in the same order of magnitude, which means that both models have similar performance in the prediction of this specific experimental scenario (Table 1 main manuscript). Undoubtedly, both models accurately predict the growth rate values by using the experimental constraints. However, the sclav_red model predicts growth rates closer than iDG1237 (Figure S1) [1]. Likewise, both models can predict production of CA but at low-rate values (Table 1 main manuscript).

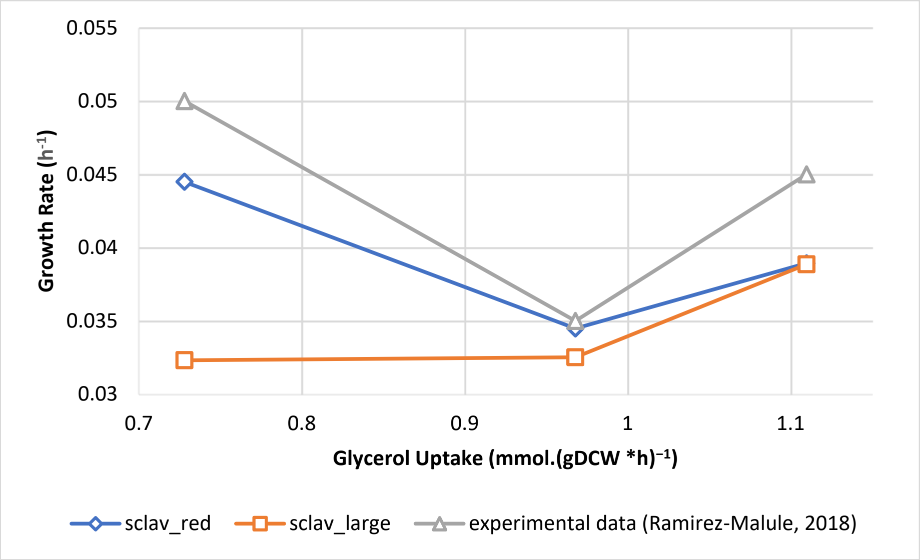

Figure S1. Comparison of growth rate predictions of sclav_red and iDG1237.

An important aspect to know is that we identified glutamate as an important metabolite for the growth and CA production of *S. clavuligerus* as both metabolic models

are very sensible to this metabolite. The robustness analysis showed that both models need glutamate for growth (Figure S2). While sclav_red improves growth rates when glutamate consumption increases until 20 mmol (gDCW.h)$^{-1}$, the iDG1237 model predicts negative effect on growth rate at glutamate uptake rates above 1.9 mmol (gDCW.h)$^{-1}$.

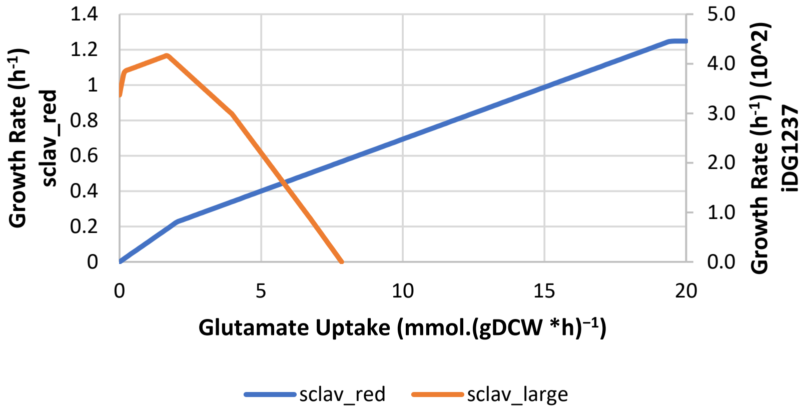

Figure S2. Robustness analysis of glutamate uptake.

Even with differences in glutamate requirements, the sclav_red constitutes a condensed representation of the metabolic capabilities of *S. clavuligerus*, with special focus on central carbon metabolism and CA biosynthesis. This model constitutes a more computationally efficient metabolic model version compared to *i*DG1237 that can be applied for exploring metabolic phenotypes using constraint-based methods that require more computational power like flux sampling [2], thermodynamically constrained stoichiometric models that use energy balance as a constraint [3] or large-scale kinetic modeling [4].

In silico Gene Knockout Mutants in S. clavuligerus

Metabolic models have been successfully used for synthetic biology of organisms and to predict rational strain design. The reduced sclav_red model was used for exploration of potential genetic interventions aimed to improve CA production in the experimental conditions of this work. The bi-level optimization approach OptKnock [5] was used to identify a set of reactions suitable to be removed from the metabolic network to redistribute the carbon fluxes leading to CA biosynthesis, yielding higher CA production rates compared to the obtained with the wild-type strain. The application of Optknock suggested four potential mutant strains that would be constructed through three genetic interventions (Table S1, and FileS3). As shown in Table S1, mutants No.1 and 4 have higher production rates of CA, 0.012 and 0.15 mmol (gDCW.h)$^{-1}$, respectively. Although there are not gene essentiality reports for *S. clavuligerus*, the genes encoding these enzymes are classified as non-essential enzymes for *E. coli* according to the Online GEne Essentiality (OGEE) Database [6], which suggests that corresponding knockout strains may be viable. All the proposed mutants induce the accumulation of central metabolism intermediates like G3P, oxaloacetate, isocitrate and 2-oxoglutarate, which is consistent with the results of sensitivity analysis with shadow prices and flux distributions. Those genetic interventions might help to improve the CA titers in fed-batch cultivations of *S. clavuligerus* with phosphate limitation.

Table S1. Mutant strains of *S. clavuligerus* proposed in this study by using the OptKnock approach.

No.	Proposed mutant strains	CA production [mmol.(gDCW*h)$^{-1}$]	Growth Rate (h^{-1})
1	2-Oxogluterate dehydrogenase (*sucA, sucB, lpd*), Isocitrate dehydrogenase (*icd*), and Phosphoenolpyruvate carboxylase (*ppc*)	0.012	0.04
2	Isocitrate dehydrogenase (*icd*), Phosphoenolpyruvate carboxylase (*ppc*), and Succinyl-CoA synthetase (*sucD, suc*C)	0.007	0.04
3	2-Oxogluterate dehydrogenase (*sucA, sucB, lpd*), Isocitrate dehydrogenase (*icd*), and Transketolase (*tktA*)	0.006	0.04
4	Glucose 6-phosphate dehydrogenase (*zwf*), Glyceraldehyde-3-phosphate dehydrogenase (*gap*), and Isocitrate dehydrogenase (*icd*)	0.015	0.035

References

1. Ramirez-Malule, H.; Junne, S.; Nicolás Cruz-Bournazou, M.; Neubauer, P.; Ríos-Estepa, R. *Streptomyces clavuligerus* shows a strong association between TCA cycle intermediate accumulation and clavulanic acid biosynthesis. *Appl. Microbiol. Biotechnol.* **2018**, *102*, 4009–4023, doi:10.1007/s00253-018-8841-8.
2. Herrmann, H.A.; Dyson, B.C.; Vass, L.; Johnson, G.N.; Schwartz, J.-M. Flux sampling is a powerful tool to study metabolism under changing environmental conditions. *Npj Syst. Biol. Appl.* **2019**, *5*, 1-8, doi:10.1038/s41540-019-0109-0.
3. Niebel, B.; Leupold, S.; Heinemann, M. An upper limit on Gibbs energy dissipation governs cellular metabolism. *Nat. Metab.* **2019**, *1*, 125–132, doi:10.1038/s42255-018-0006-7.
4. Tokic, M.; Hatzimanikatis, V.; Miskovic, L. Large-scale kinetic metabolic models of *Pseudomonas putida* KT2440 for consistent design of metabolic engineering strategies. *Biotechnol. Biofuels* **2020**, *13*, 1-19, doi:10.1186/s13068-020-1665-7.
5. Burgard, A.P.; Pharkya, P.; Maranas, C.D. OptKnock: A bilevel programming framework for identifying gene knockout strategies for microbial strain optimization. *Biotechnol. Bioeng*. **2003**, *84*, 647–657, doi:10.1002/bit.10803.
6. Gurumayum, S.; Jiang, P.; Hao, X.; Campos, T.L.; Young, N.D.; Korhonen, P.K.; Gasser, R.B.; Bork, P.; Zhao, X.M.; He, L.J.; Chen, W.H. OGEE v3: Online GEne Essentiality database with increased coverage of organisms and human cell lines. *Nucleic Acids Res.* **2021**, D998–D1003, doi:10.1093/nar/gkaa884.
