# Supplementary figures and images for: TCA Cycle and Its Relationship with Clavulanic Acid Production: A Further Interpretation by Using a Reduced Genome-Scale Metabolic Model of Streptomyces clavuligerus (part 2 of 2)
Source: Bioengineering (Basel). 2021 Jul 22;8(8):103. doi: 10.3390/bioengineering8080103 (PMC8389198; doi:10.3390/bioengineering8080103)

# Citric Acid Cycle (MDH2)

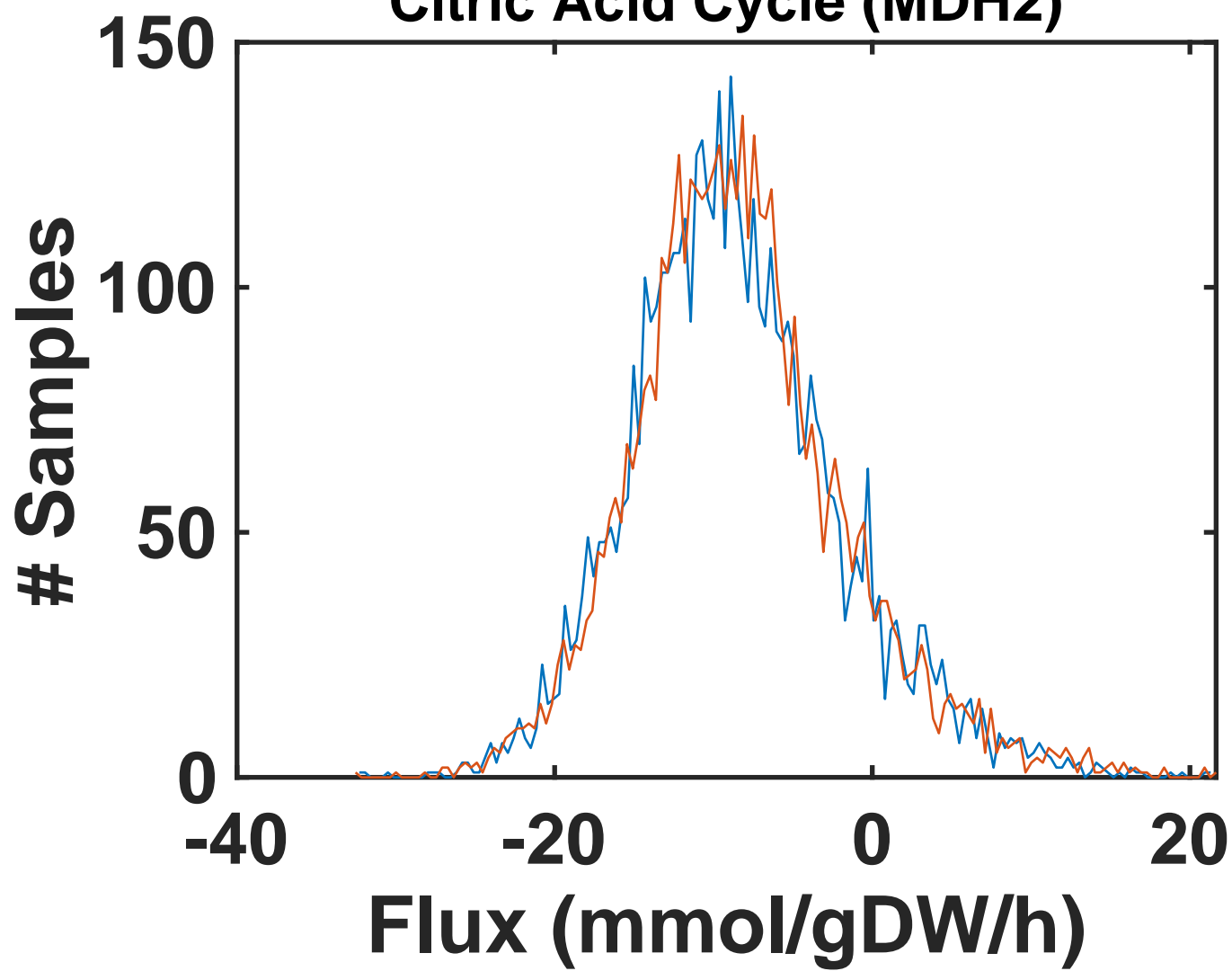

Supplement: Supplementary file 1 [file bioengineering-08-00103-s001.zip › FileS2/figure_sampling_noFVA-MDH2.pdf]

# Citric Acid Cycle (MDH3)

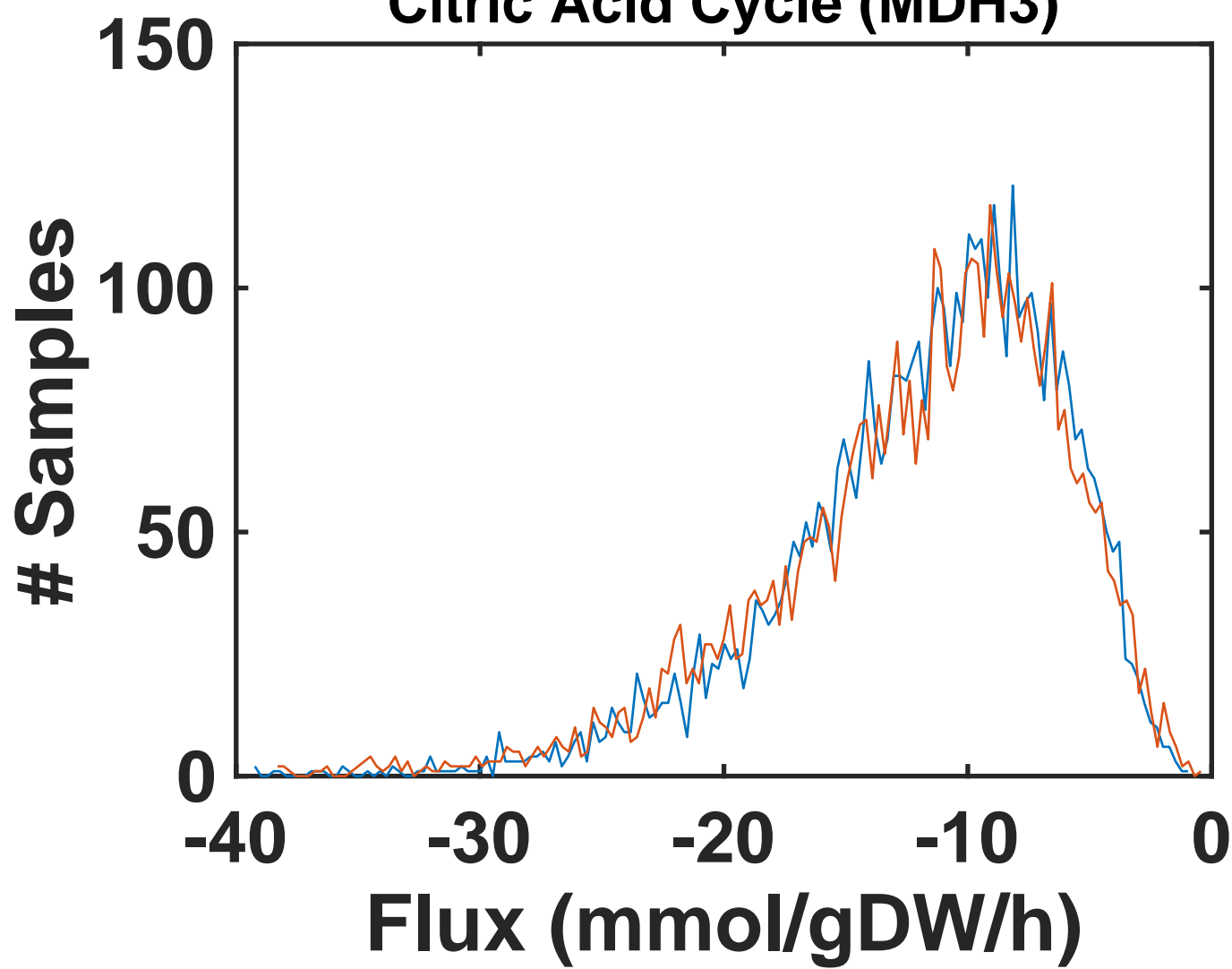

Supplement: Supplementary file 1 [file bioengineering-08-00103-s001.zip › FileS2/figure_sampling_noFVA-MDH3.pdf]

## Anaplerotic Reactions (ME1)

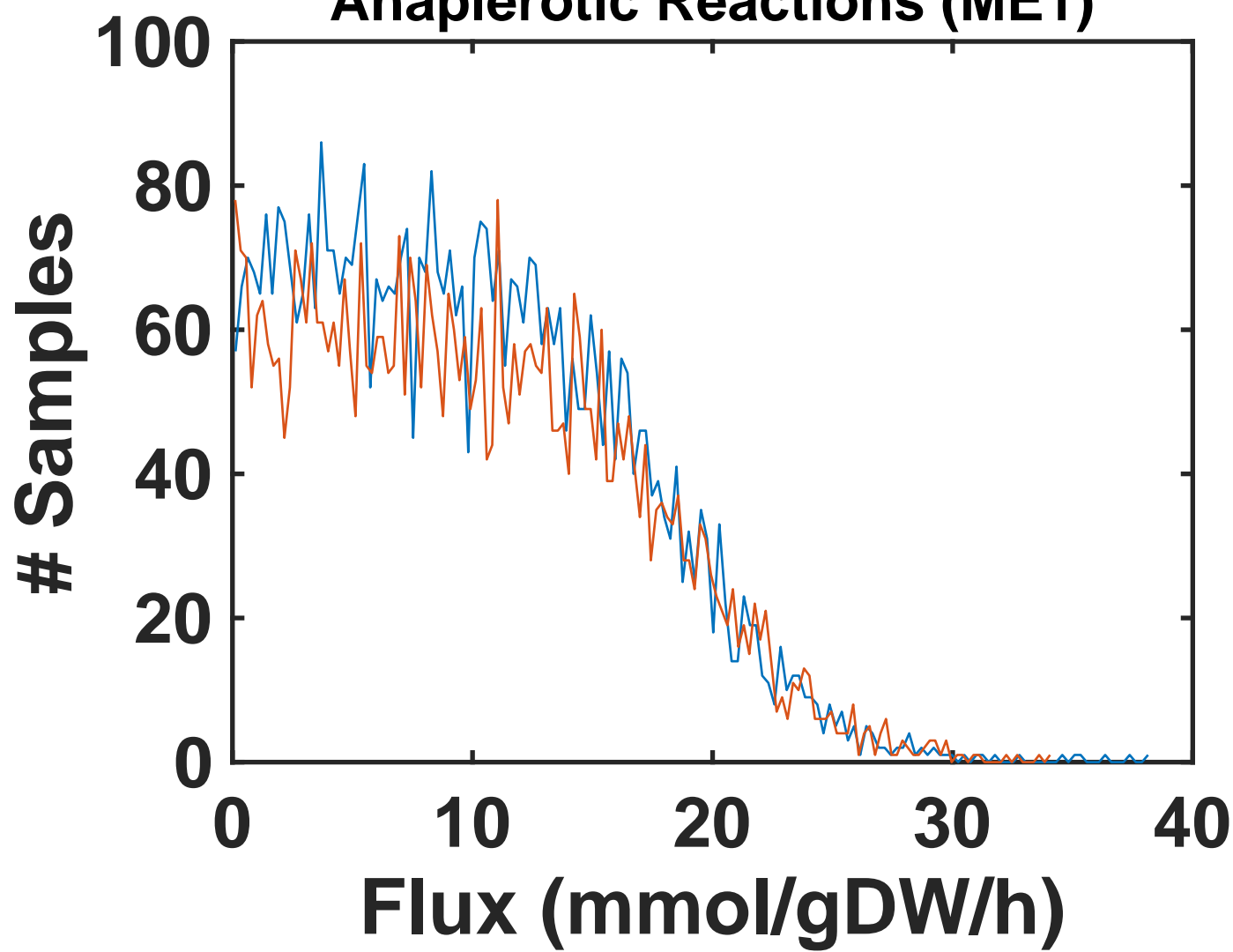

Supplement: Supplementary file 1 [file bioengineering-08-00103-s001.zip › FileS2/figure_sampling_noFVA-ME1.pdf]

## Anaplerotic Reactions (ME2)

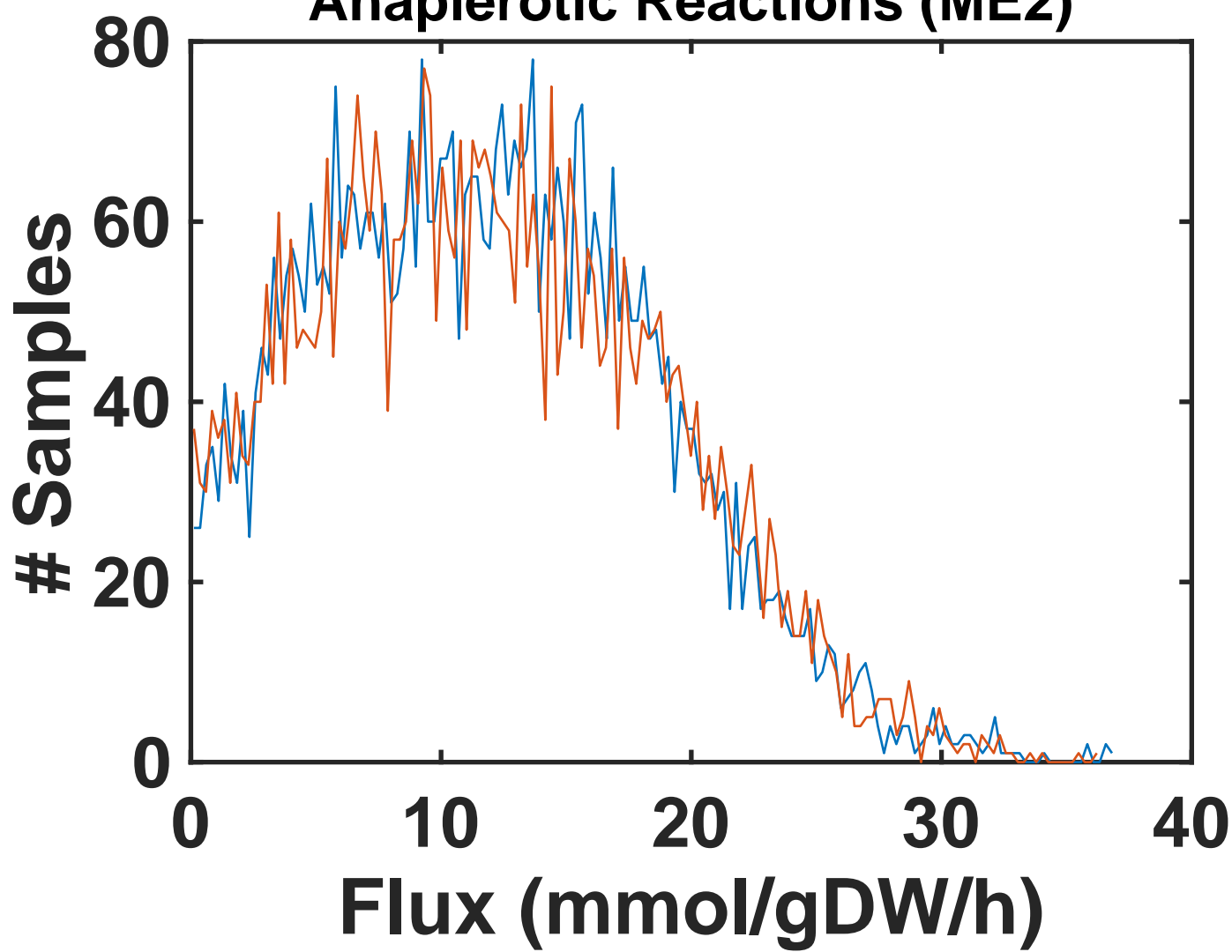

Supplement: Supplementary file 1 [file bioengineering-08-00103-s001.zip › FileS2/figure_sampling_noFVA-ME2.pdf]

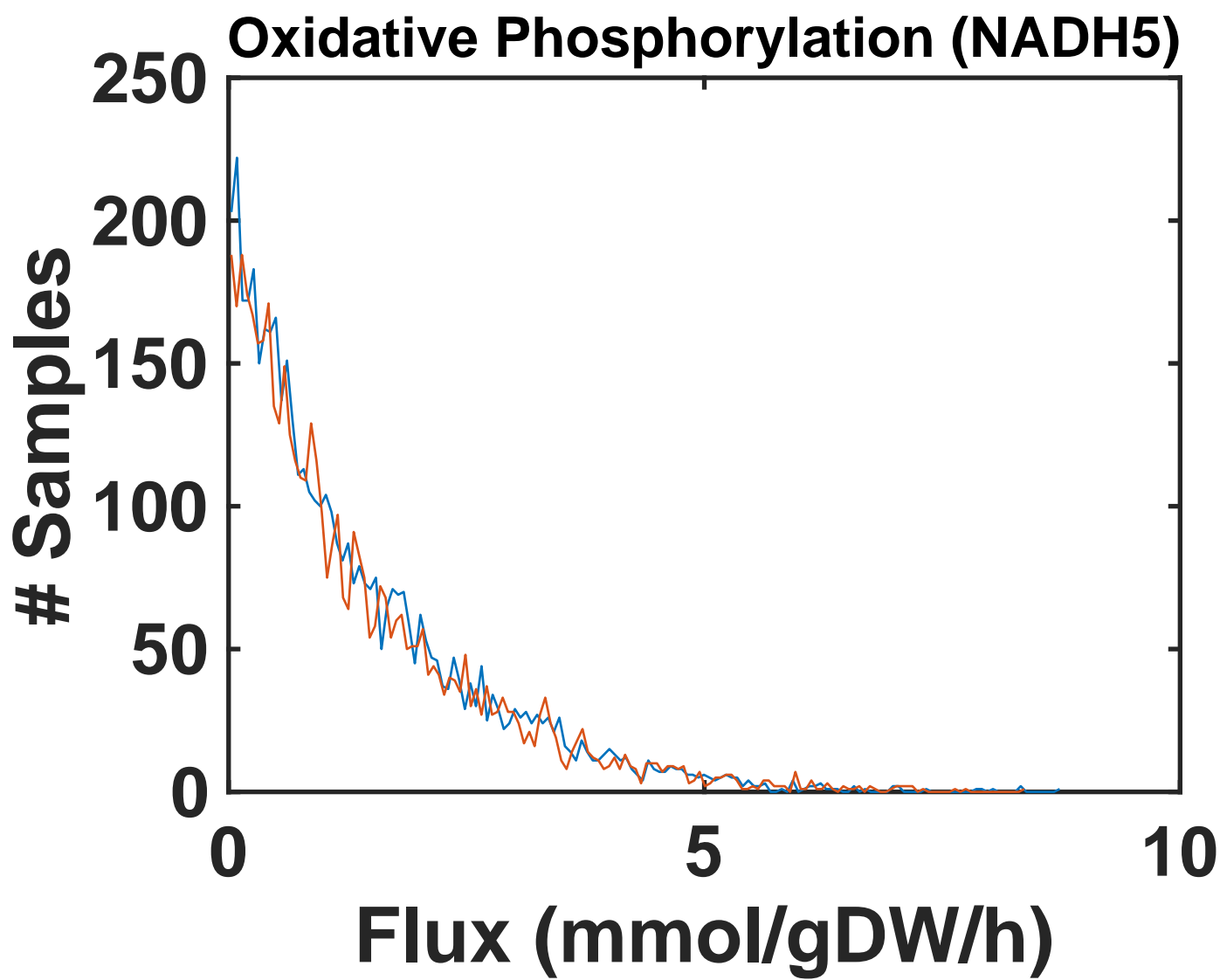

Supplement: Supplementary file 1 [file bioengineering-08-00103-s001.zip › FileS2/figure_sampling_noFVA-NADH5.pdf]

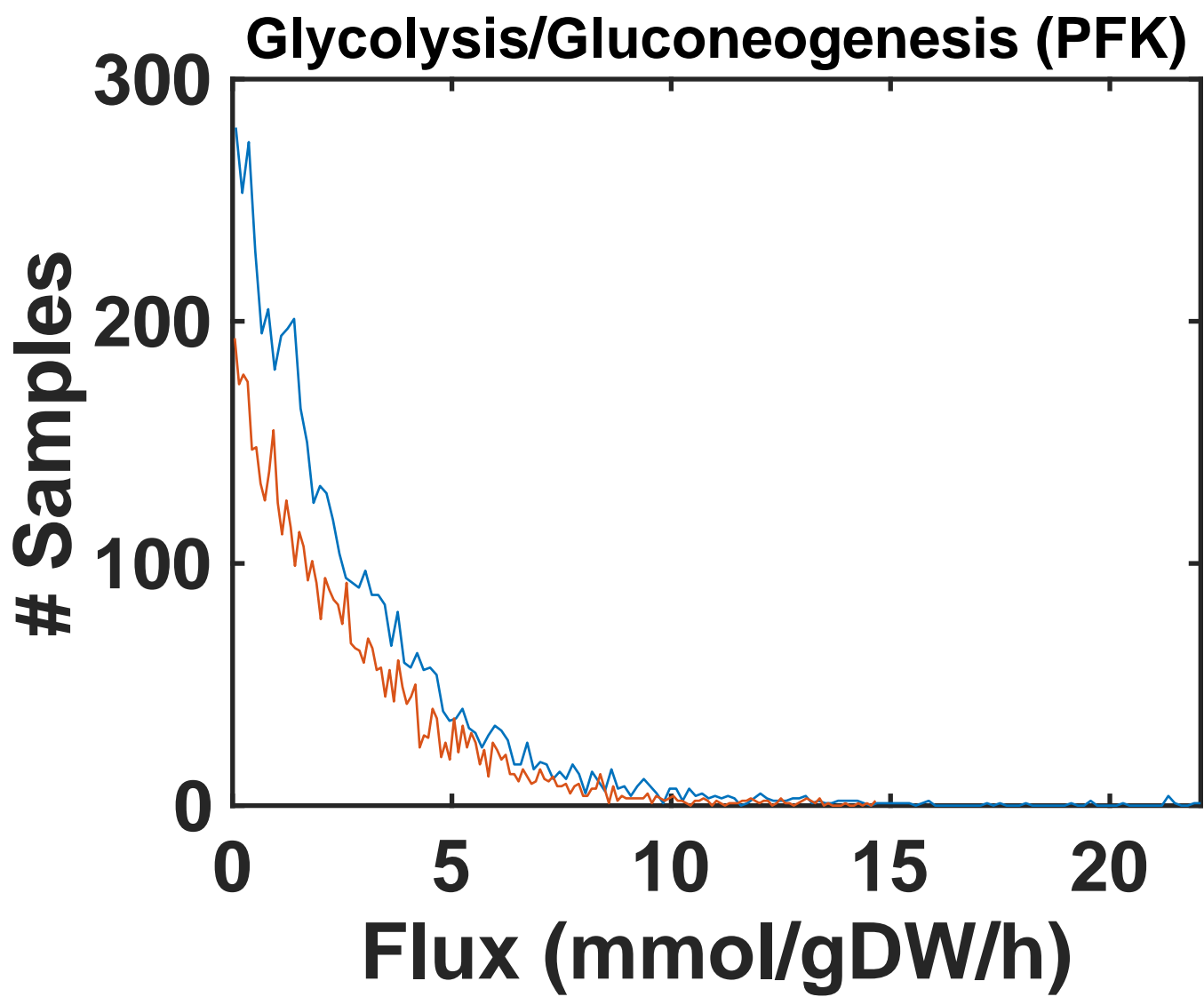

Supplement: Supplementary file 1 [file bioengineering-08-00103-s001.zip › FileS2/figure_sampling_noFVA-PFK.pdf]

## Pyruvate Metabolism (PFL)

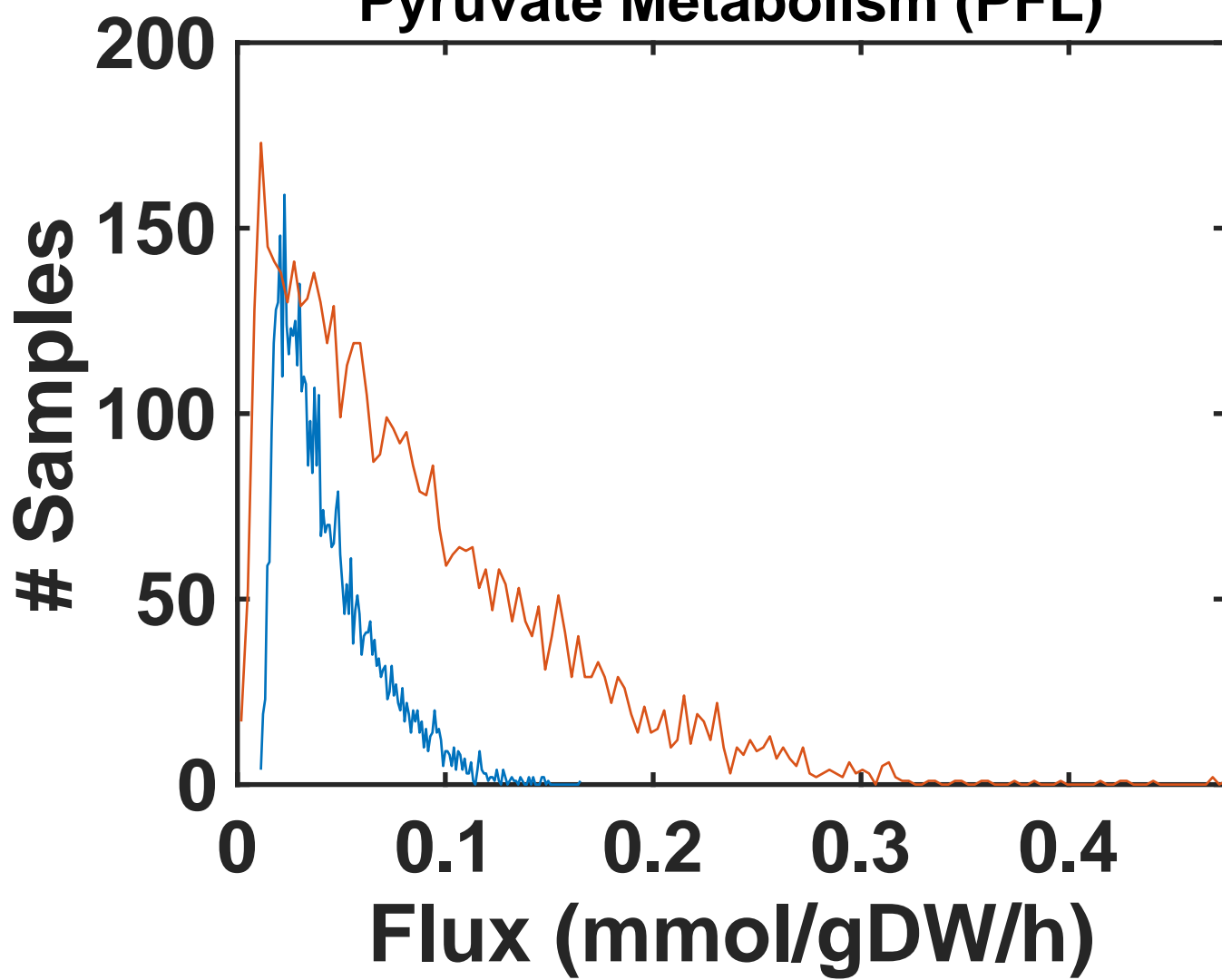

Supplement: Supplementary file 1 [file bioengineering-08-00103-s001.zip › FileS2/figure_sampling_noFVA-PFL.pdf]

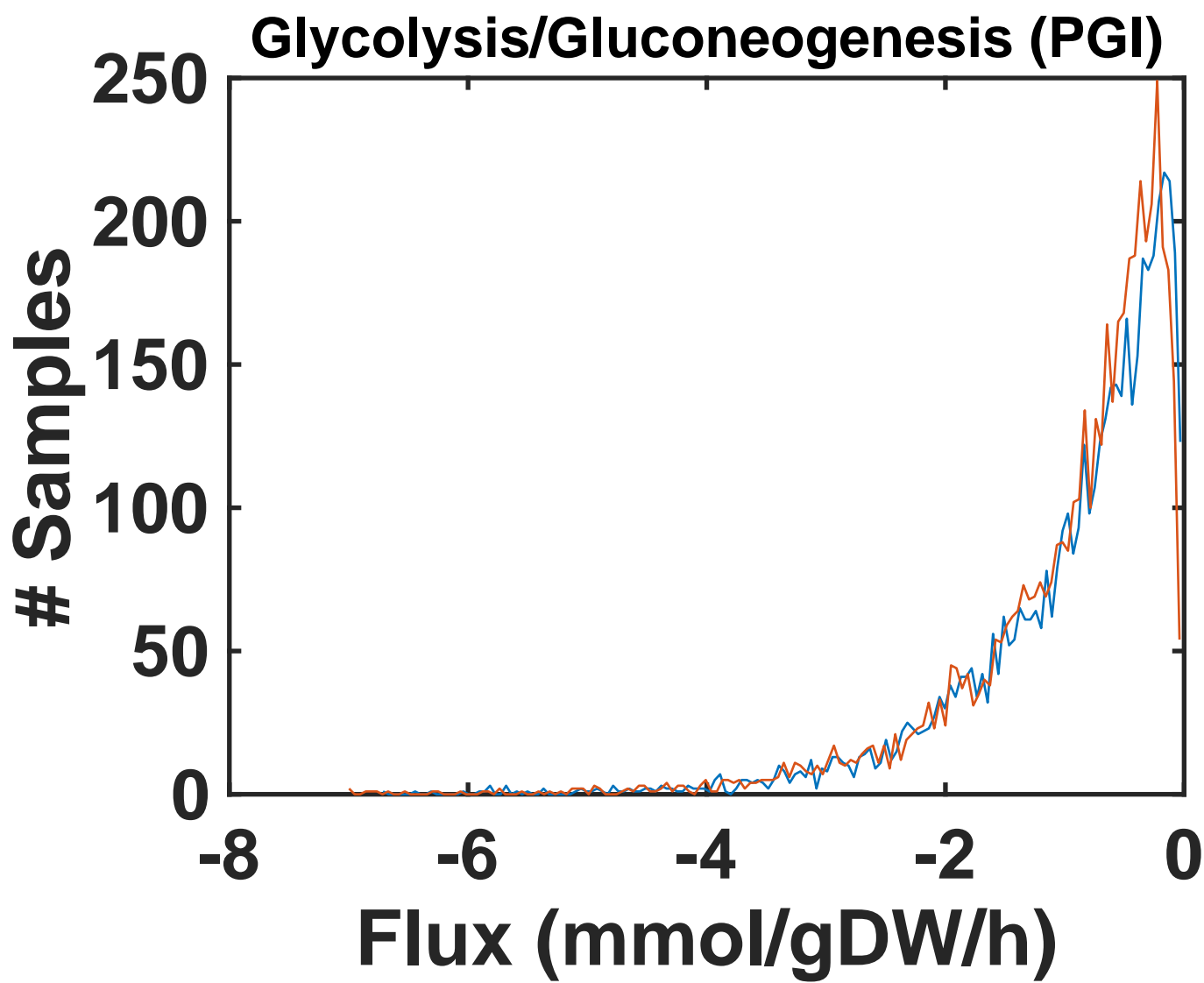

Supplement: Supplementary file 1 [file bioengineering-08-00103-s001.zip › FileS2/figure_sampling_noFVA-PGI.pdf]

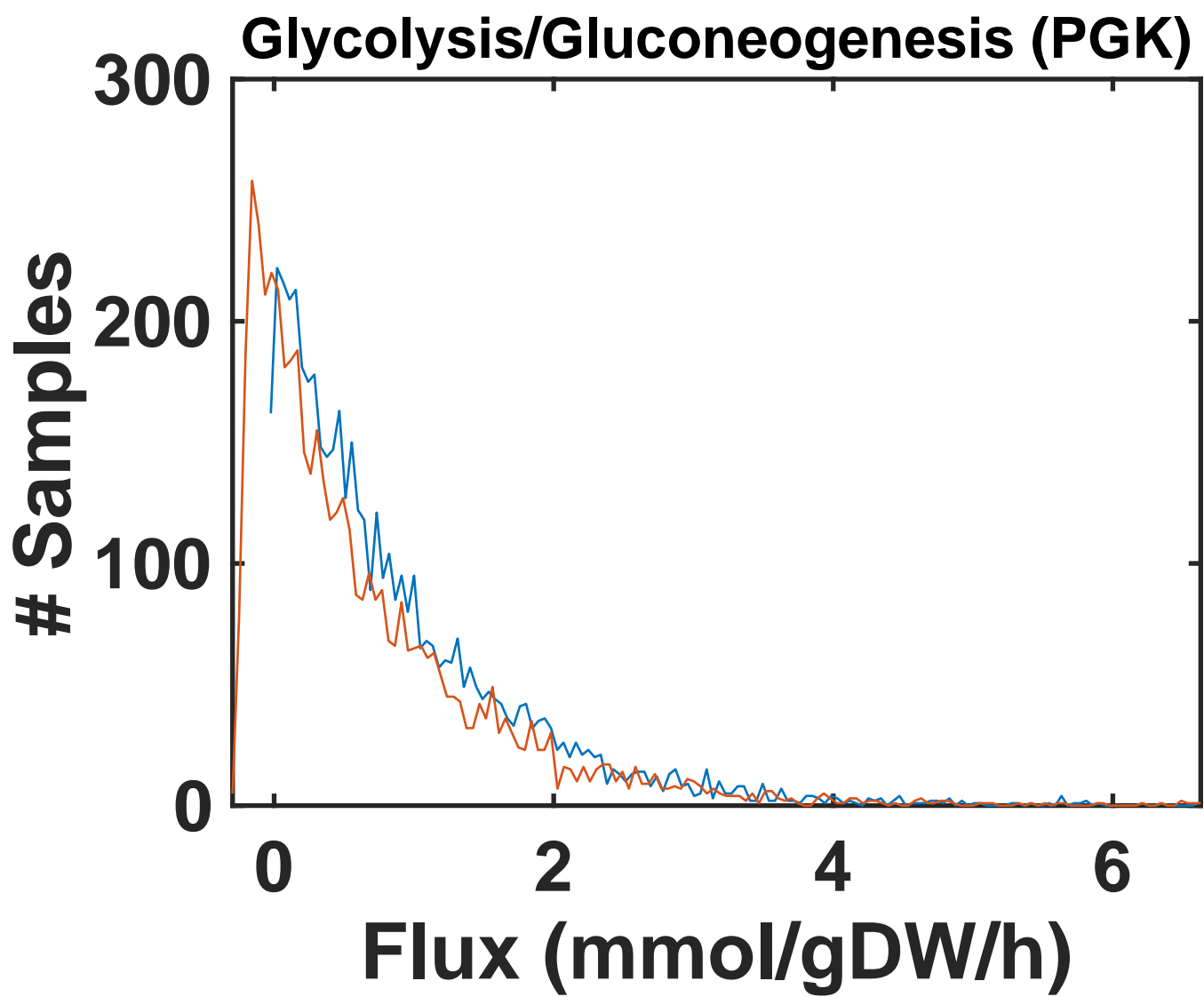

Supplement: Supplementary file 1 [file bioengineering-08-00103-s001.zip › FileS2/figure_sampling_noFVA-PGK.pdf]

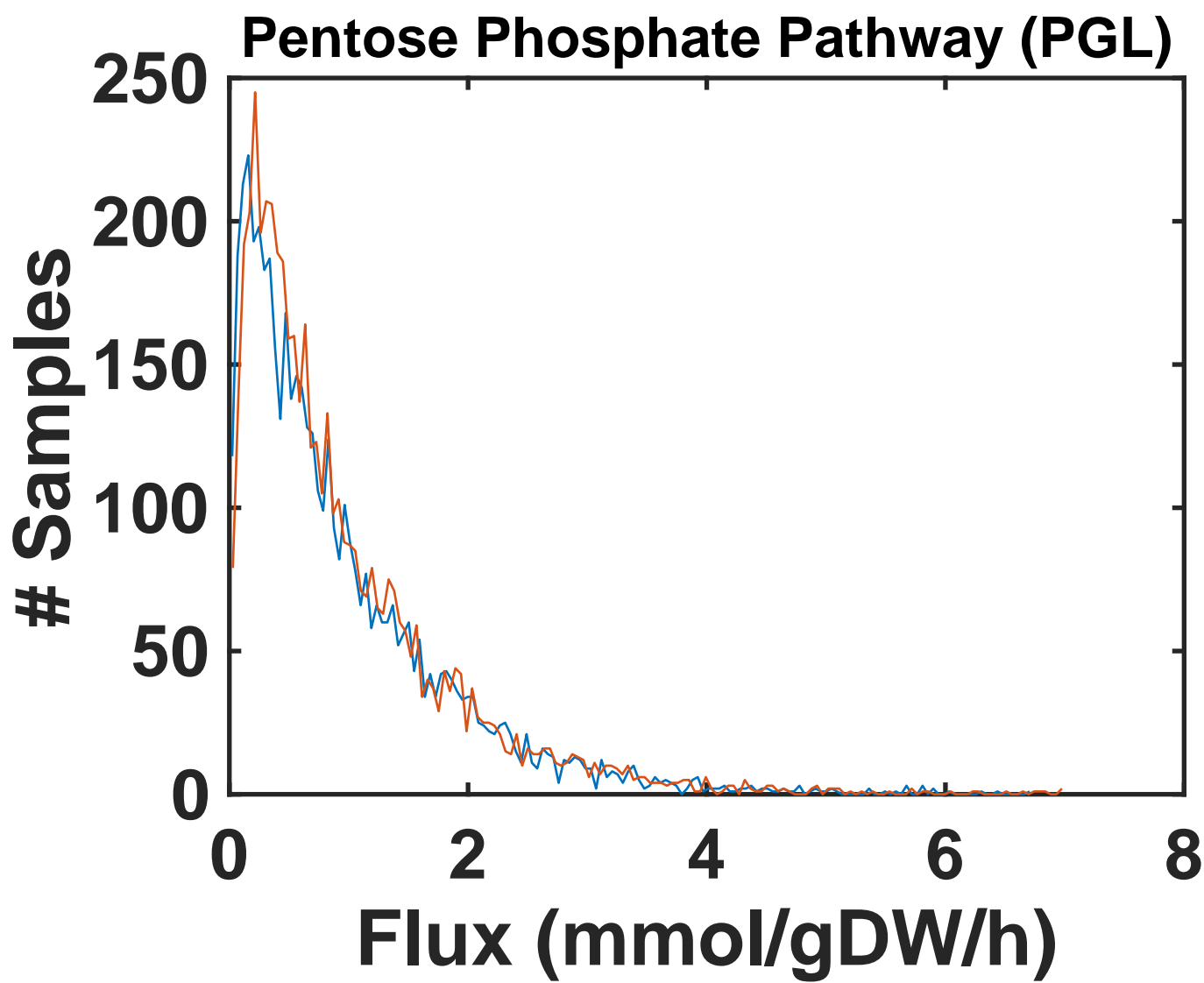

Supplement: Supplementary file 1 [file bioengineering-08-00103-s001.zip › FileS2/figure_sampling_noFVA-PGL.pdf]

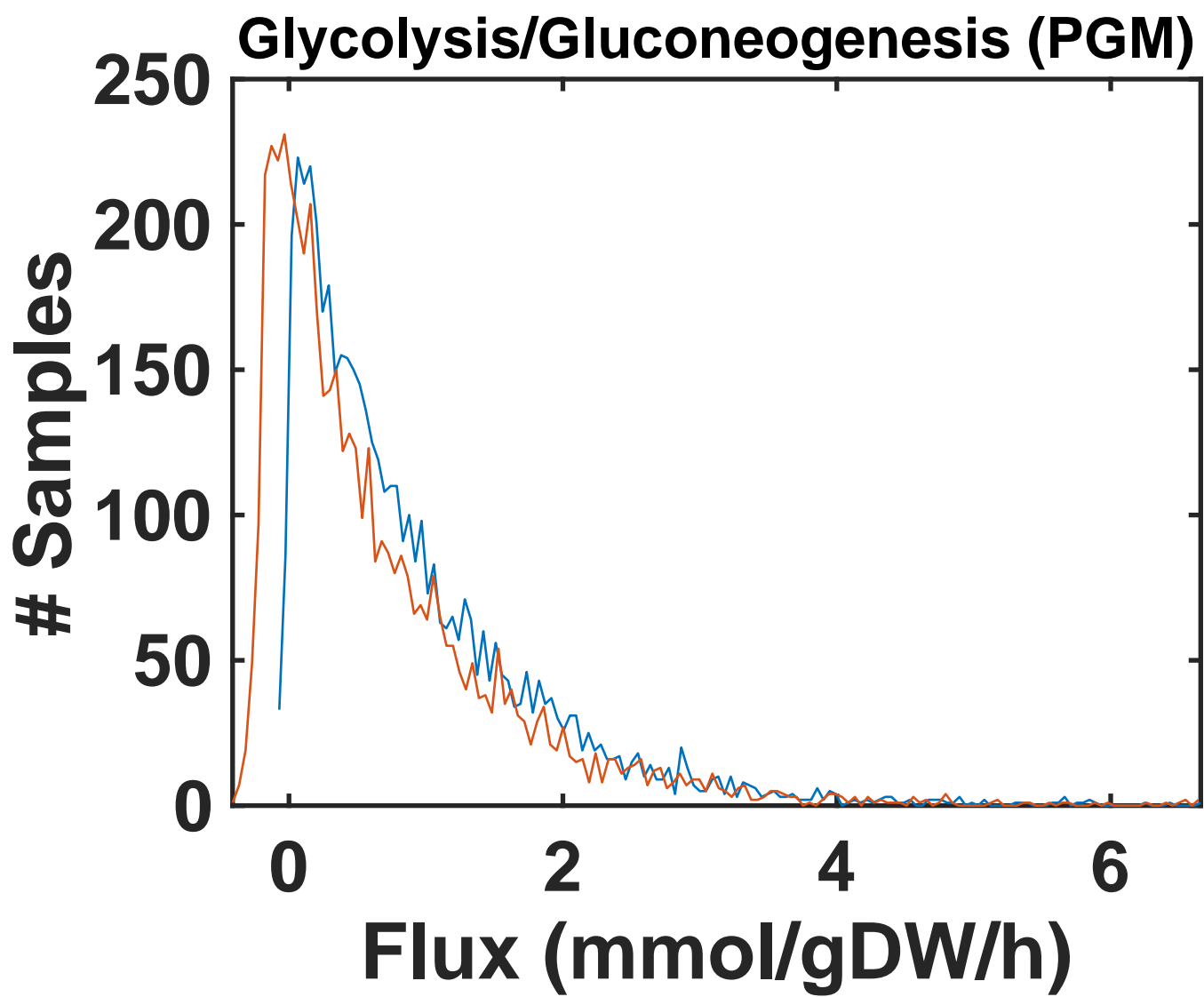

Supplement: Supplementary file 1 [file bioengineering-08-00103-s001.zip › FileS2/figure_sampling_noFVA-PGM.pdf]

## Anaplerotic Reactions (PPC)

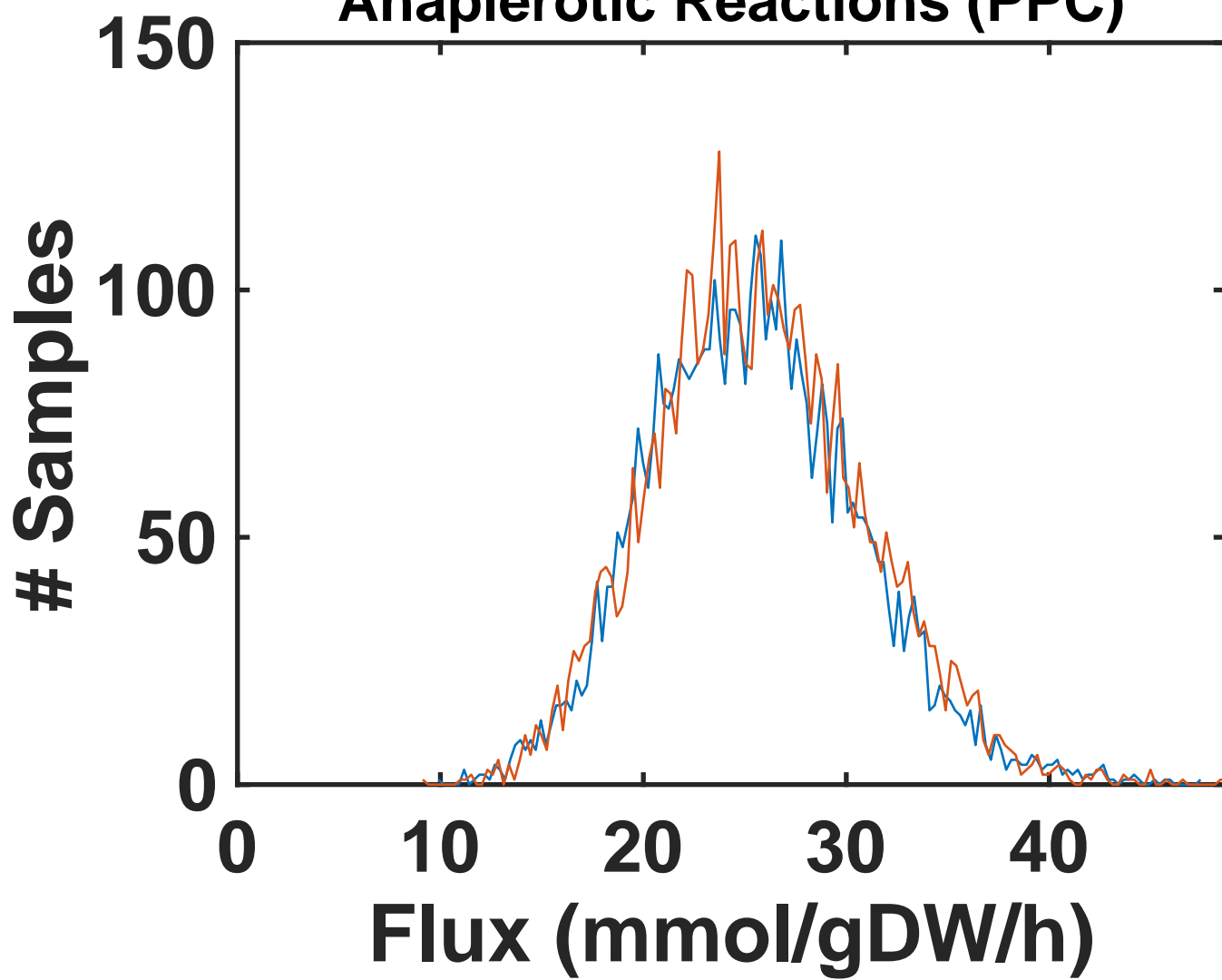

Supplement: Supplementary file 1 [file bioengineering-08-00103-s001.zip › FileS2/figure_sampling_noFVA-PPC.pdf]

## Anaplerotic Reactions (PPCK)

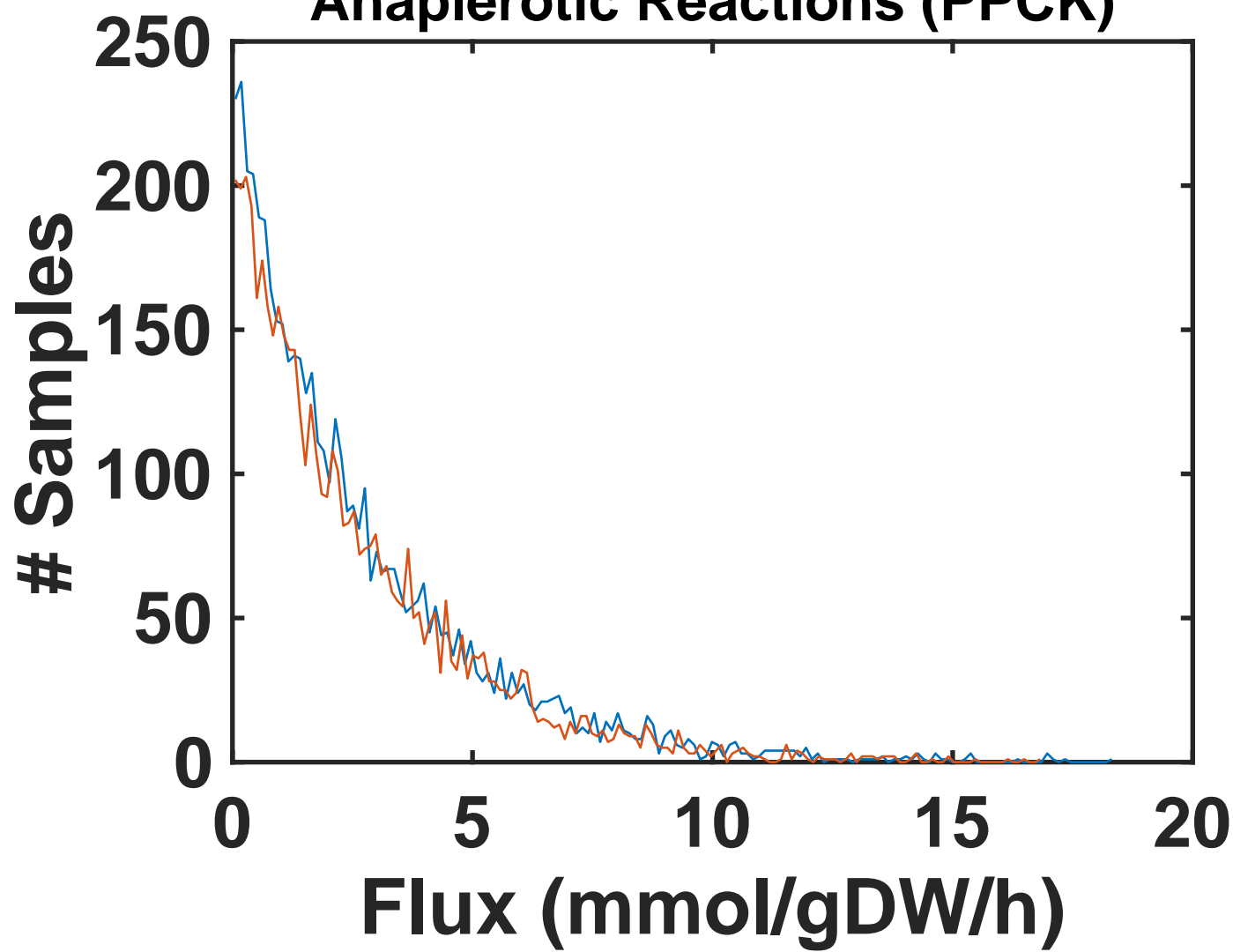

Supplement: Supplementary file 1 [file bioengineering-08-00103-s001.zip › FileS2/figure_sampling_noFVA-PPCK.pdf]

## Pyruvate Metabolism (PTAr)

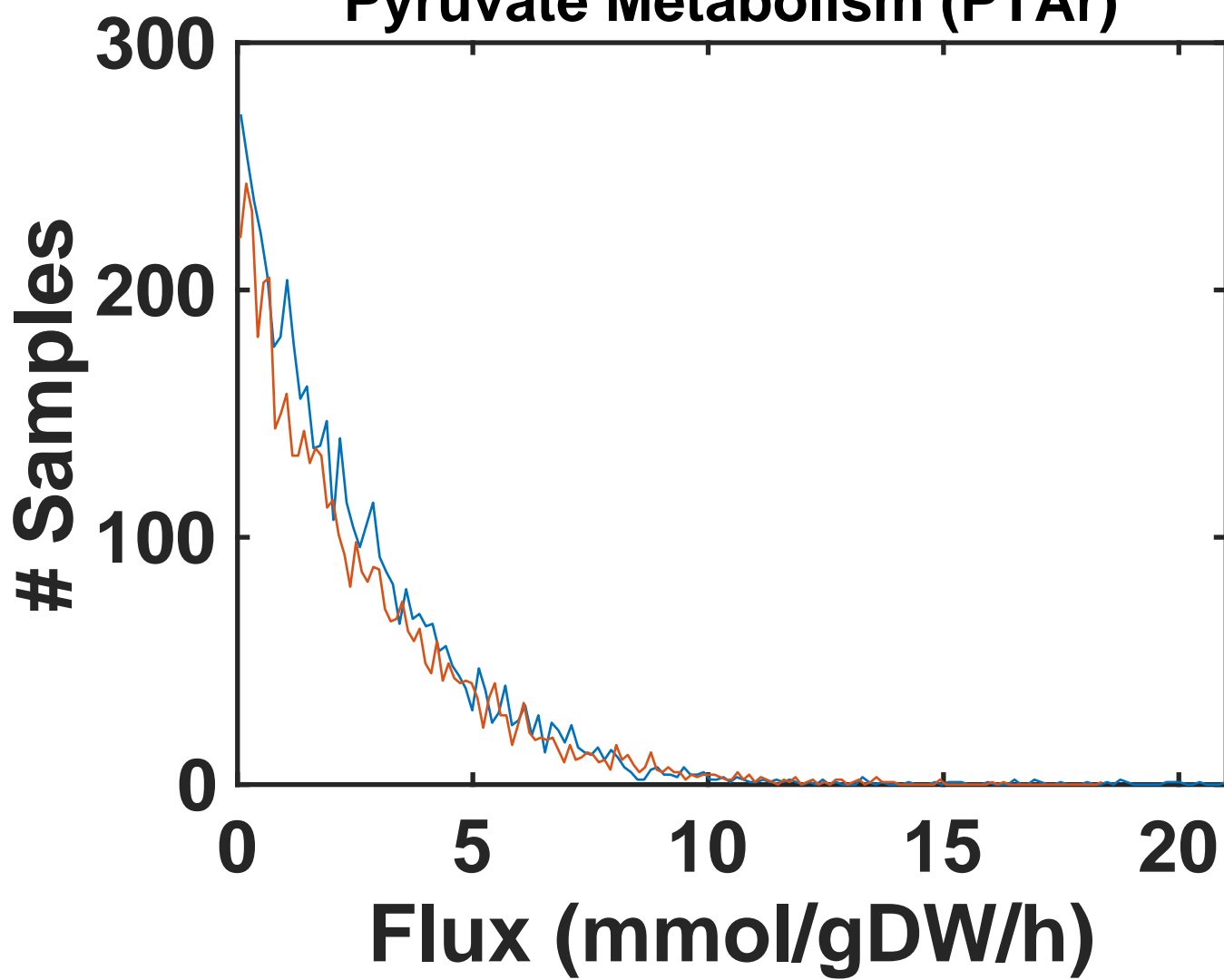

Supplement: Supplementary file 1 [file bioengineering-08-00103-s001.zip › FileS2/figure_sampling_noFVA-PTAr.pdf]

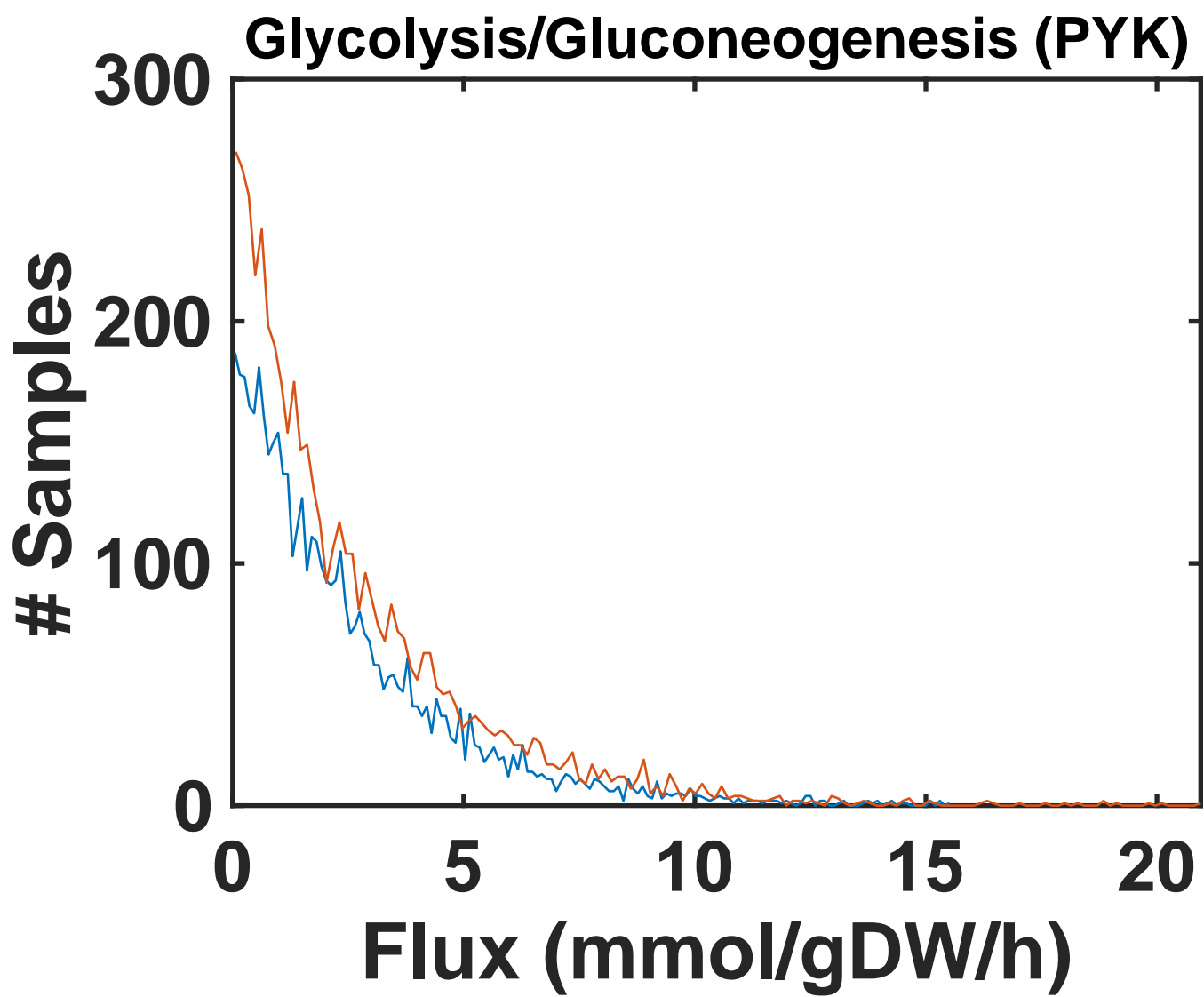

Supplement: Supplementary file 1 [file bioengineering-08-00103-s001.zip › FileS2/figure_sampling_noFVA-PYK.pdf]

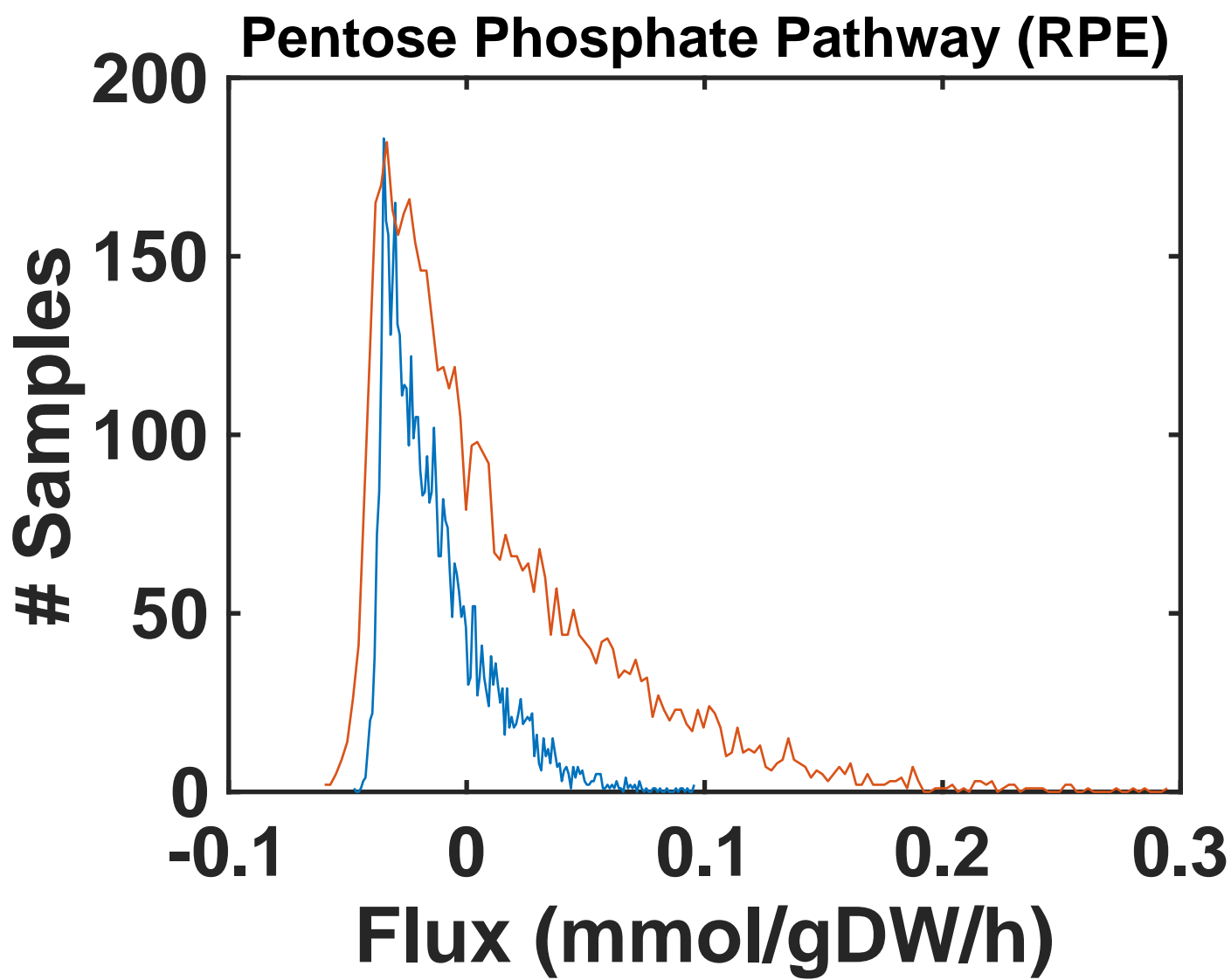

Supplement: Supplementary file 1 [file bioengineering-08-00103-s001.zip › FileS2/figure_sampling_noFVA-RPE.pdf]

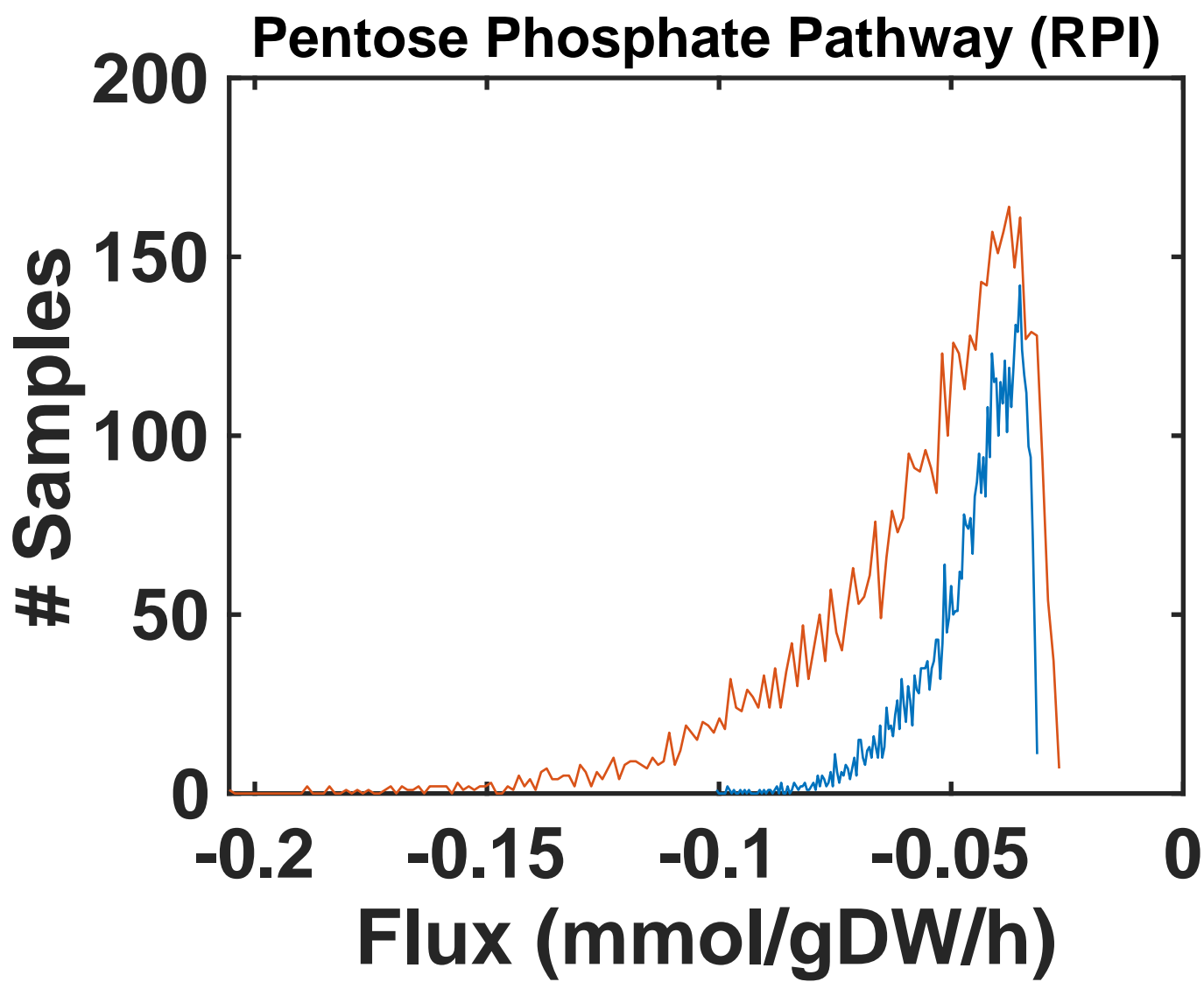

Supplement: Supplementary file 1 [file bioengineering-08-00103-s001.zip › FileS2/figure_sampling_noFVA-RPI.pdf]

# Citric Acid Cycle (SUCD1)

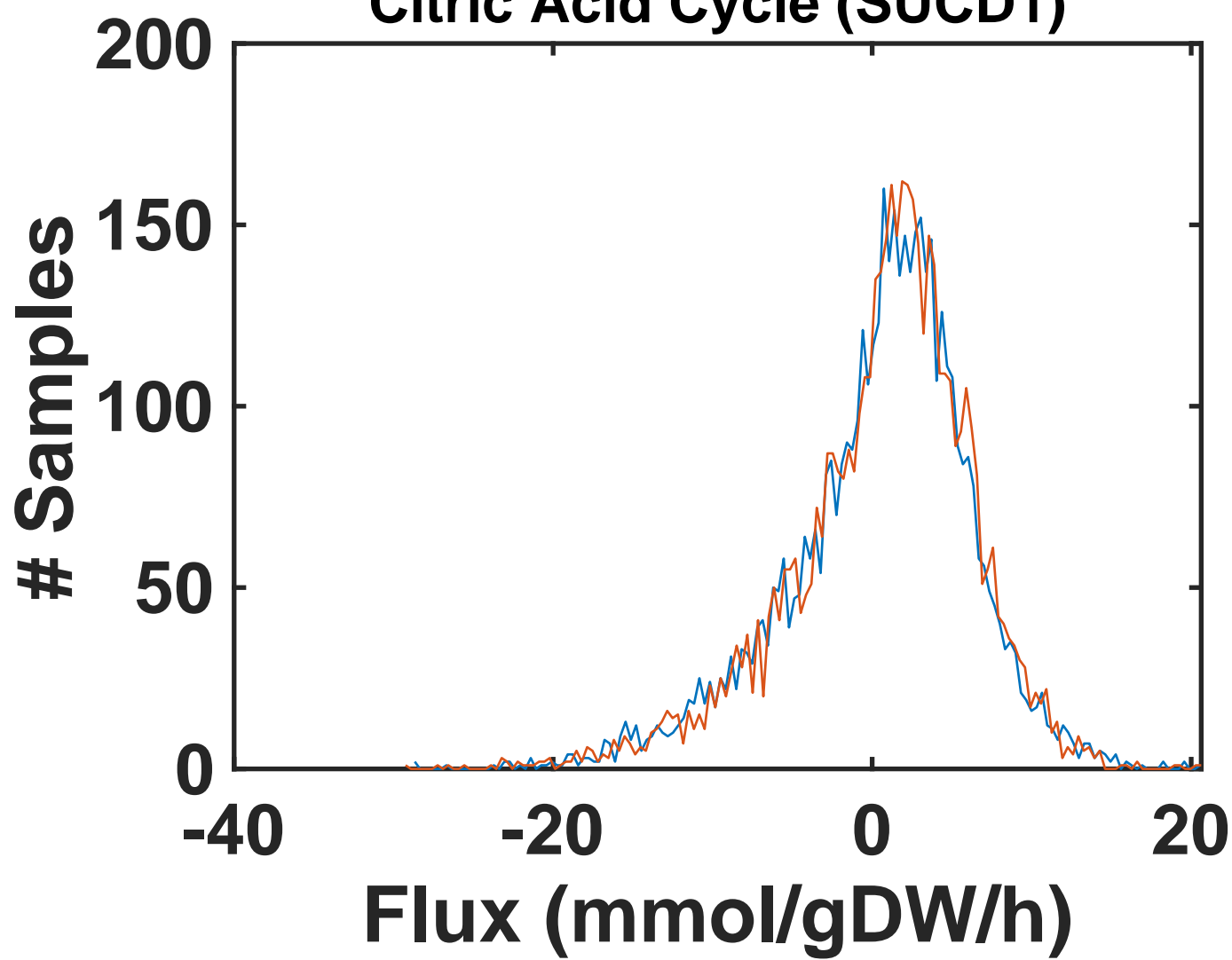

Supplement: Supplementary file 1 [file bioengineering-08-00103-s001.zip › FileS2/figure_sampling_noFVA-SUCD1.pdf]

# Citric Acid Cycle (SUCOAS)

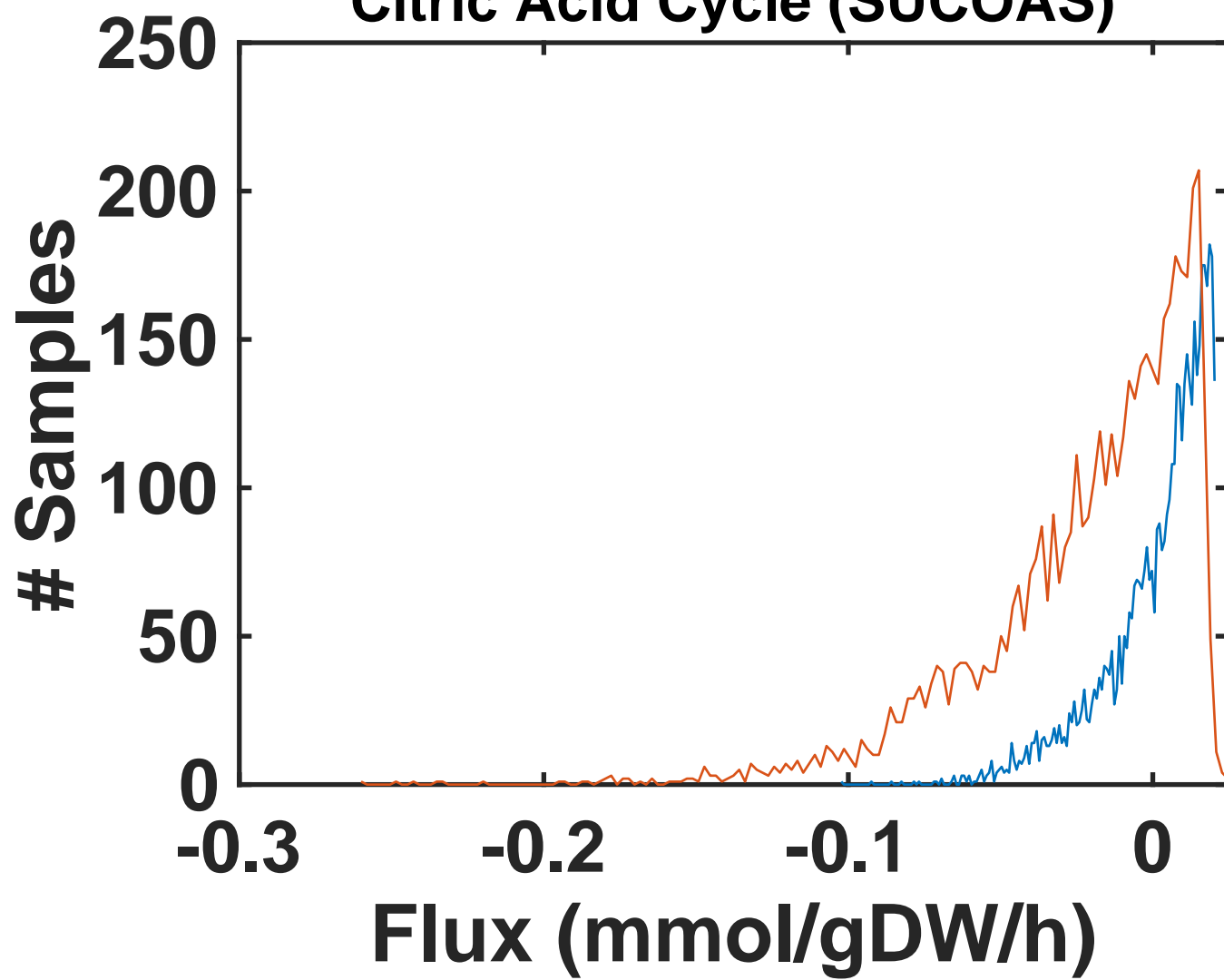

Supplement: Supplementary file 1 [file bioengineering-08-00103-s001.zip › FileS2/figure_sampling_noFVA-SUCOAS.pdf]

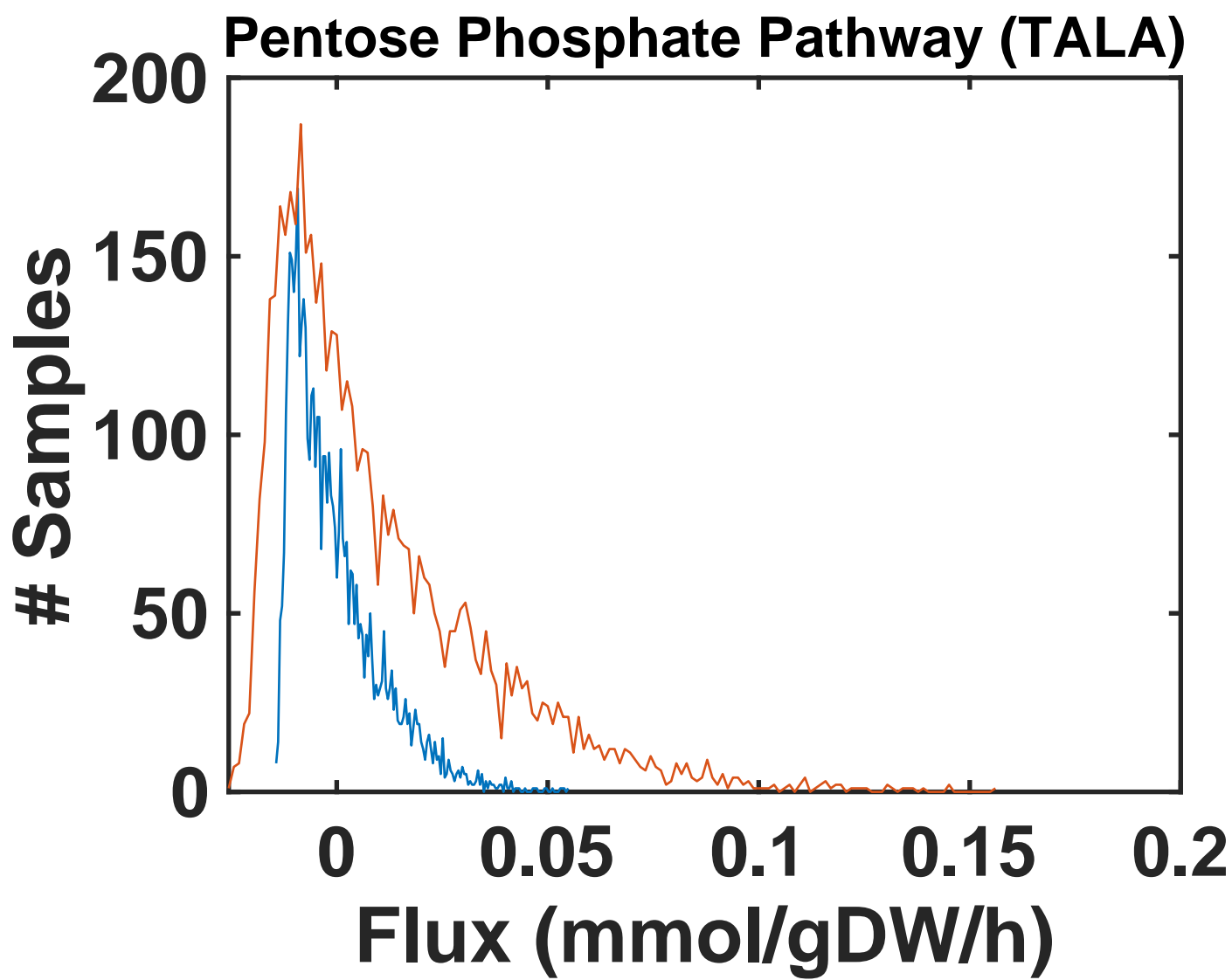

Supplement: Supplementary file 1 [file bioengineering-08-00103-s001.zip › FileS2/figure_sampling_noFVA-TALA.pdf]

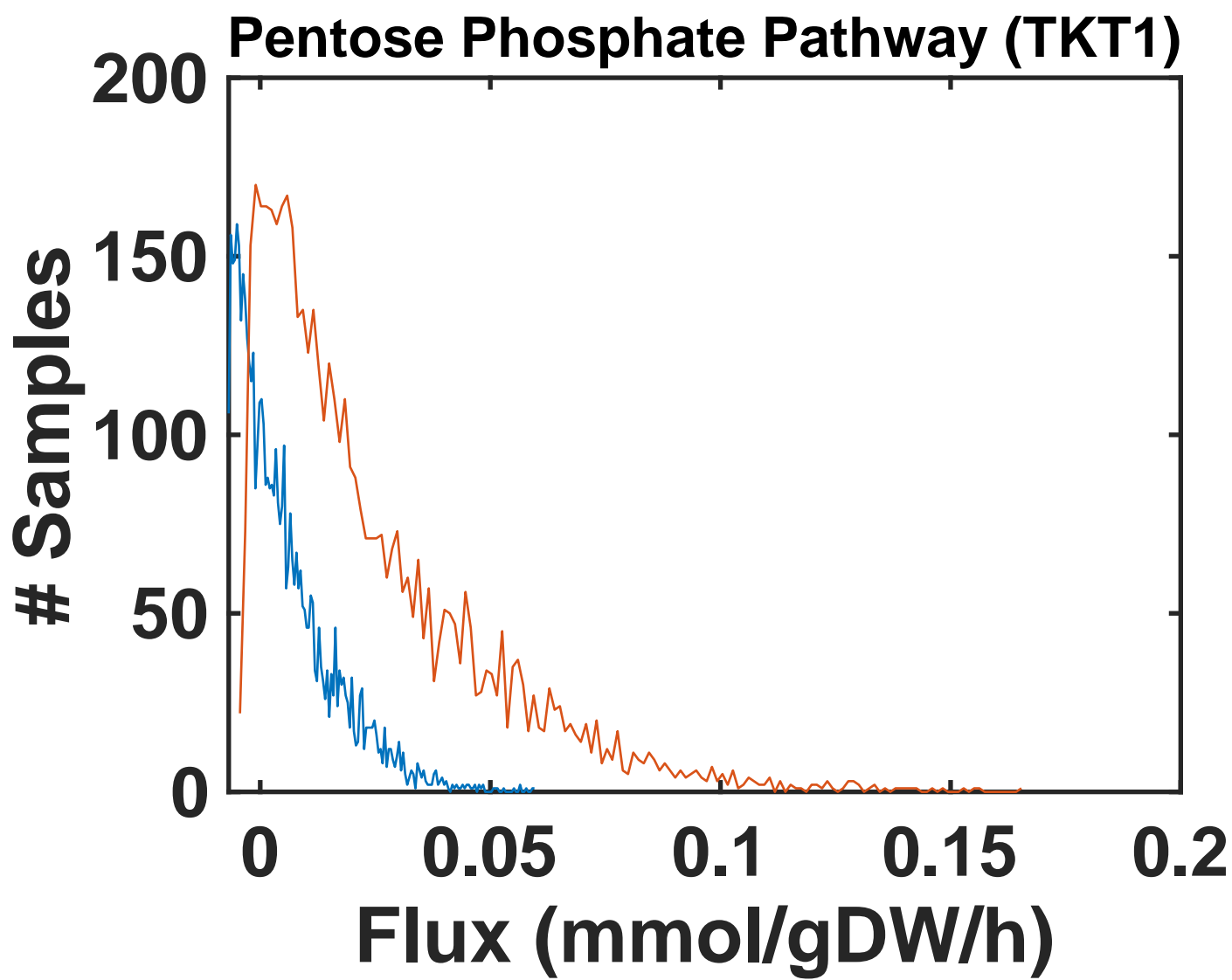

Supplement: Supplementary file 1 [file bioengineering-08-00103-s001.zip › FileS2/figure_sampling_noFVA-TKT1.pdf]

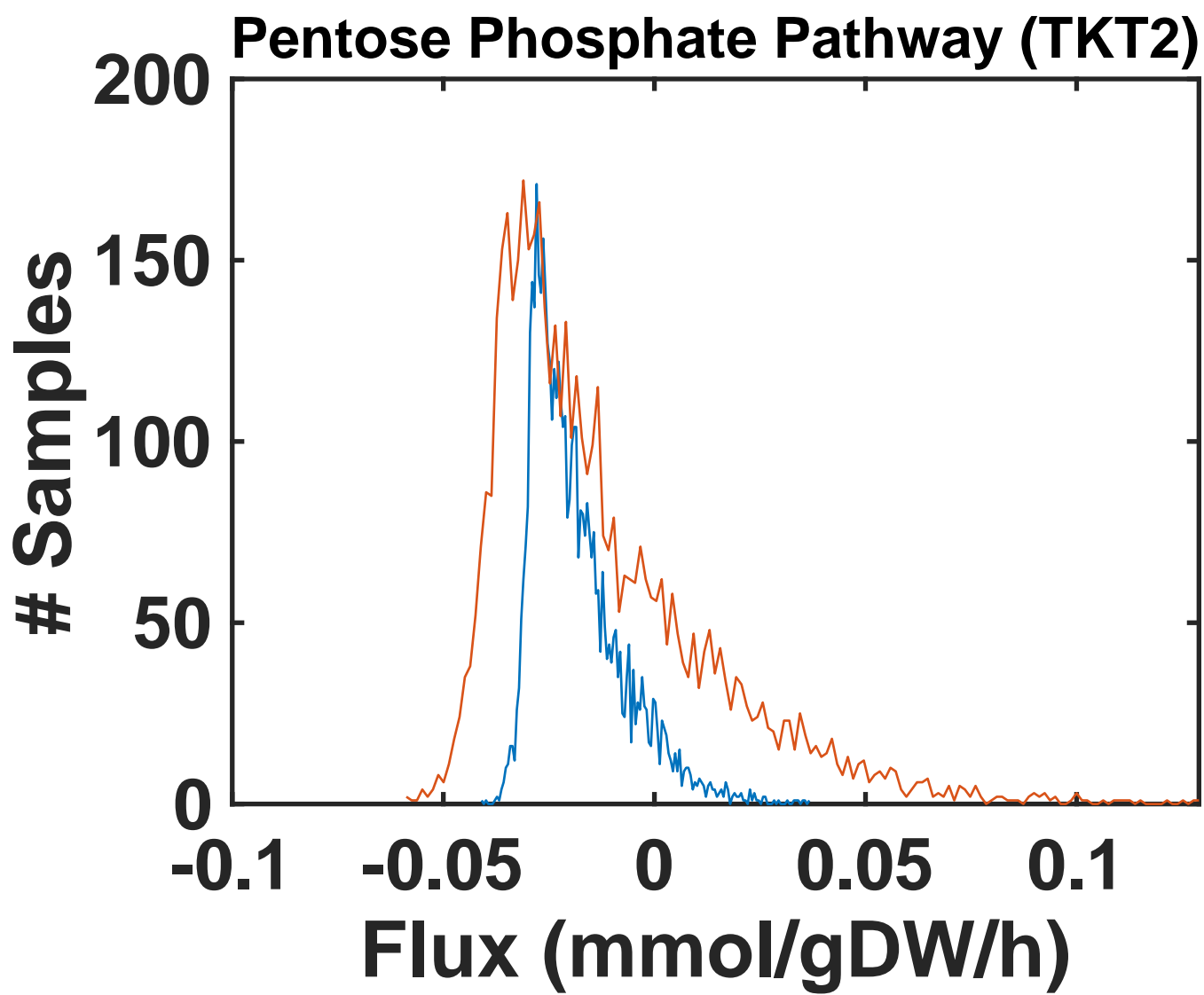

Supplement: Supplementary file 1 [file bioengineering-08-00103-s001.zip › FileS2/figure_sampling_noFVA-TKT2.pdf]

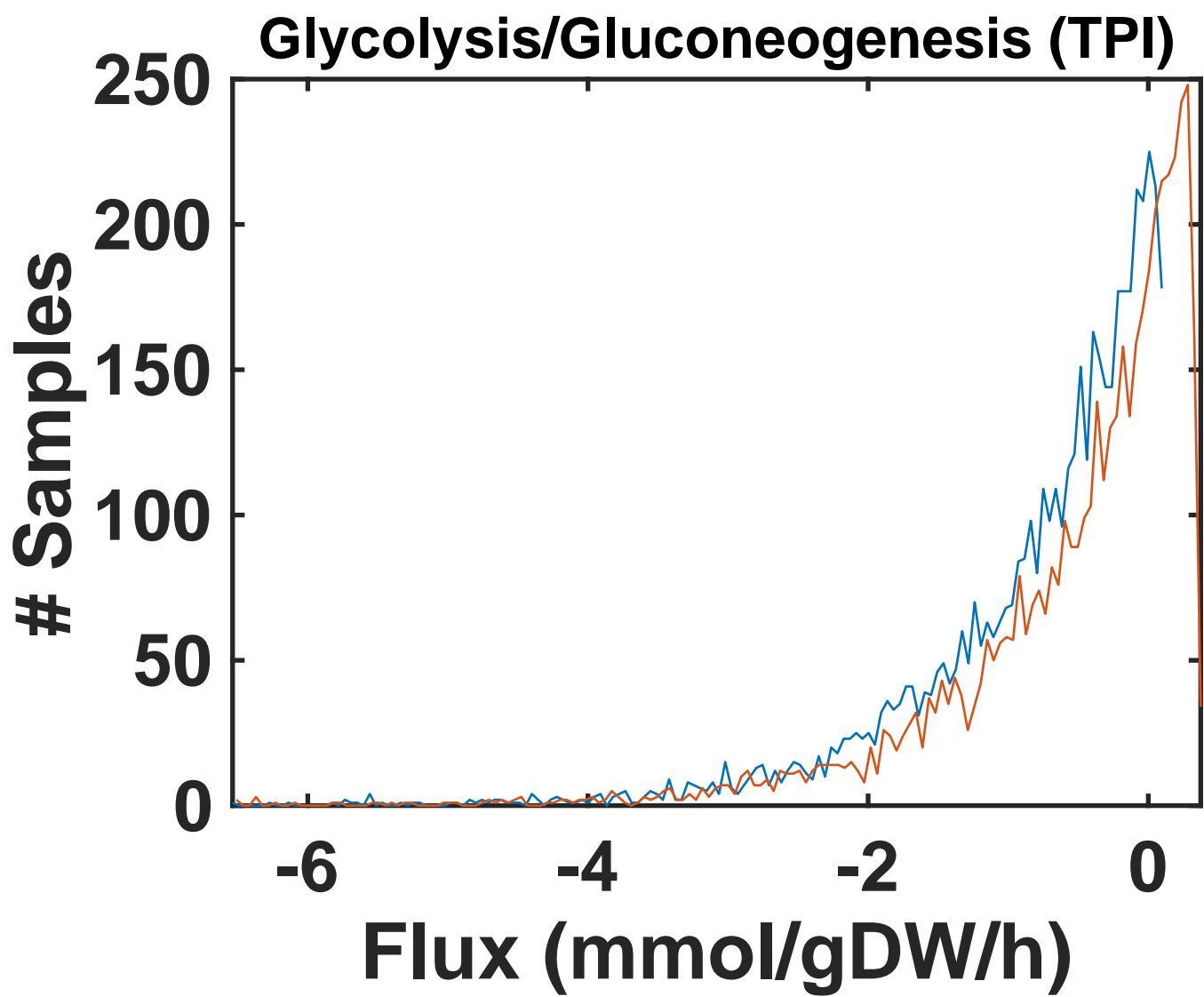

Supplement: Supplementary file 1 [file bioengineering-08-00103-s001.zip › FileS2/figure_sampling_noFVA-TPI.pdf]
