# Supplementary figures and images for: TCA Cycle and Its Relationship with Clavulanic Acid Production: A Further Interpretation by Using a Reduced Genome-Scale Metabolic Model of Streptomyces clavuligerus (part 1 of 2)
Source: Bioengineering (Basel). 2021 Jul 22;8(8):103. doi: 10.3390/bioengineering8080103 (PMC8389198; doi:10.3390/bioengineering8080103)

■ Batch, 36 h  
■ Fed-batch, 48 h

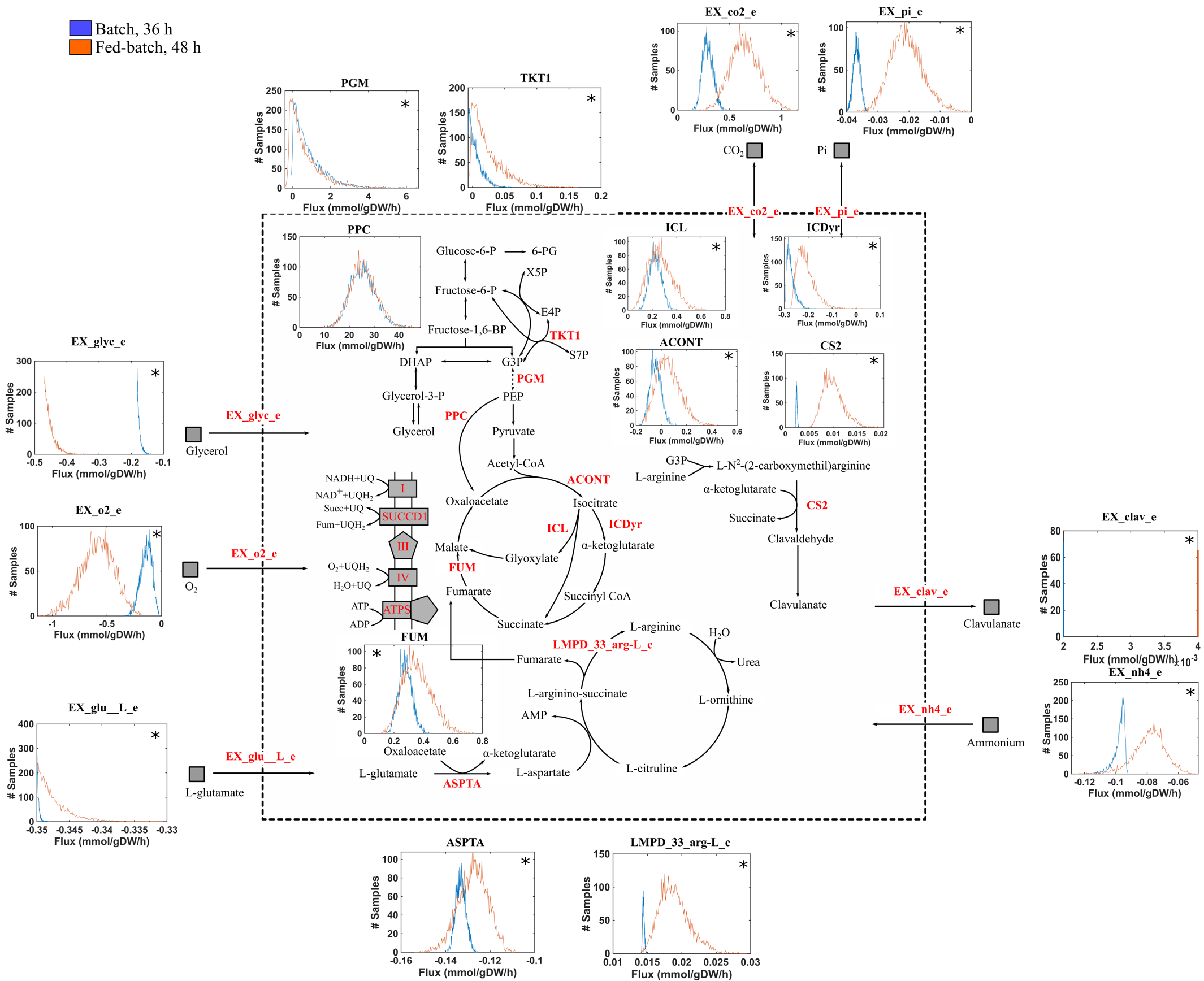

Supplement: Supplementary file 1 [file bioengineering-08-00103-s001.zip › FigureS1.pdf]

## Pyruvate Metabolism (ACALD)

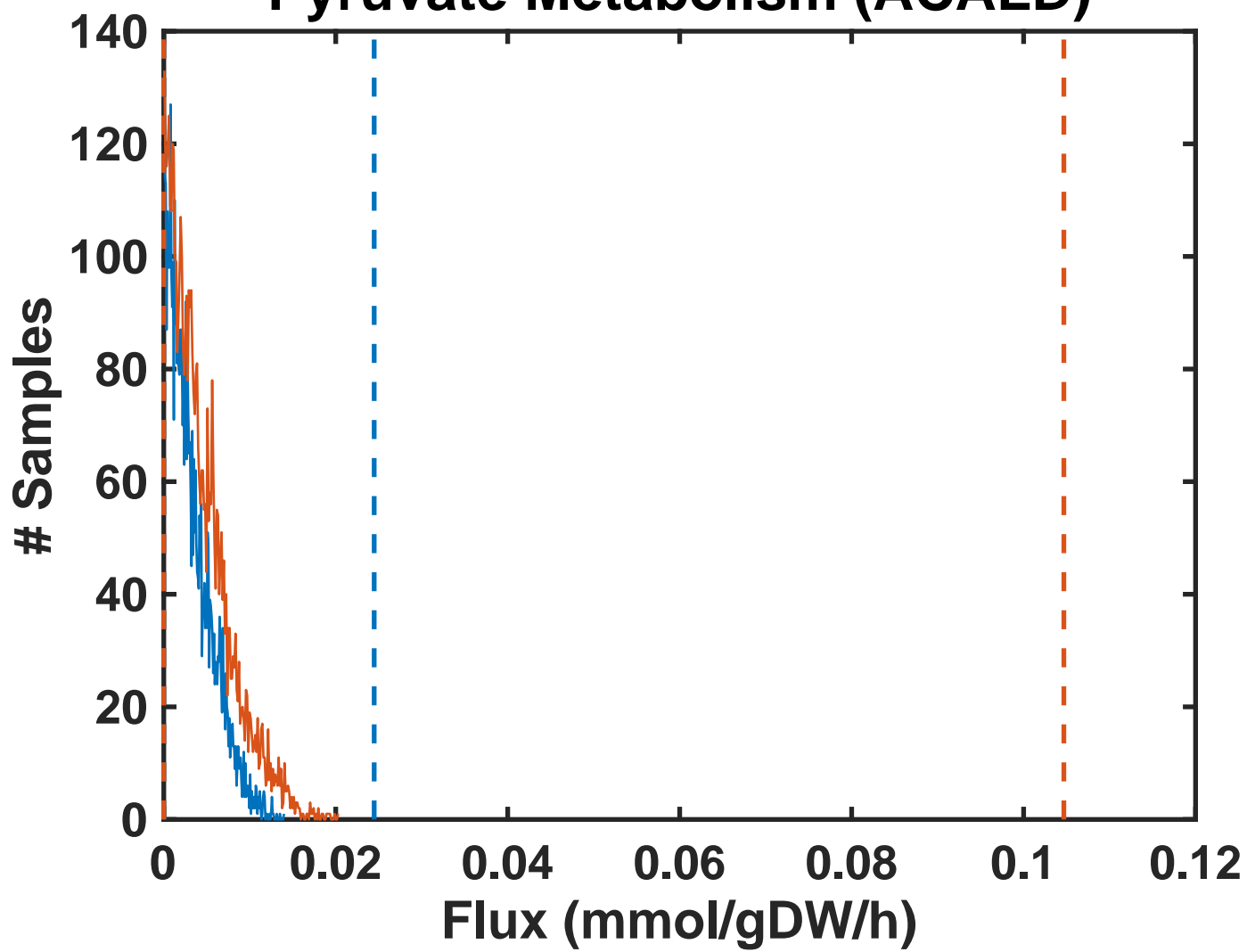

Supplement: Supplementary file 1 [file bioengineering-08-00103-s001.zip › FileS2/figure_sampling-ACALD.pdf]

## Pyruvate Metabolism (ACKr)

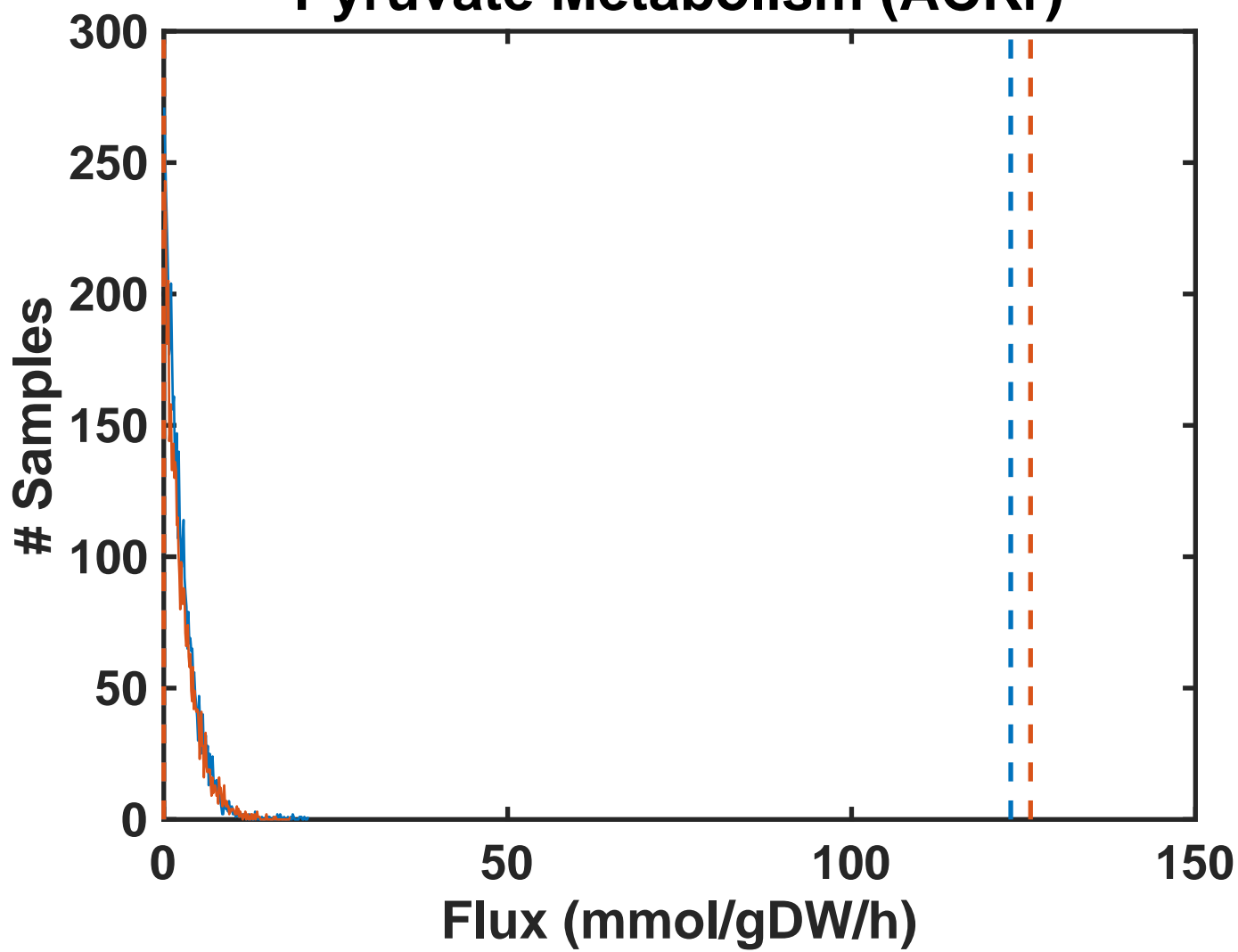

Supplement: Supplementary file 1 [file bioengineering-08-00103-s001.zip › FileS2/figure_sampling-ACKr.pdf]

## Citric Acid Cycle (ACONT)

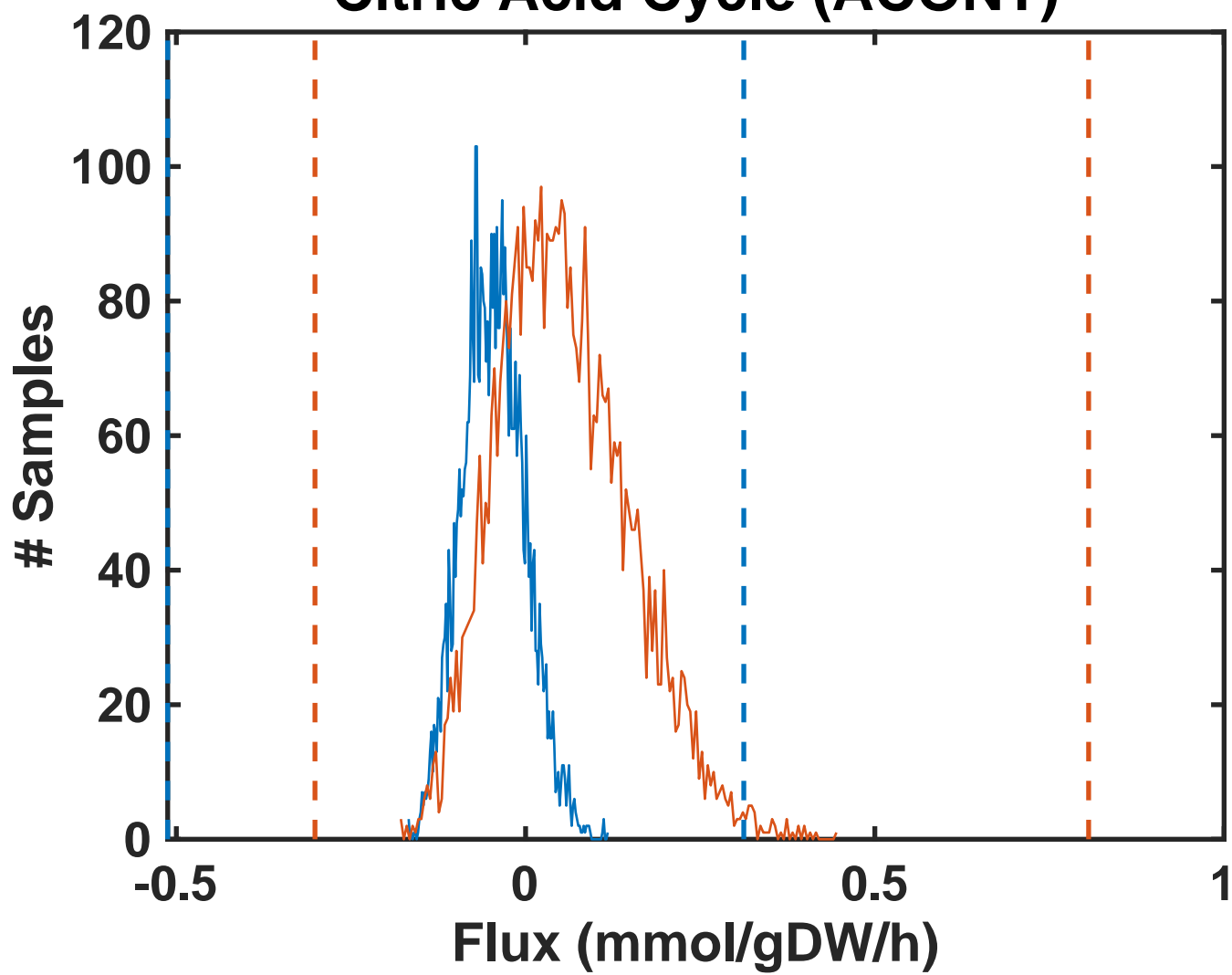

Supplement: Supplementary file 1 [file bioengineering-08-00103-s001.zip › FileS2/figure_sampling-ACONT.pdf]

(AKGDH)

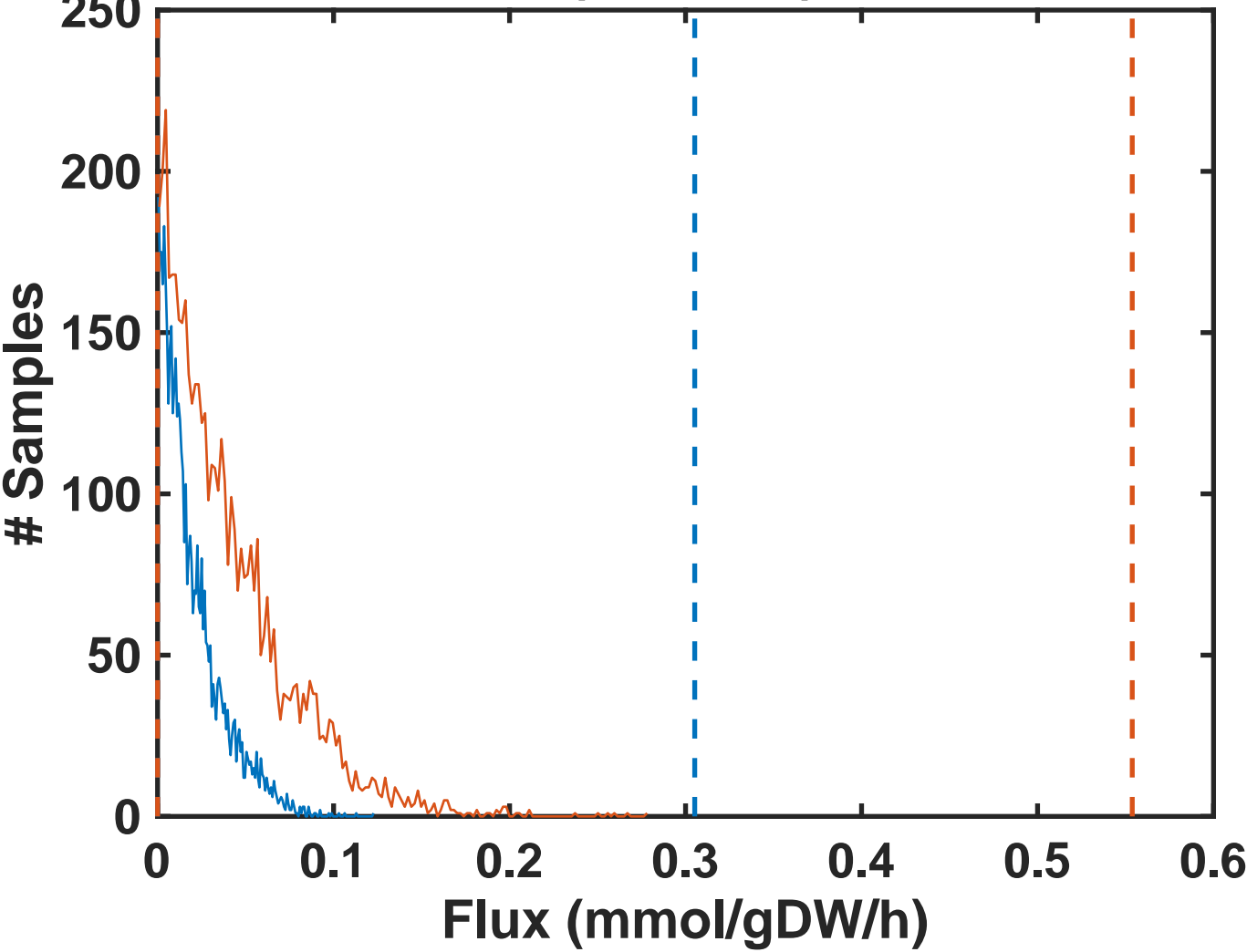

Supplement: Supplementary file 1 [file bioengineering-08-00103-s001.zip › FileS2/figure_sampling-AKGDH.pdf]

## Pyruvate Metabolism (ALCD2x)

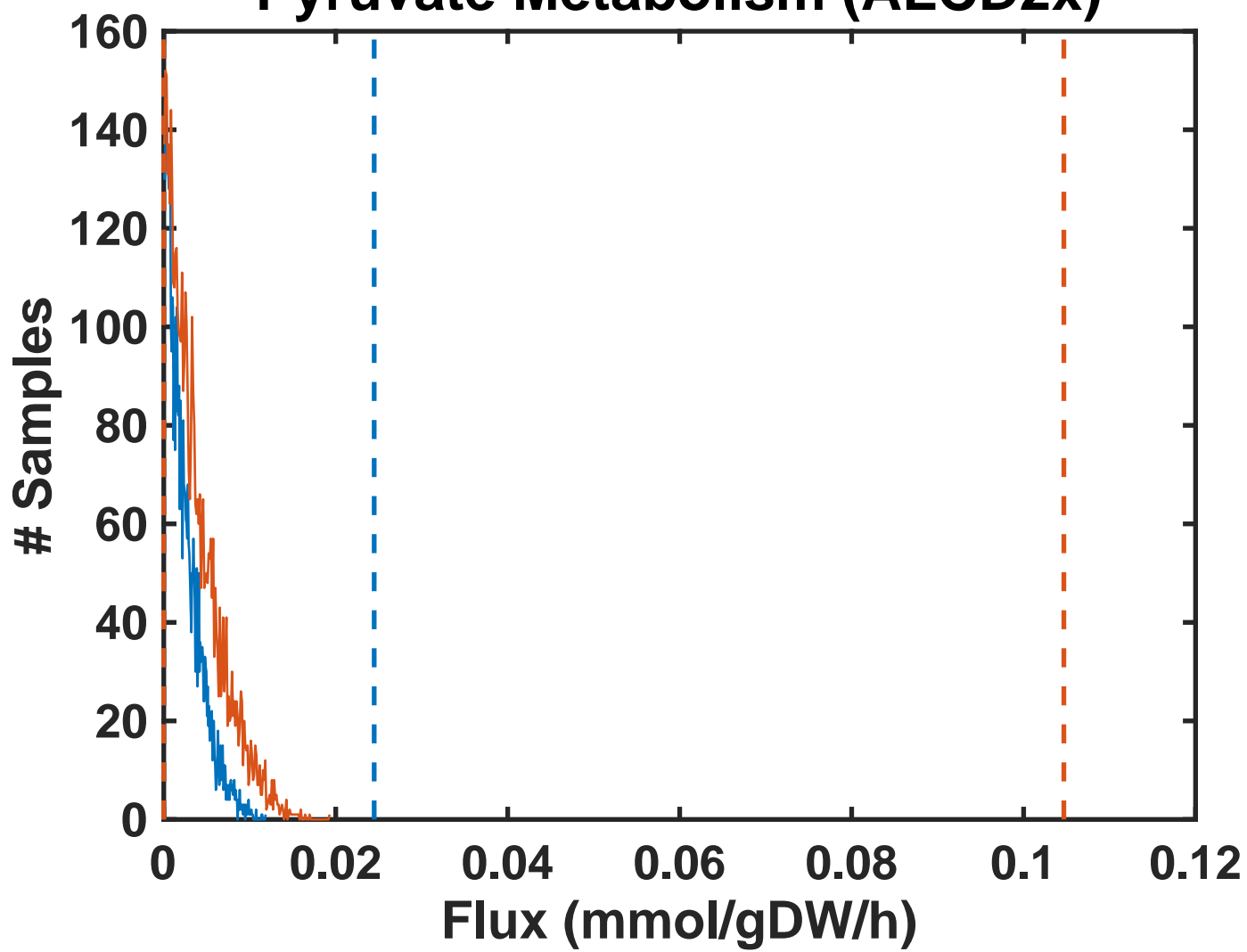

Supplement: Supplementary file 1 [file bioengineering-08-00103-s001.zip › FileS2/figure_sampling-ALCD2x.pdf]

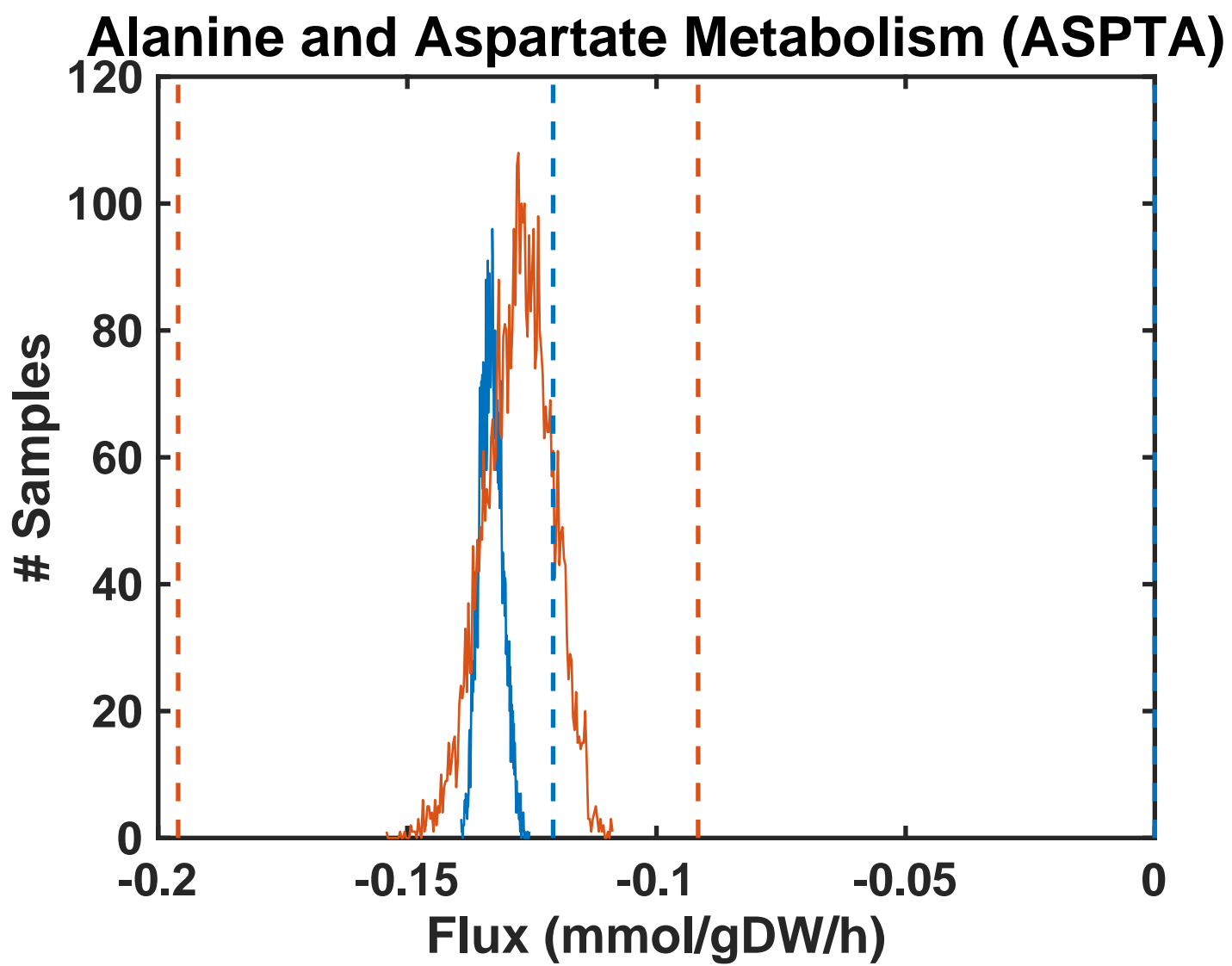

Supplement: Supplementary file 1 [file bioengineering-08-00103-s001.zip › FileS2/figure_sampling-ASPTA.pdf]

# Oxidative Phosphorylation (ATPS4rpp)

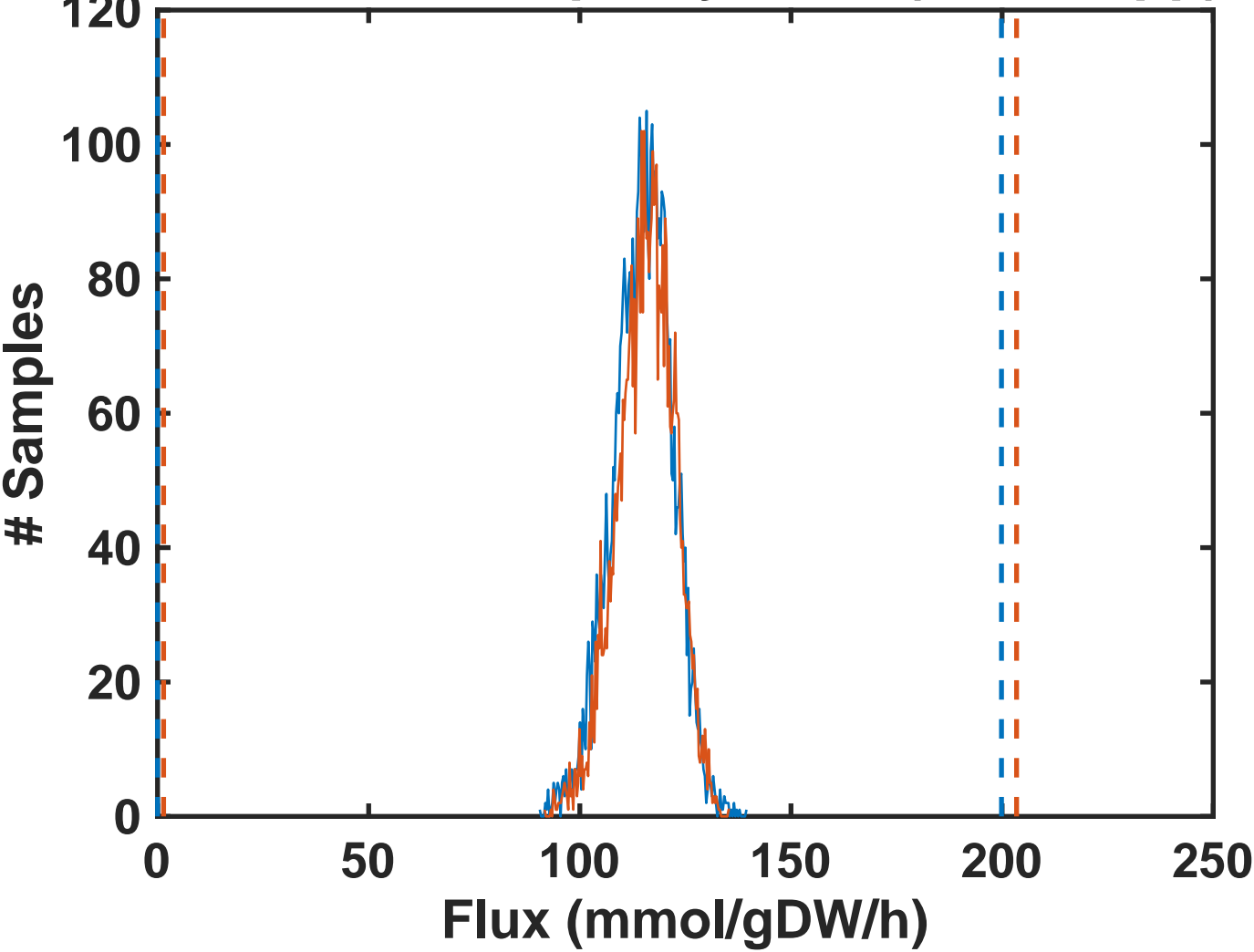

Supplement: Supplementary file 1 [file bioengineering-08-00103-s001.zip › FileS2/figure_sampling-ATPS4rpp.pdf]

## Arginine and Proline Metabolism (CBPS)

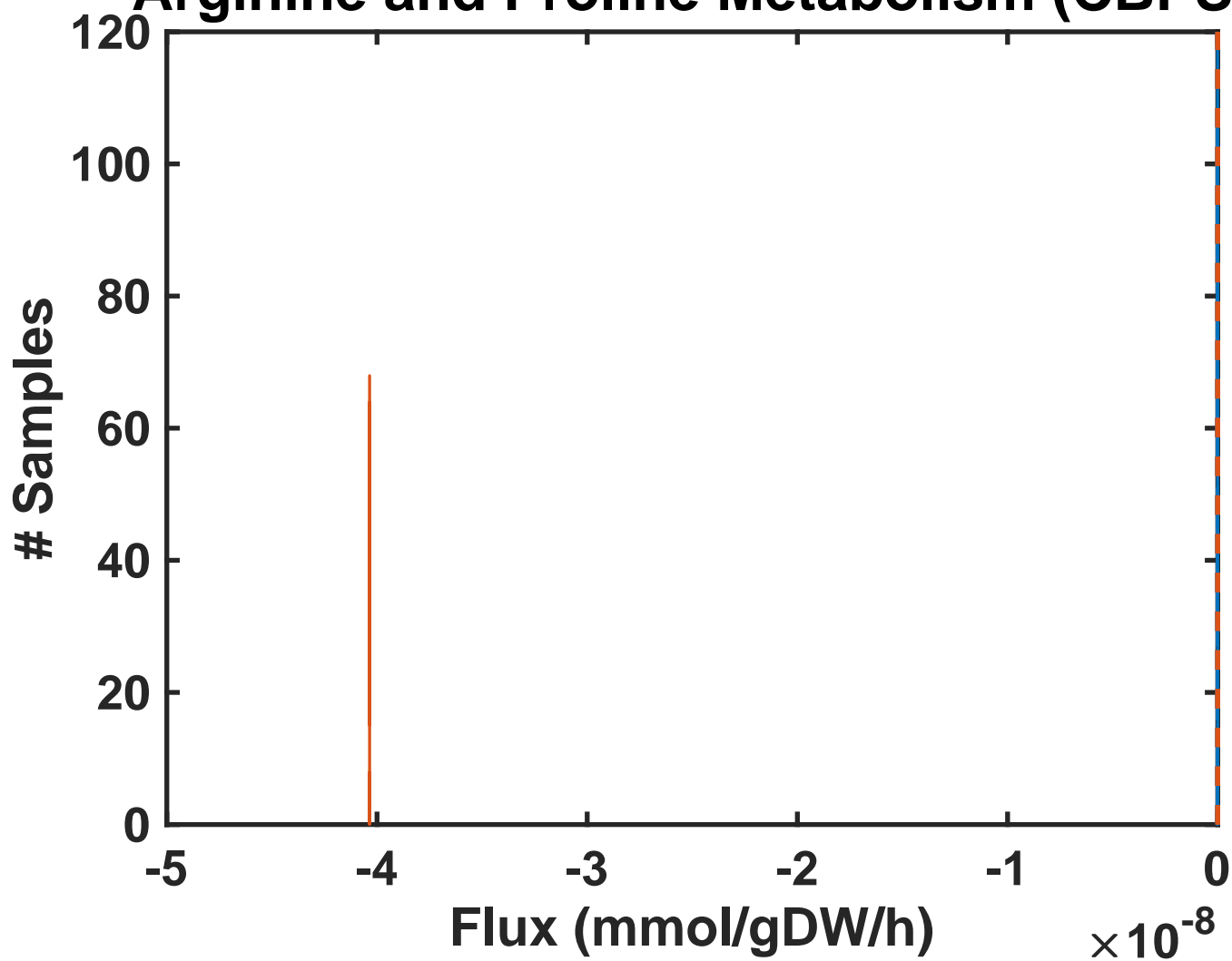

Supplement: Supplementary file 1 [file bioengineering-08-00103-s001.zip › FileS2/figure_sampling-CBPS.pdf]

## Clavulanic acid biosynthesis (CEAS)

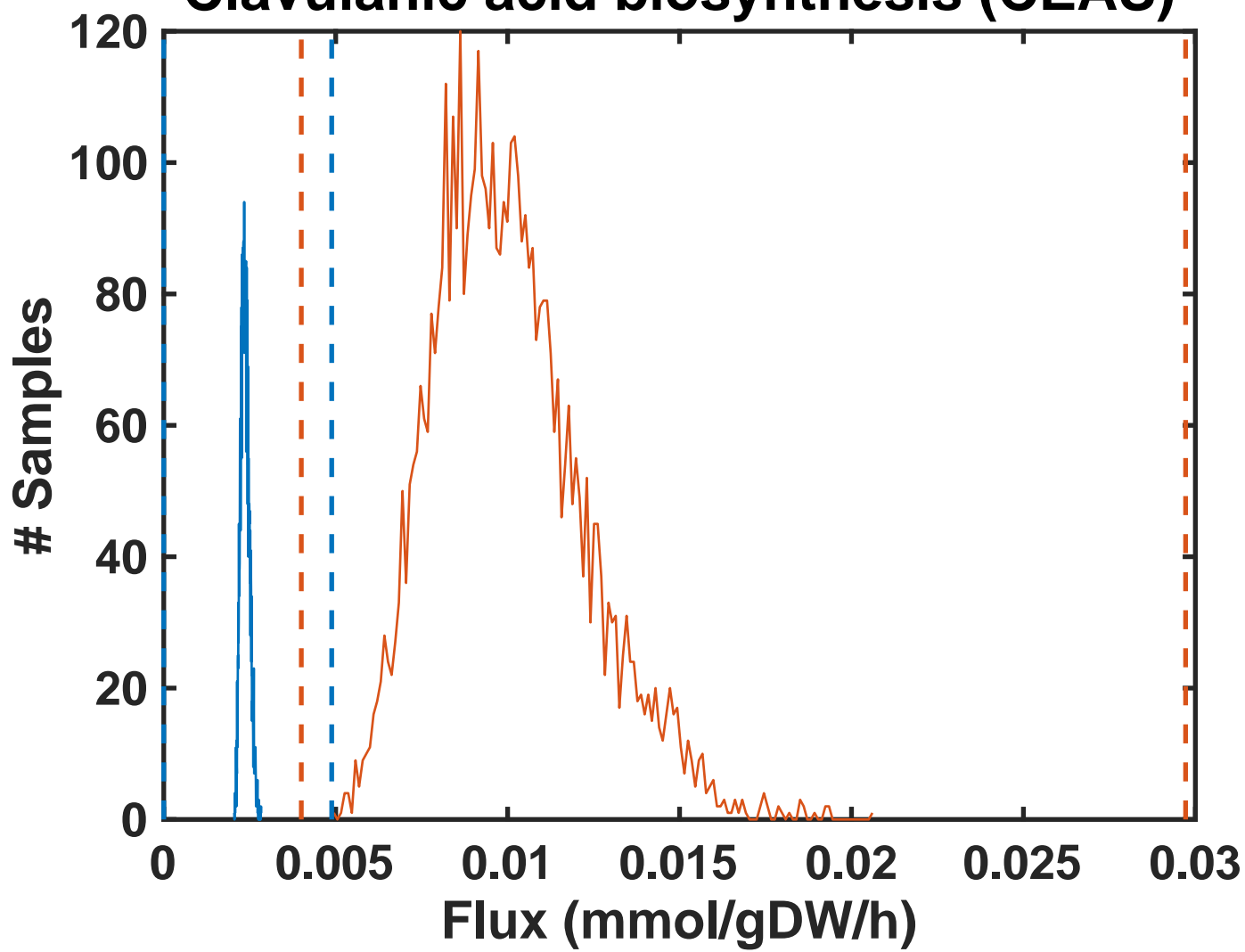

Supplement: Supplementary file 1 [file bioengineering-08-00103-s001.zip › FileS2/figure_sampling-CEAS.pdf]

## Citric Acid Cycle (CITL)

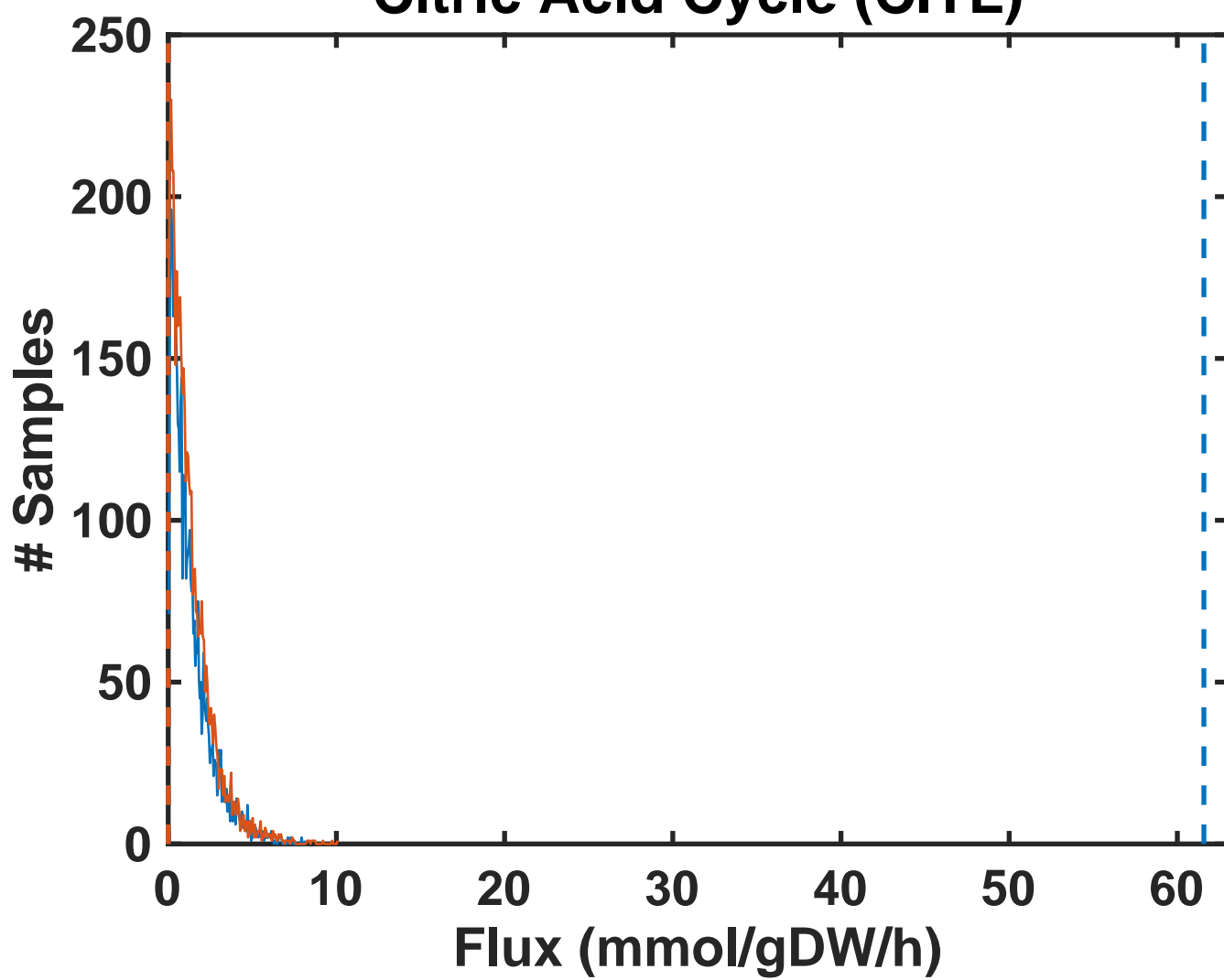

Supplement: Supplementary file 1 [file bioengineering-08-00103-s001.zip › FileS2/figure_sampling-CITL.pdf]

## Penicillin-Cephalosporin biosynthesis (CMC1)

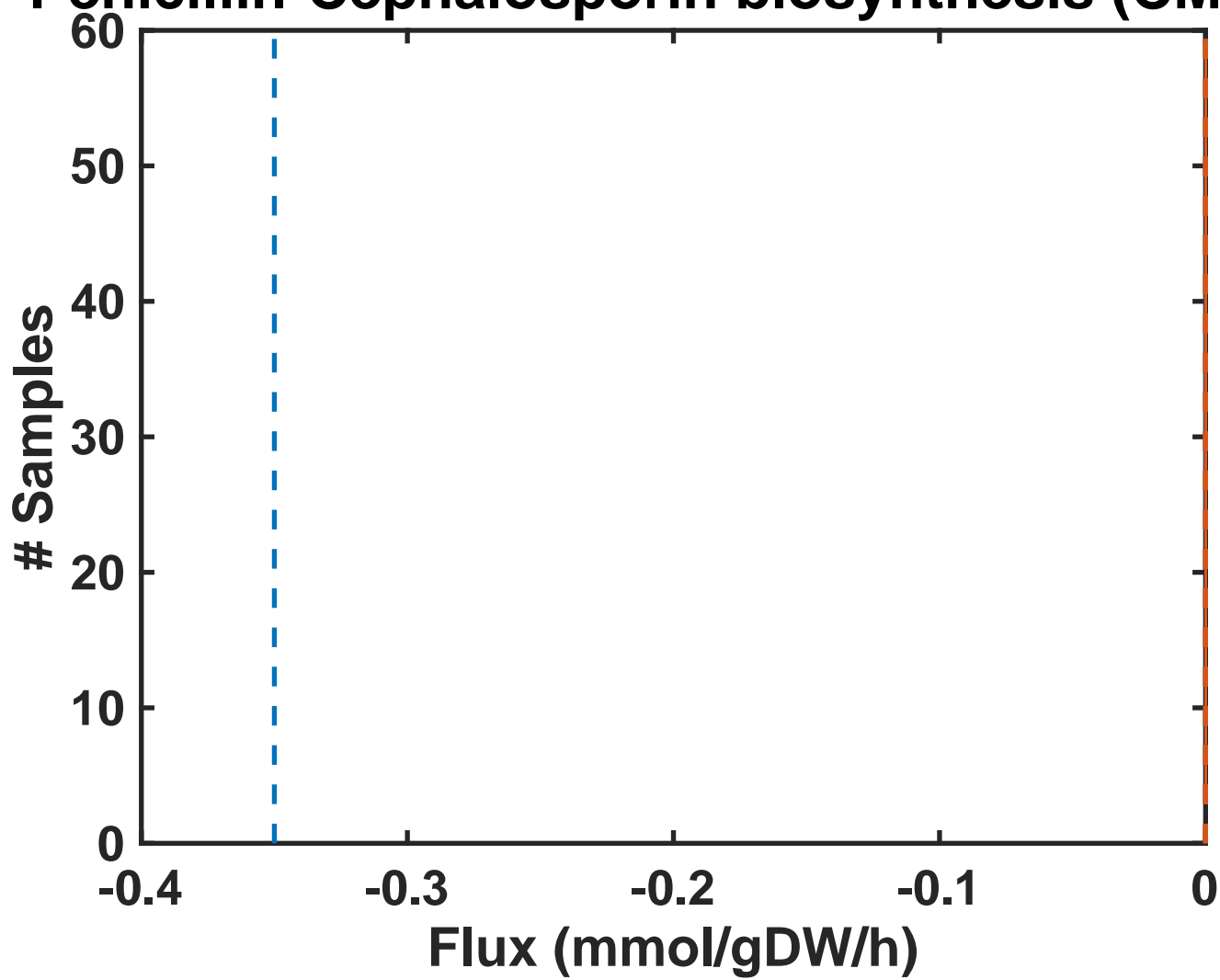

Supplement: Supplementary file 1 [file bioengineering-08-00103-s001.zip › FileS2/figure_sampling-CMC1.pdf]

## Citric Acid Cycle (CS)

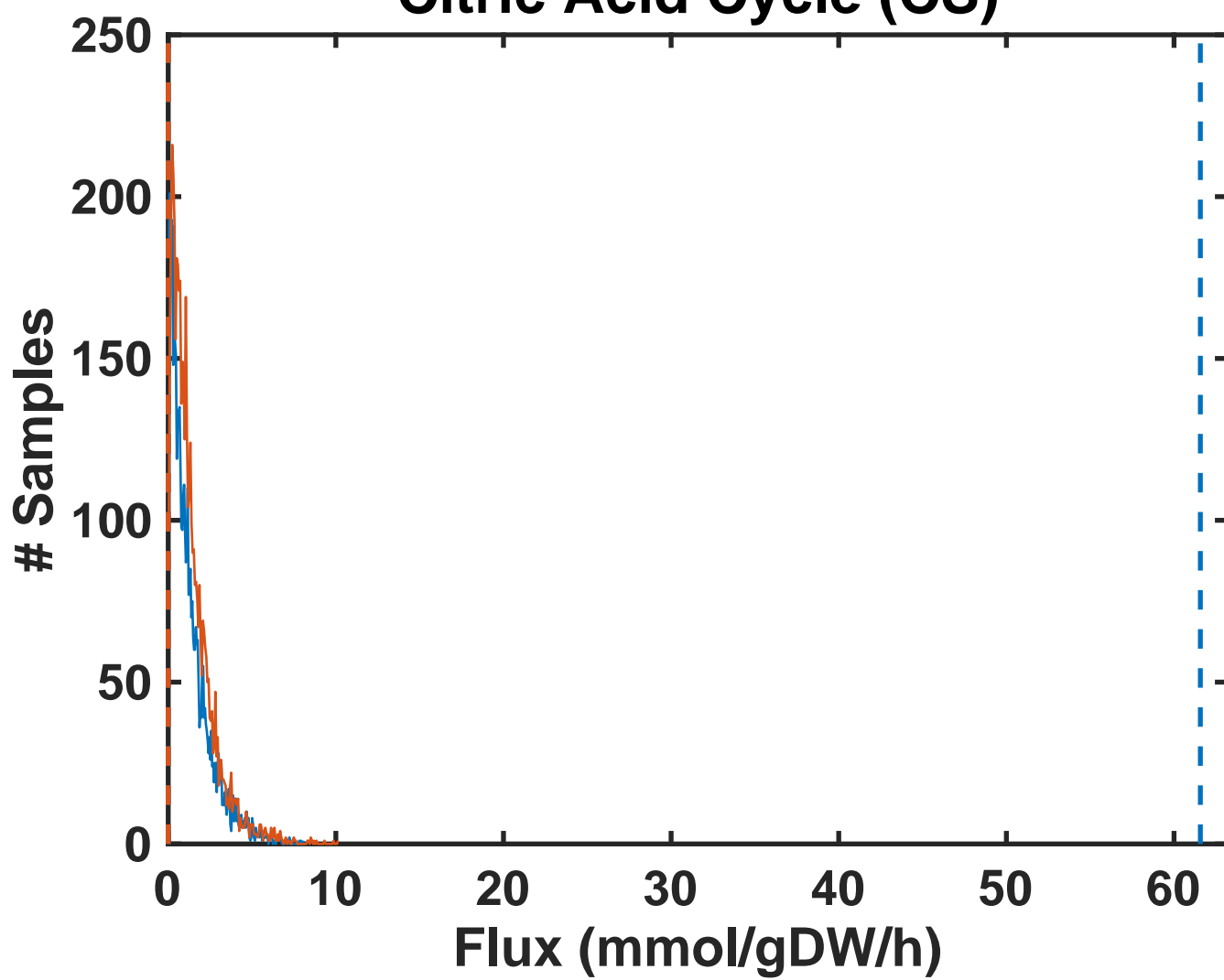

Supplement: Supplementary file 1 [file bioengineering-08-00103-s001.zip › FileS2/figure_sampling-CS.pdf]

## Clavulanic acid biosynthesis (CS1)

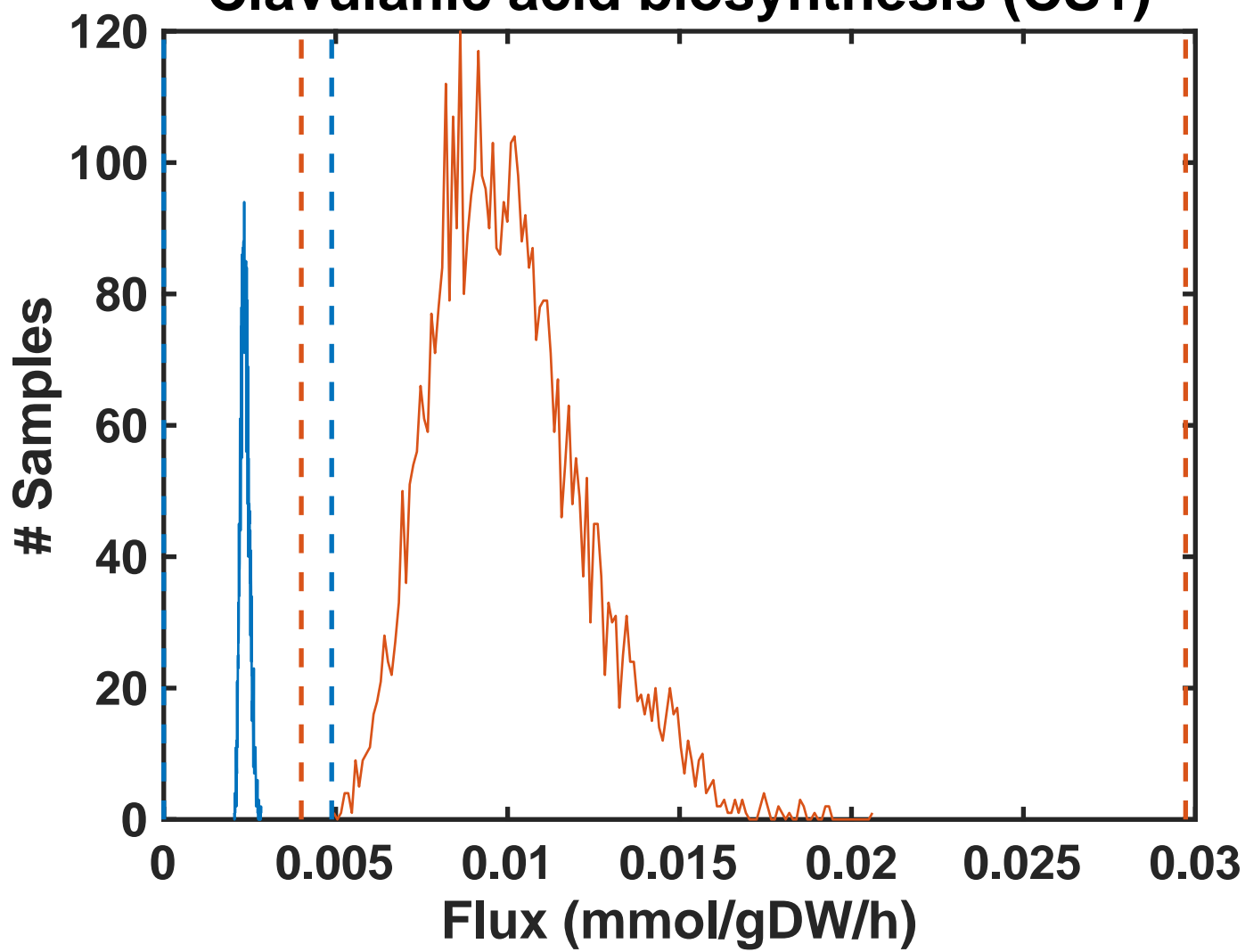

Supplement: Supplementary file 1 [file bioengineering-08-00103-s001.zip › FileS2/figure_sampling-CS1.pdf]

# Clavulanic acid biosynthesis (CS2)

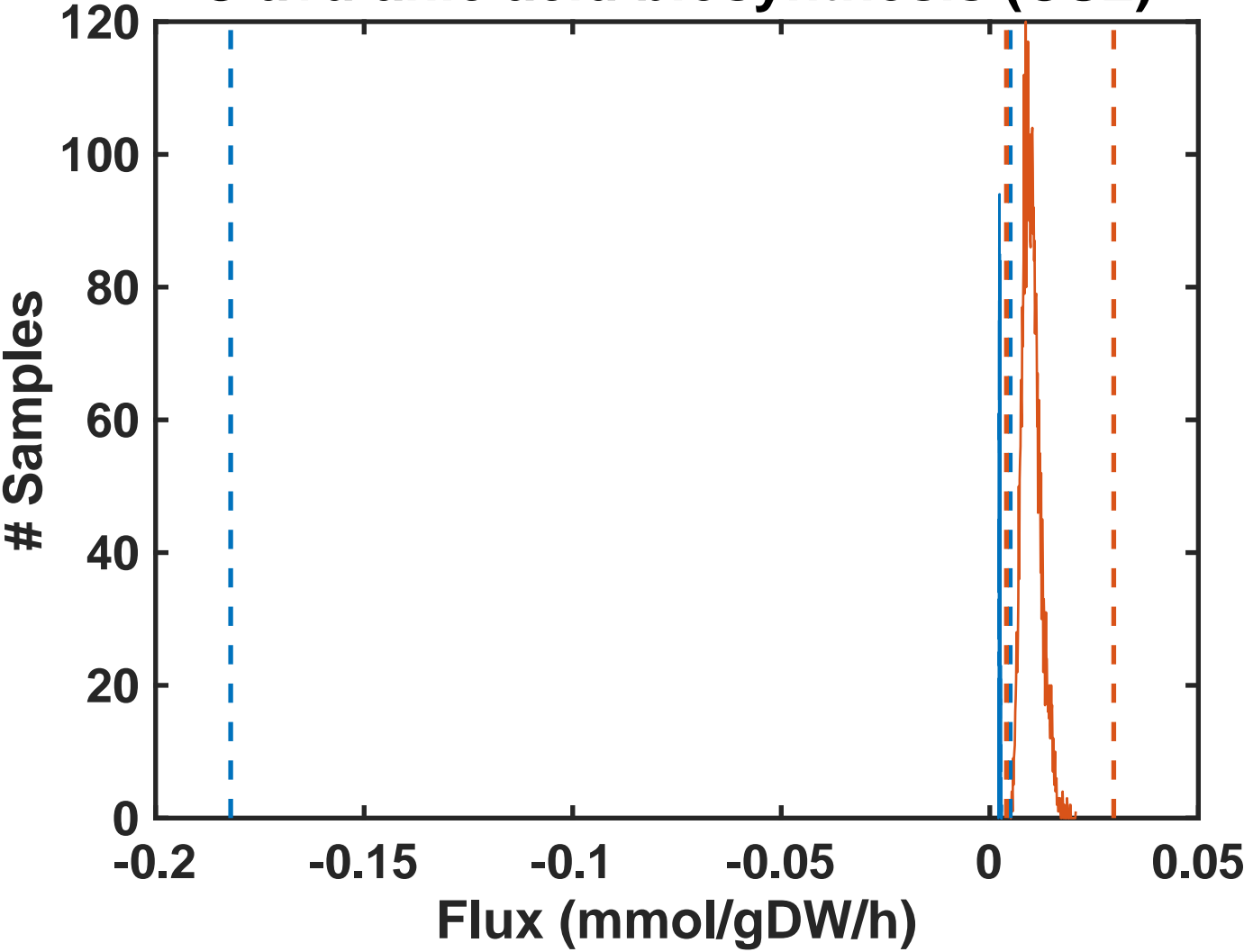

Supplement: Supplementary file 1 [file bioengineering-08-00103-s001.zip › FileS2/figure_sampling-CS2.pdf]

## Clavulanic acid biosynthesis (CS3)

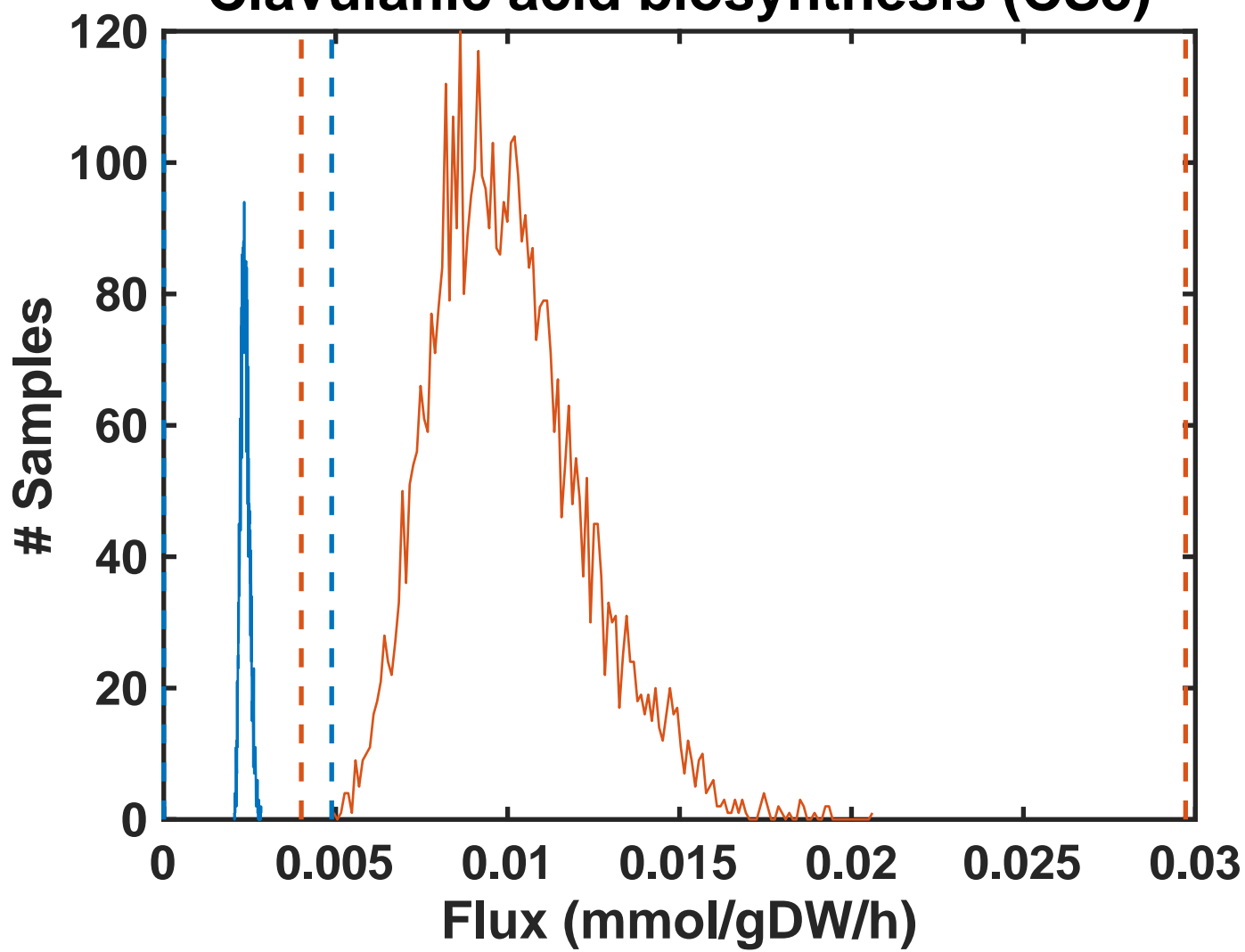

Supplement: Supplementary file 1 [file bioengineering-08-00103-s001.zip › FileS2/figure_sampling-CS3.pdf]

## Glycolysis/Gluconeogenesis (ENO)

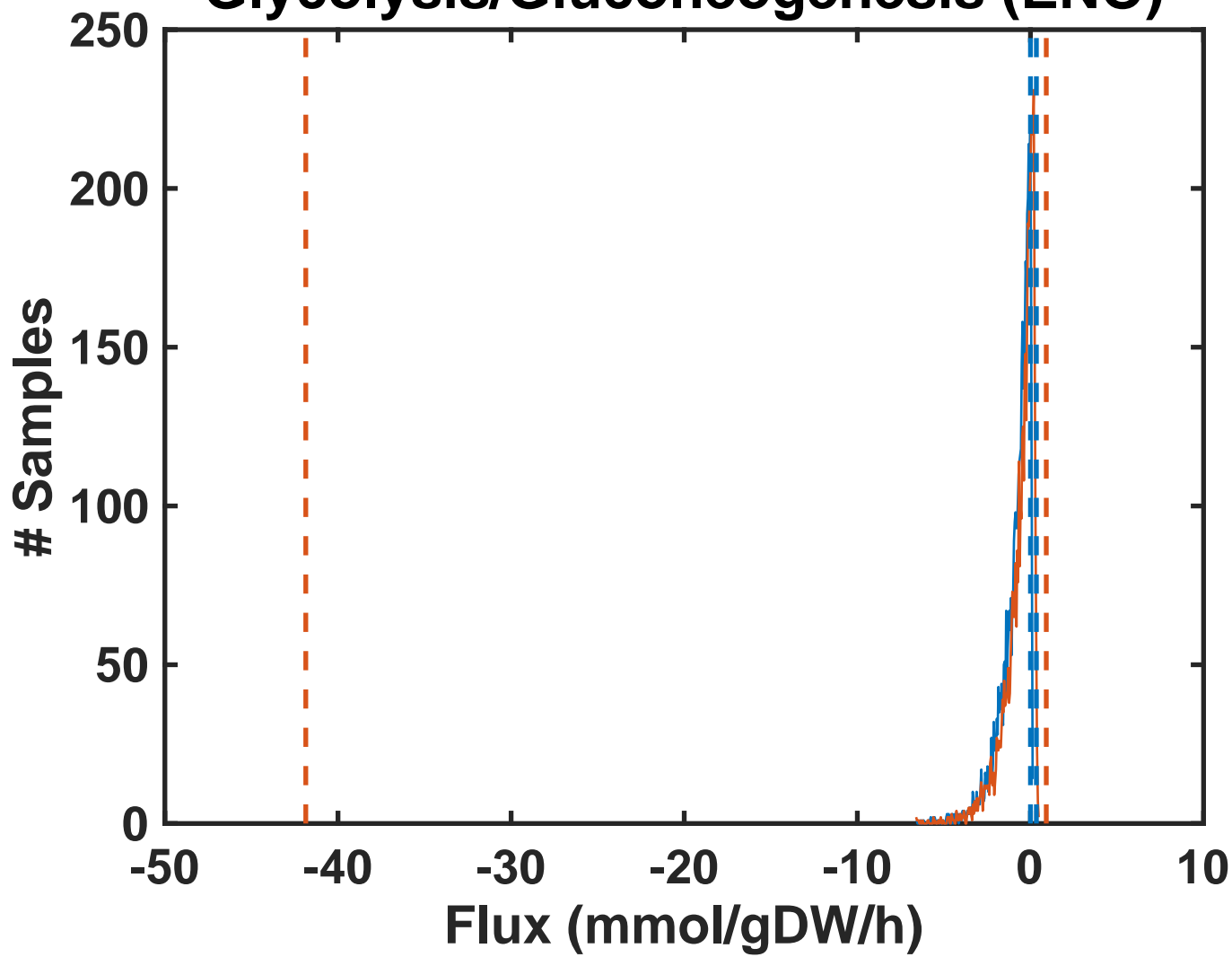

Supplement: Supplementary file 1 [file bioengineering-08-00103-s001.zip › FileS2/figure_sampling-ENO.pdf]

# Exchange ( $EX_c lav_e$ )

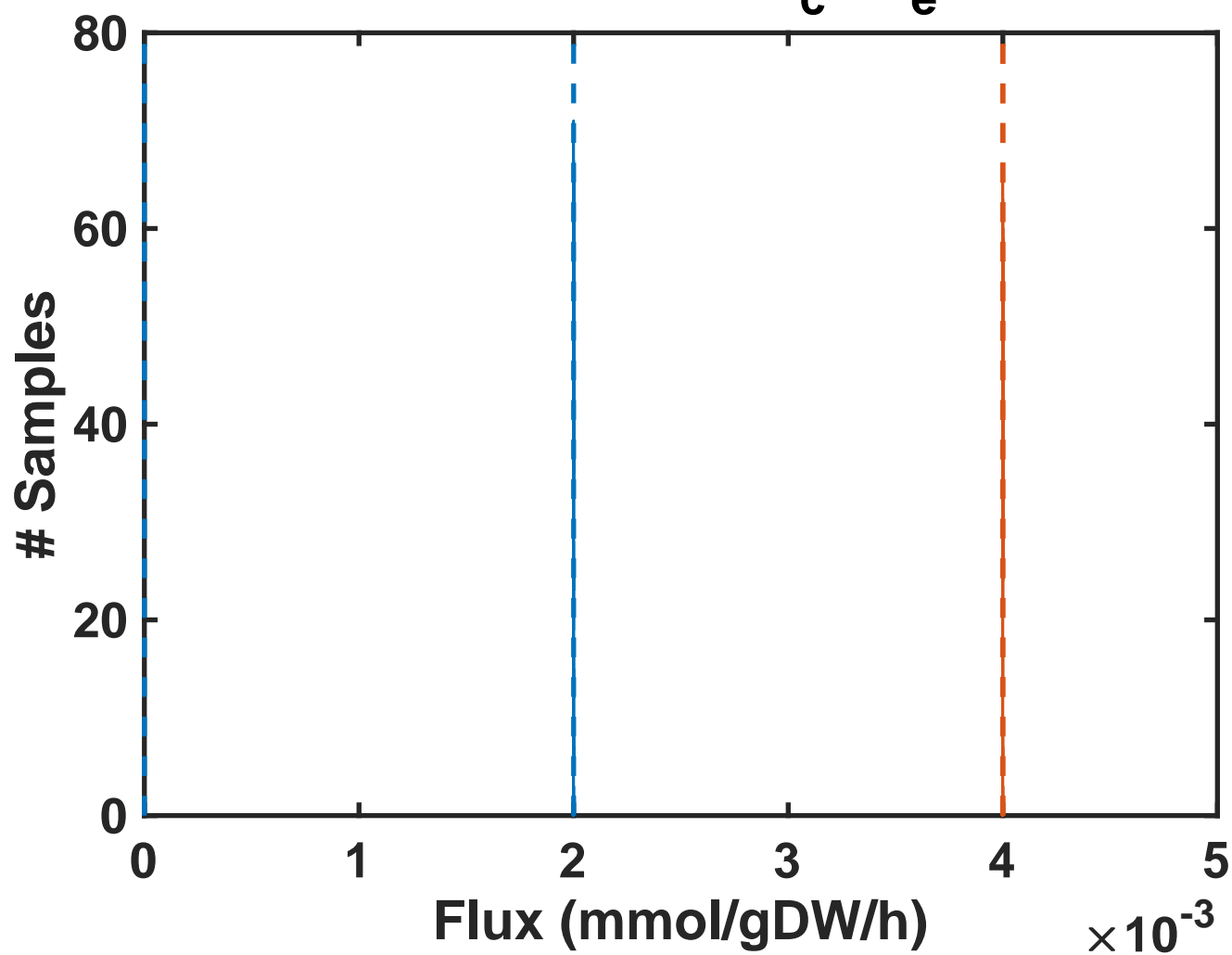

Supplement: Supplementary file 1 [file bioengineering-08-00103-s001.zip › FileS2/figure_sampling-EX_clav_e.pdf]

# Exchange ( $EX_c o2_e$ )

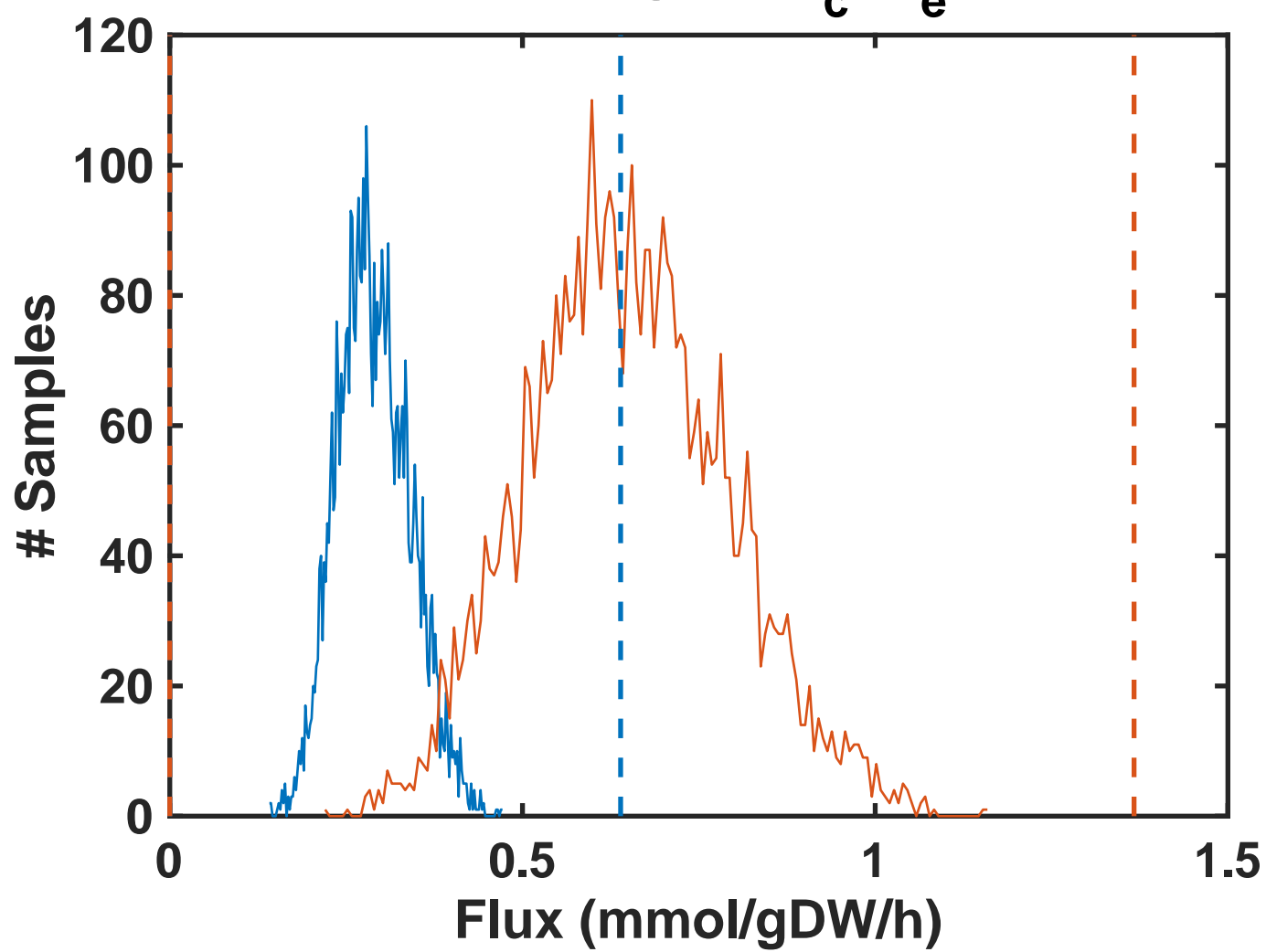

Supplement: Supplementary file 1 [file bioengineering-08-00103-s001.zip › FileS2/figure_sampling-EX_co2_e.pdf]

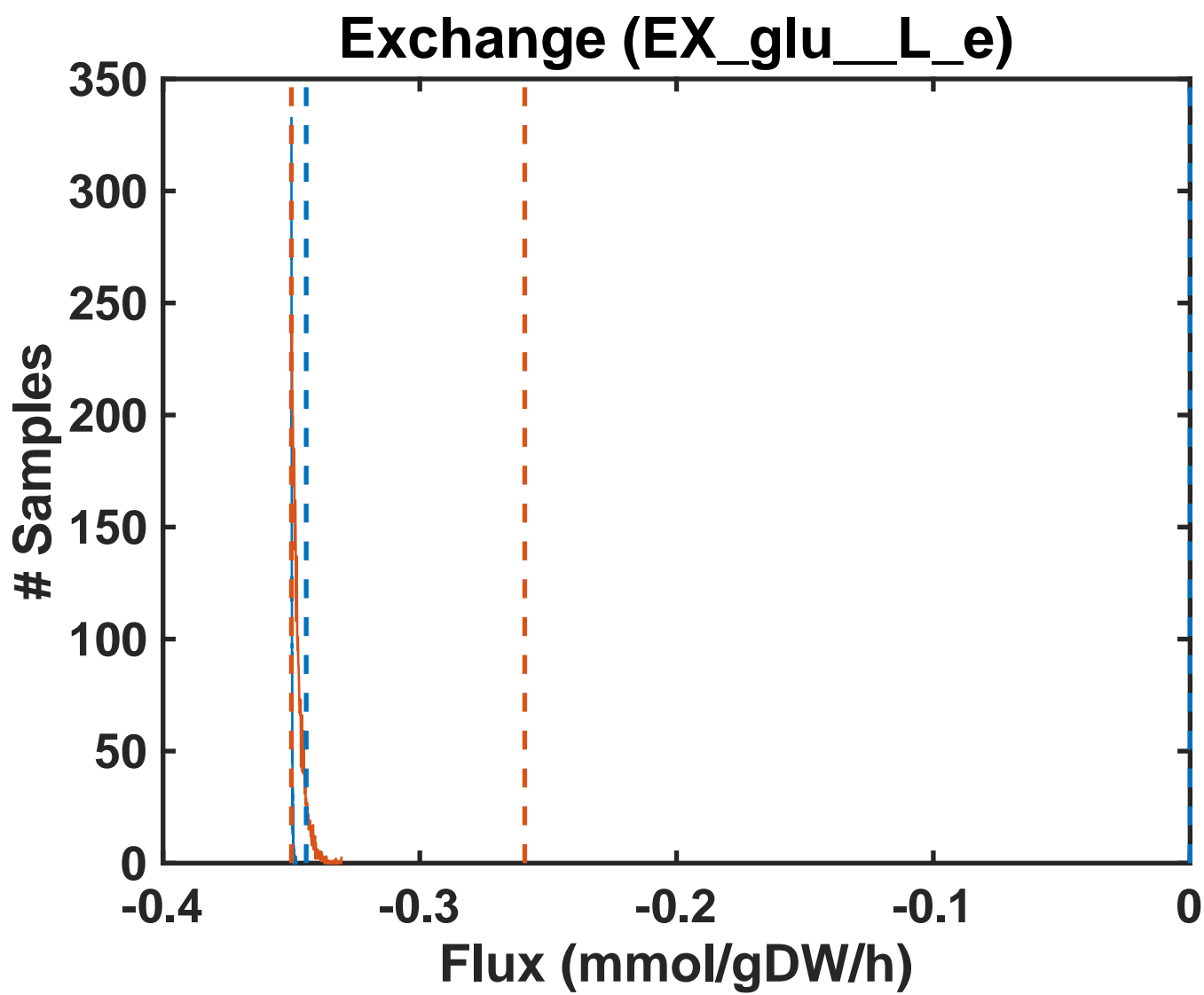

Supplement: Supplementary file 1 [file bioengineering-08-00103-s001.zip › FileS2/figure_sampling-EX_glu__L_e.pdf]

# Exchange ( $EX_{g\text{ lyc}_e}$ )

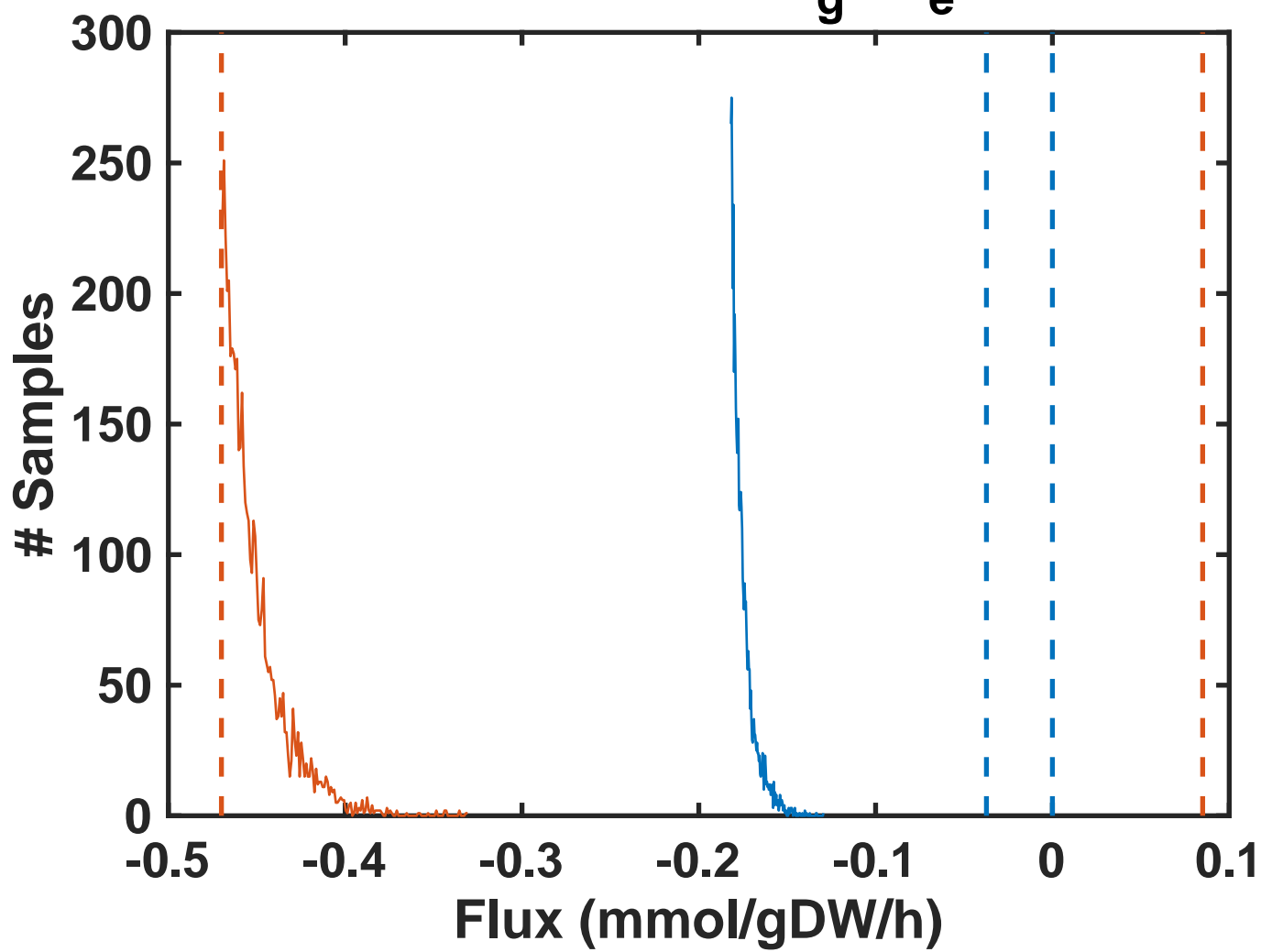

Supplement: Supplementary file 1 [file bioengineering-08-00103-s001.zip › FileS2/figure_sampling-EX_glyc_e.pdf]

# Exchange ( $EX_n h4_e$ )

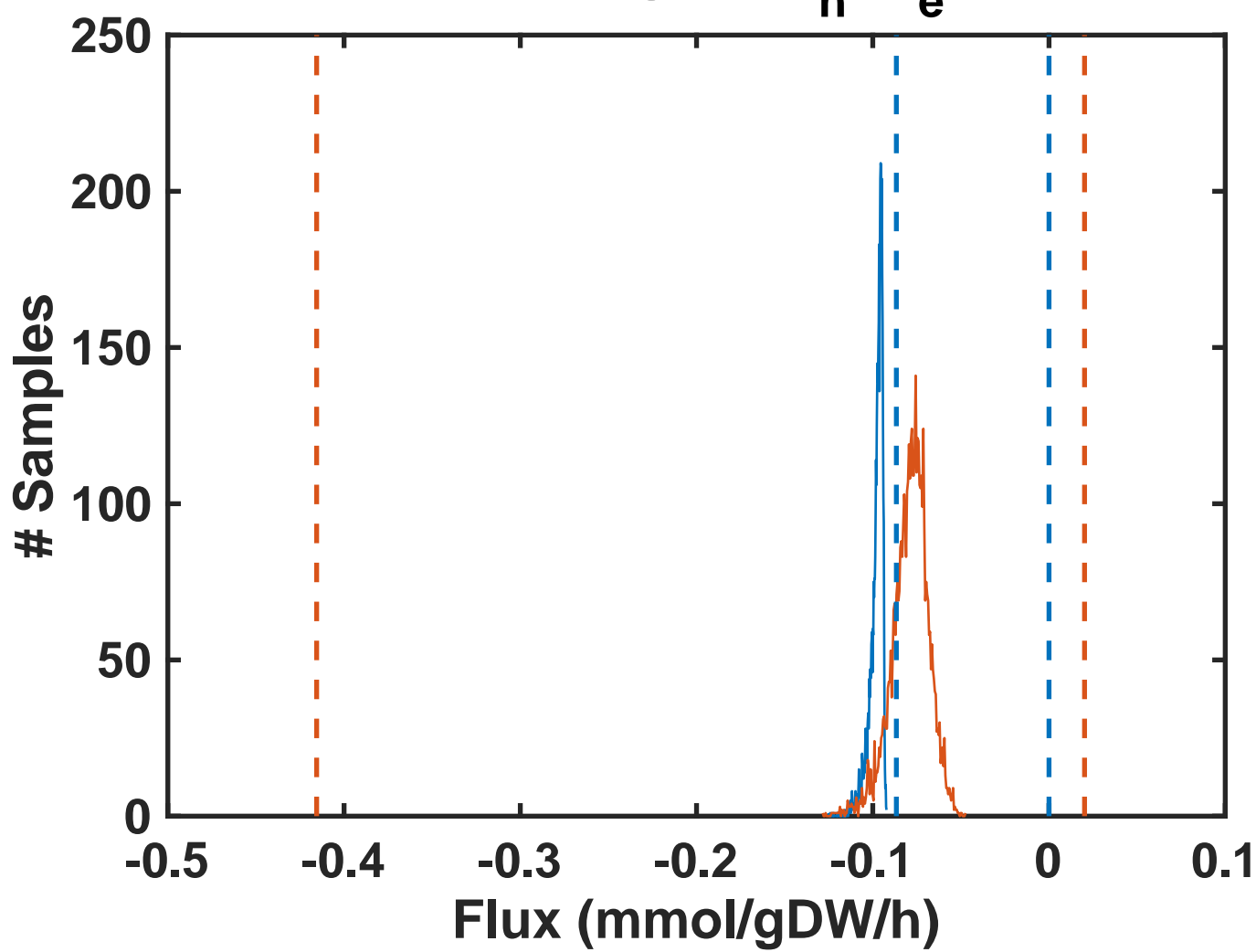

Supplement: Supplementary file 1 [file bioengineering-08-00103-s001.zip › FileS2/figure_sampling-EX_nh4_e.pdf]

# Exchange ( $EX_o 2_e$ )

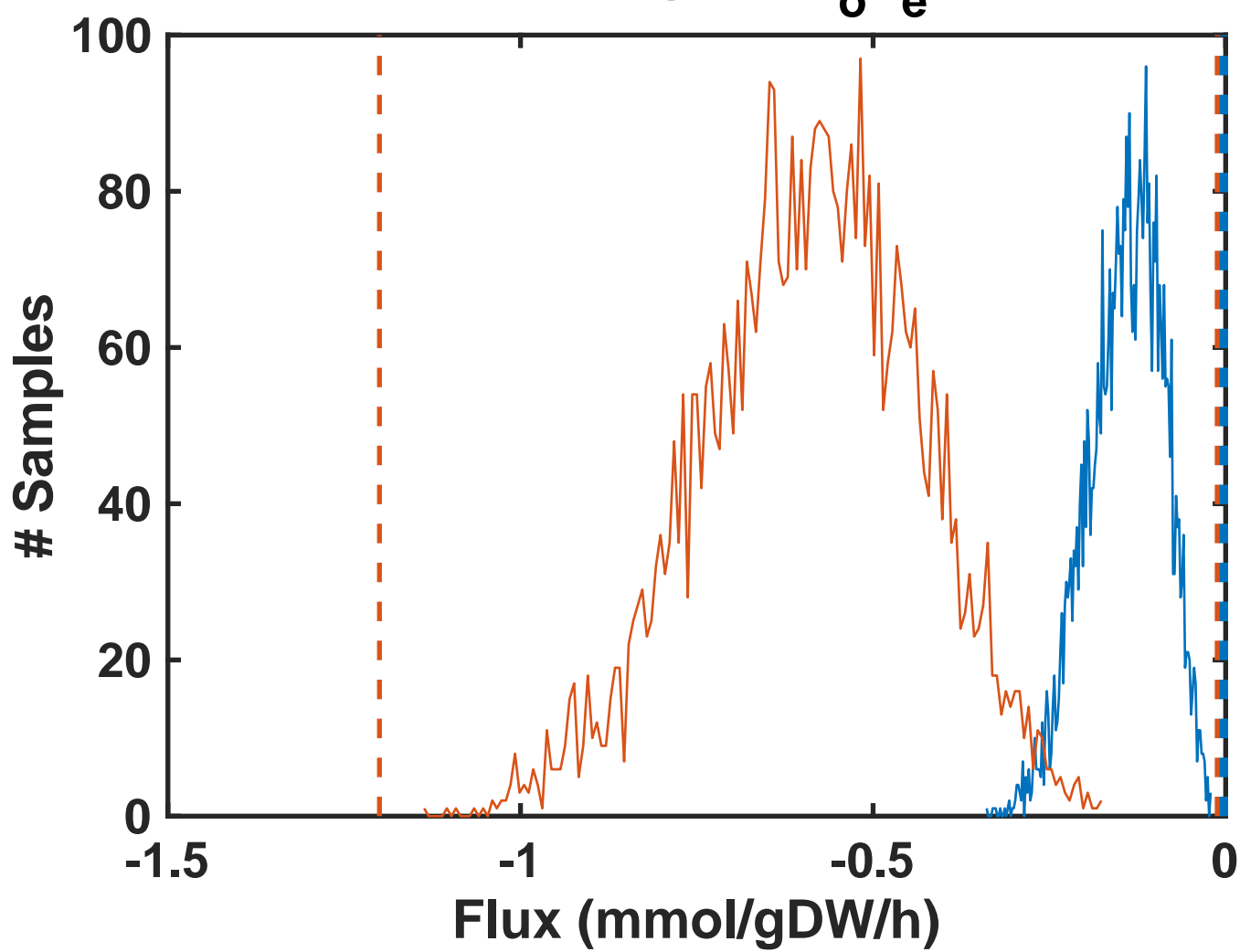

Supplement: Supplementary file 1 [file bioengineering-08-00103-s001.zip › FileS2/figure_sampling-EX_o2_e.pdf]

# Exchange ( $EX_{pe}$ )

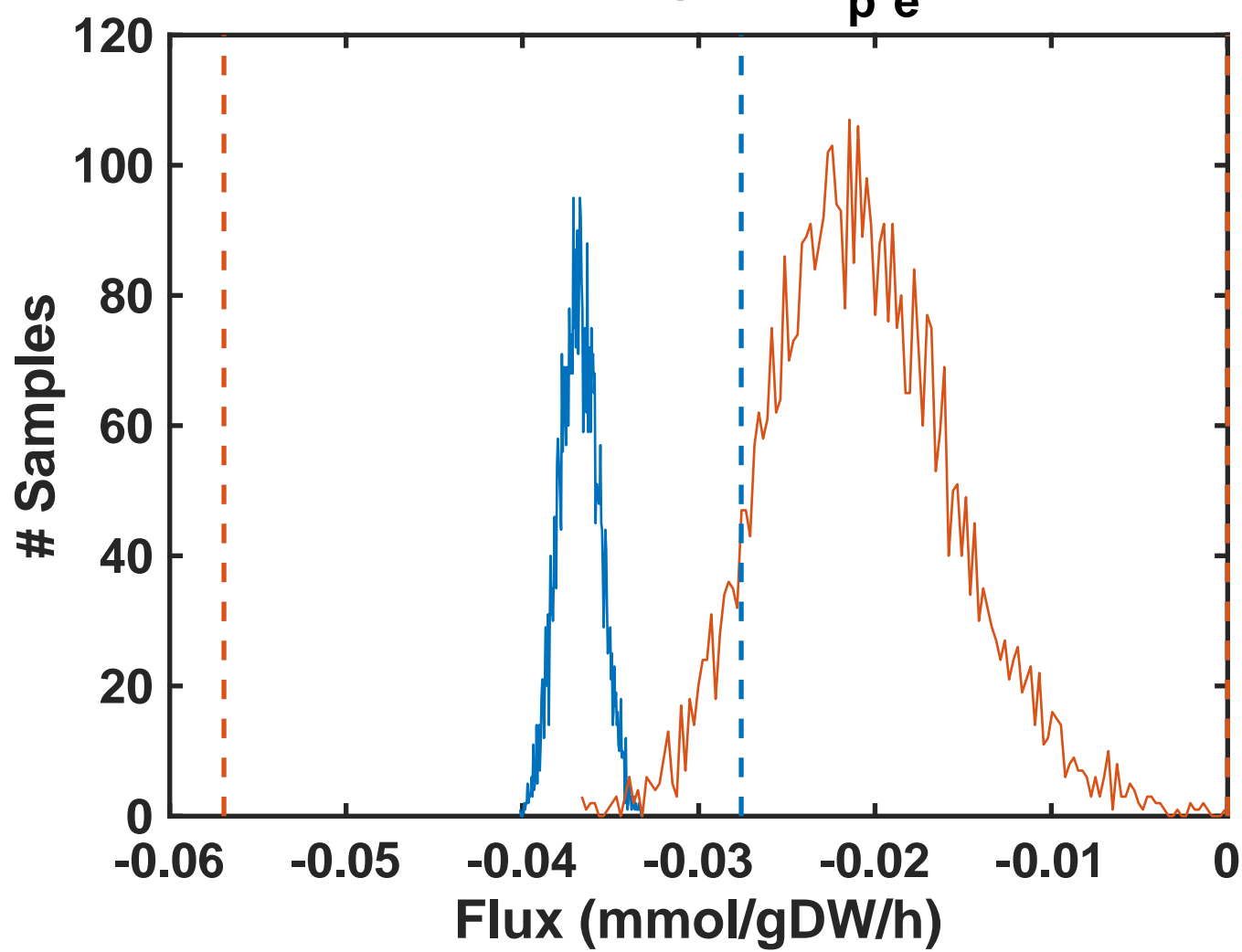

Supplement: Supplementary file 1 [file bioengineering-08-00103-s001.zip › FileS2/figure_sampling-EX_pi_e.pdf]

## Glycolysis/Gluconeogenesis (FBA)

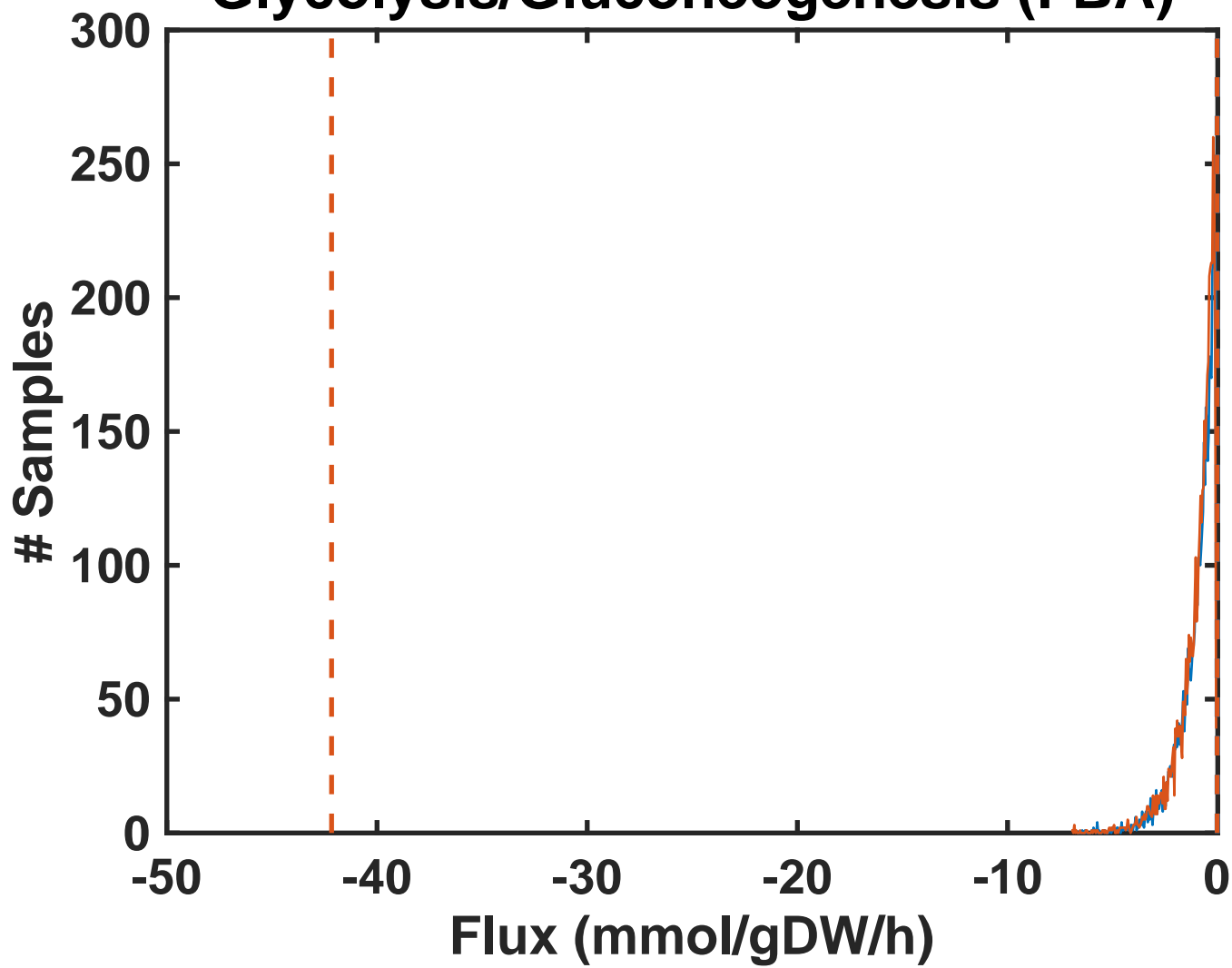

Supplement: Supplementary file 1 [file bioengineering-08-00103-s001.zip › FileS2/figure_sampling-FBA.pdf]

## Citric Acid Cycle (FUM)

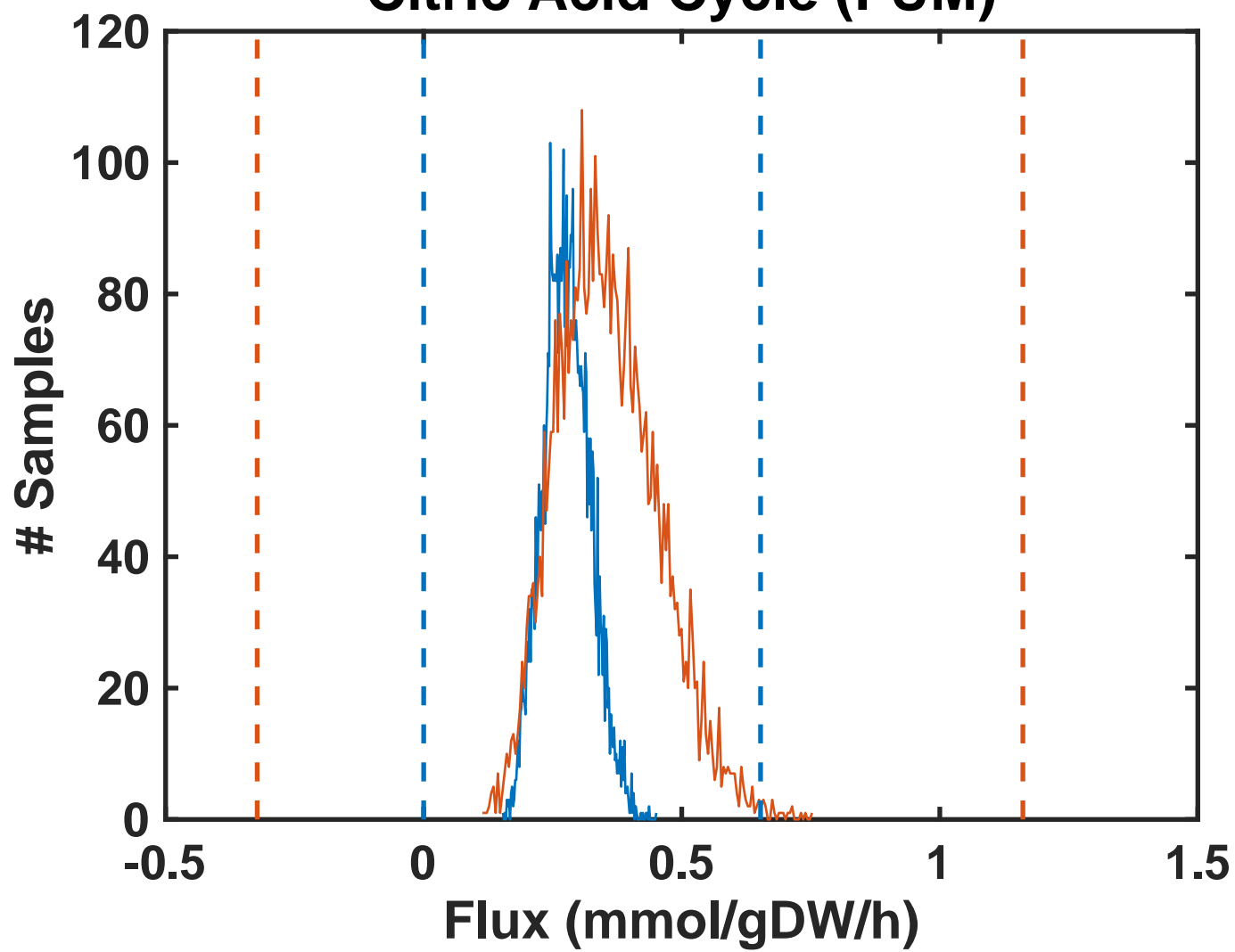

Supplement: Supplementary file 1 [file bioengineering-08-00103-s001.zip › FileS2/figure_sampling-FUM.pdf]

**Phosphoglycerolipid metabolism (G3PD1)**

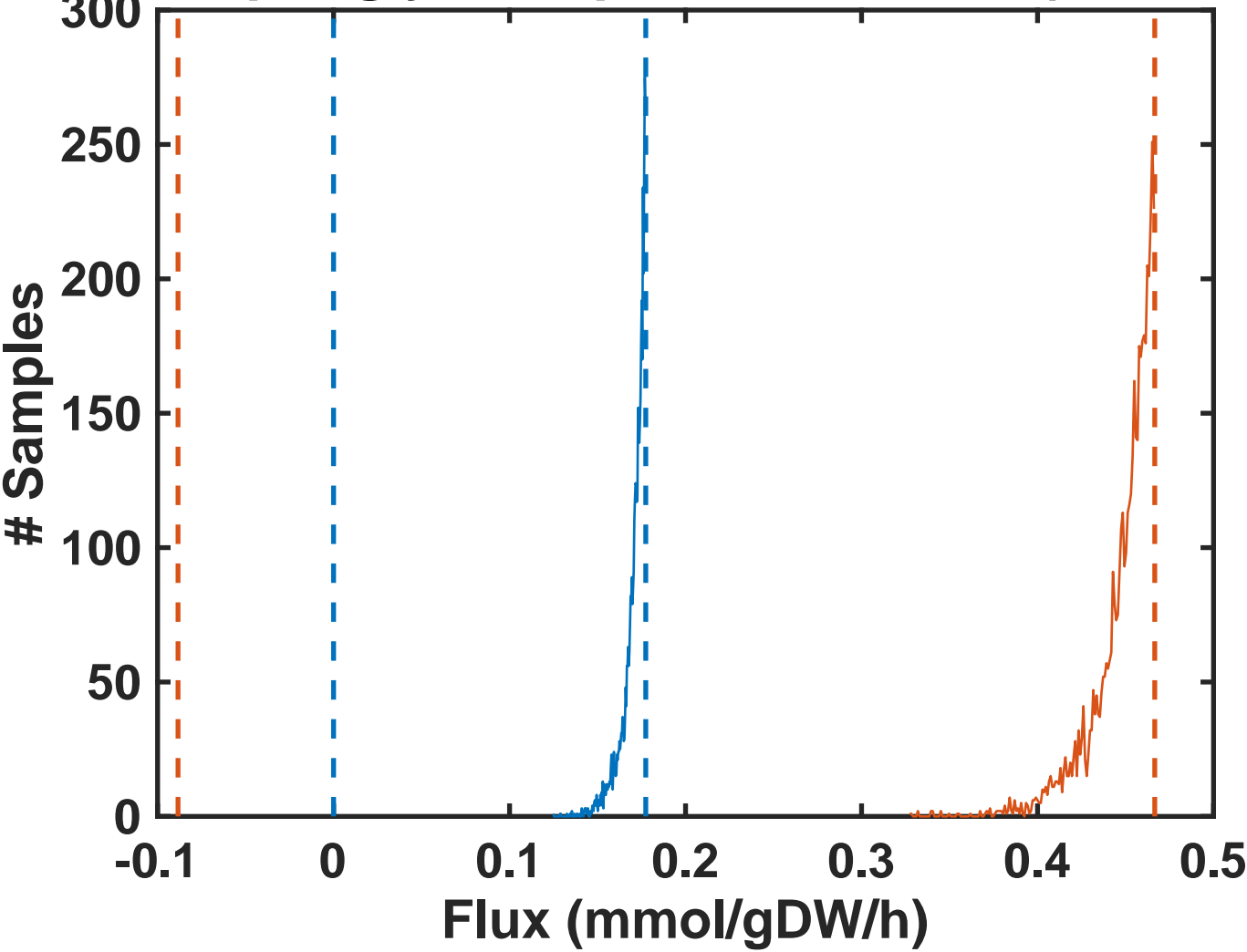

Supplement: Supplementary file 1 [file bioengineering-08-00103-s001.zip › FileS2/figure_sampling-G3PD1.pdf]

# Pentose Phosphate Pathway (G6PDH2r)

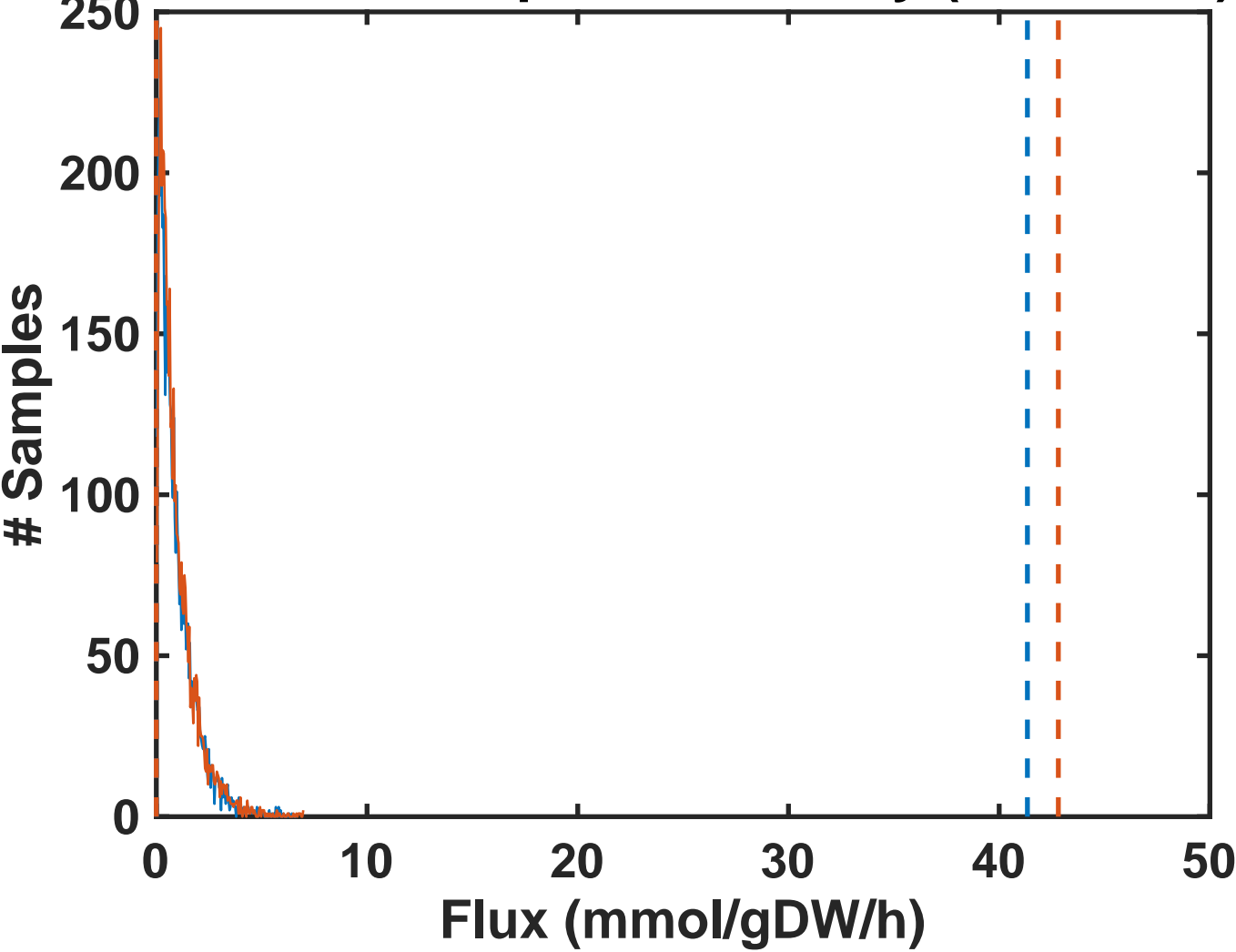

Supplement: Supplementary file 1 [file bioengineering-08-00103-s001.zip › FileS2/figure_sampling-G6PDH2r.pdf]

## Glycolysis/Gluconeogenesis (GAPD)

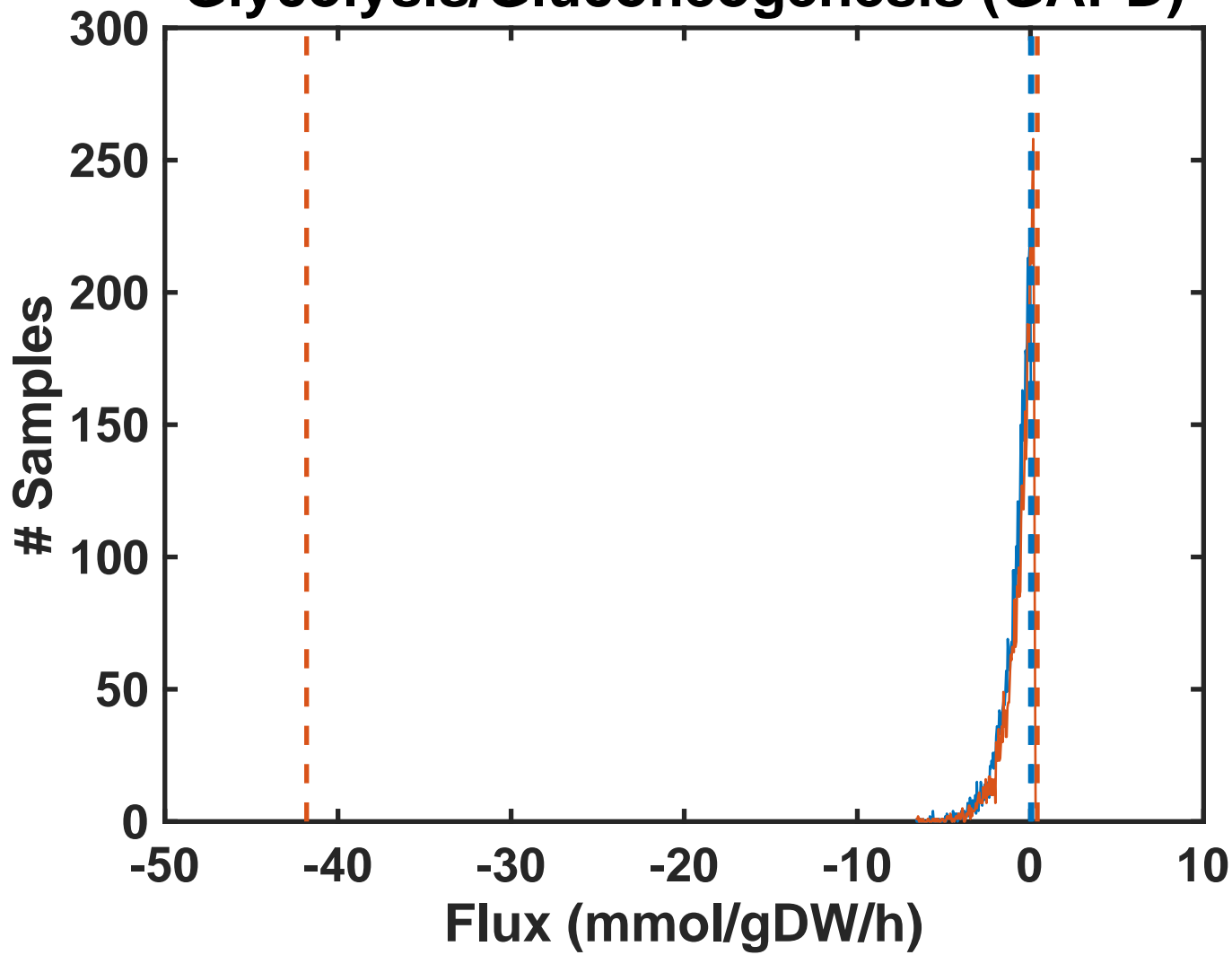

Supplement: Supplementary file 1 [file bioengineering-08-00103-s001.zip › FileS2/figure_sampling-GAPD.pdf]

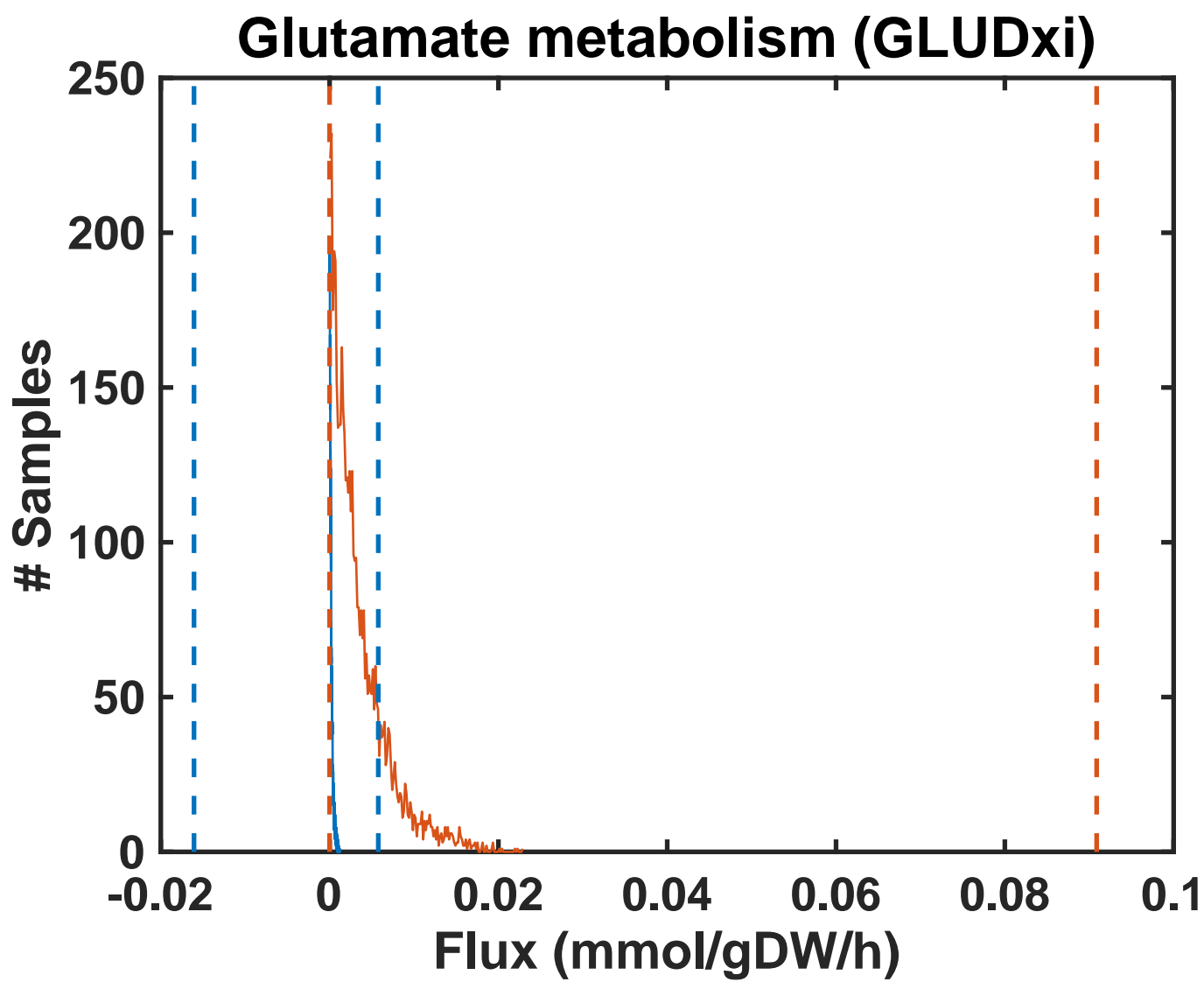

Supplement: Supplementary file 1 [file bioengineering-08-00103-s001.zip › FileS2/figure_sampling-GLUDxi.pdf]

## Pentose Phosphate Pathway (GND)

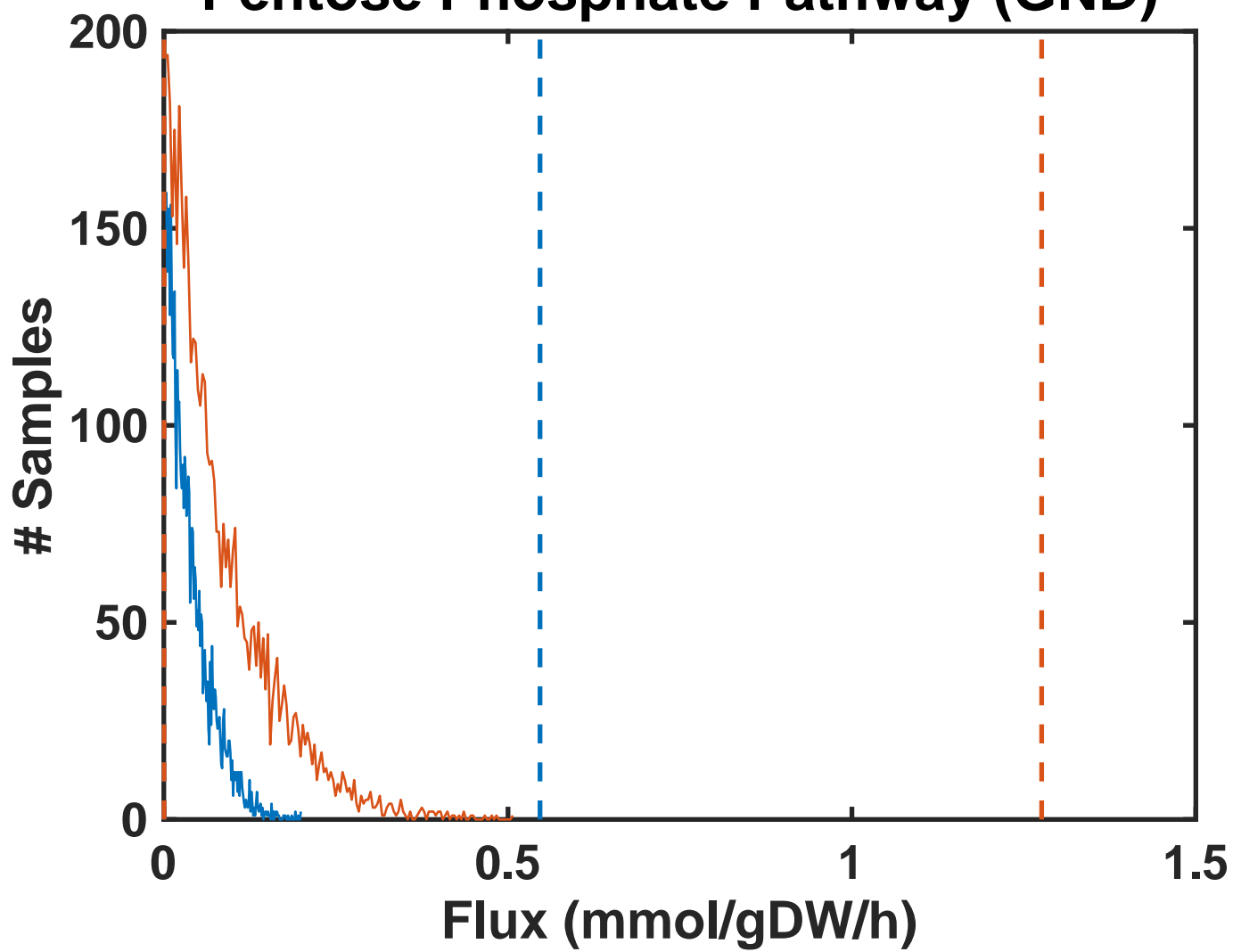

Supplement: Supplementary file 1 [file bioengineering-08-00103-s001.zip › FileS2/figure_sampling-GND.pdf]

## Citric Acid Cycle (ICDHyr)

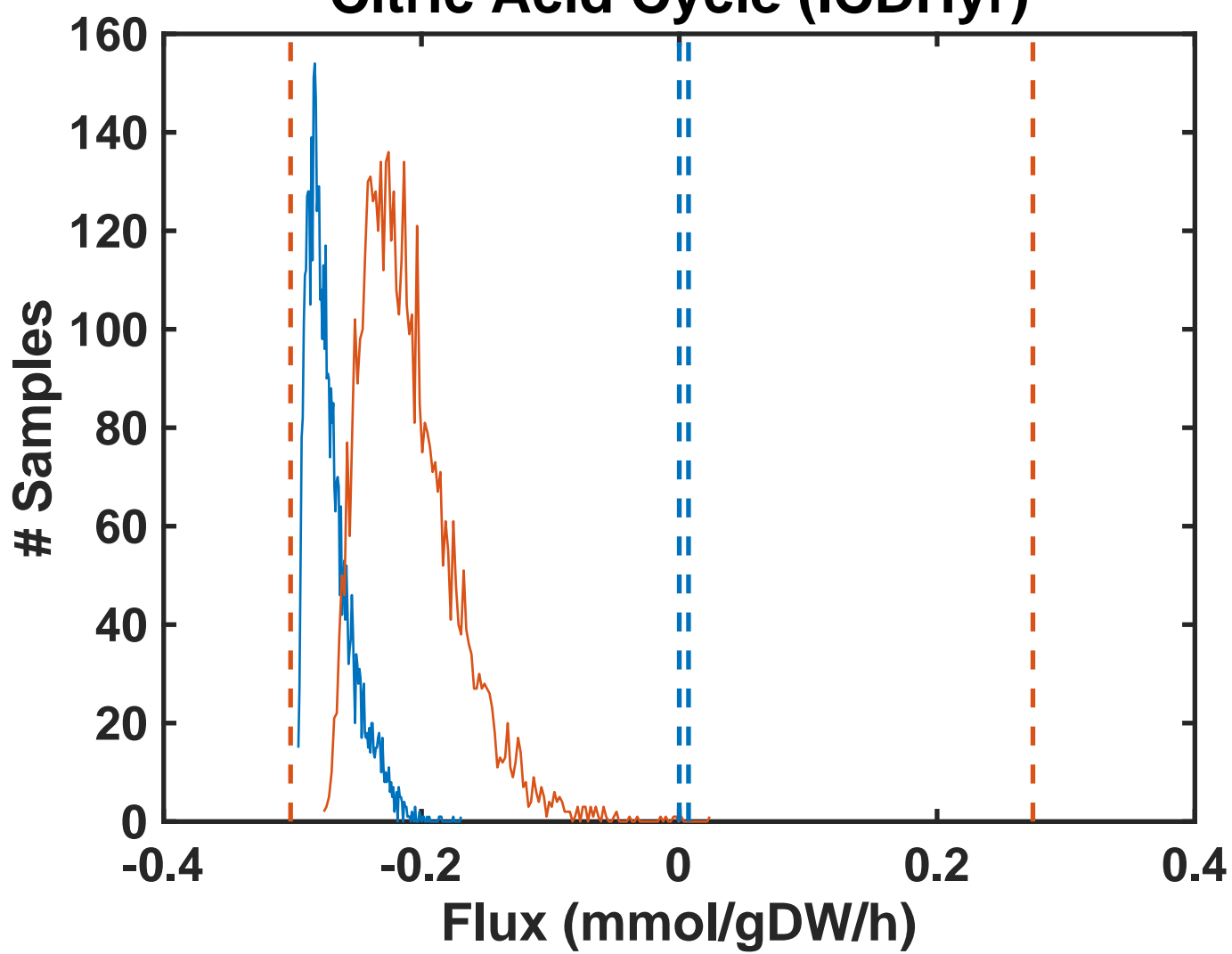

Supplement: Supplementary file 1 [file bioengineering-08-00103-s001.zip › FileS2/figure_sampling-ICDHyr.pdf]

## Anaplerotic Reactions (ICL)

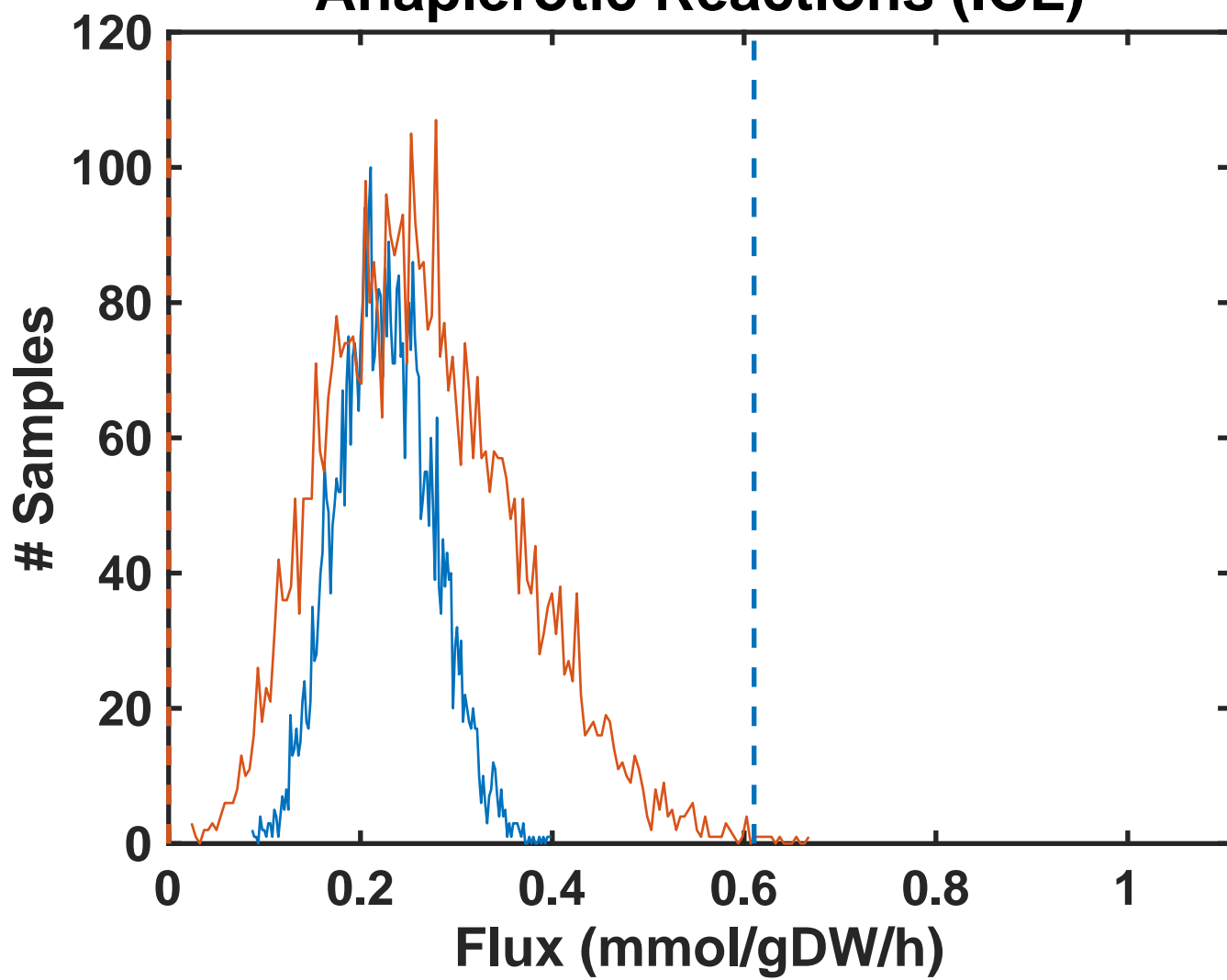

Supplement: Supplementary file 1 [file bioengineering-08-00103-s001.zip › FileS2/figure_sampling-ICL.pdf]

## Pyruvate Metabolism (LDH<sub>D</sub>)

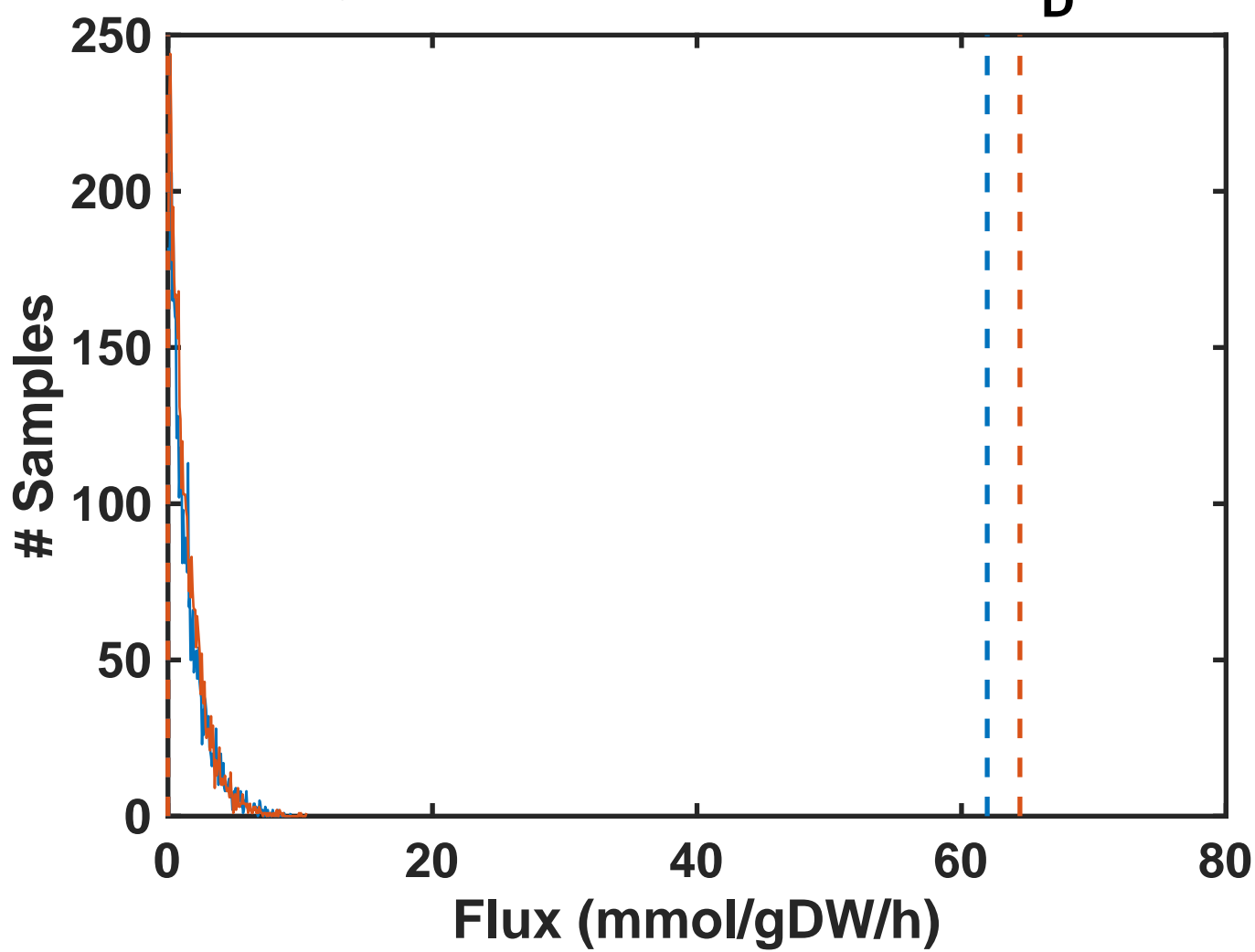

Supplement: Supplementary file 1 [file bioengineering-08-00103-s001.zip › FileS2/figure_sampling-LDH_D.pdf]

(LMPD<sub>1</sub>16<sub>g</sub>In-L<sub>c</sub>)

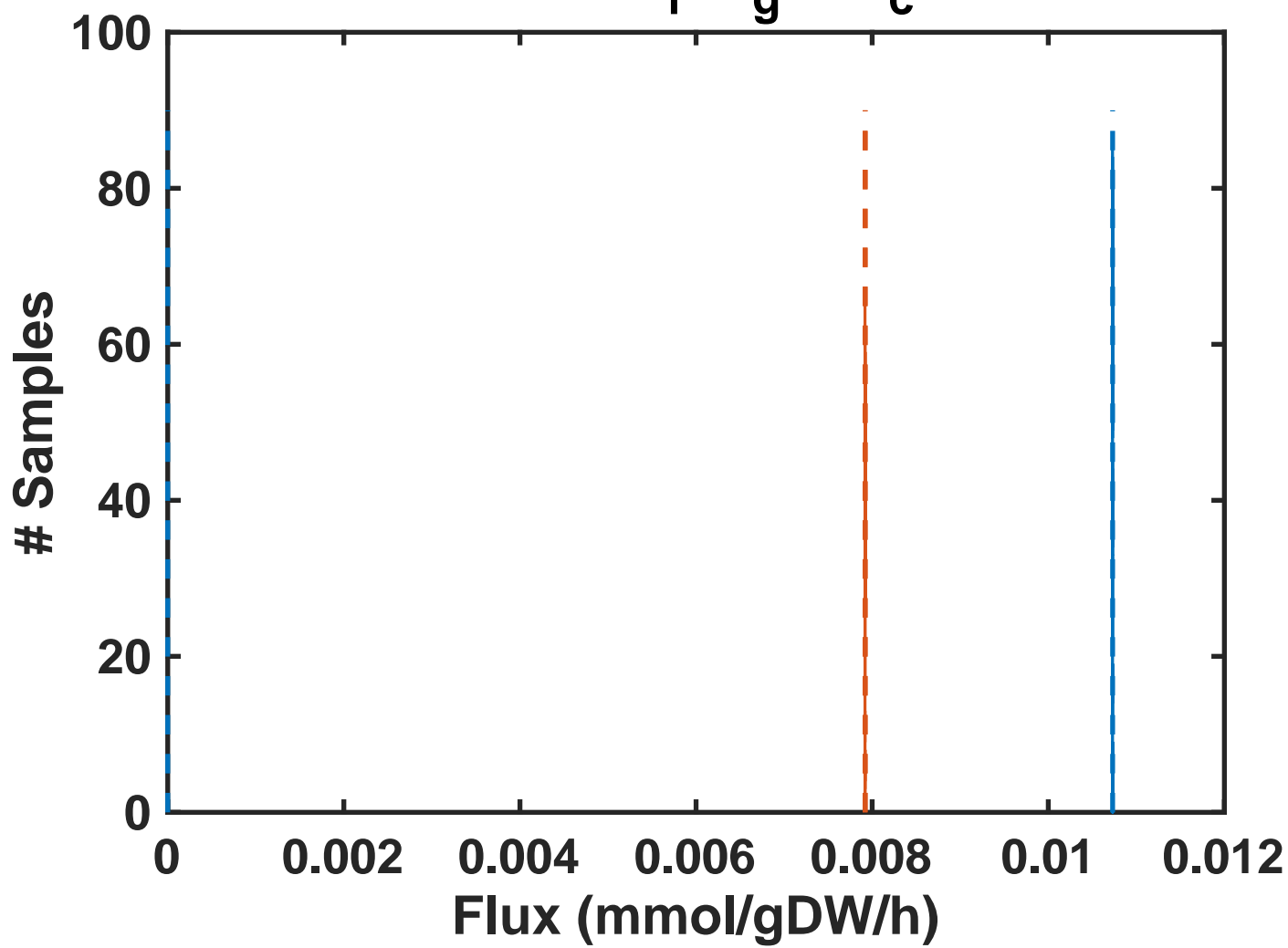

Supplement: Supplementary file 1 [file bioengineering-08-00103-s001.zip › FileS2/figure_sampling-LMPD_116_gln-L_c.pdf]

(LMPD<sub>3</sub>3<sub>a</sub>rg-L<sub>c</sub>)

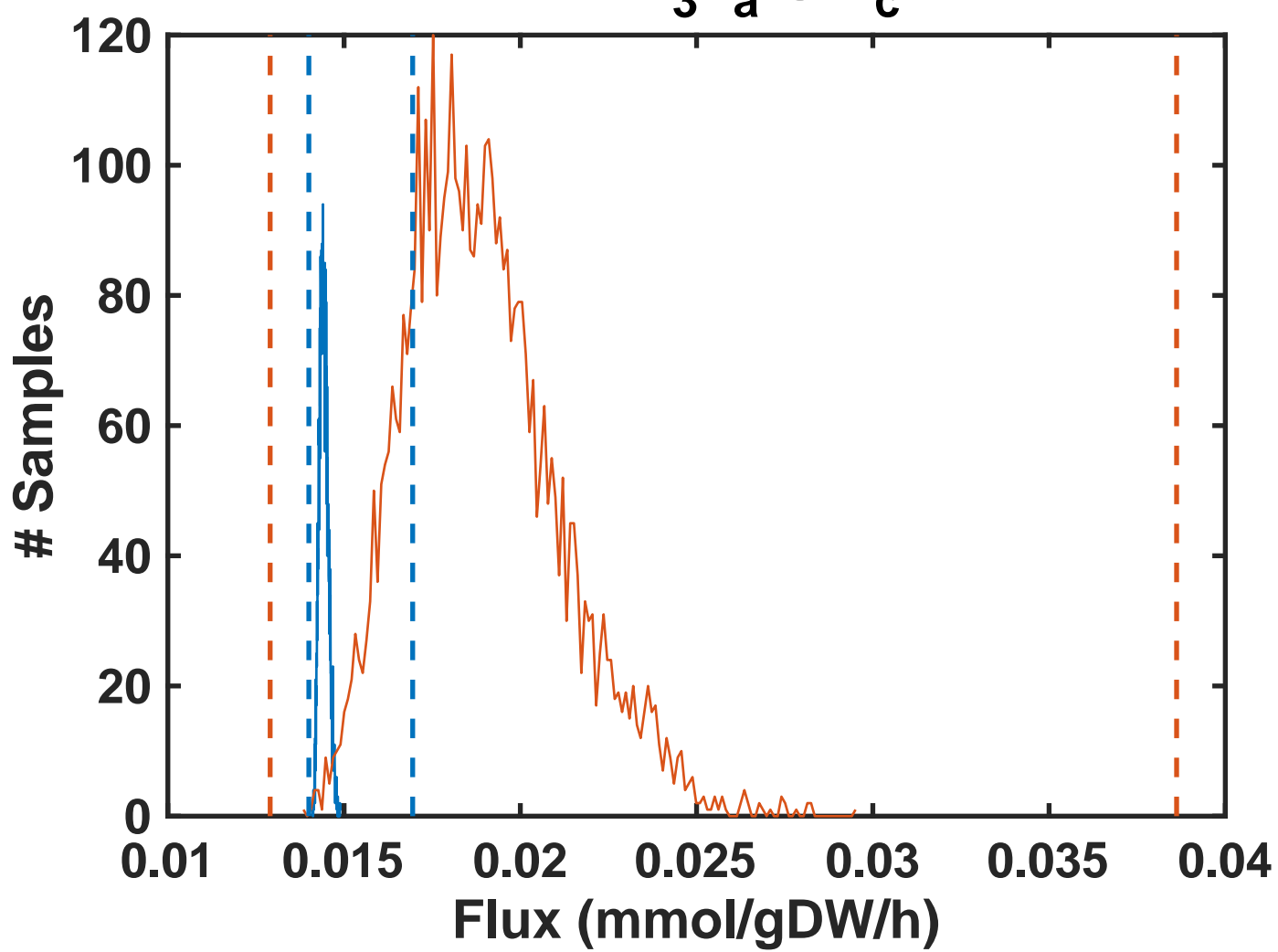

Supplement: Supplementary file 1 [file bioengineering-08-00103-s001.zip › FileS2/figure_sampling-LMPD_33_arg-L_c.pdf]

# Oxidative Phosphorylation ( $L_L$ ACD2)

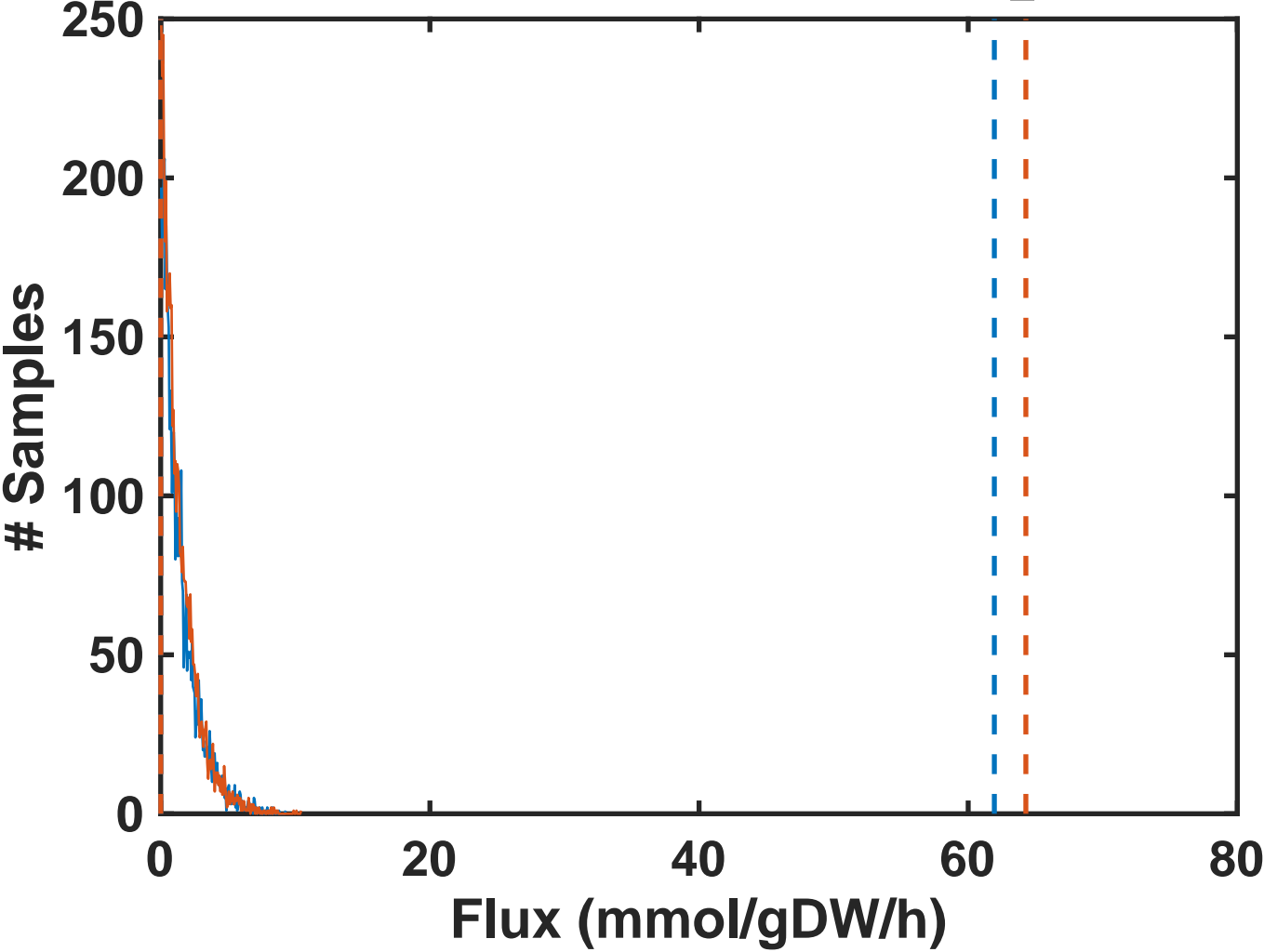

Supplement: Supplementary file 1 [file bioengineering-08-00103-s001.zip › FileS2/figure_sampling-L_LACD2.pdf]

## Anaplerotic Reactions (MALS)

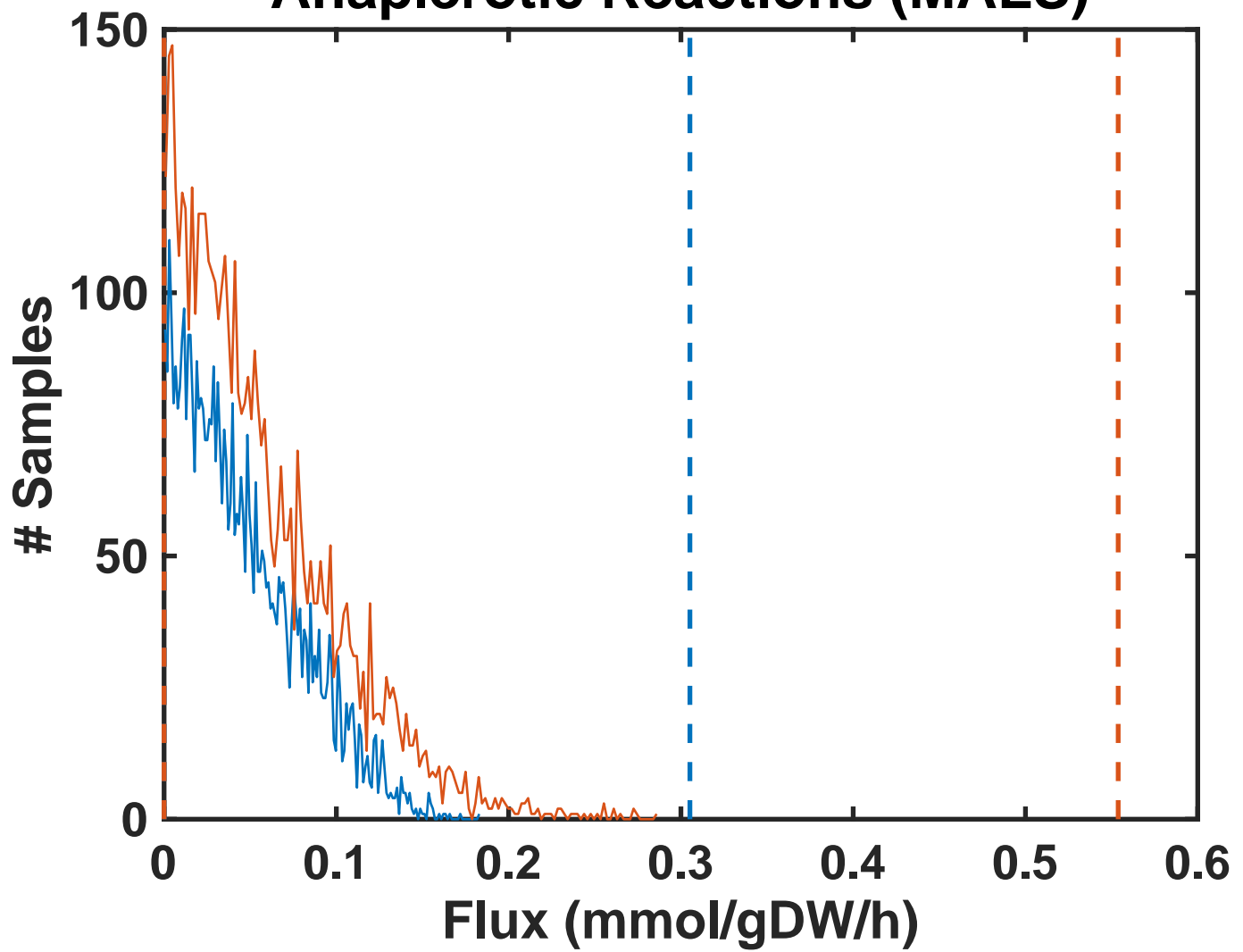

Supplement: Supplementary file 1 [file bioengineering-08-00103-s001.zip › FileS2/figure_sampling-MALS.pdf]

# Citric Acid Cycle (MDH)

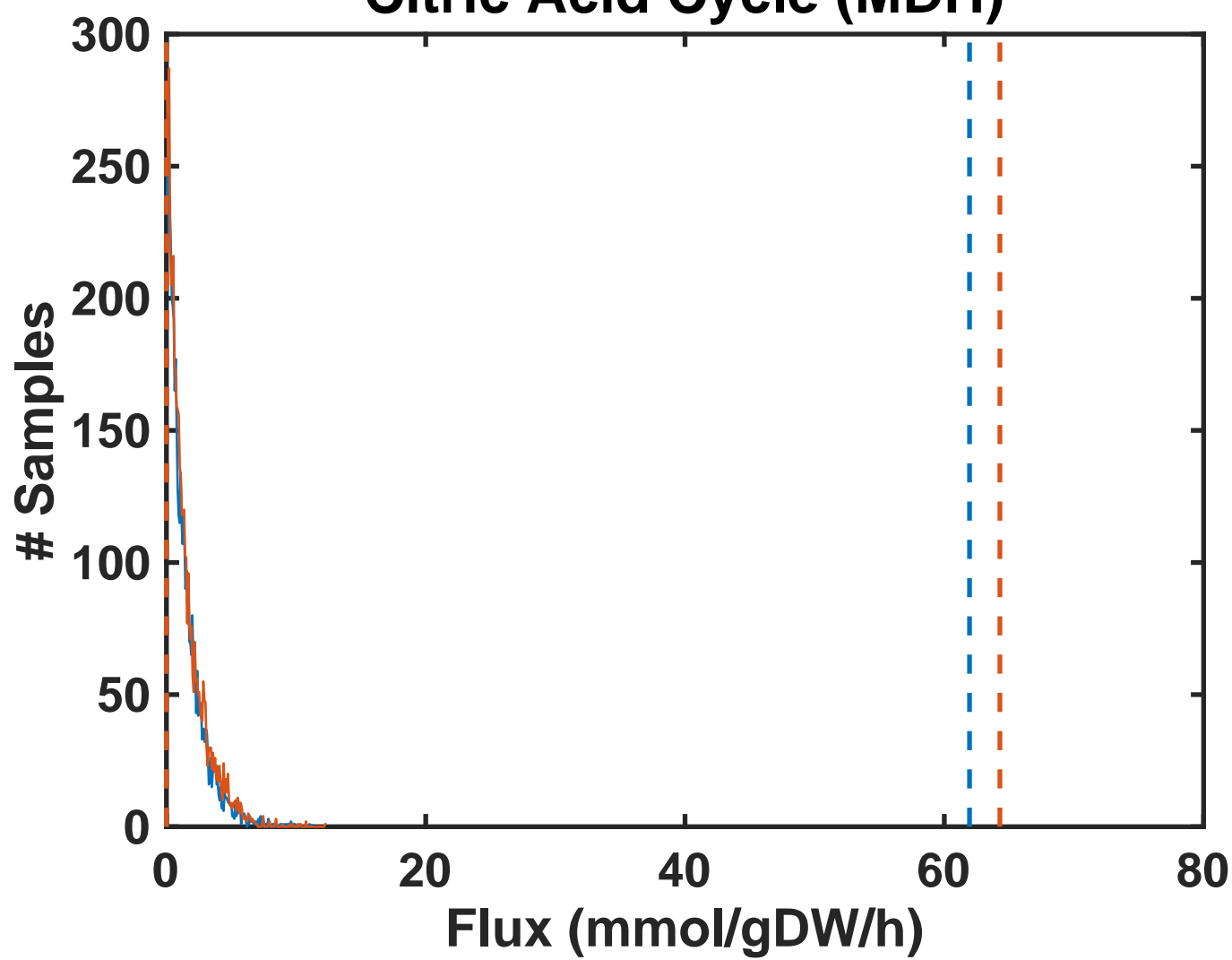

Supplement: Supplementary file 1 [file bioengineering-08-00103-s001.zip › FileS2/figure_sampling-MDH.pdf]

## Citric Acid Cycle (MDH2)

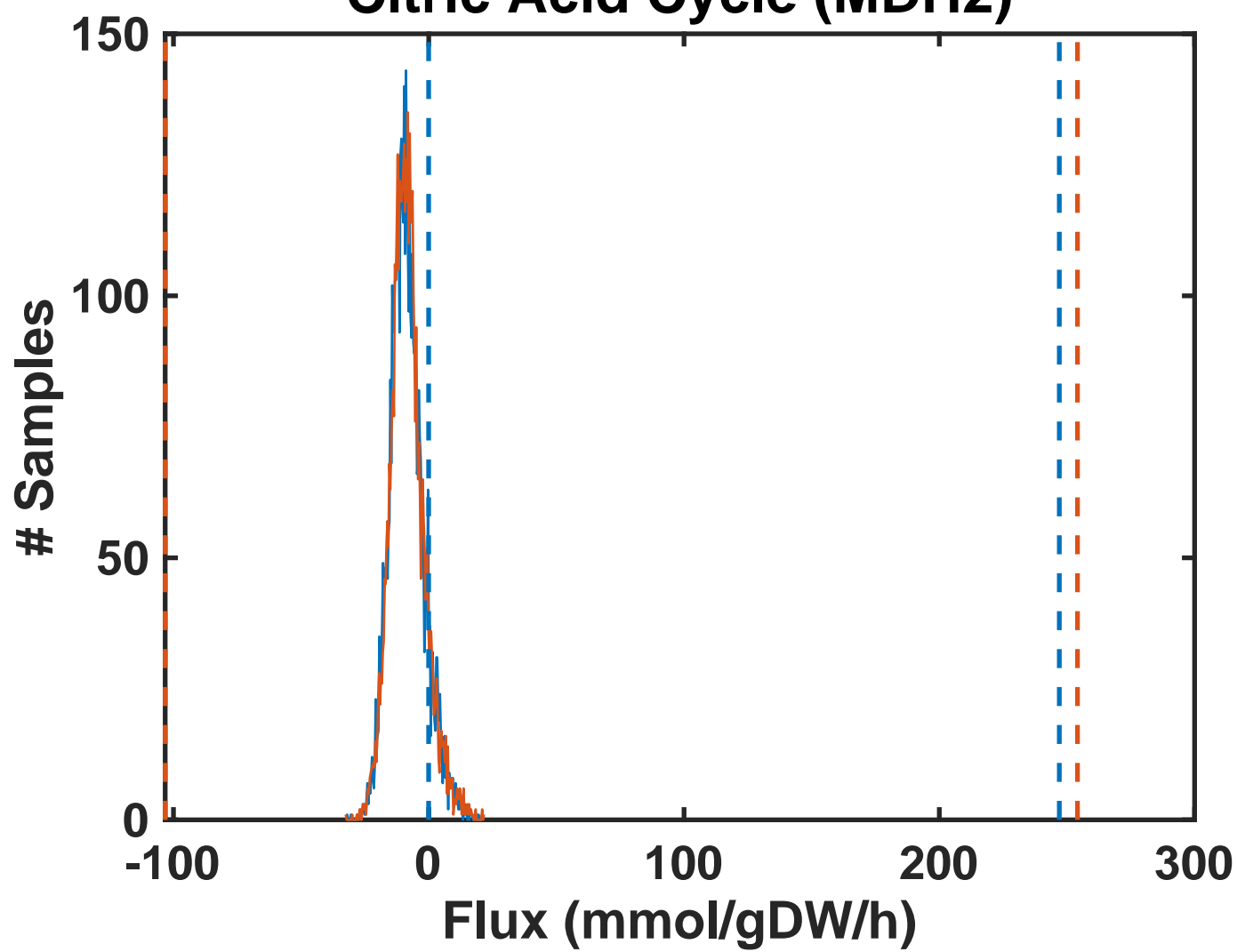

Supplement: Supplementary file 1 [file bioengineering-08-00103-s001.zip › FileS2/figure_sampling-MDH2.pdf]

## Citric Acid Cycle (MDH3)

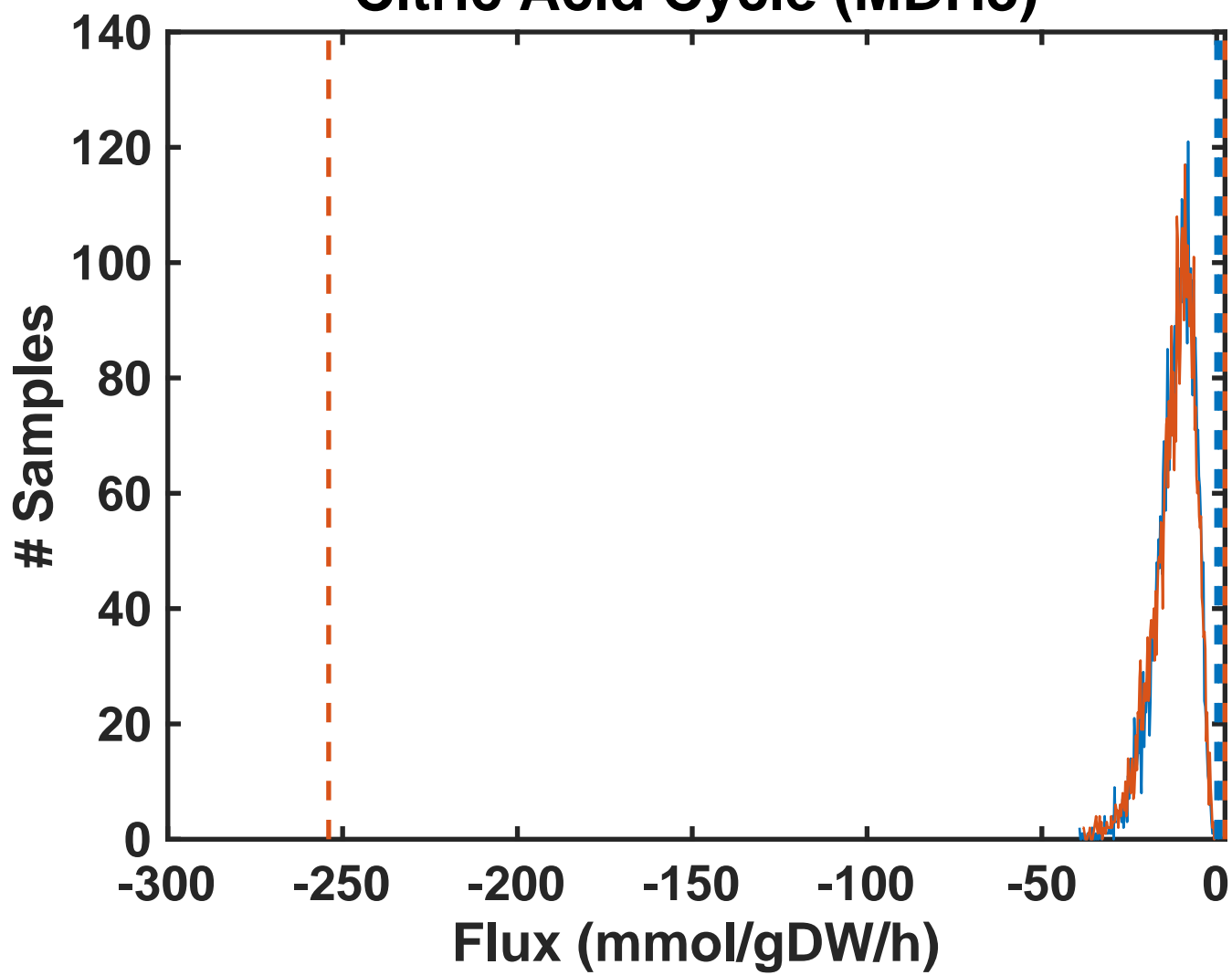

Supplement: Supplementary file 1 [file bioengineering-08-00103-s001.zip › FileS2/figure_sampling-MDH3.pdf]

## Anaplerotic Reactions (ME1)

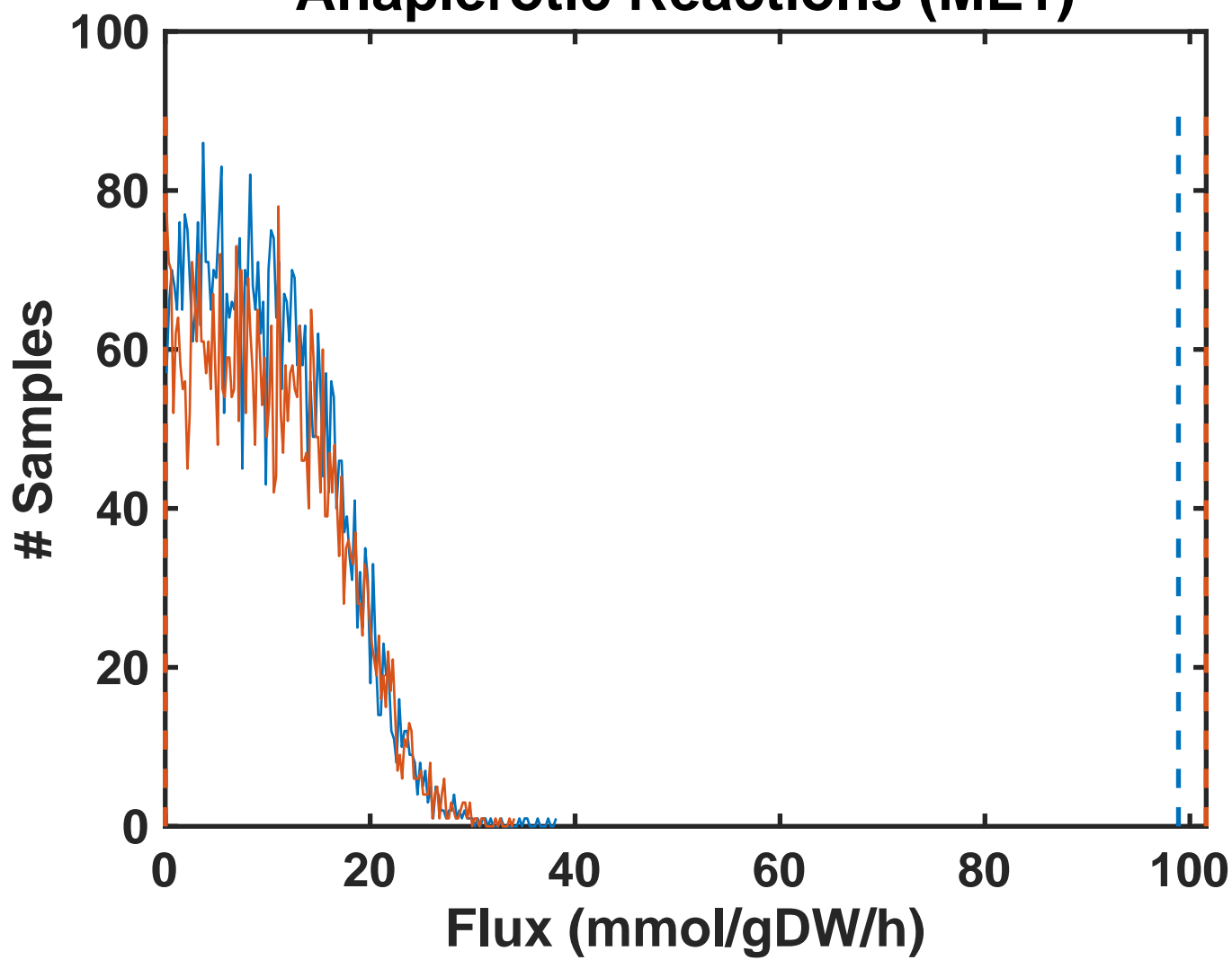

Supplement: Supplementary file 1 [file bioengineering-08-00103-s001.zip › FileS2/figure_sampling-ME1.pdf]

## Anaplerotic Reactions (ME2)

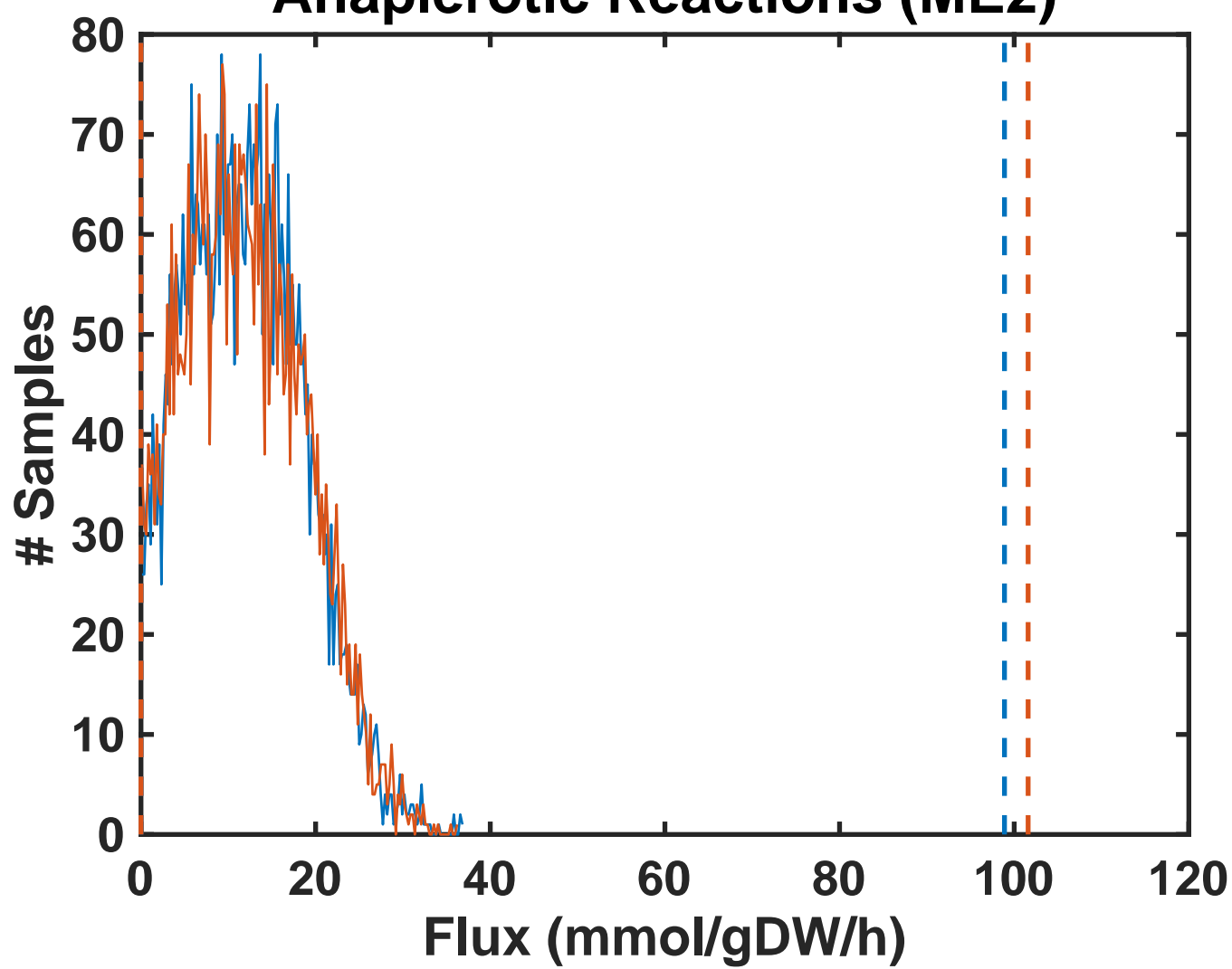

Supplement: Supplementary file 1 [file bioengineering-08-00103-s001.zip › FileS2/figure_sampling-ME2.pdf]

## Oxidative Phosphorylation (NADH5)

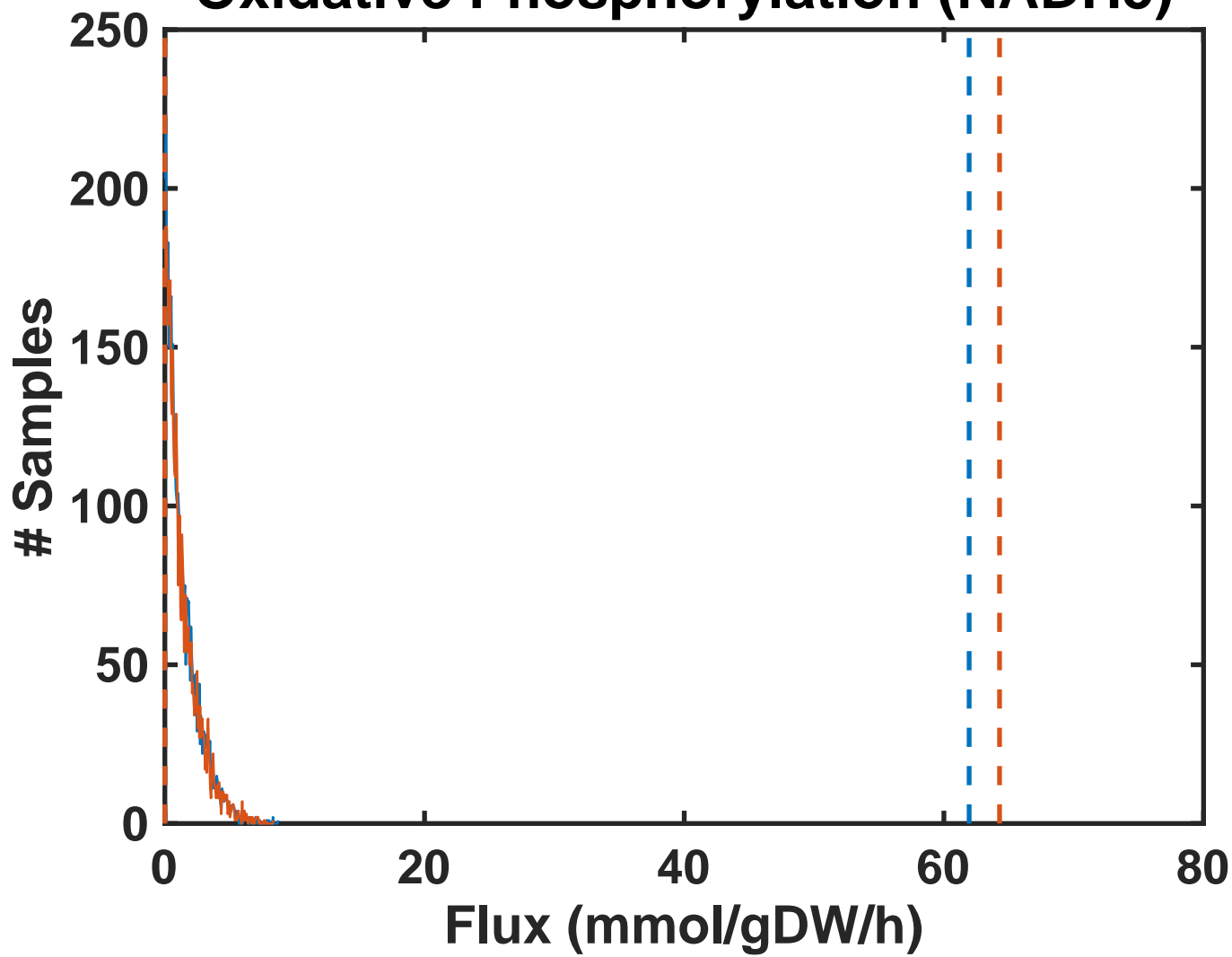

Supplement: Supplementary file 1 [file bioengineering-08-00103-s001.zip › FileS2/figure_sampling-NADH5.pdf]

## Glycolysis/Gluconeogenesis (PFK)

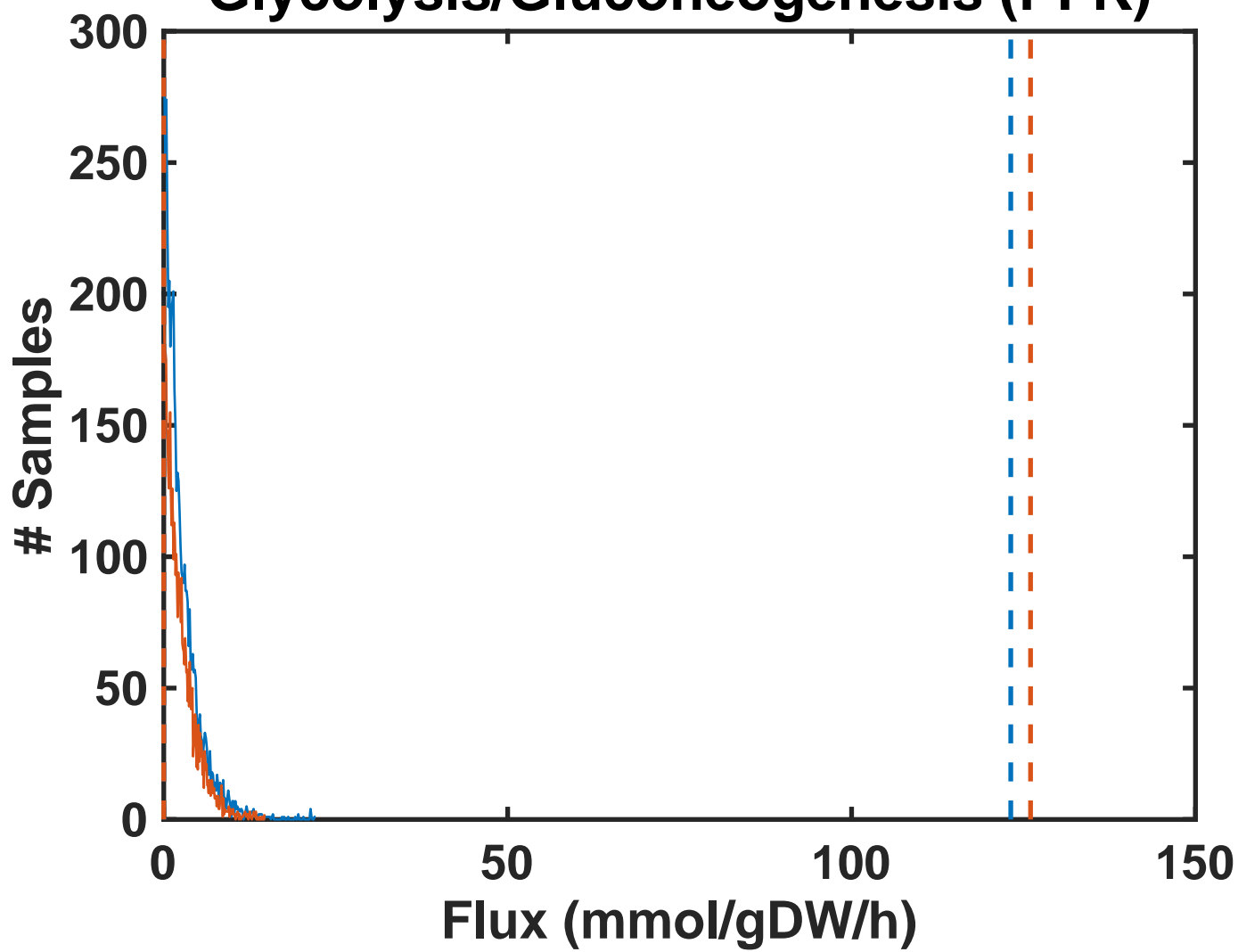

Supplement: Supplementary file 1 [file bioengineering-08-00103-s001.zip › FileS2/figure_sampling-PFK.pdf]

## Pyruvate Metabolism (PFL)

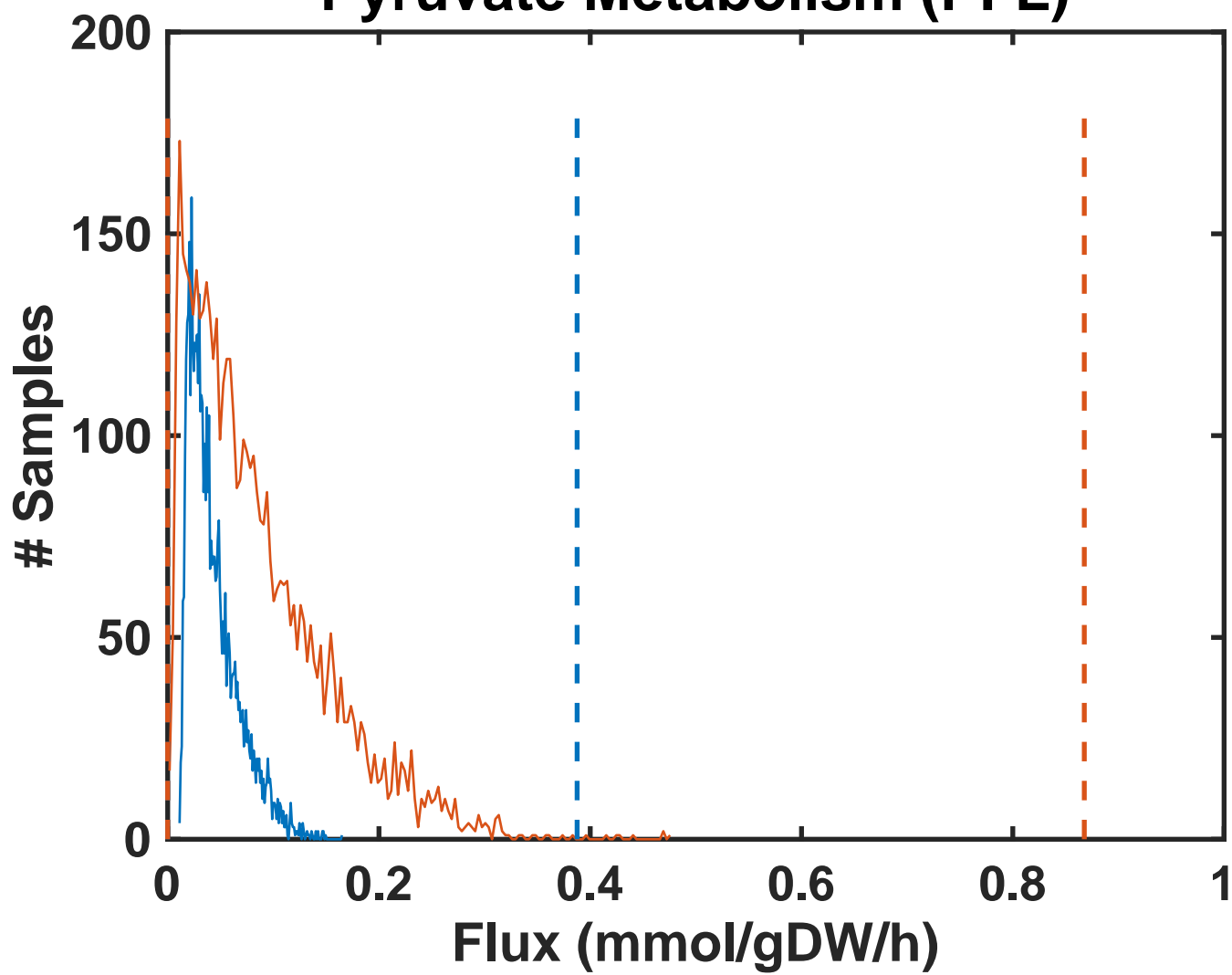

Supplement: Supplementary file 1 [file bioengineering-08-00103-s001.zip › FileS2/figure_sampling-PFL.pdf]

# Glycolysis/Gluconeogenesis (PGI)

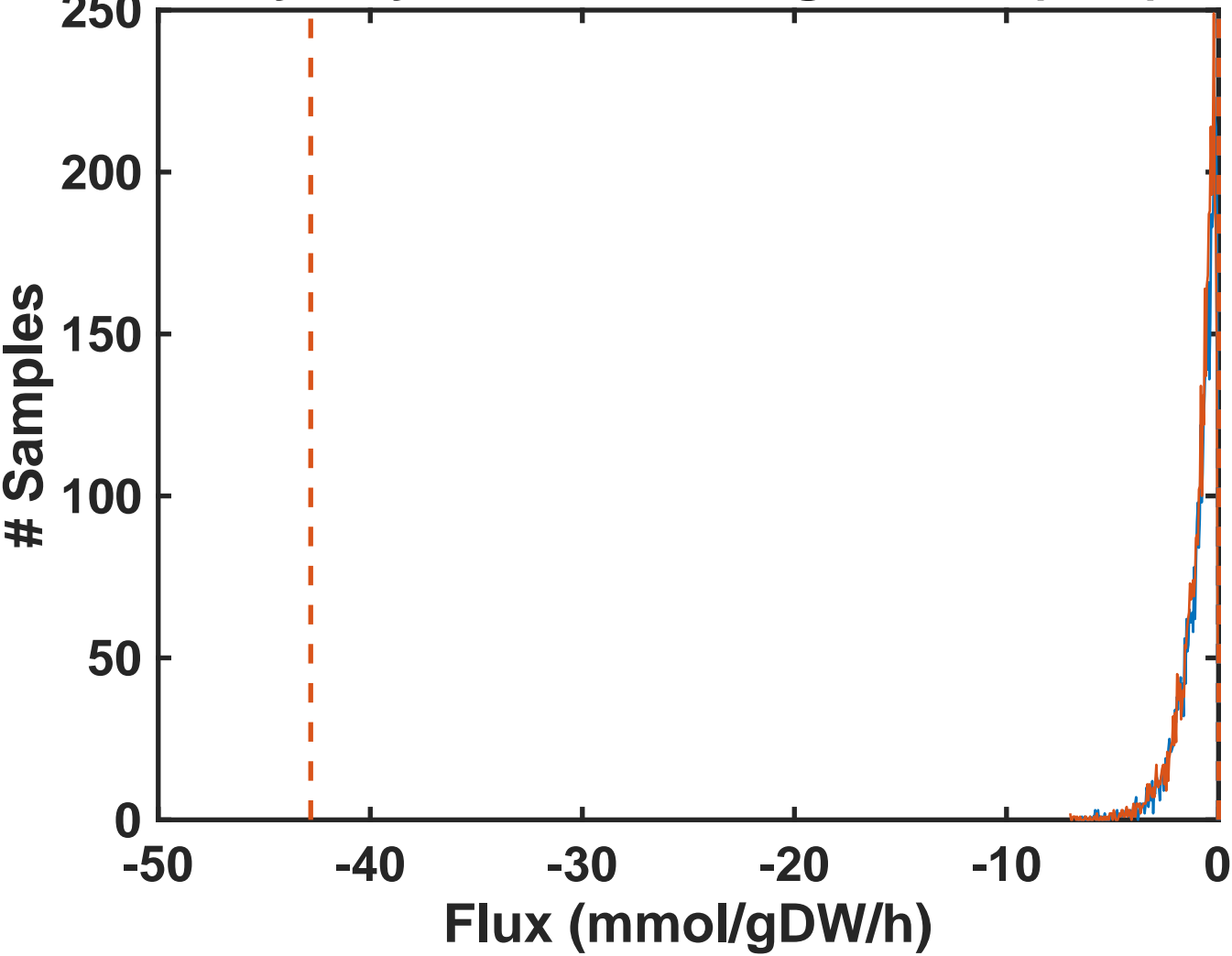

Supplement: Supplementary file 1 [file bioengineering-08-00103-s001.zip › FileS2/figure_sampling-PGI.pdf]

## Glycolysis/Gluconeogenesis (PGK)

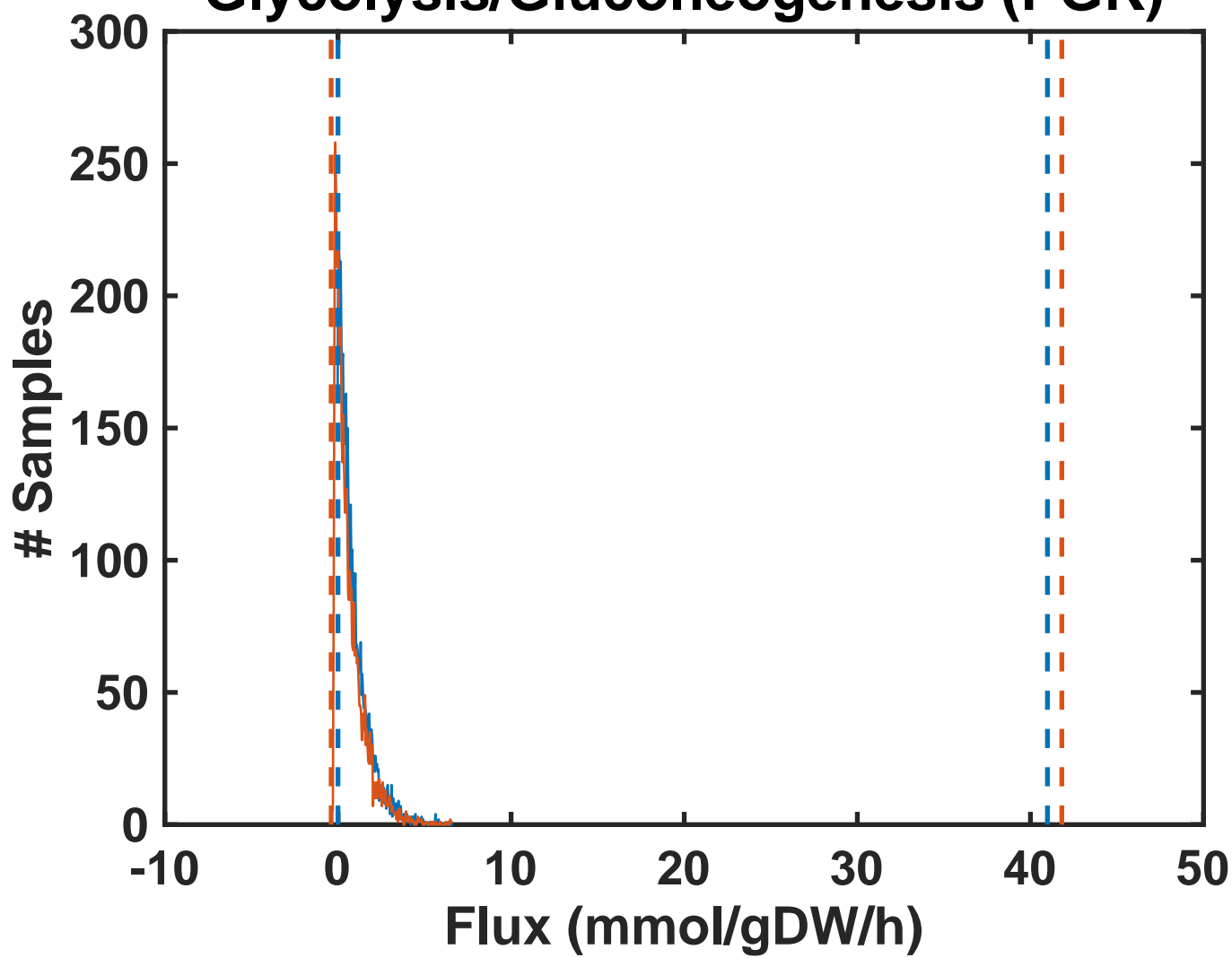

Supplement: Supplementary file 1 [file bioengineering-08-00103-s001.zip › FileS2/figure_sampling-PGK.pdf]

## Pentose Phosphate Pathway (PGL)

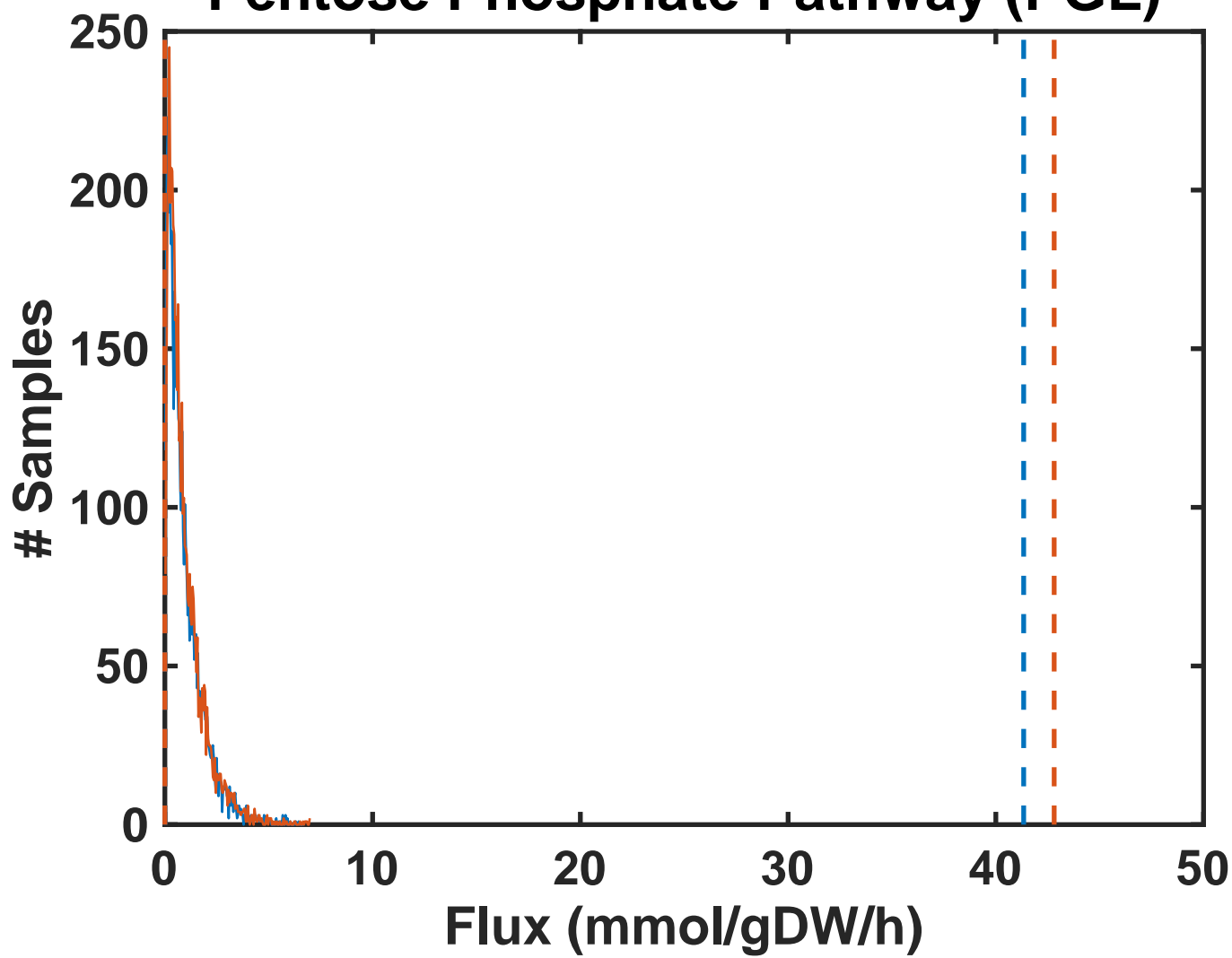

Supplement: Supplementary file 1 [file bioengineering-08-00103-s001.zip › FileS2/figure_sampling-PGL.pdf]

## Glycolysis/Gluconeogenesis (PGM)

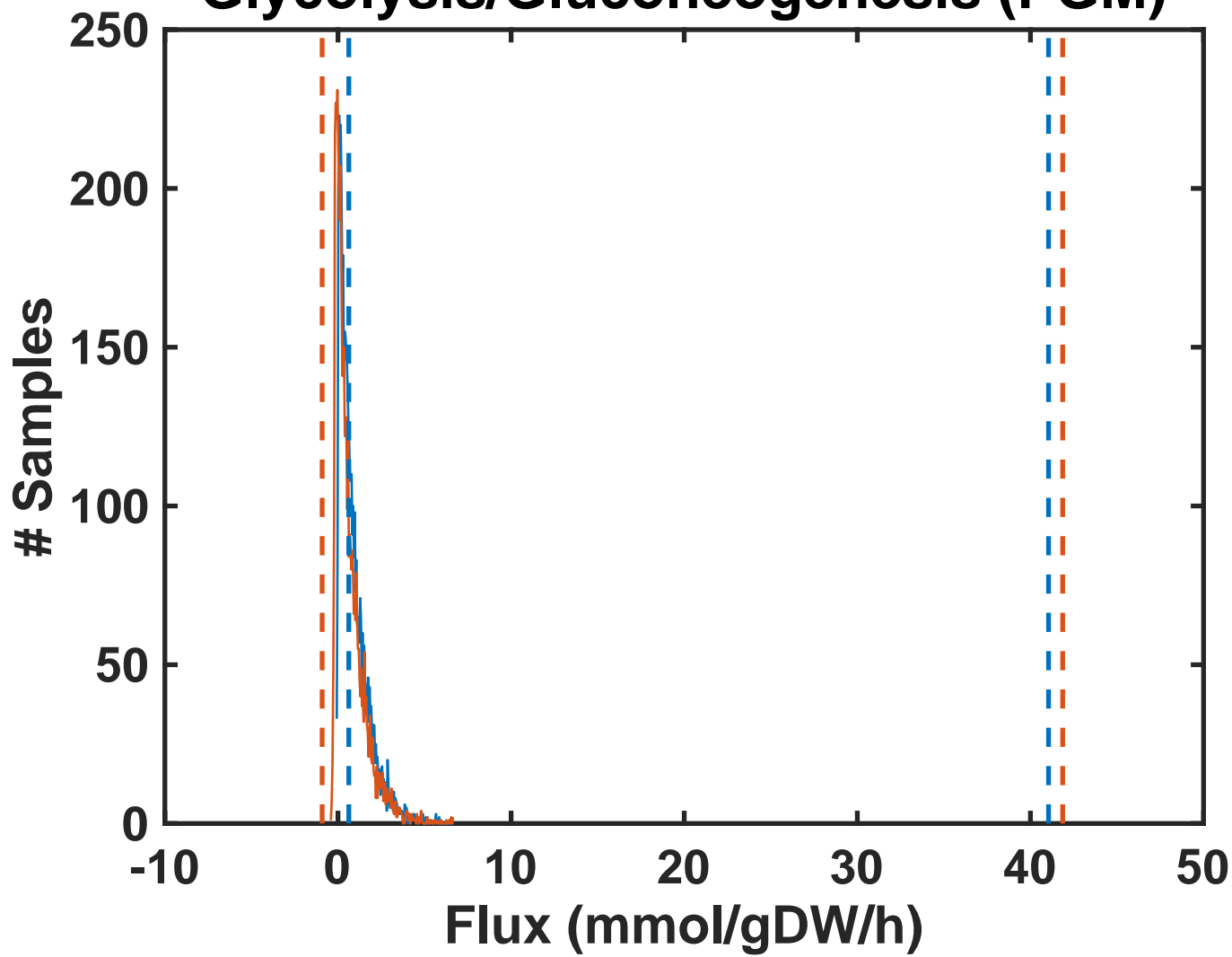

Supplement: Supplementary file 1 [file bioengineering-08-00103-s001.zip › FileS2/figure_sampling-PGM.pdf]

## Anaplerotic Reactions (PPC)

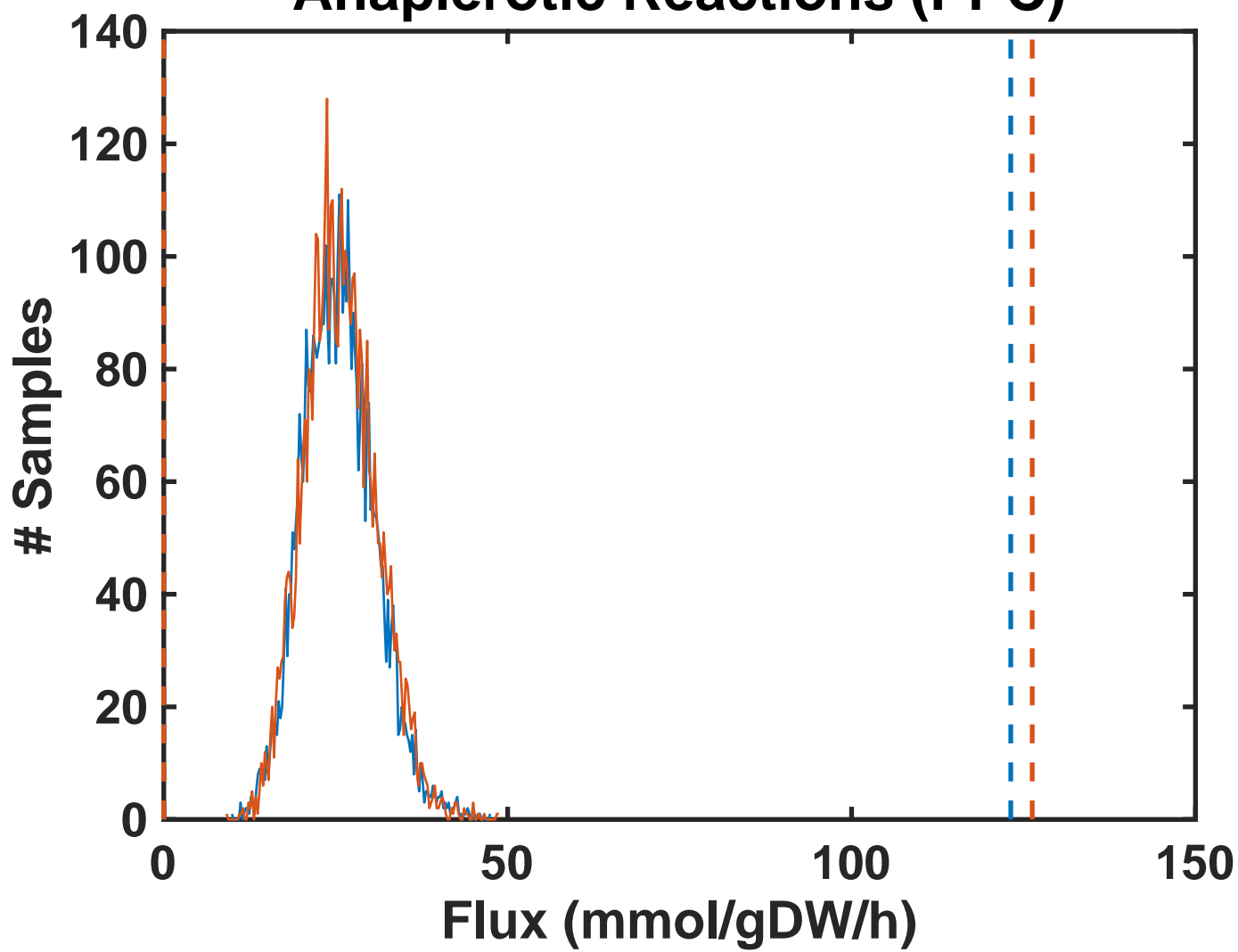

Supplement: Supplementary file 1 [file bioengineering-08-00103-s001.zip › FileS2/figure_sampling-PPC.pdf]

## Anaplerotic Reactions (PPCK)

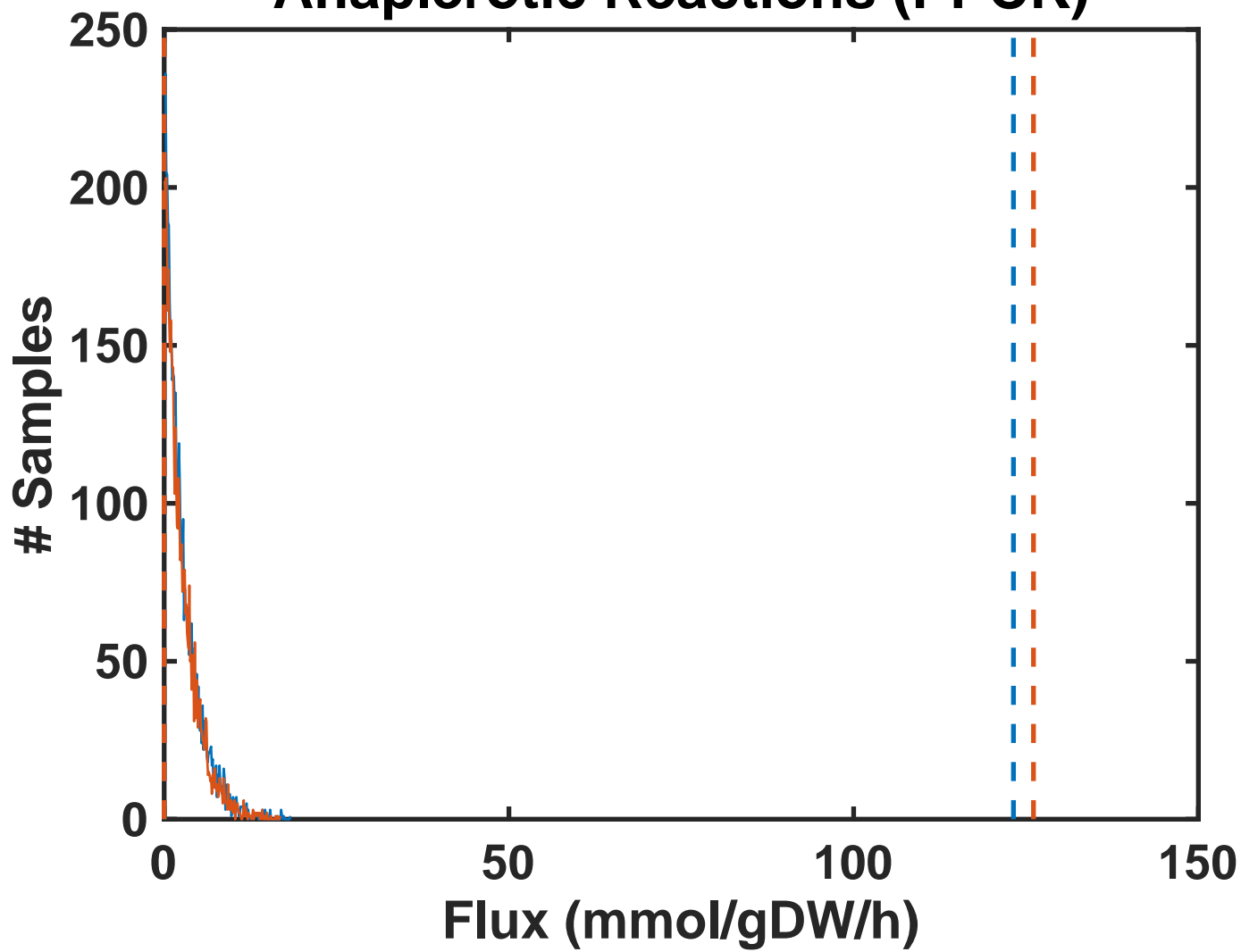

Supplement: Supplementary file 1 [file bioengineering-08-00103-s001.zip › FileS2/figure_sampling-PPCK.pdf]

## Pyruvate Metabolism (PTAr)

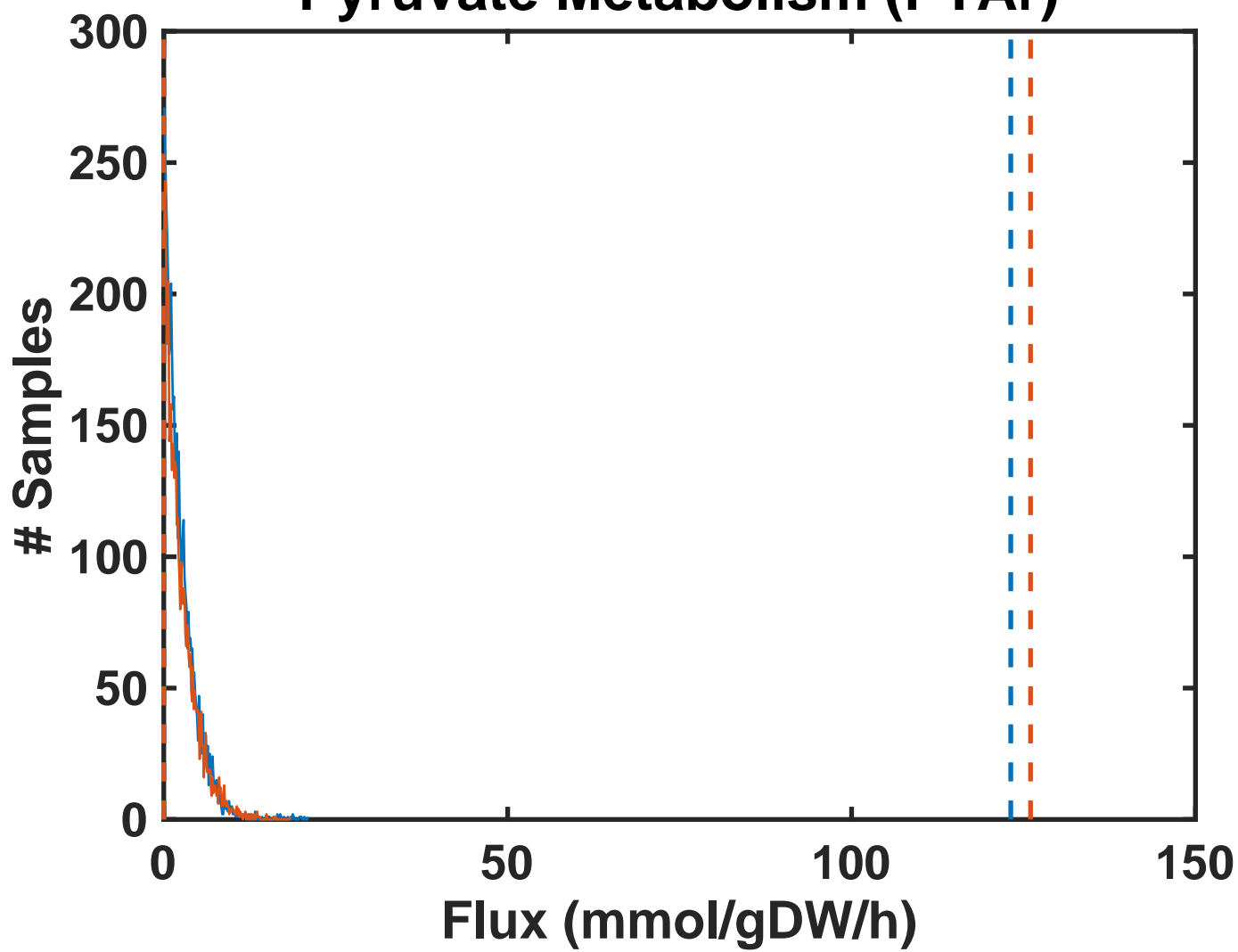

Supplement: Supplementary file 1 [file bioengineering-08-00103-s001.zip › FileS2/figure_sampling-PTAr.pdf]

## Glycolysis/Gluconeogenesis (PYK)

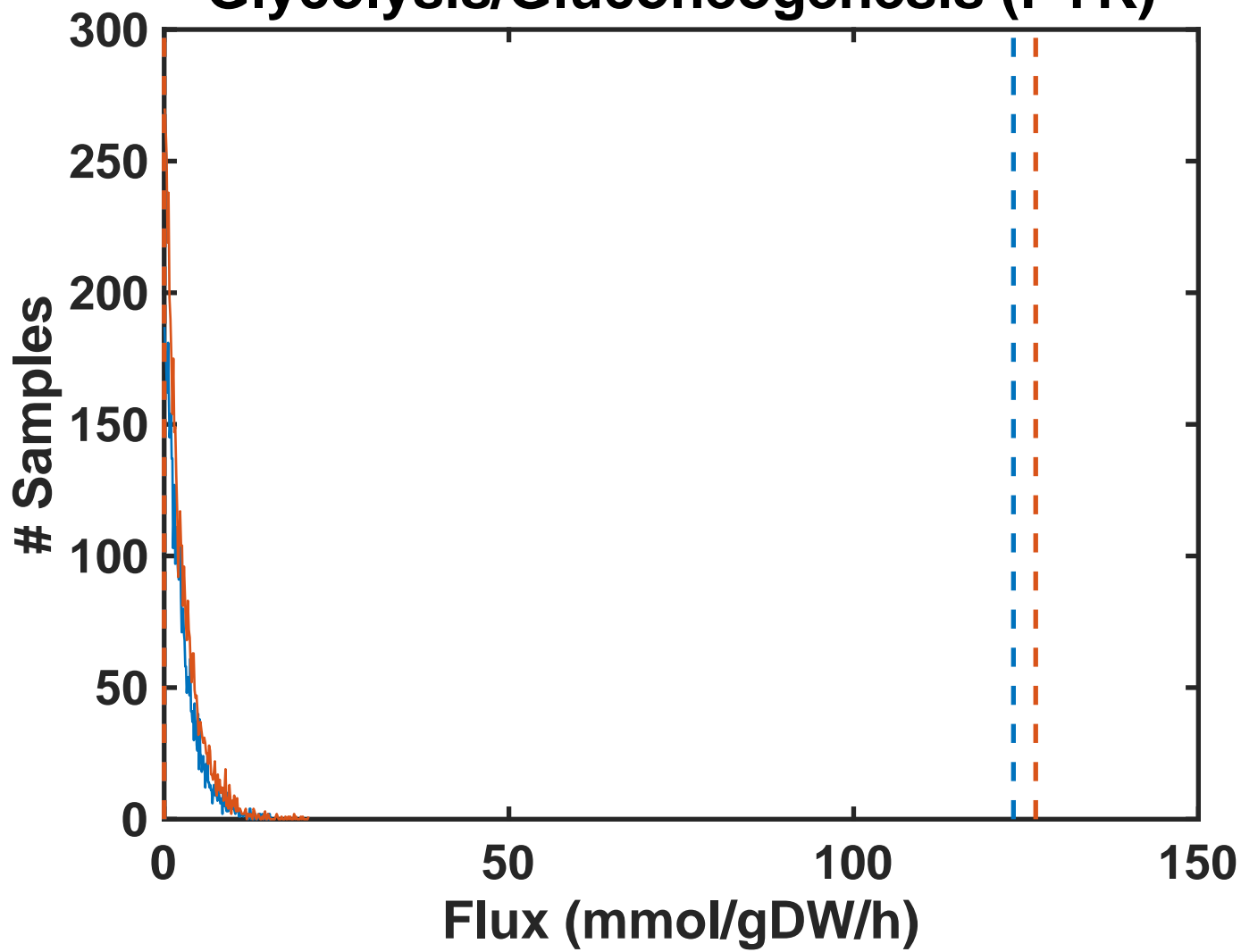

Supplement: Supplementary file 1 [file bioengineering-08-00103-s001.zip › FileS2/figure_sampling-PYK.pdf]

## Pentose Phosphate Pathway (RPE)

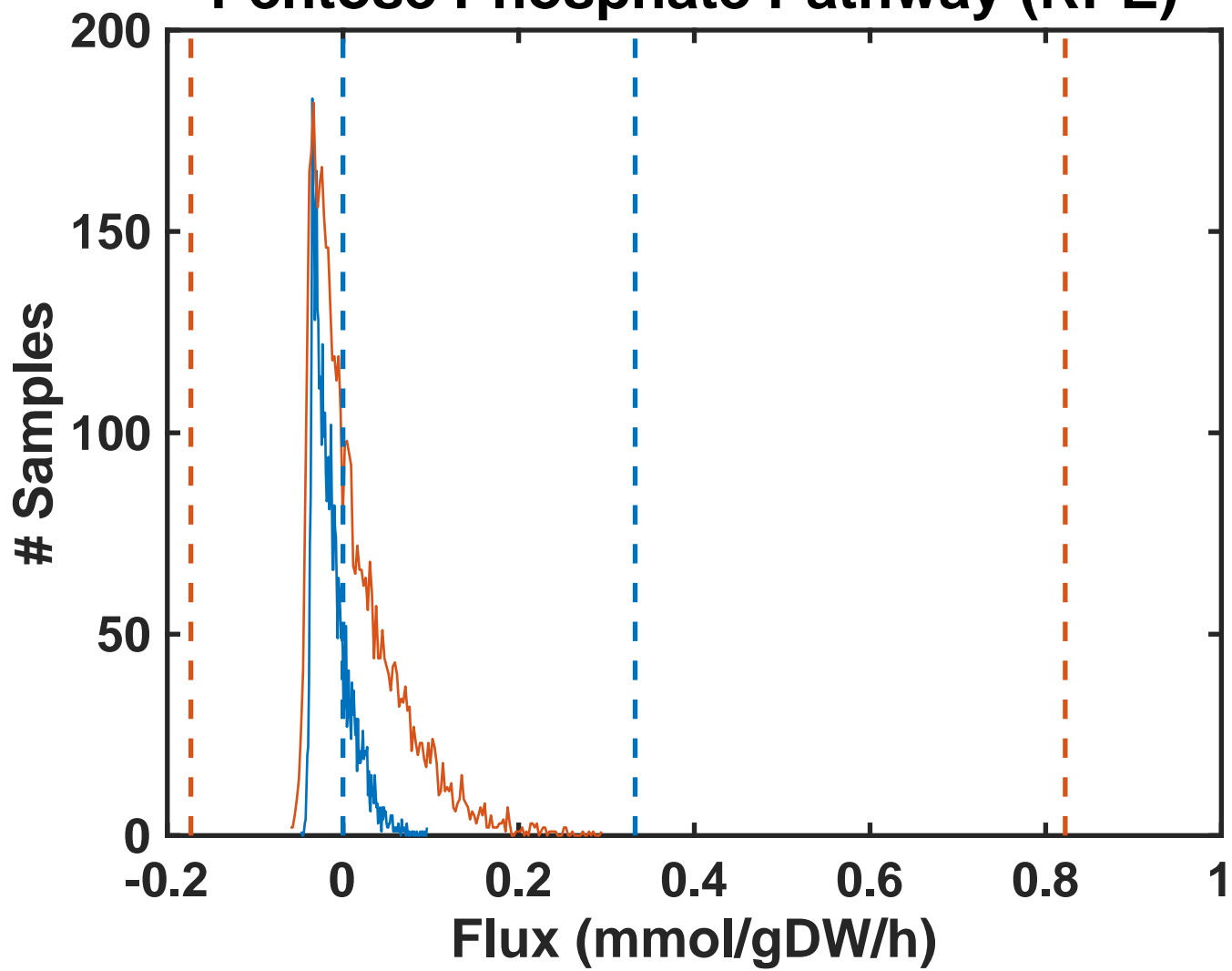

Supplement: Supplementary file 1 [file bioengineering-08-00103-s001.zip › FileS2/figure_sampling-RPE.pdf]

## Pentose Phosphate Pathway (RPI)

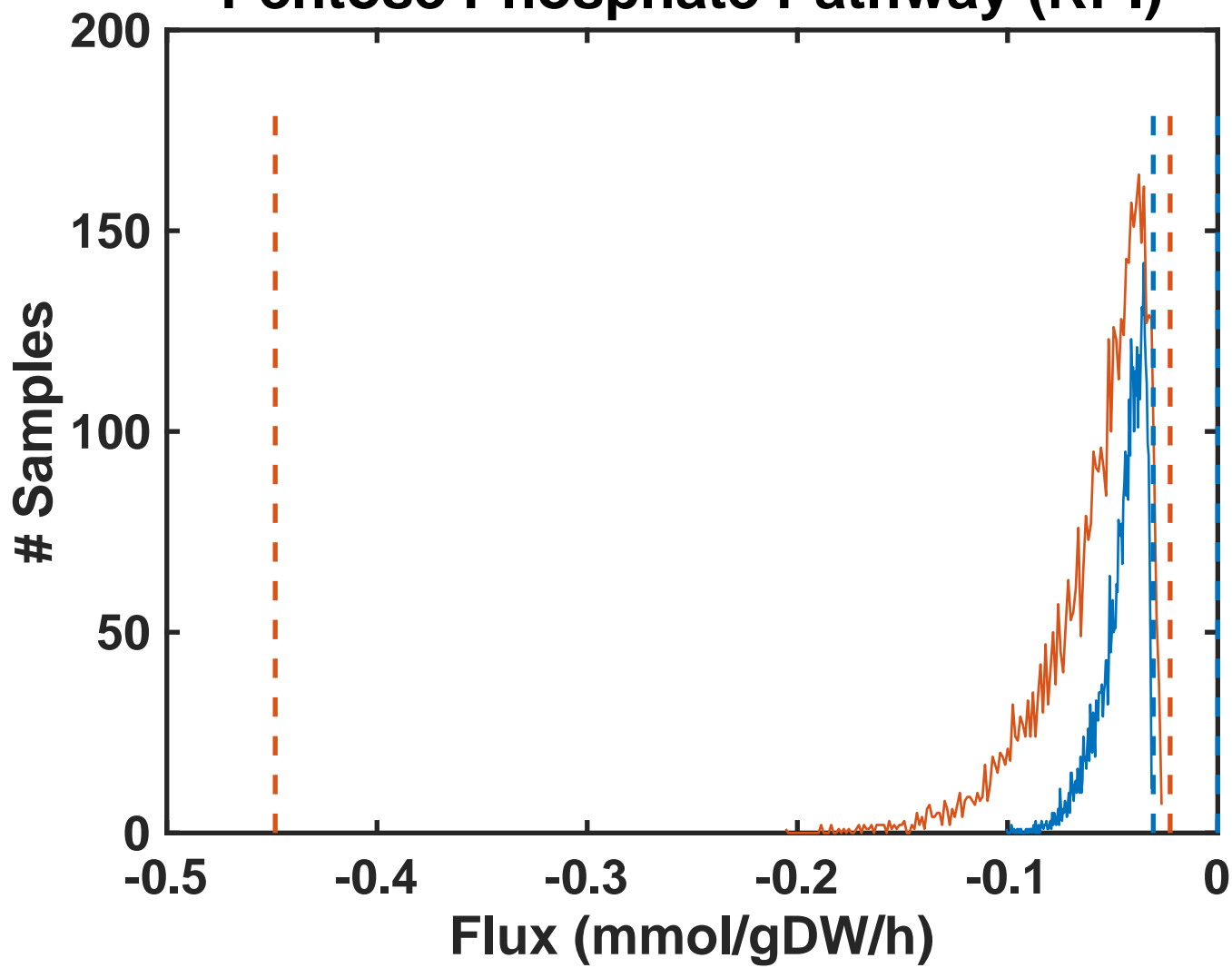

Supplement: Supplementary file 1 [file bioengineering-08-00103-s001.zip › FileS2/figure_sampling-RPI.pdf]

## Citric Acid Cycle (SUCD1)

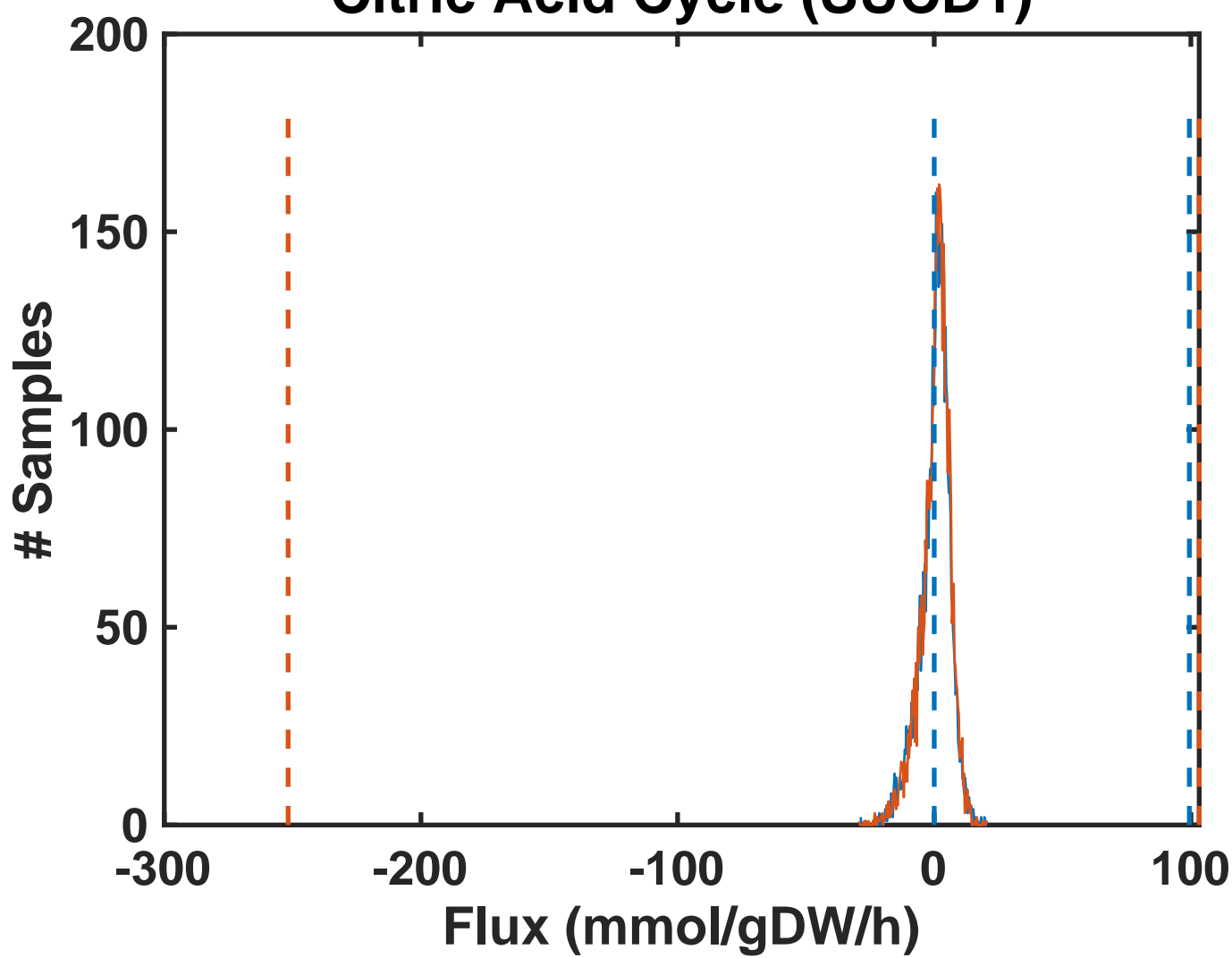

Supplement: Supplementary file 1 [file bioengineering-08-00103-s001.zip › FileS2/figure_sampling-SUCD1.pdf]

# Citric Acid Cycle (SUCOAS)

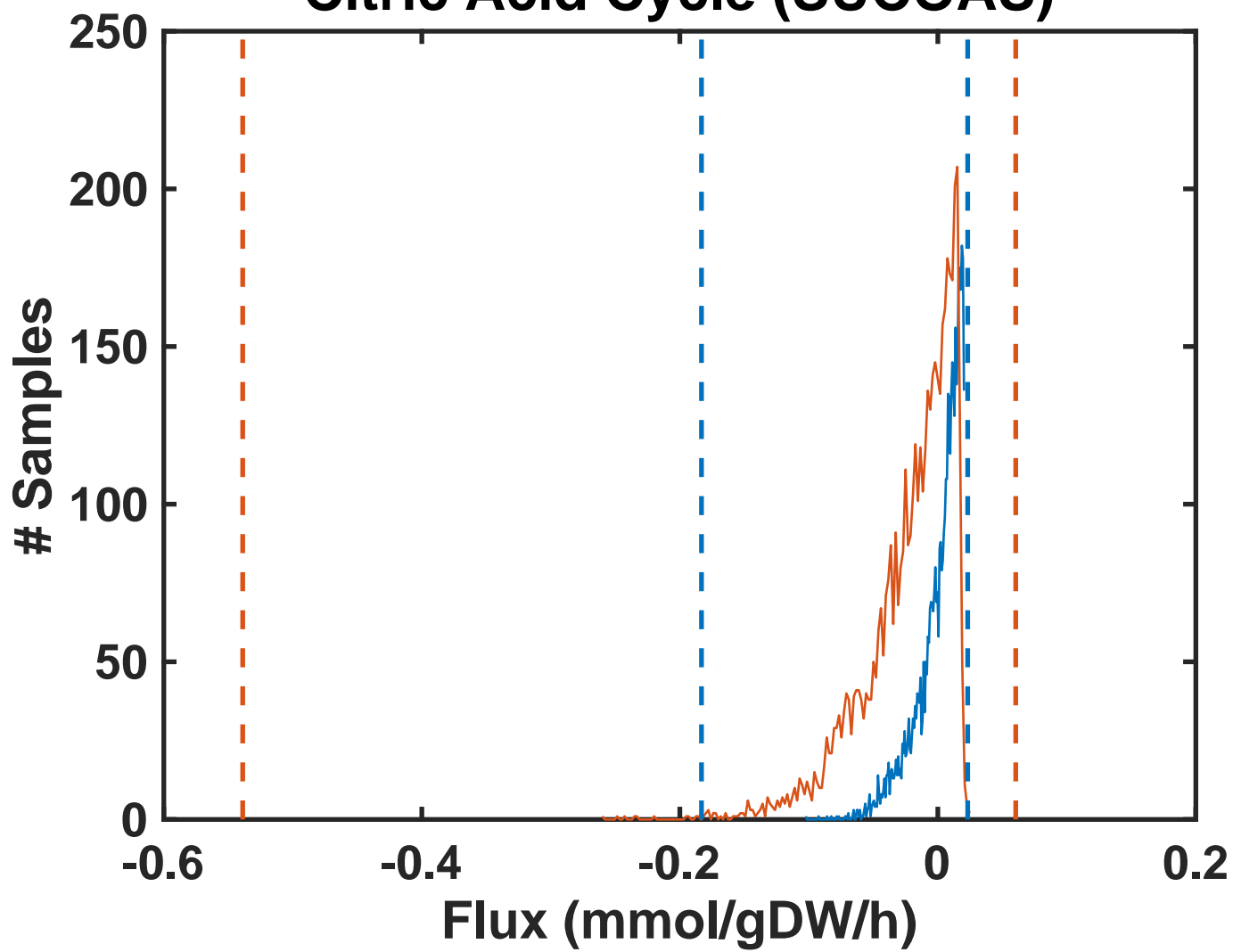

Supplement: Supplementary file 1 [file bioengineering-08-00103-s001.zip › FileS2/figure_sampling-SUCOAS.pdf]

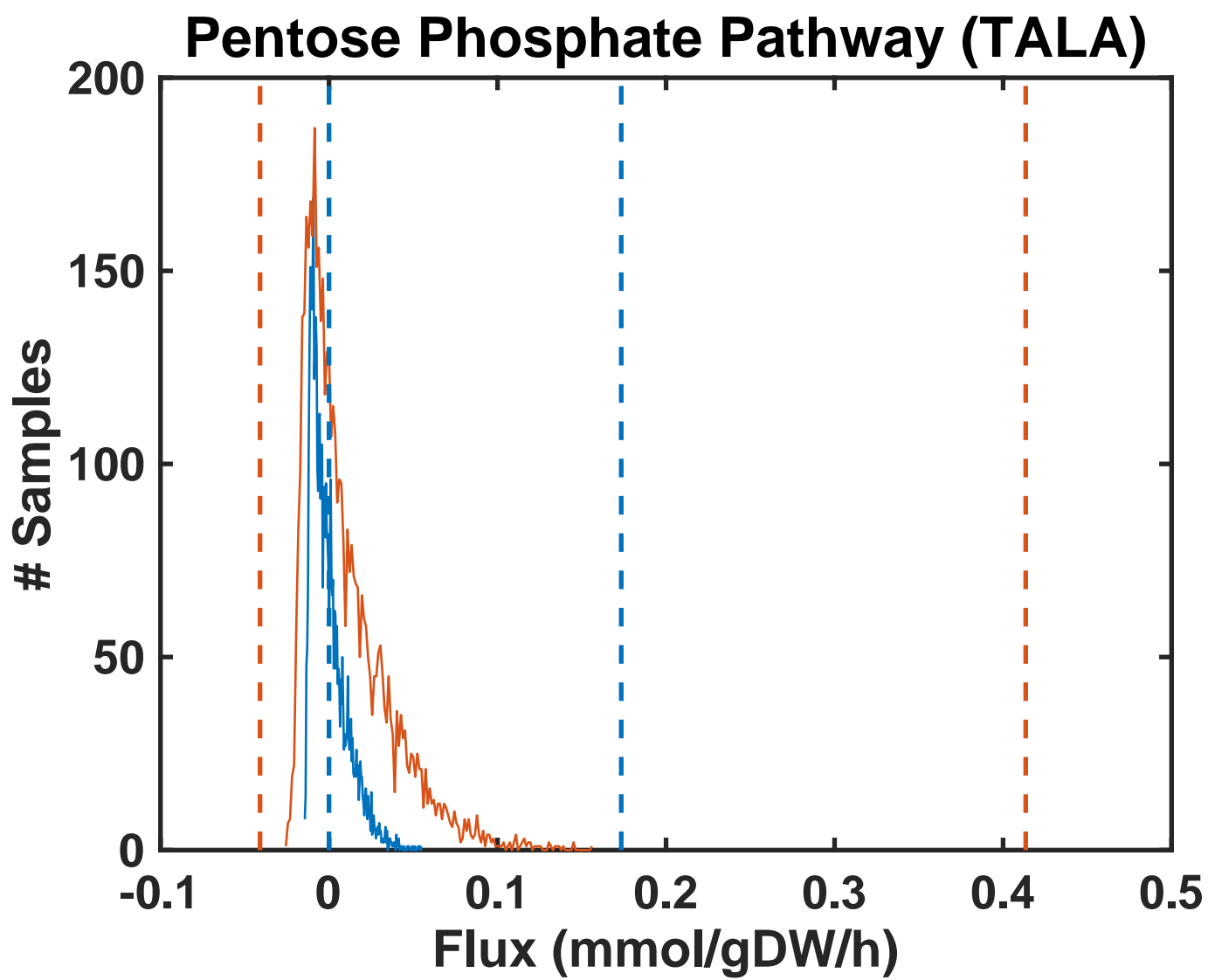

Supplement: Supplementary file 1 [file bioengineering-08-00103-s001.zip › FileS2/figure_sampling-TALA.pdf]

## Pentose Phosphate Pathway (TKT1)

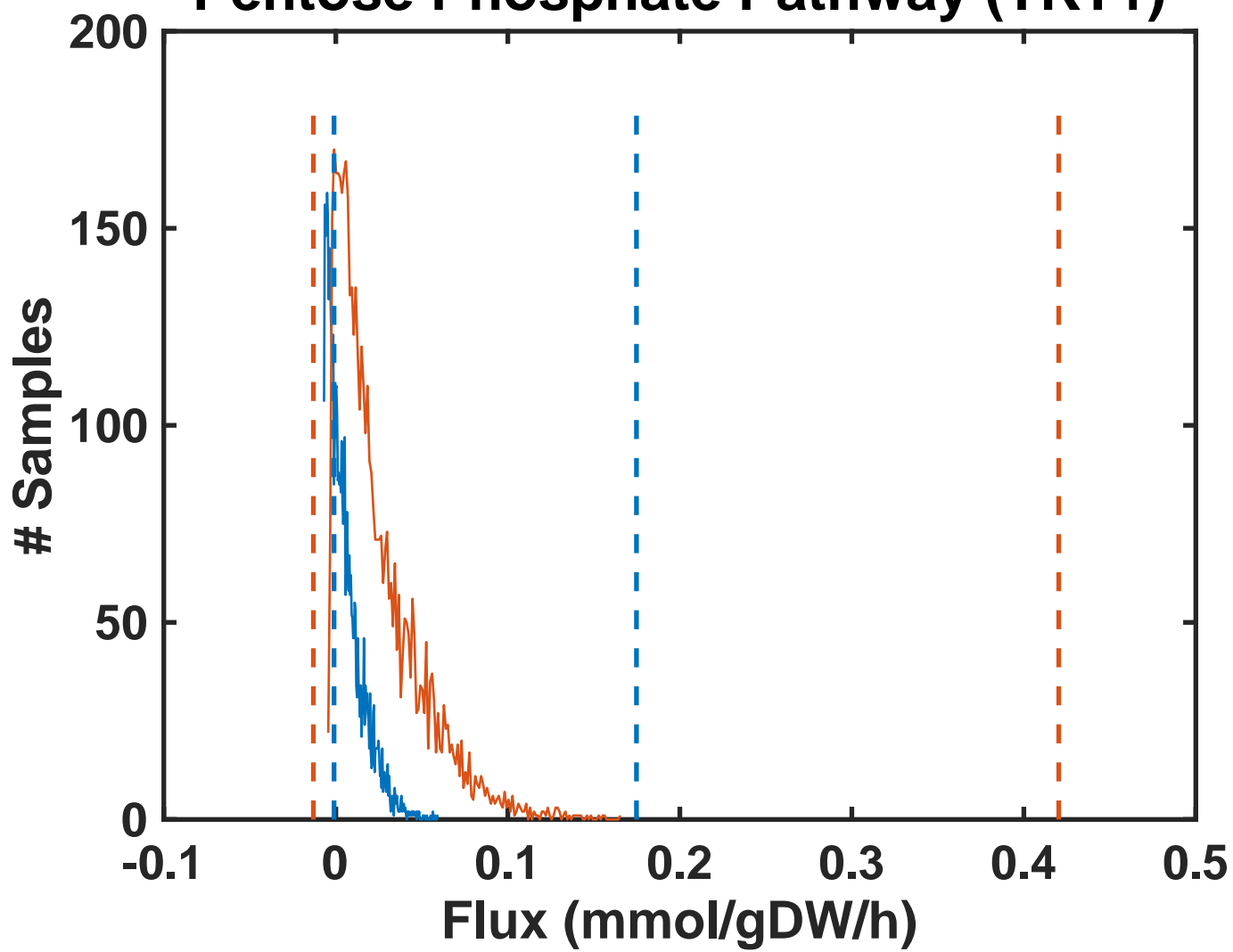

Supplement: Supplementary file 1 [file bioengineering-08-00103-s001.zip › FileS2/figure_sampling-TKT1.pdf]

## Pentose Phosphate Pathway (TKT2)

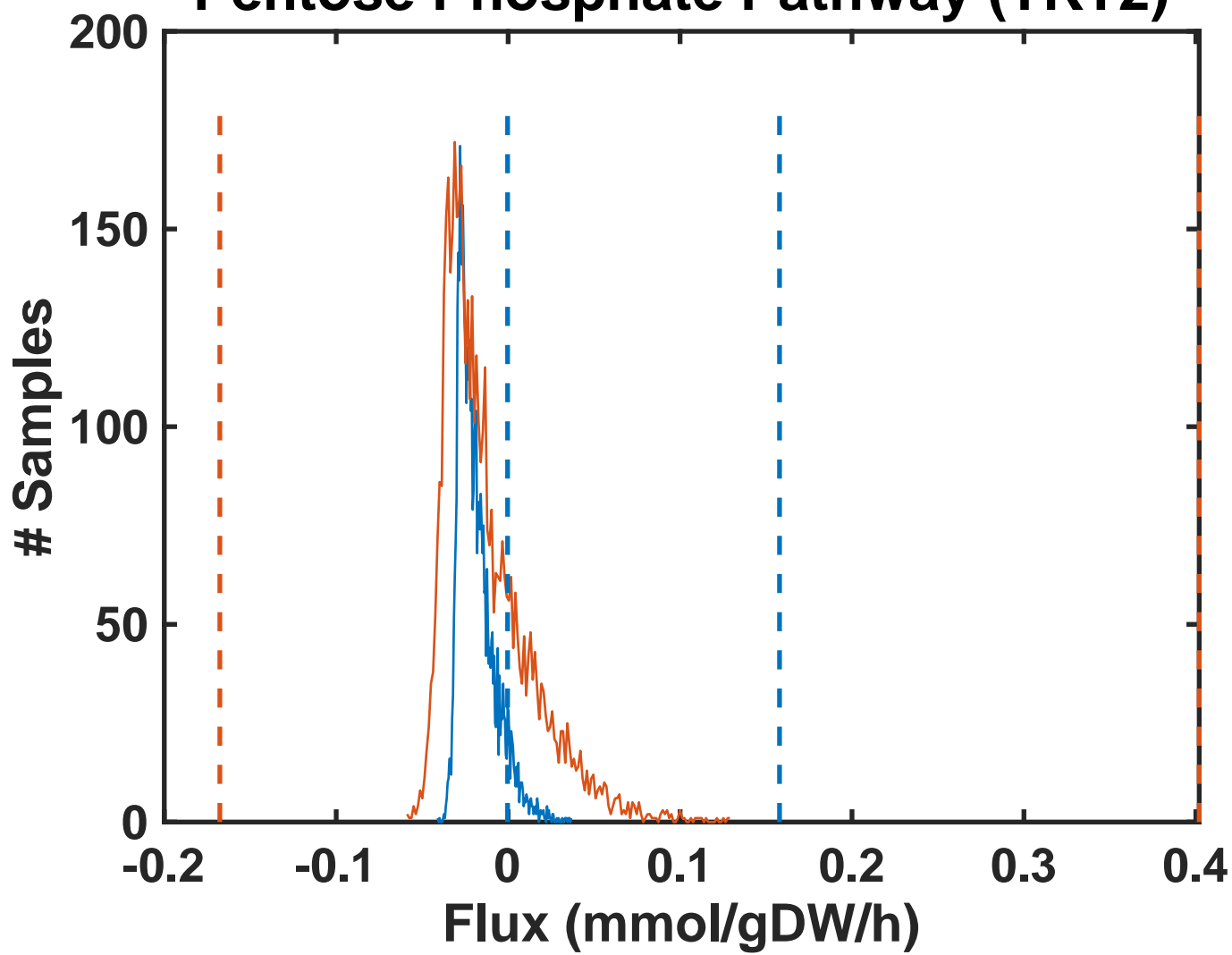

Supplement: Supplementary file 1 [file bioengineering-08-00103-s001.zip › FileS2/figure_sampling-TKT2.pdf]

## Glycolysis/Gluconeogenesis (TPI)

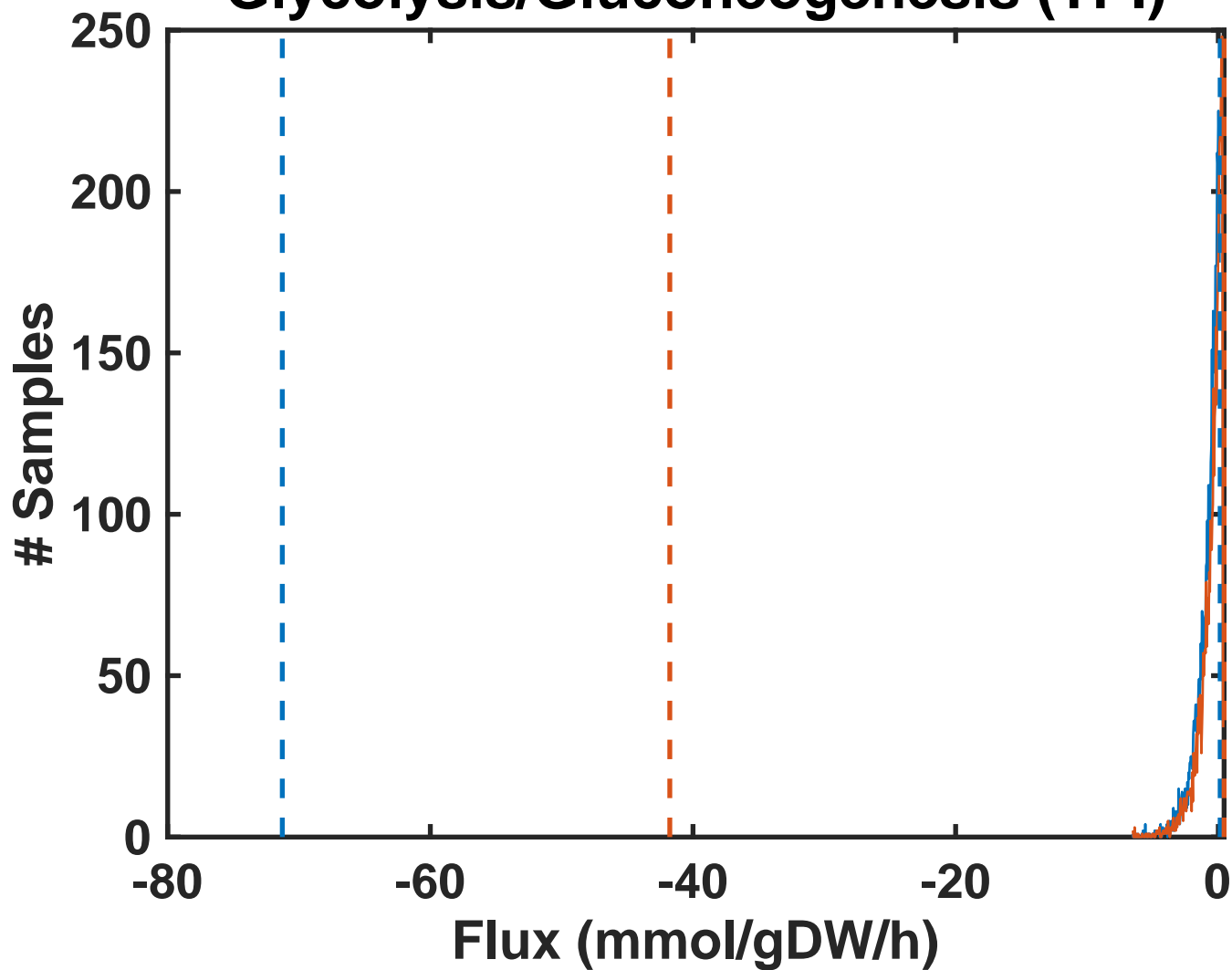

Supplement: Supplementary file 1 [file bioengineering-08-00103-s001.zip › FileS2/figure_sampling-TPI.pdf]

# Pyruvate Metabolism (ACALD)

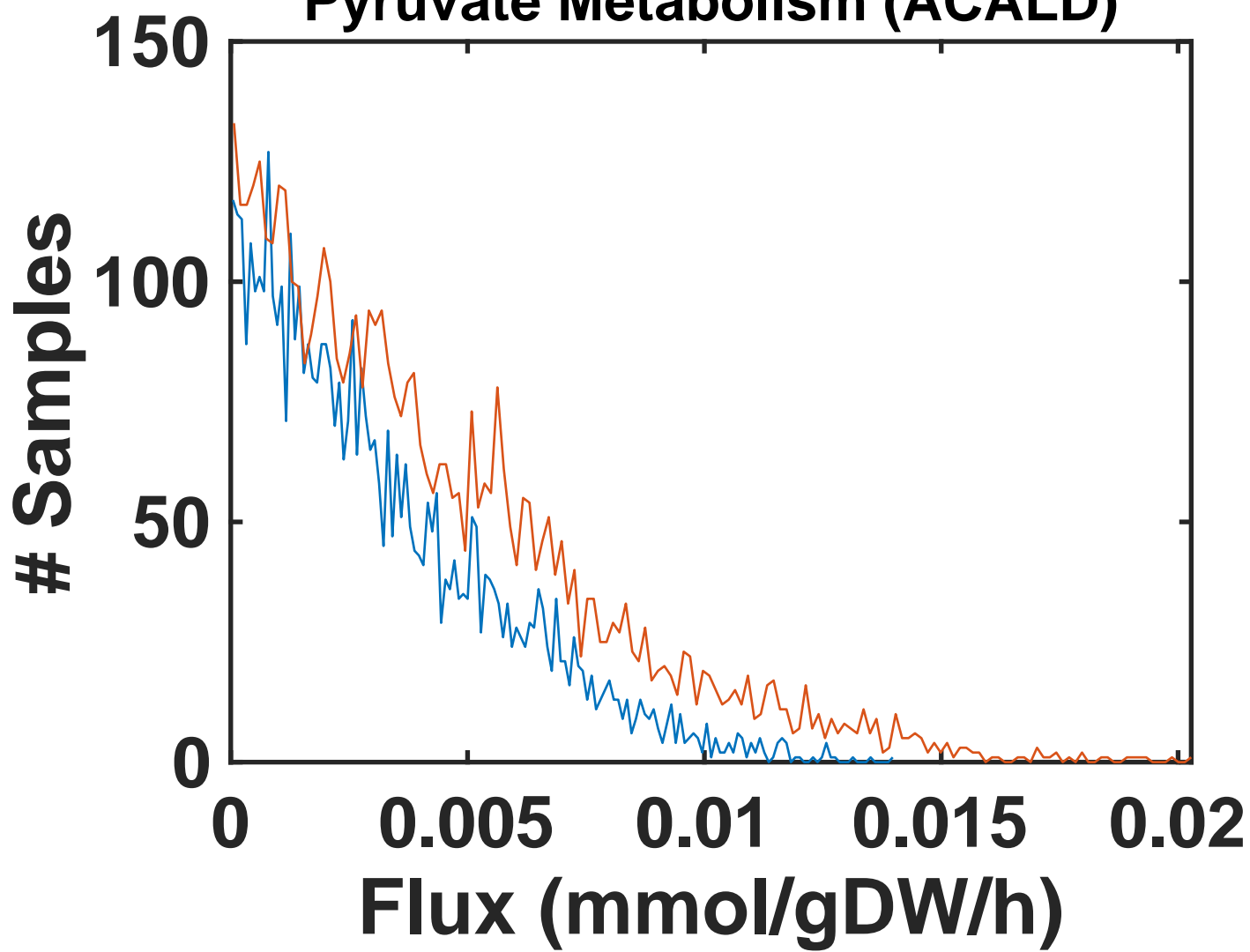

Supplement: Supplementary file 1 [file bioengineering-08-00103-s001.zip › FileS2/figure_sampling_noFVA-ACALD.pdf]

## Pyruvate Metabolism (ACKr)

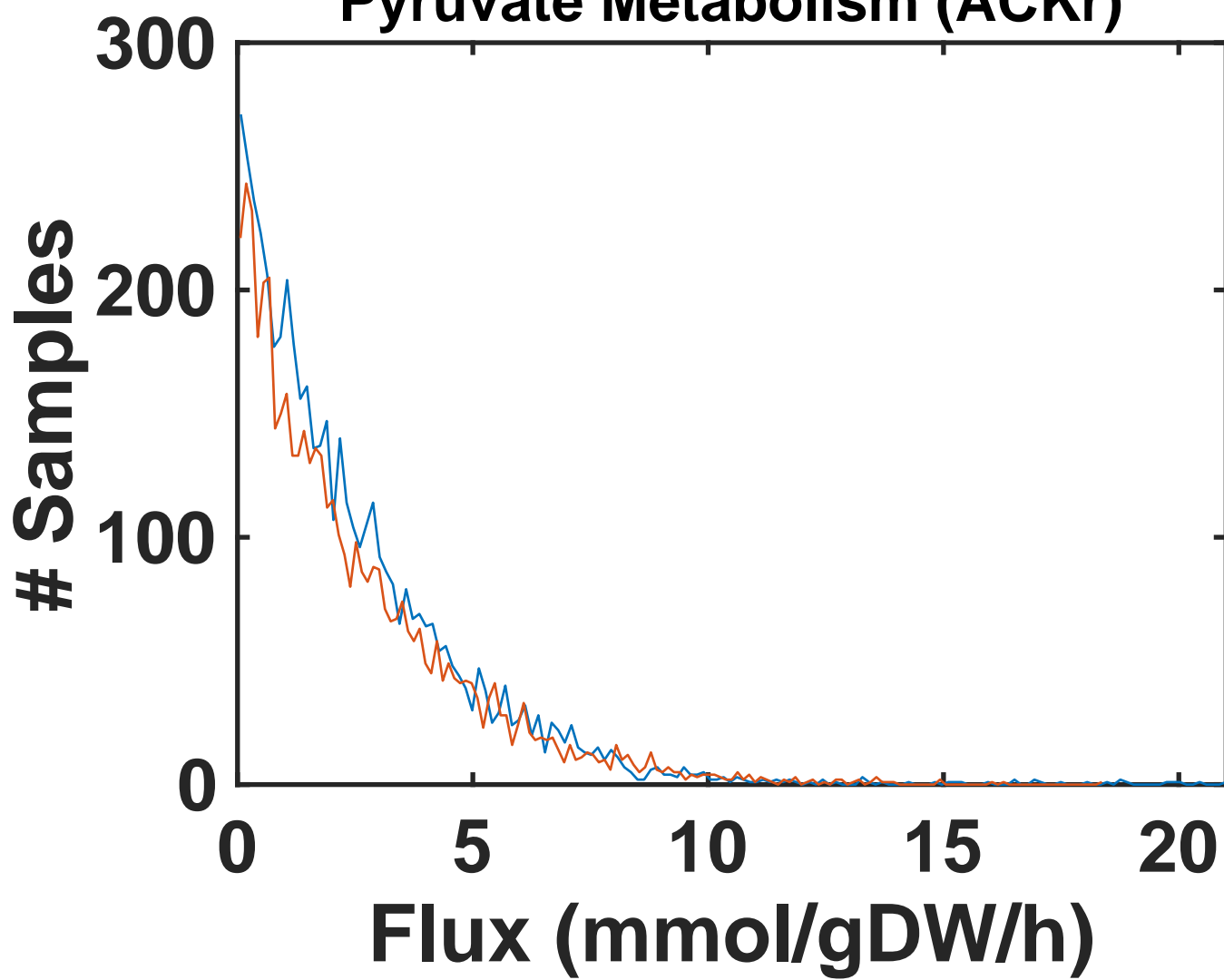

Supplement: Supplementary file 1 [file bioengineering-08-00103-s001.zip › FileS2/figure_sampling_noFVA-ACKr.pdf]

# Citric Acid Cycle (ACONT)

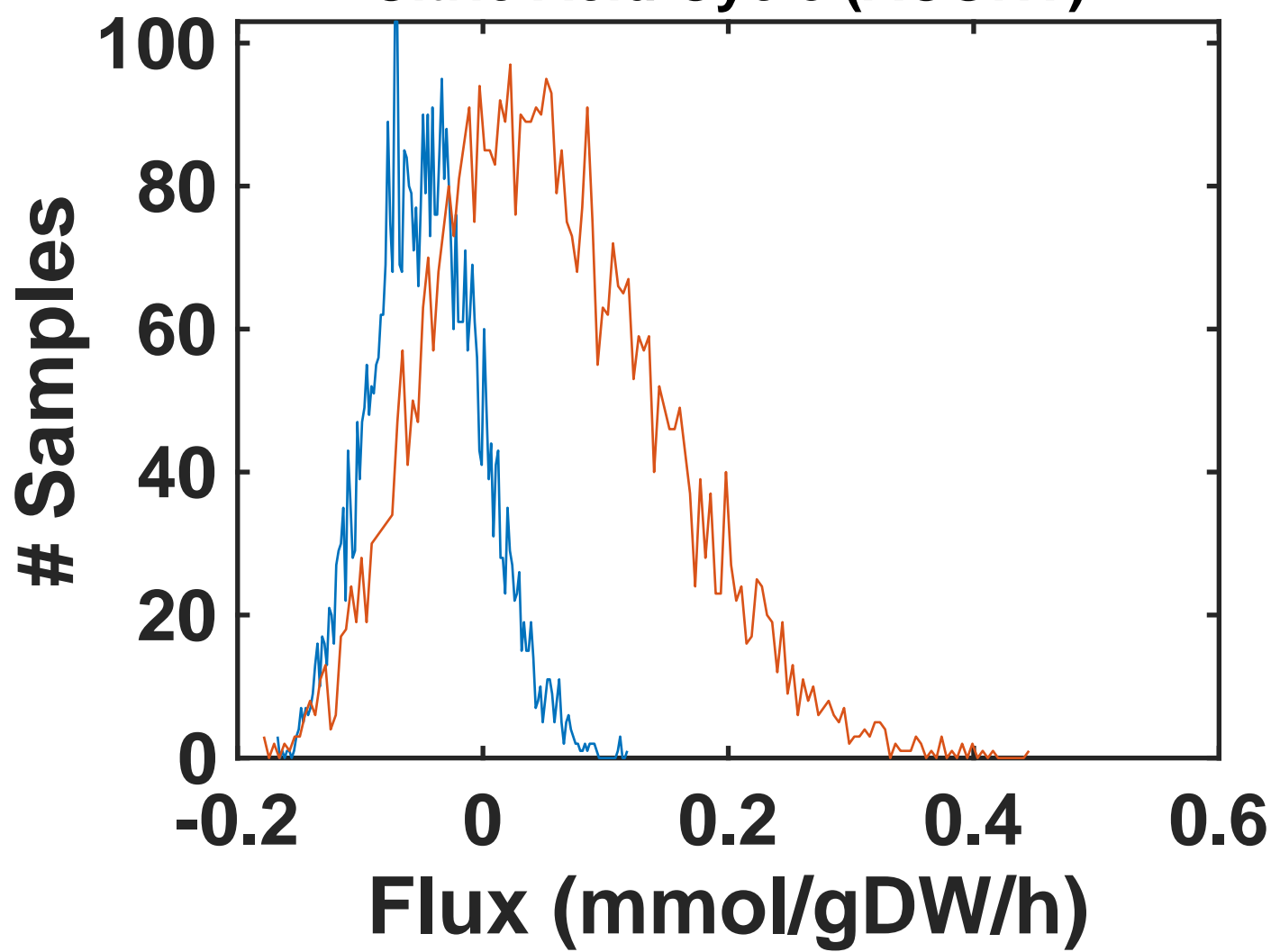

Supplement: Supplementary file 1 [file bioengineering-08-00103-s001.zip › FileS2/figure_sampling_noFVA-ACONT.pdf]

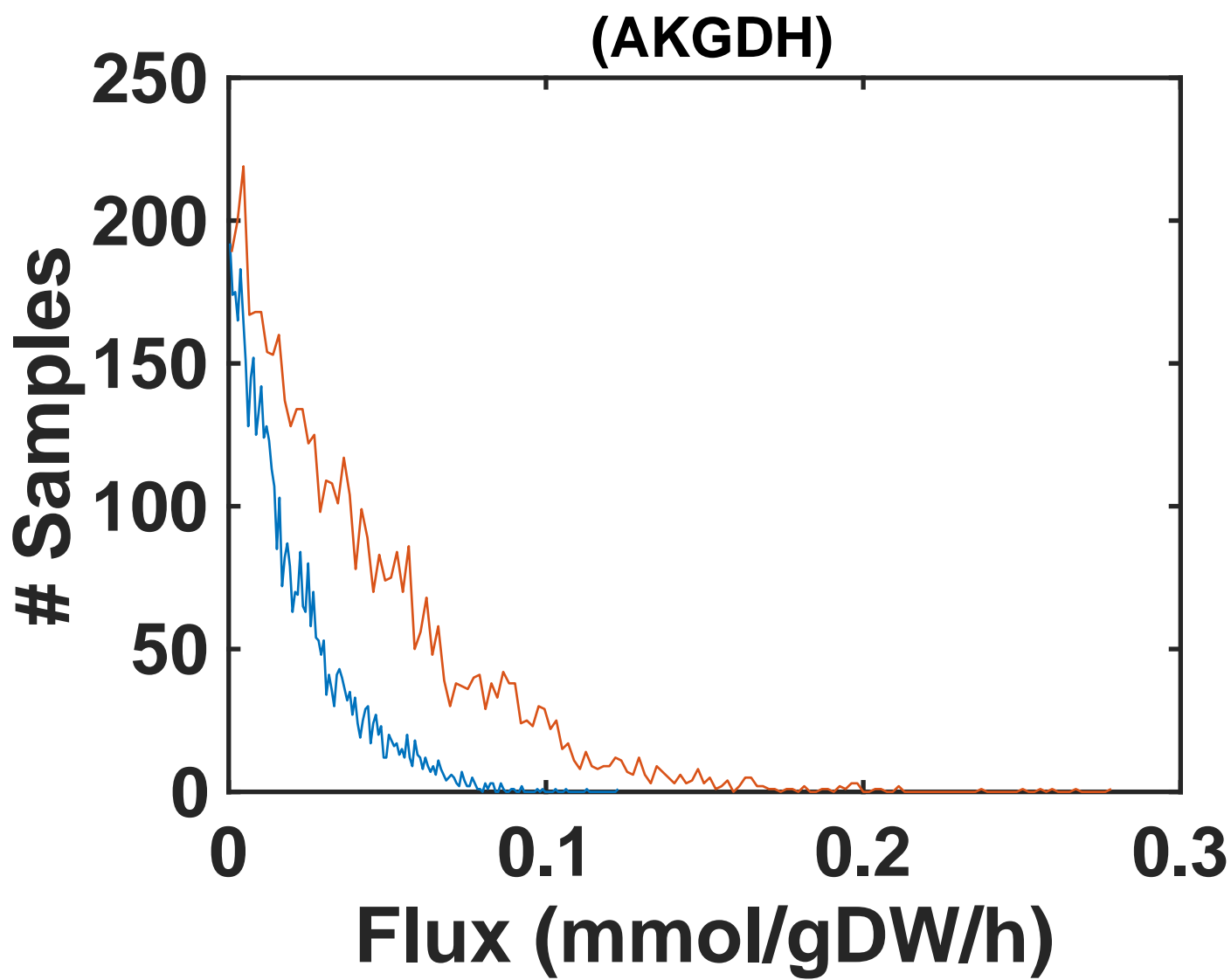

Supplement: Supplementary file 1 [file bioengineering-08-00103-s001.zip › FileS2/figure_sampling_noFVA-AKGDH.pdf]

# Pyruvate Metabolism (ALCD2x)

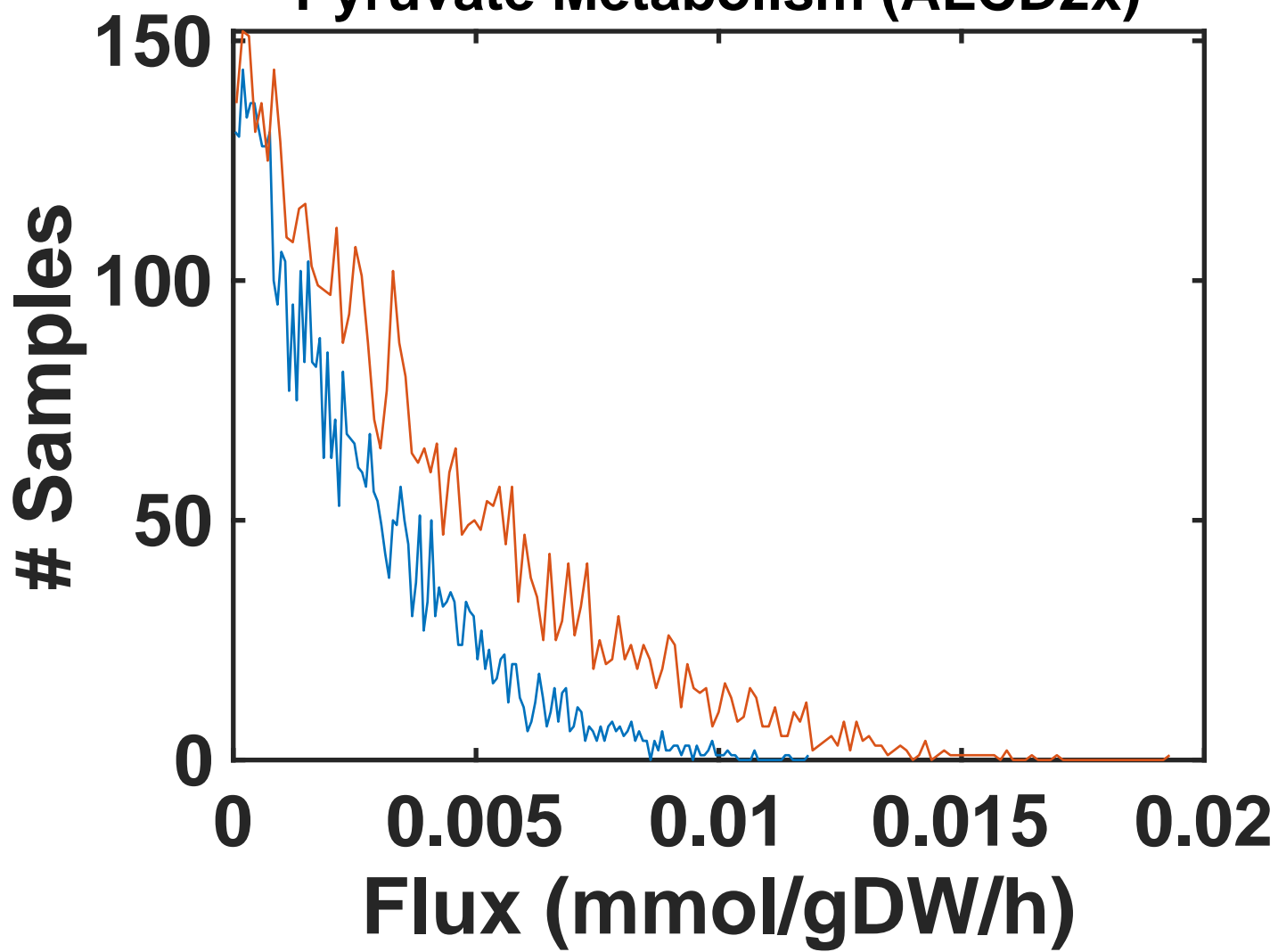

Supplement: Supplementary file 1 [file bioengineering-08-00103-s001.zip › FileS2/figure_sampling_noFVA-ALCD2x.pdf]

## Alanine and Aspartate Metabolism (ASPTA)

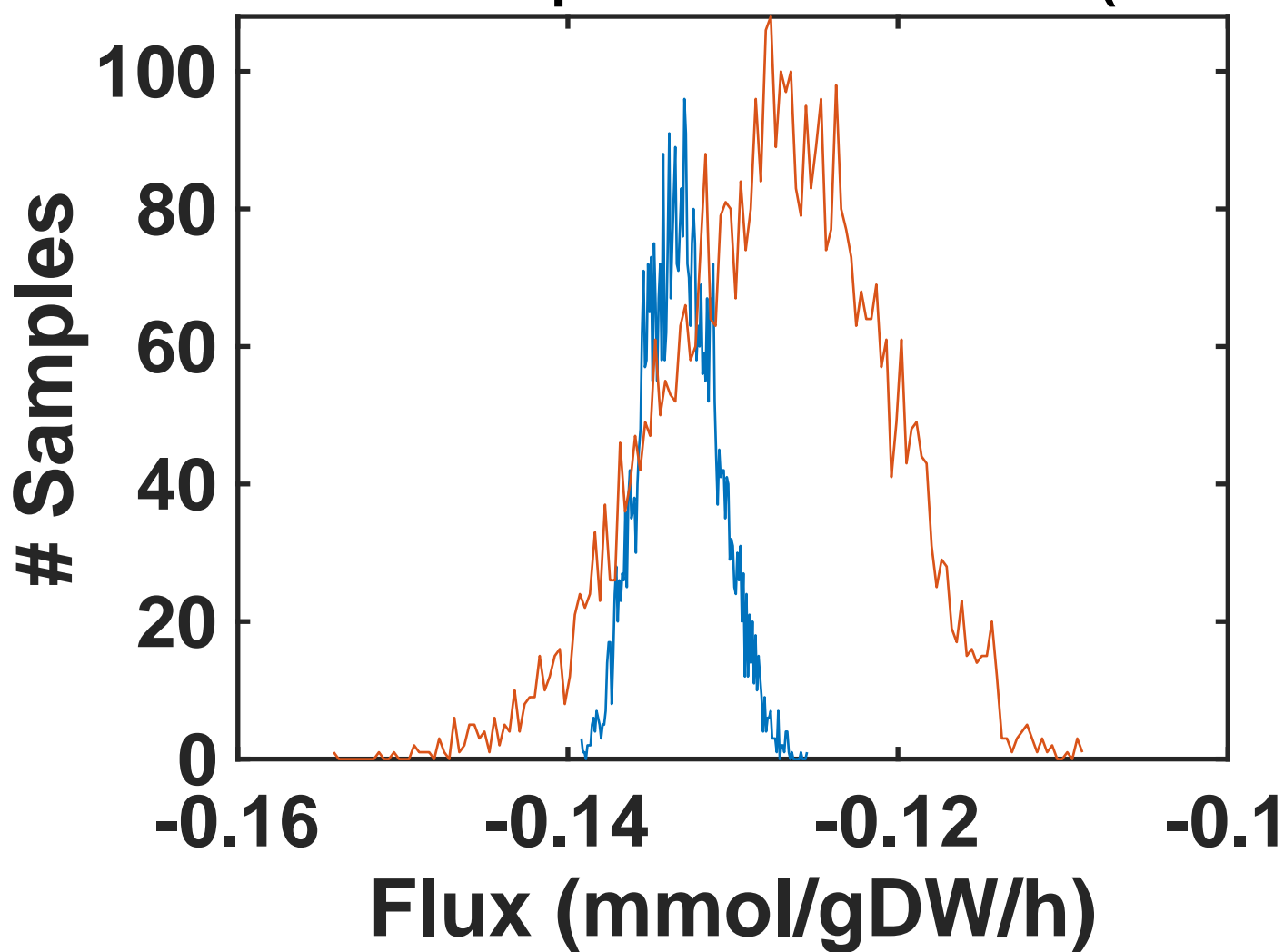

Supplement: Supplementary file 1 [file bioengineering-08-00103-s001.zip › FileS2/figure_sampling_noFVA-ASPTA.pdf]

## Oxidative Phosphorylation (ATPS4rpp)

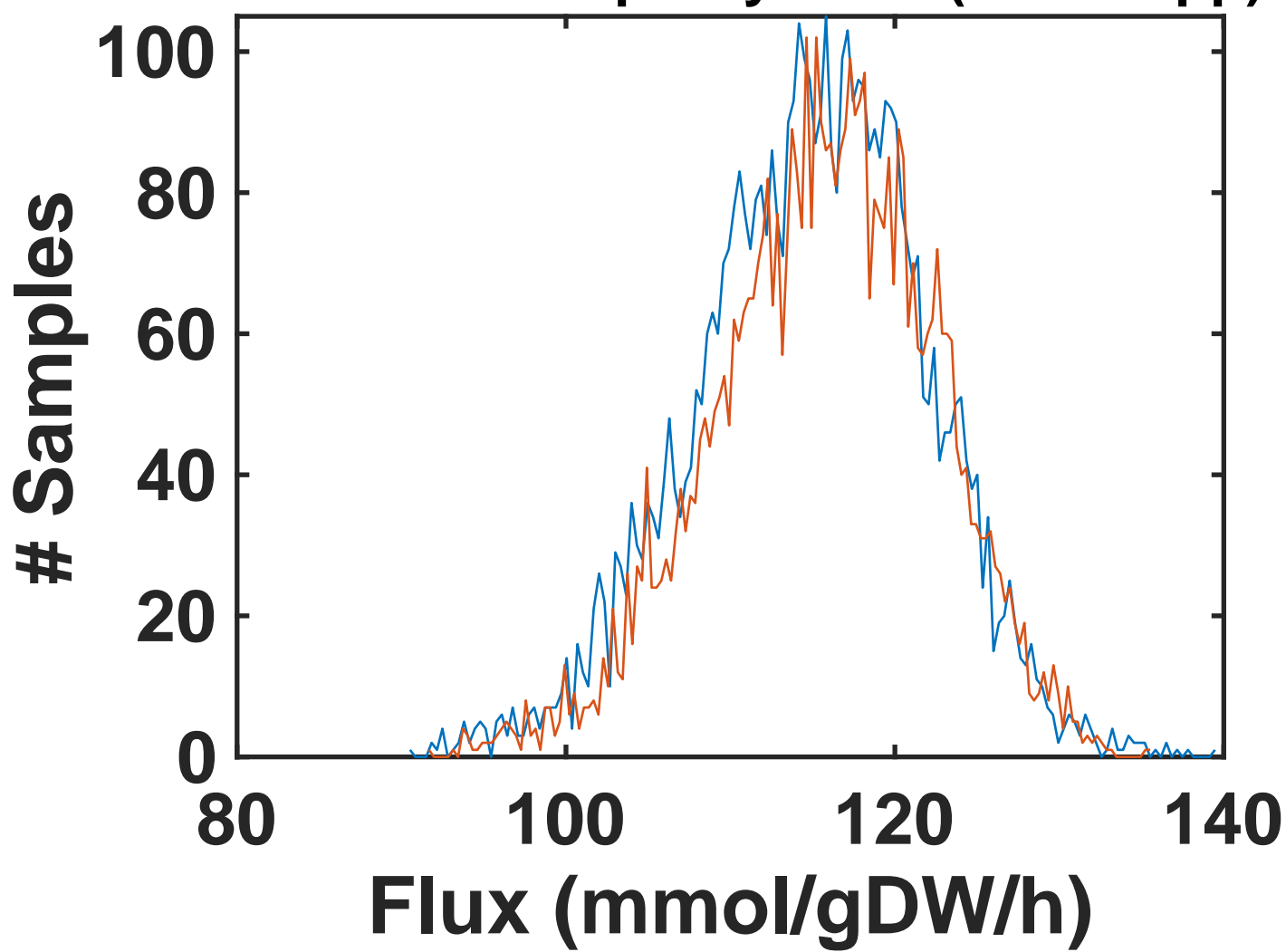

Supplement: Supplementary file 1 [file bioengineering-08-00103-s001.zip › FileS2/figure_sampling_noFVA-ATPS4rpp.pdf]

## Arginine and Proline Metabolism (CBPS)

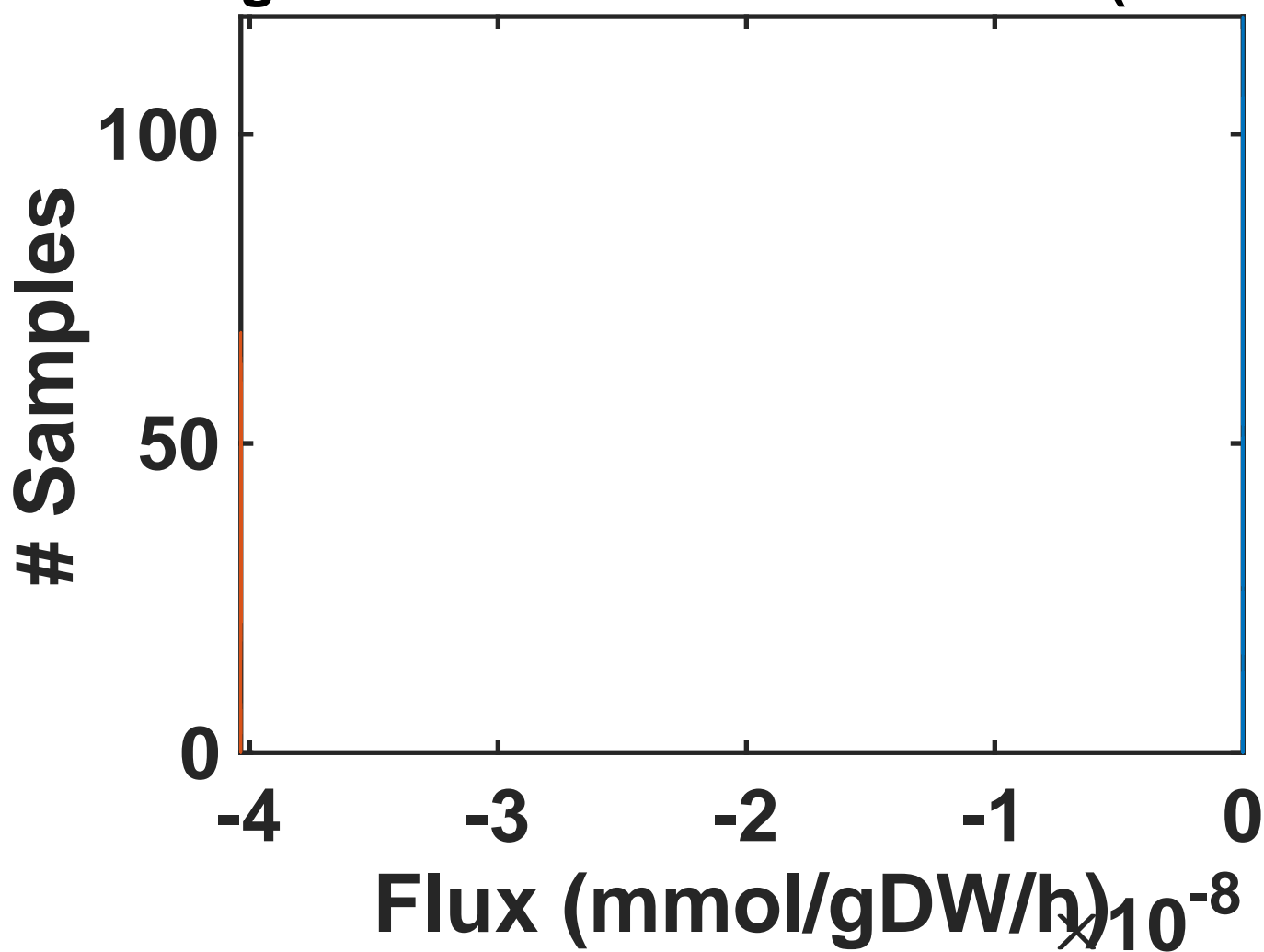

Supplement: Supplementary file 1 [file bioengineering-08-00103-s001.zip › FileS2/figure_sampling_noFVA-CBPS.pdf]

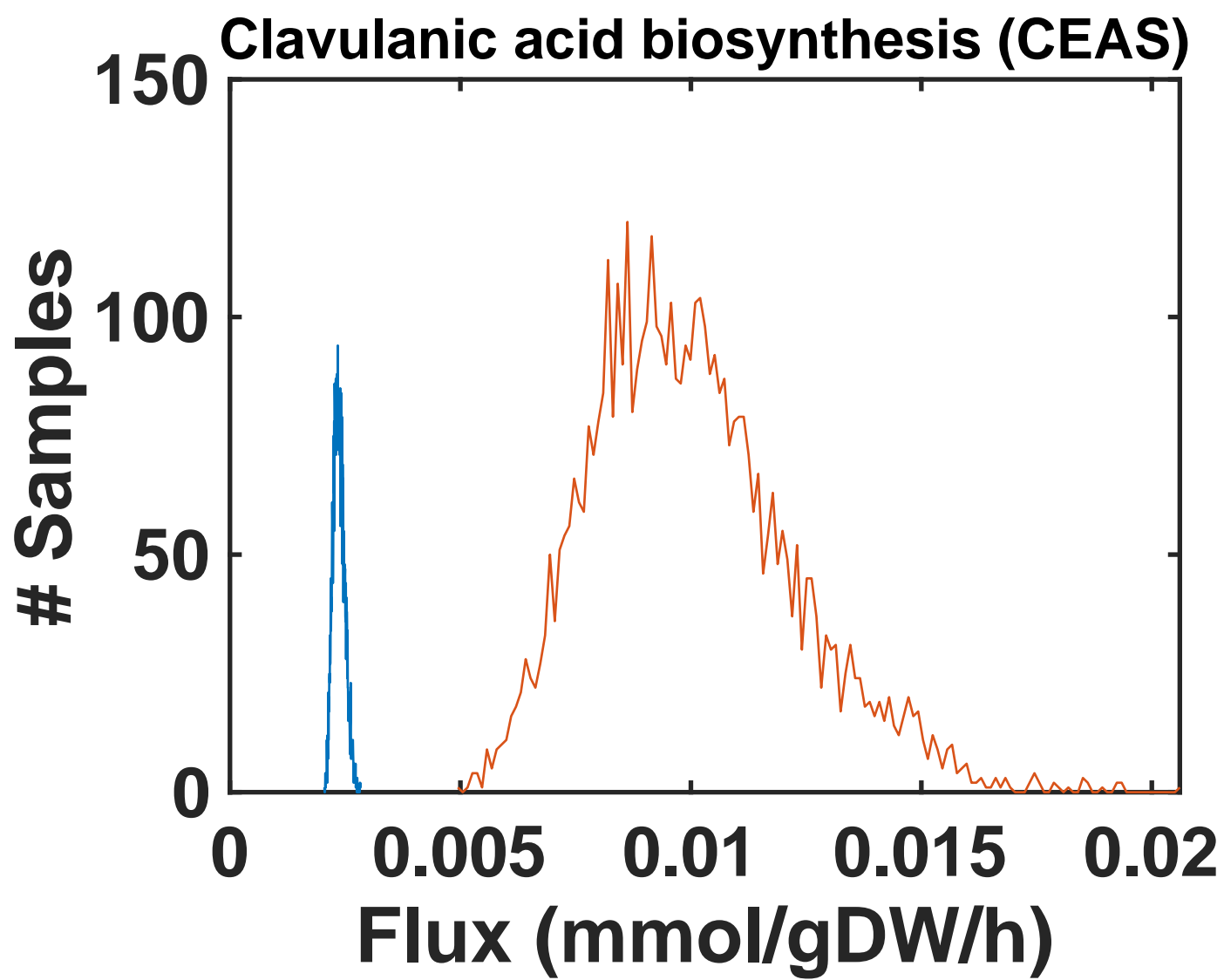

Supplement: Supplementary file 1 [file bioengineering-08-00103-s001.zip › FileS2/figure_sampling_noFVA-CEAS.pdf]

# Citric Acid Cycle (CITL)

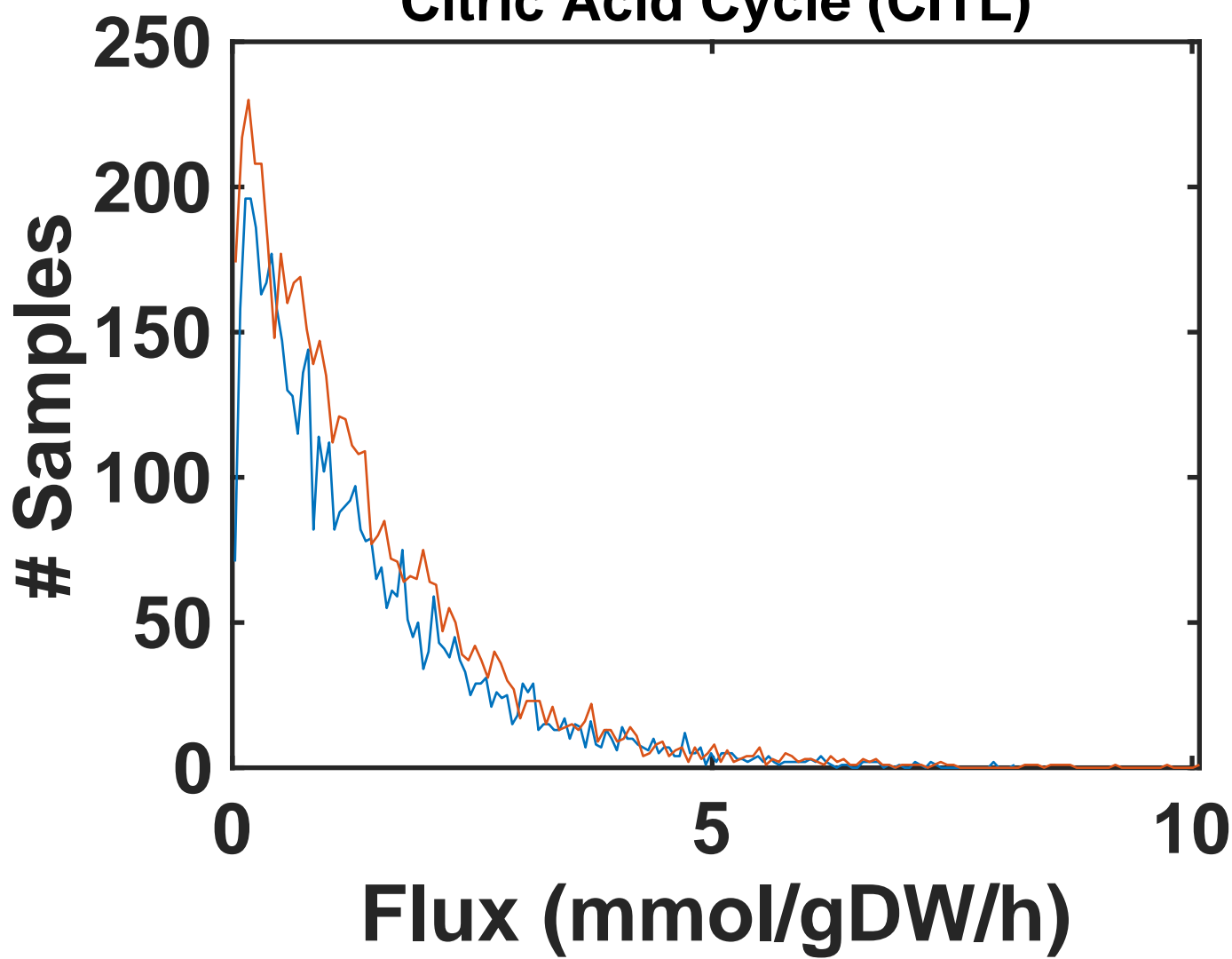

Supplement: Supplementary file 1 [file bioengineering-08-00103-s001.zip › FileS2/figure_sampling_noFVA-CITL.pdf]

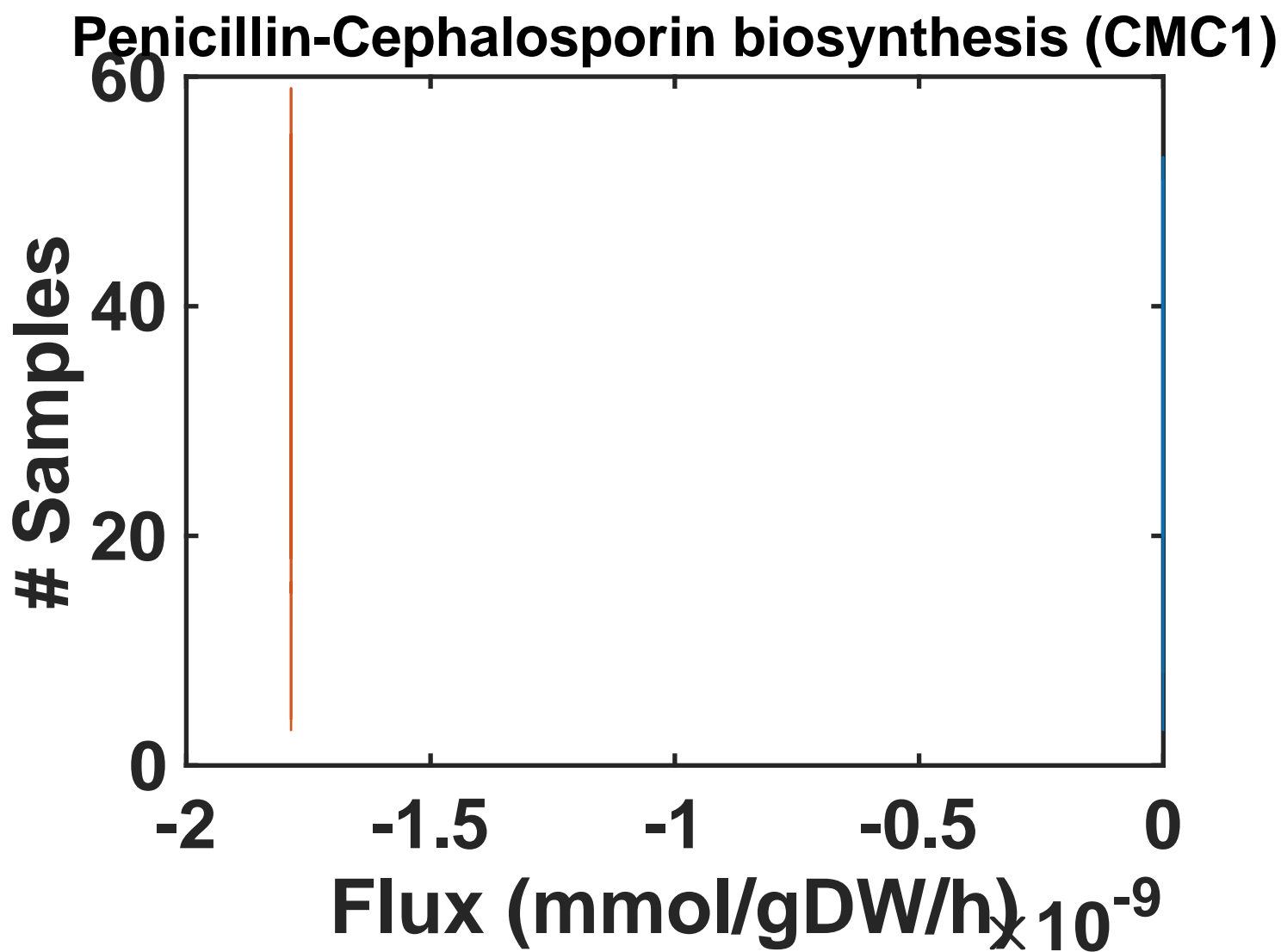

Supplement: Supplementary file 1 [file bioengineering-08-00103-s001.zip › FileS2/figure_sampling_noFVA-CMC1.pdf]

# Citric Acid Cycle (CS)

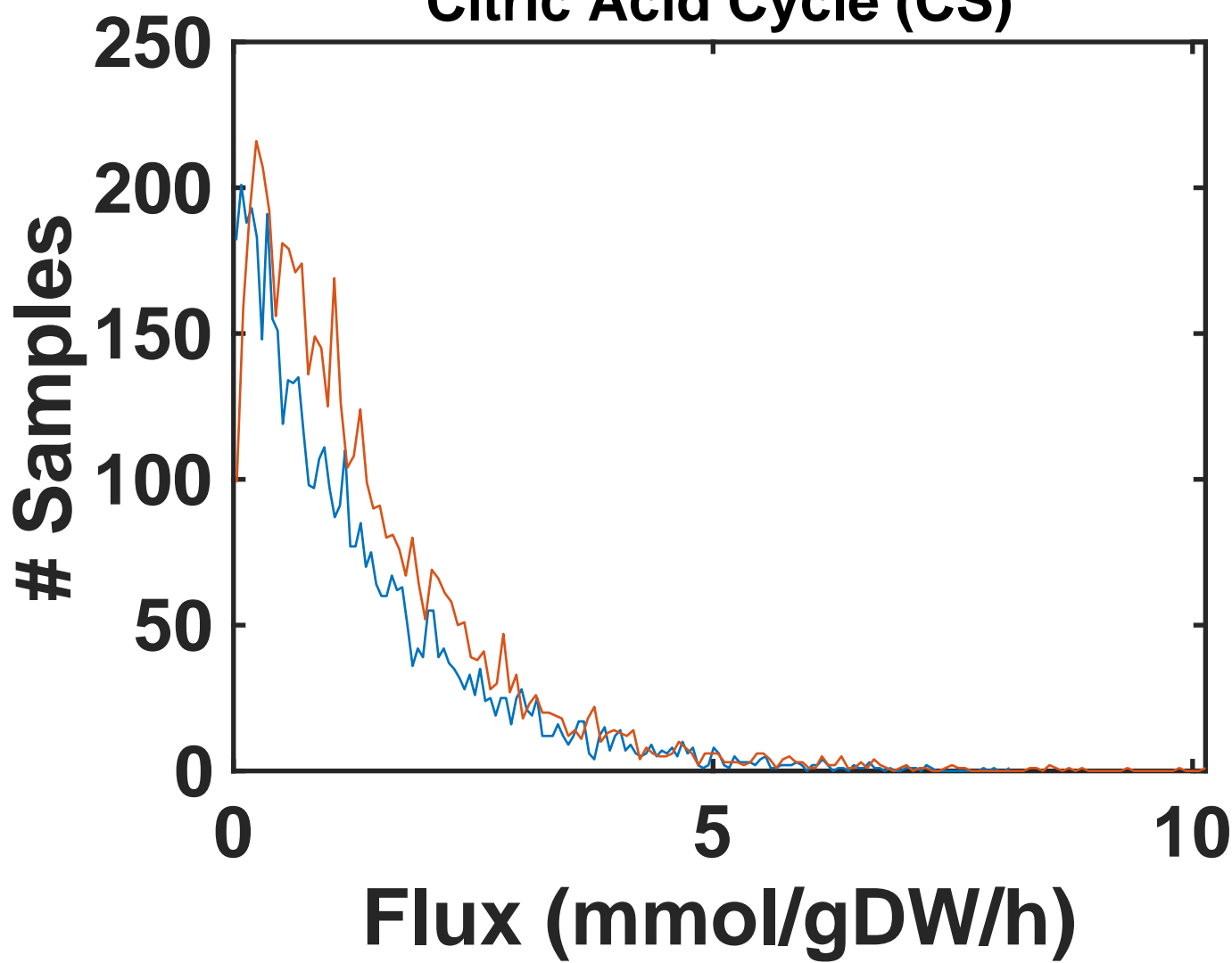

Supplement: Supplementary file 1 [file bioengineering-08-00103-s001.zip › FileS2/figure_sampling_noFVA-CS.pdf]

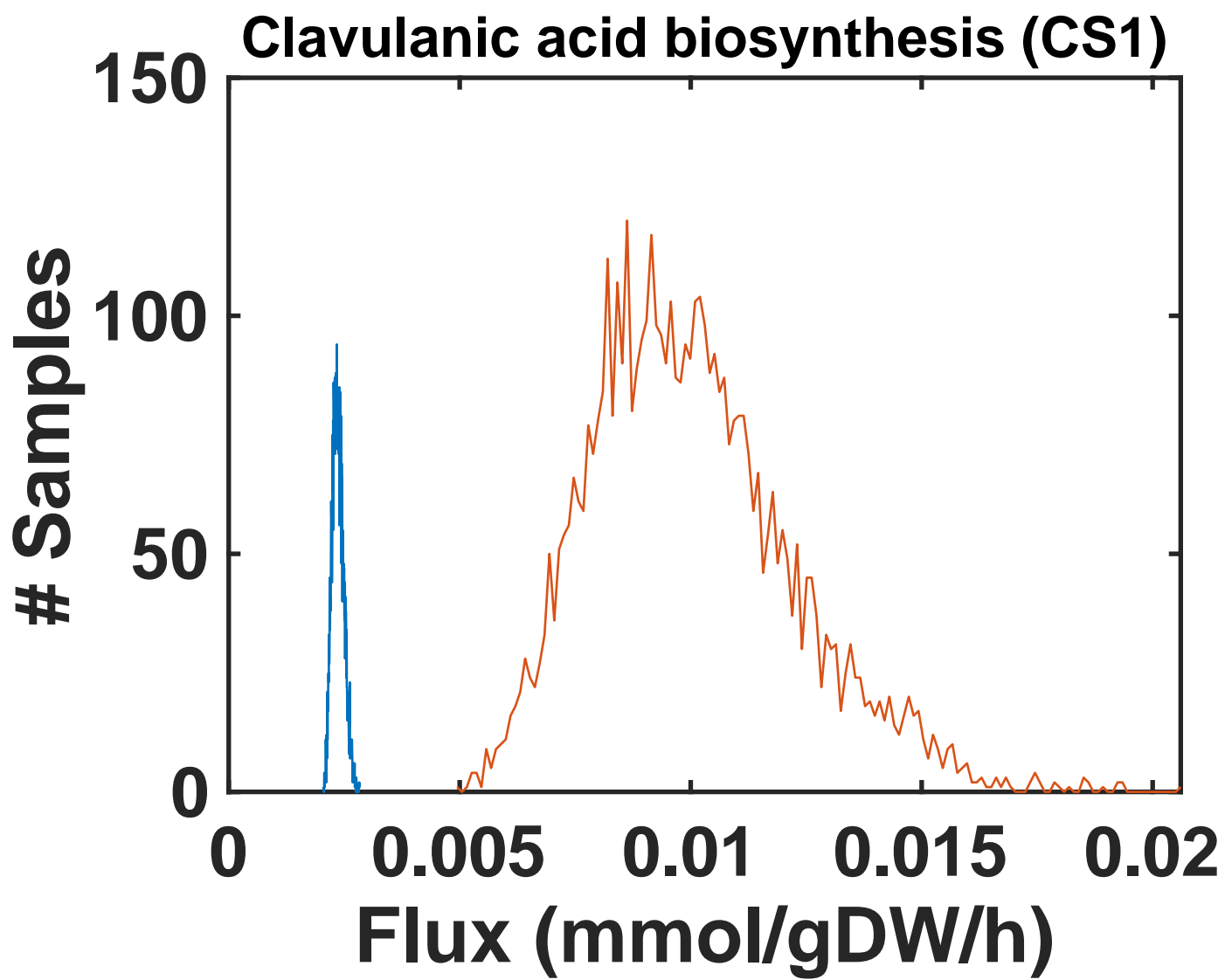

Supplement: Supplementary file 1 [file bioengineering-08-00103-s001.zip › FileS2/figure_sampling_noFVA-CS1.pdf]

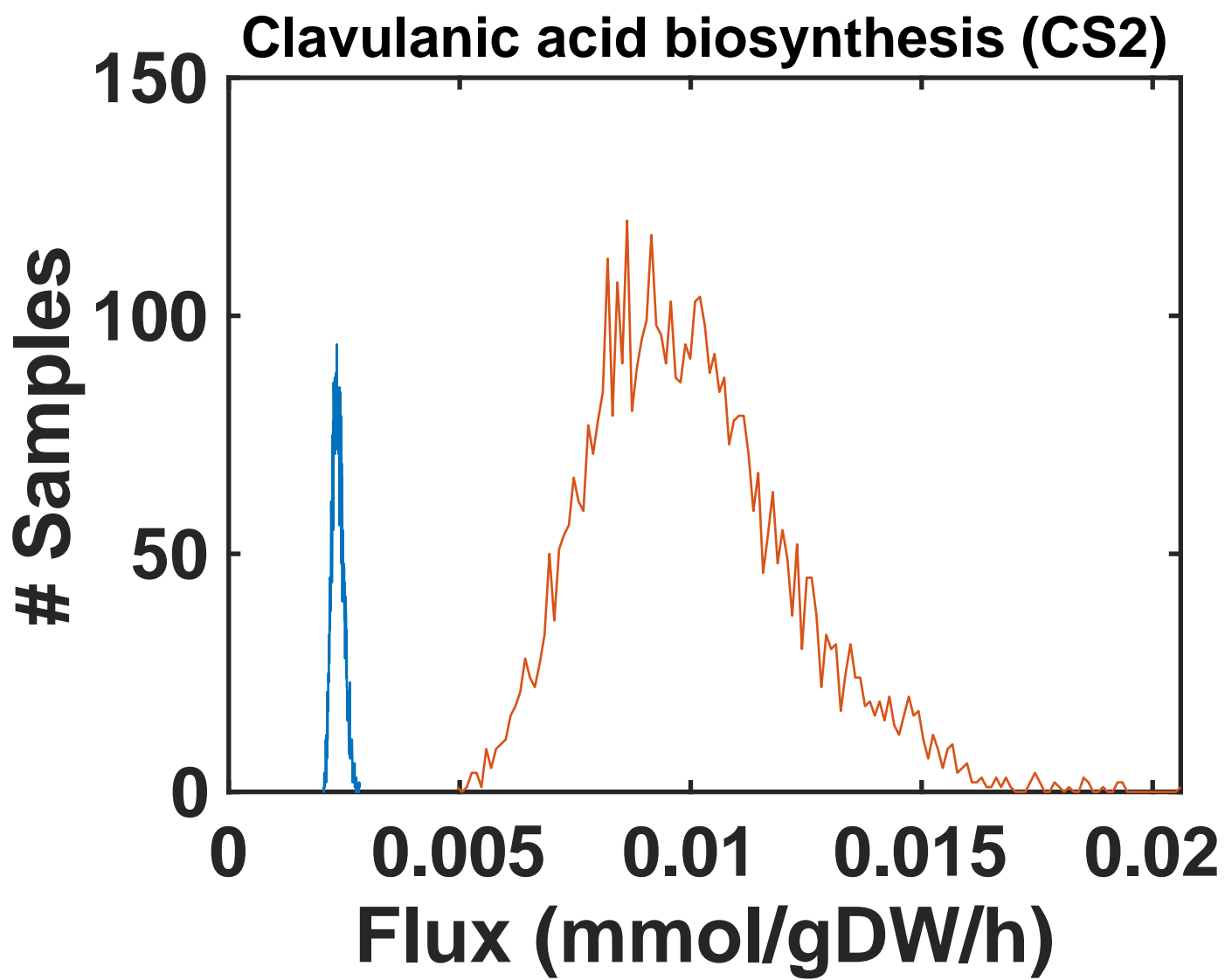

Supplement: Supplementary file 1 [file bioengineering-08-00103-s001.zip › FileS2/figure_sampling_noFVA-CS2.pdf]

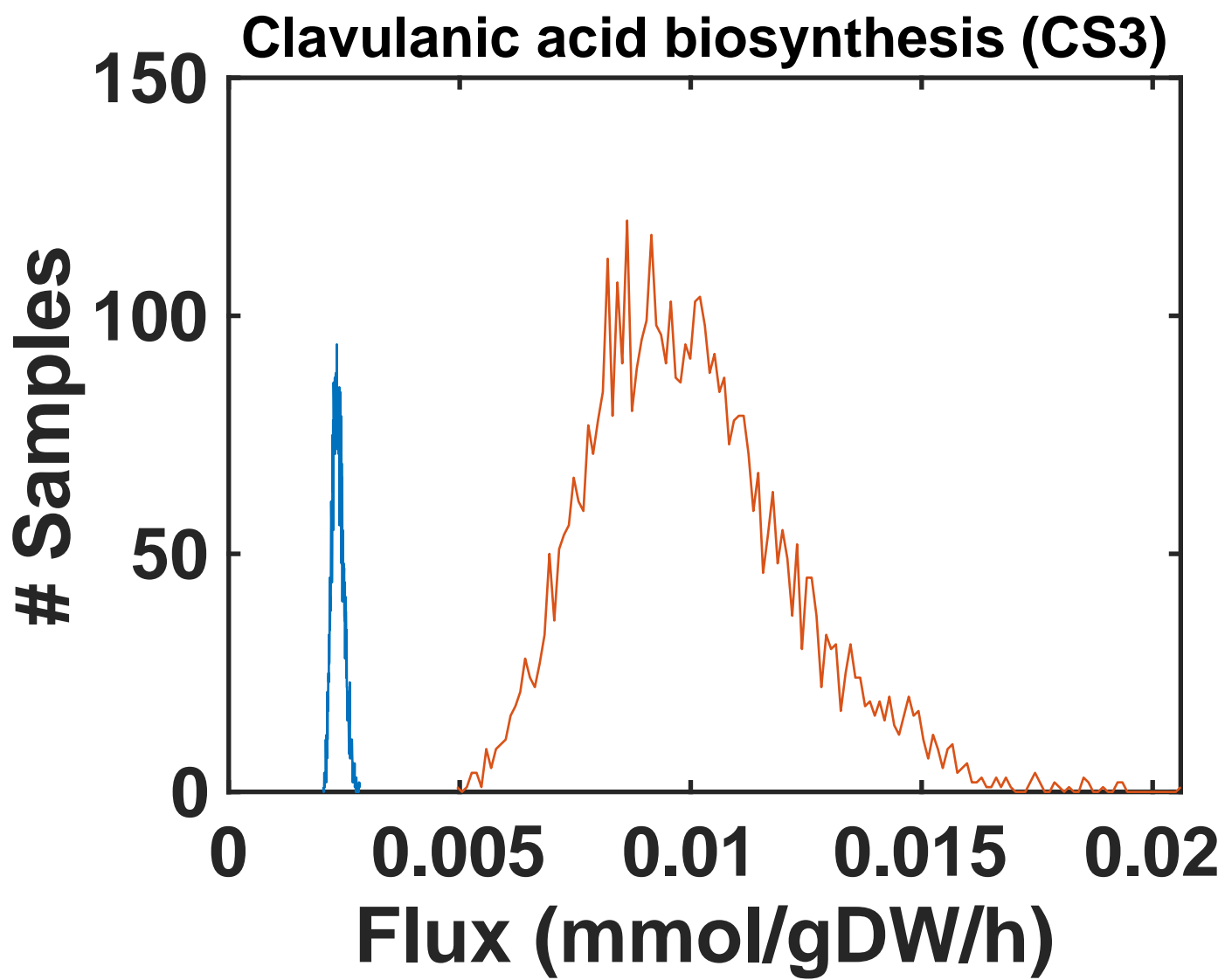

Supplement: Supplementary file 1 [file bioengineering-08-00103-s001.zip › FileS2/figure_sampling_noFVA-CS3.pdf]

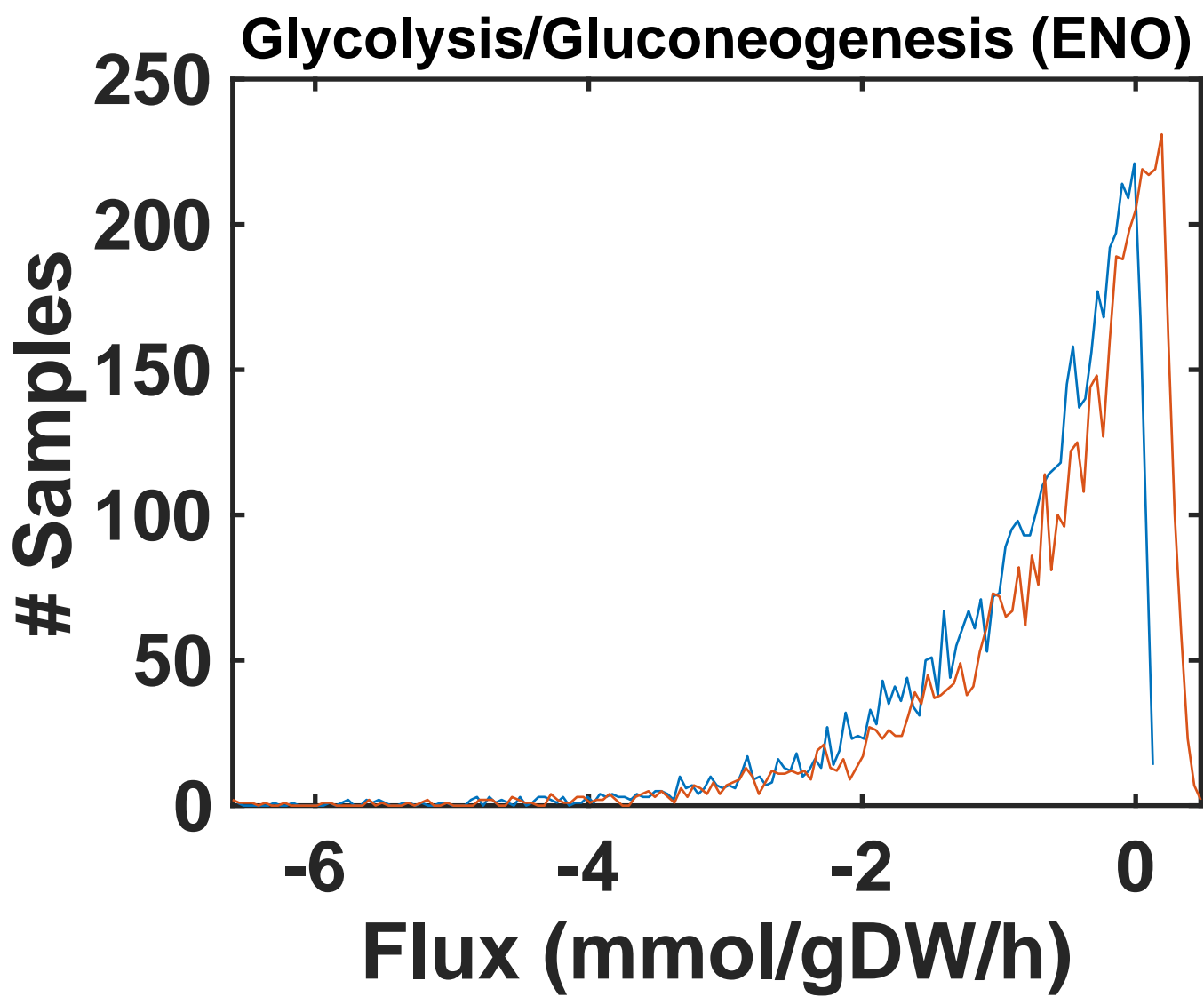

Supplement: Supplementary file 1 [file bioengineering-08-00103-s001.zip › FileS2/figure_sampling_noFVA-ENO.pdf]

Exchange ( $EX_c$  lav<sub>e</sub>)

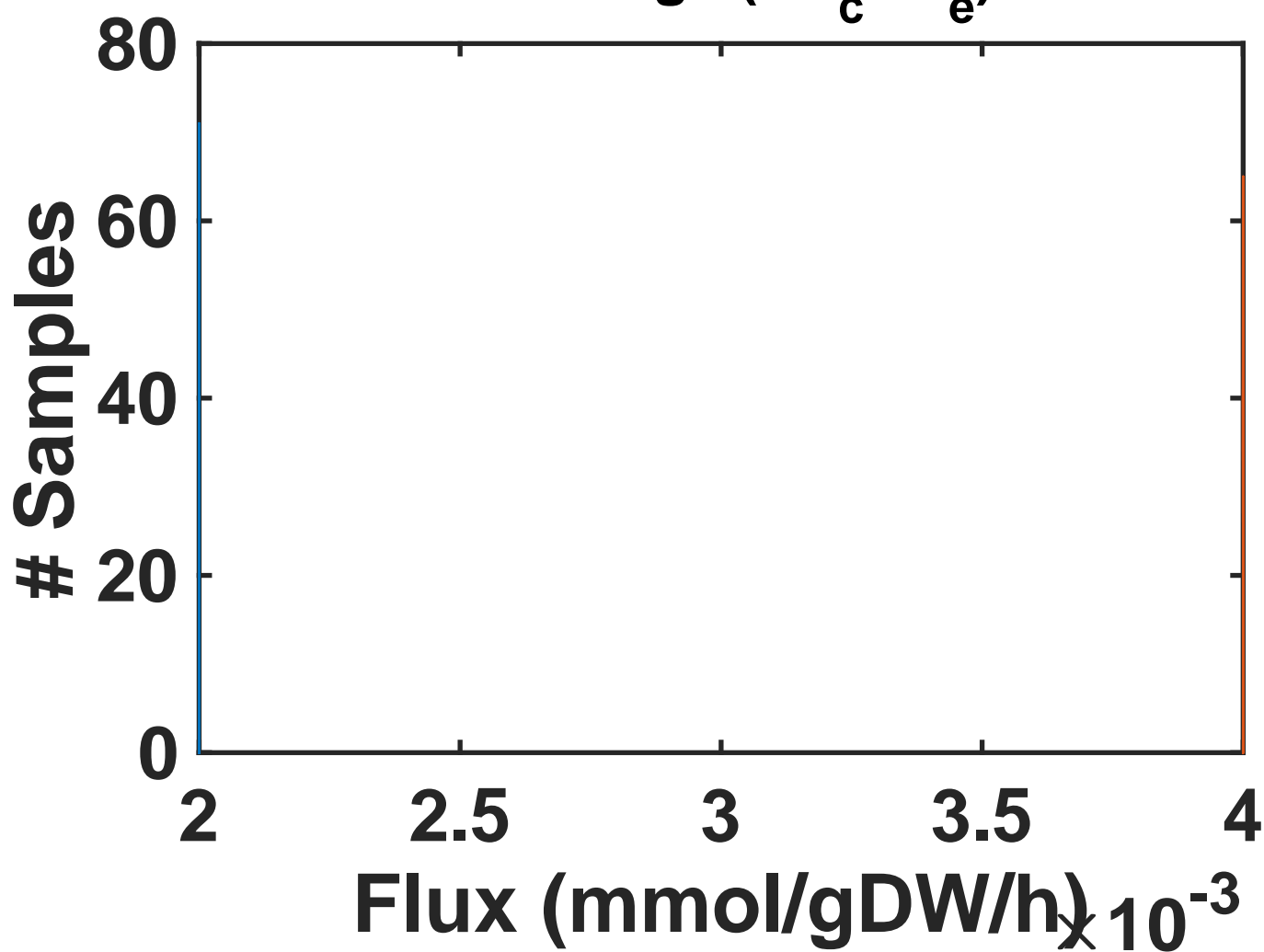

Supplement: Supplementary file 1 [file bioengineering-08-00103-s001.zip › FileS2/figure_sampling_noFVA-EX_clav_e.pdf]

Exchange ( $EX_{c\ o2_e}$ )

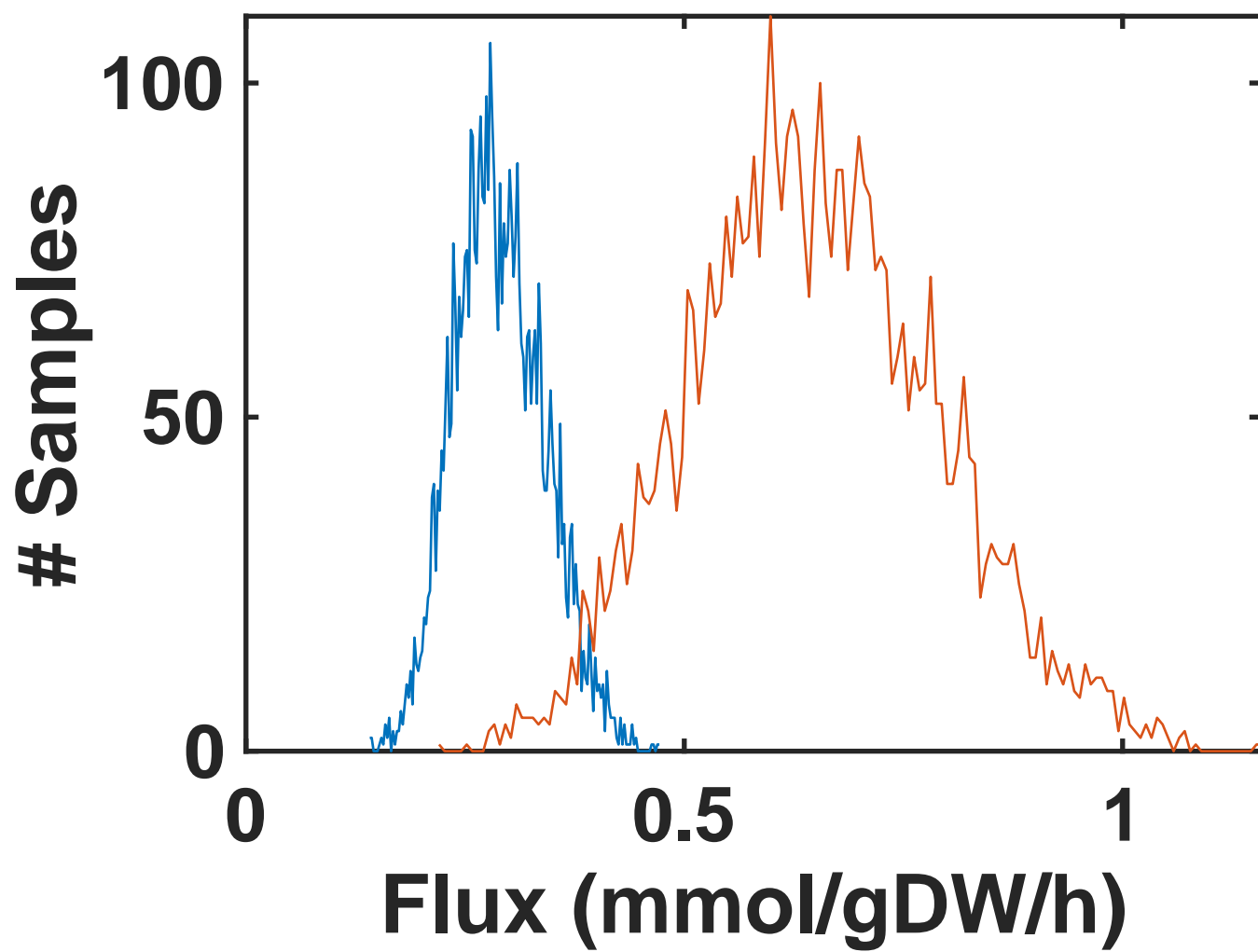

Supplement: Supplementary file 1 [file bioengineering-08-00103-s001.zip › FileS2/figure_sampling_noFVA-EX_co2_e.pdf]

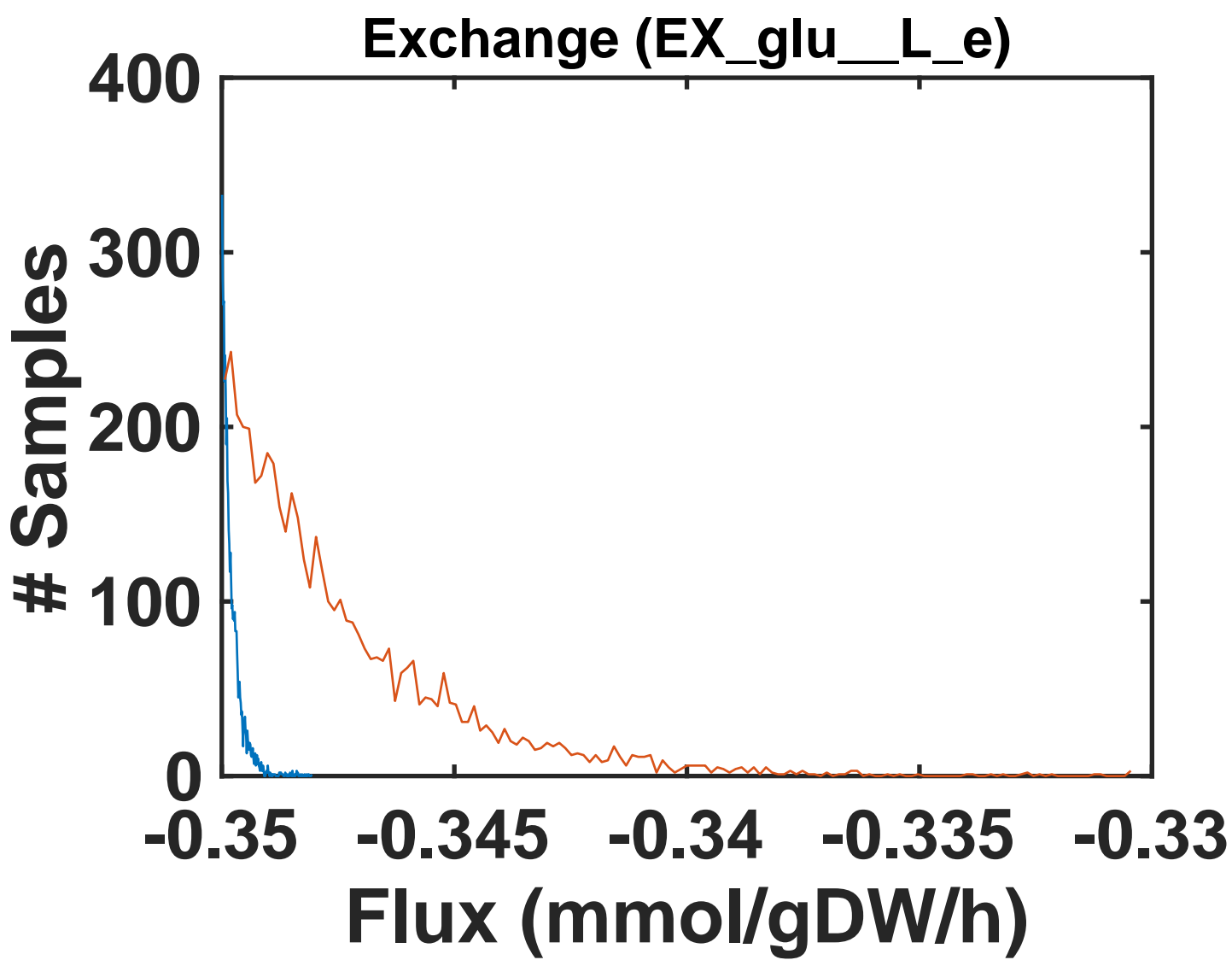

Supplement: Supplementary file 1 [file bioengineering-08-00103-s001.zip › FileS2/figure_sampling_noFVA-EX_glu__L_e.pdf]

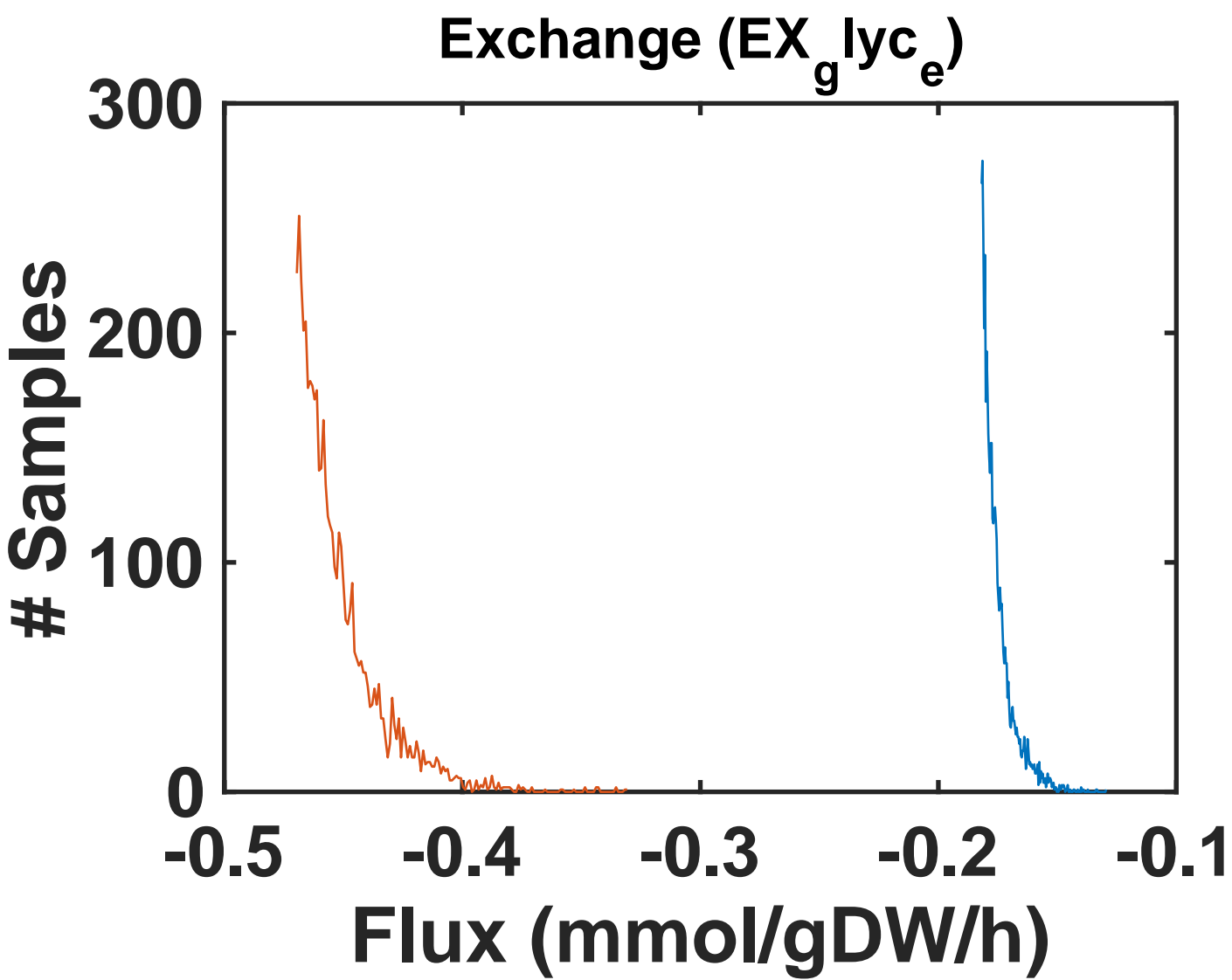

Supplement: Supplementary file 1 [file bioengineering-08-00103-s001.zip › FileS2/figure_sampling_noFVA-EX_glyc_e.pdf]

Exchange ( $EX_n h4_e$ )

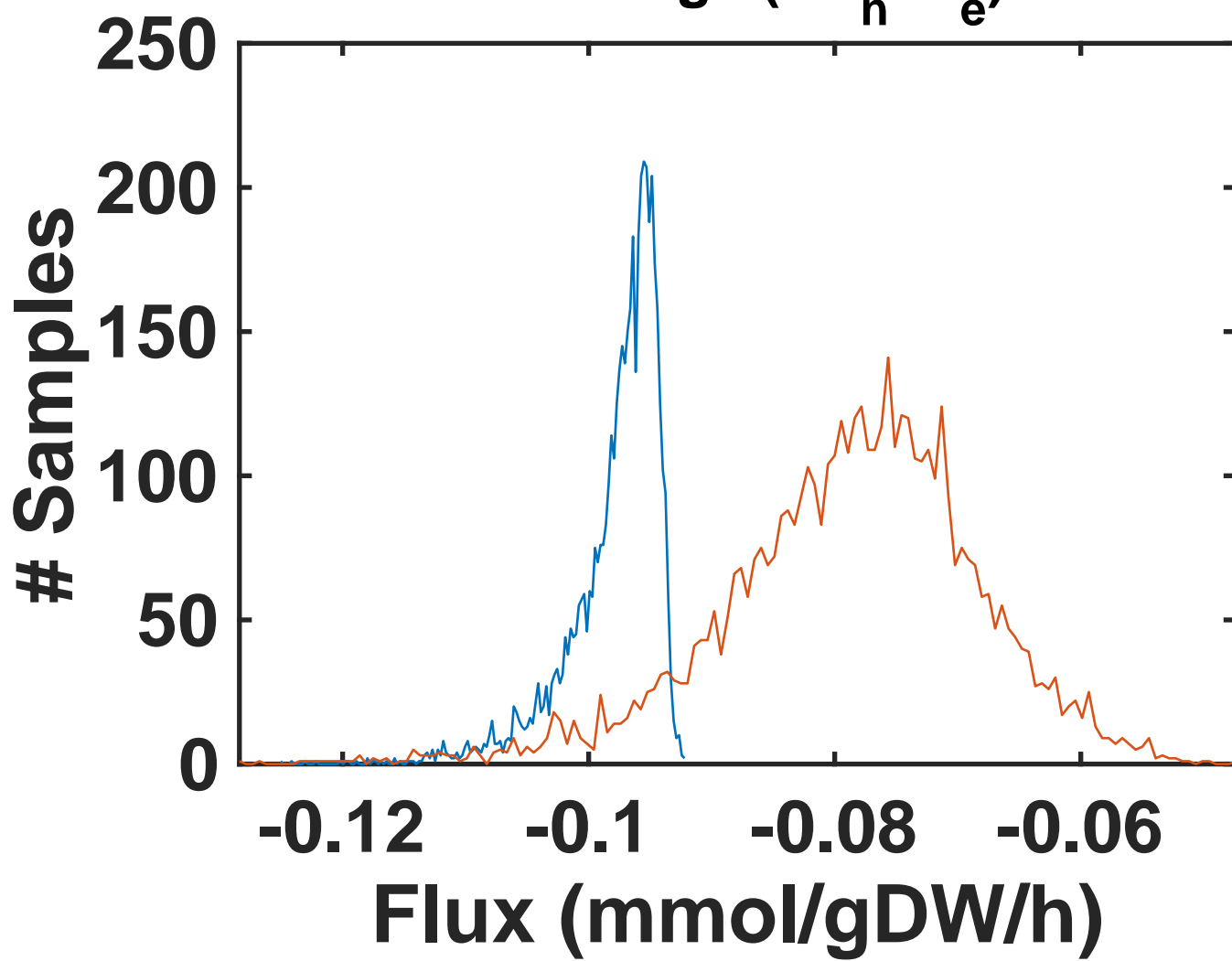

Supplement: Supplementary file 1 [file bioengineering-08-00103-s001.zip › FileS2/figure_sampling_noFVA-EX_nh4_e.pdf]

Exchange ( $EX_o 2_e$ )

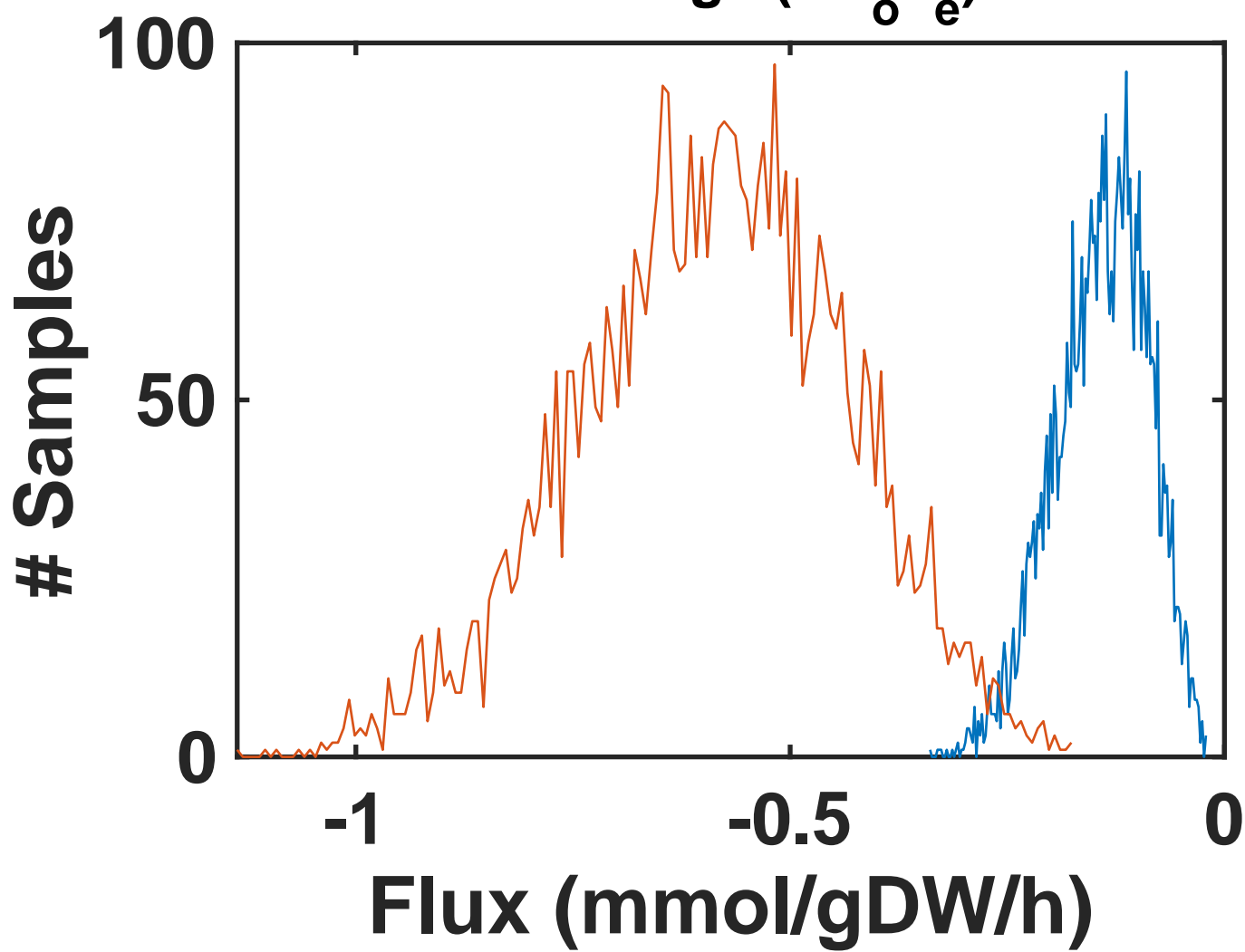

Supplement: Supplementary file 1 [file bioengineering-08-00103-s001.zip › FileS2/figure_sampling_noFVA-EX_o2_e.pdf]

Exchange ( $EX_{pe}$ )

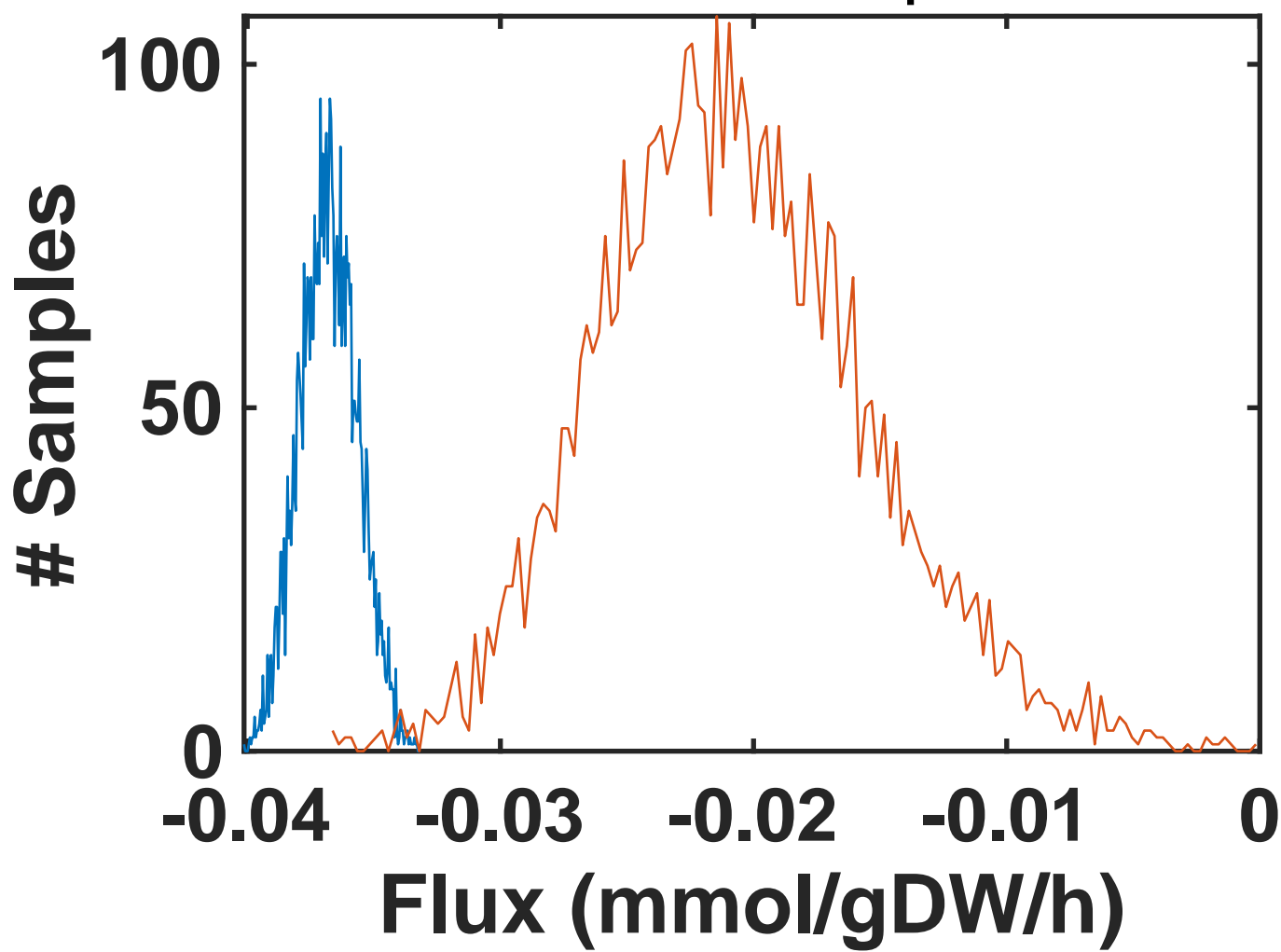

Supplement: Supplementary file 1 [file bioengineering-08-00103-s001.zip › FileS2/figure_sampling_noFVA-EX_pi_e.pdf]

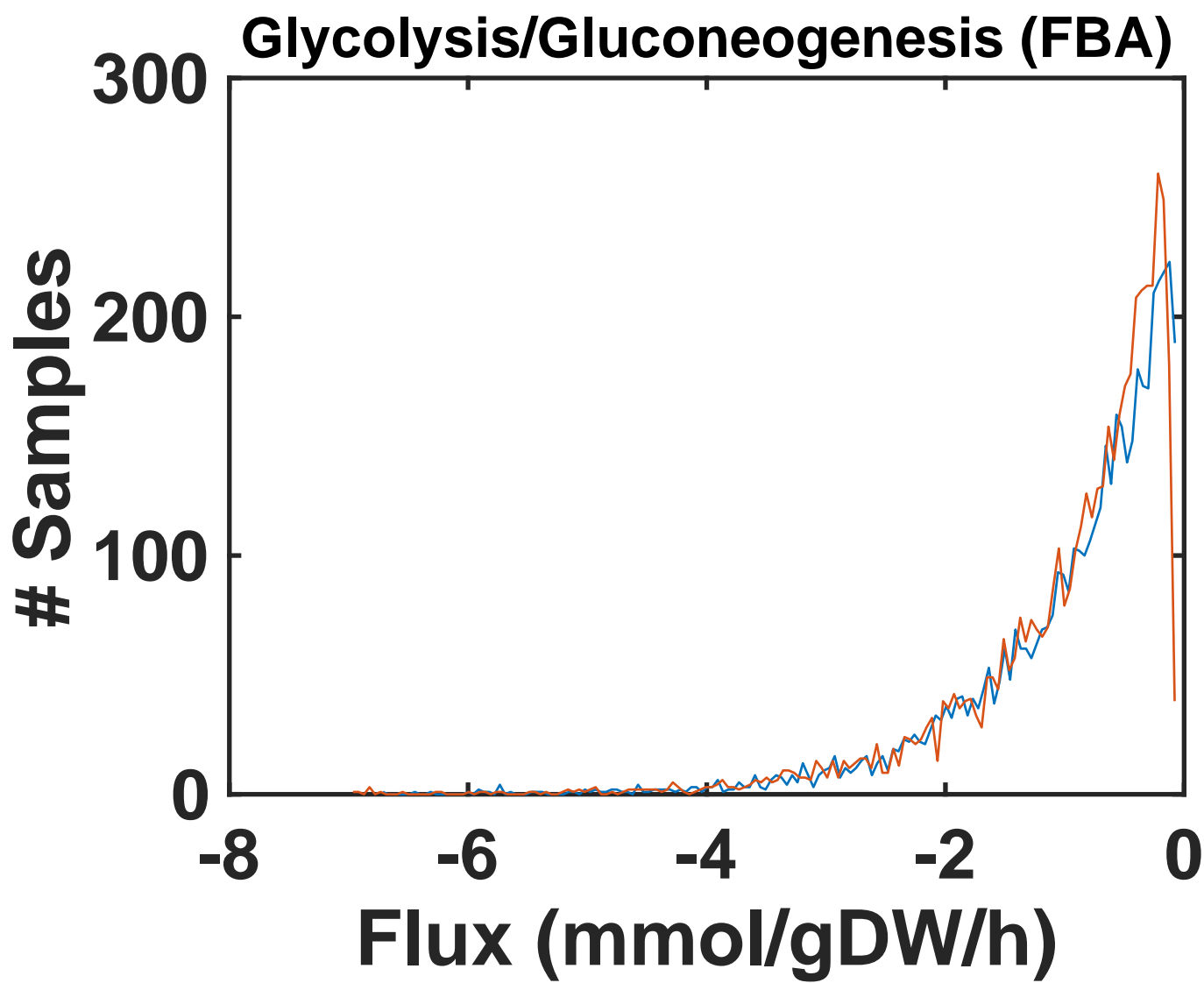

Supplement: Supplementary file 1 [file bioengineering-08-00103-s001.zip › FileS2/figure_sampling_noFVA-FBA.pdf]

# Citric Acid Cycle (FUM)

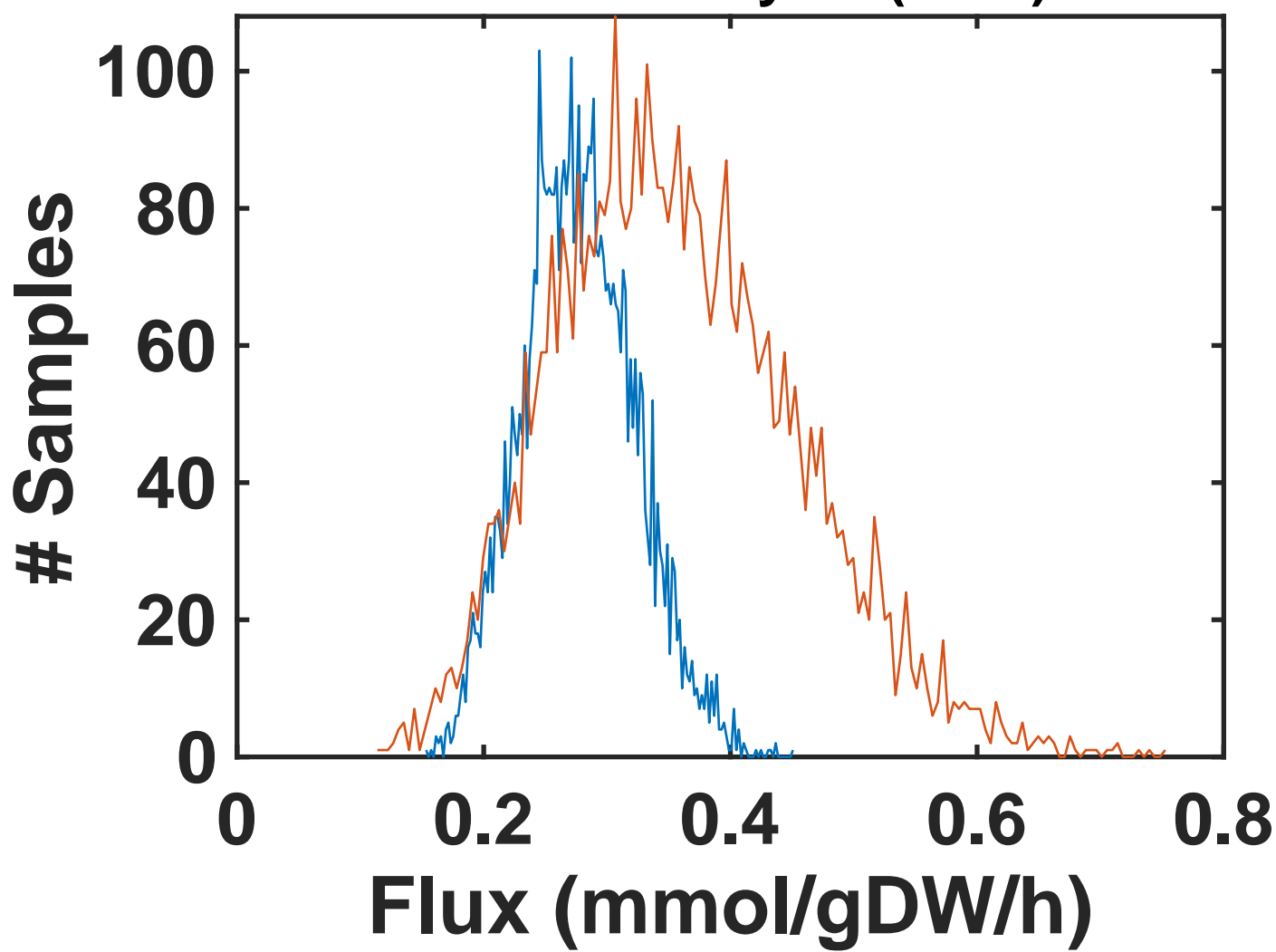

Supplement: Supplementary file 1 [file bioengineering-08-00103-s001.zip › FileS2/figure_sampling_noFVA-FUM.pdf]

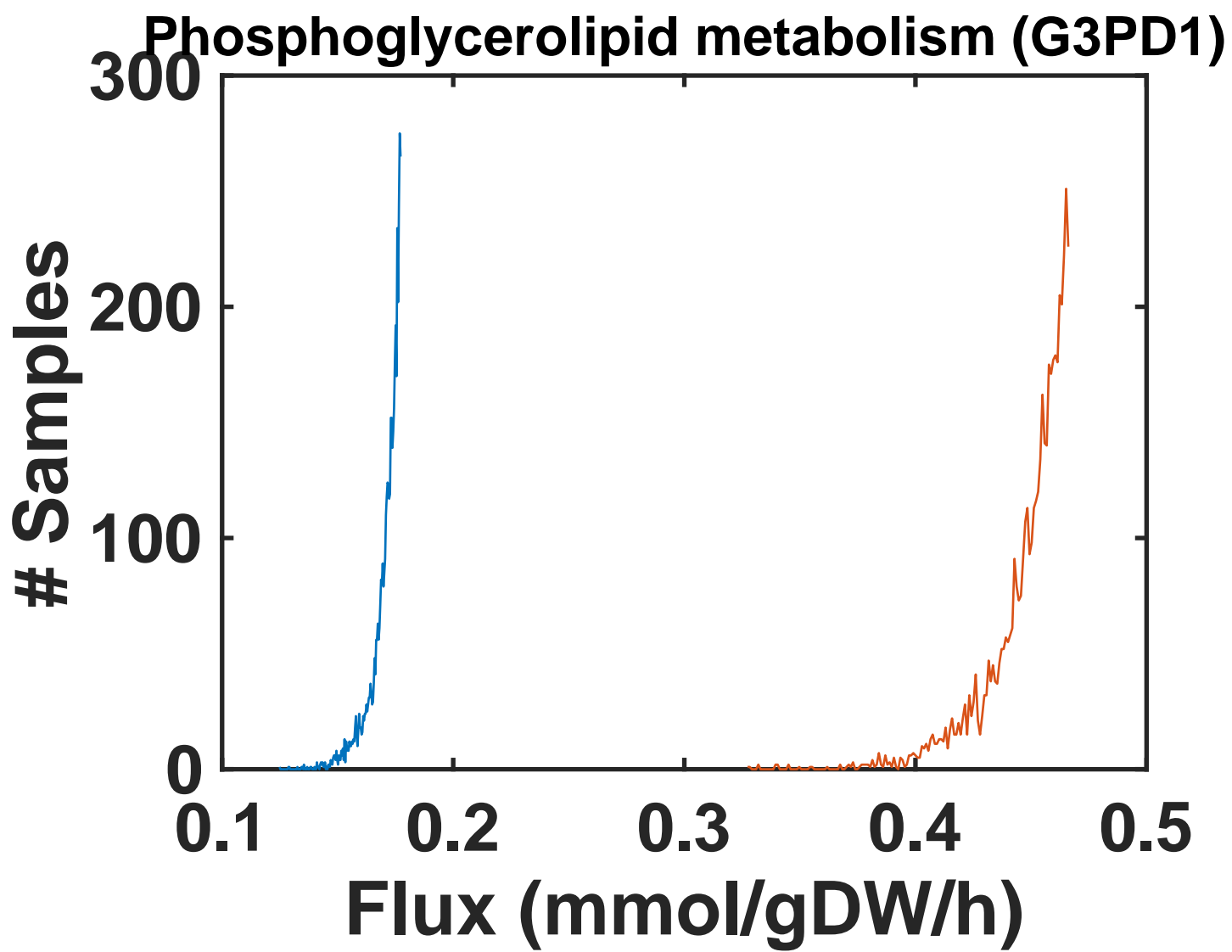

Supplement: Supplementary file 1 [file bioengineering-08-00103-s001.zip › FileS2/figure_sampling_noFVA-G3PD1.pdf]

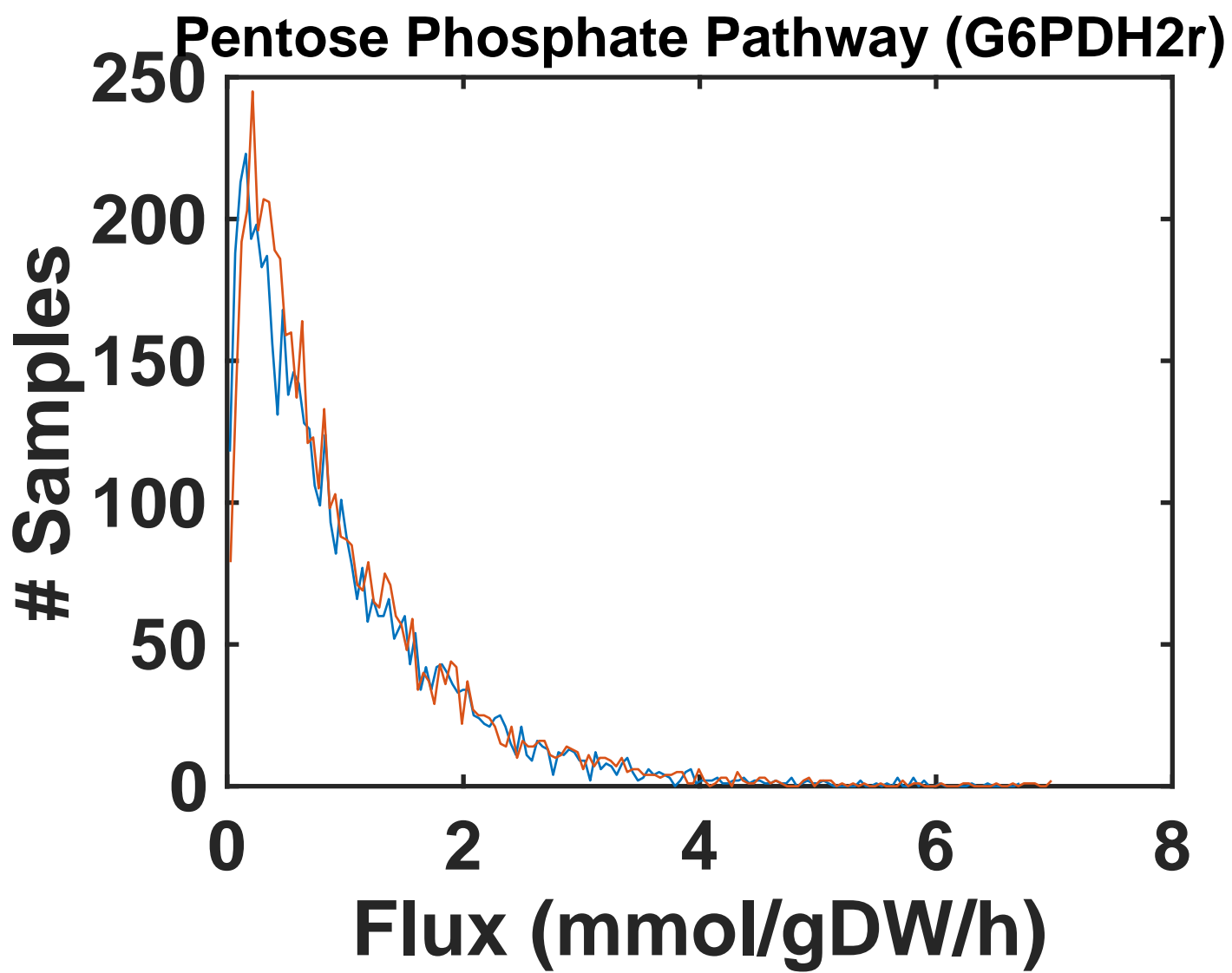

Supplement: Supplementary file 1 [file bioengineering-08-00103-s001.zip › FileS2/figure_sampling_noFVA-G6PDH2r.pdf]

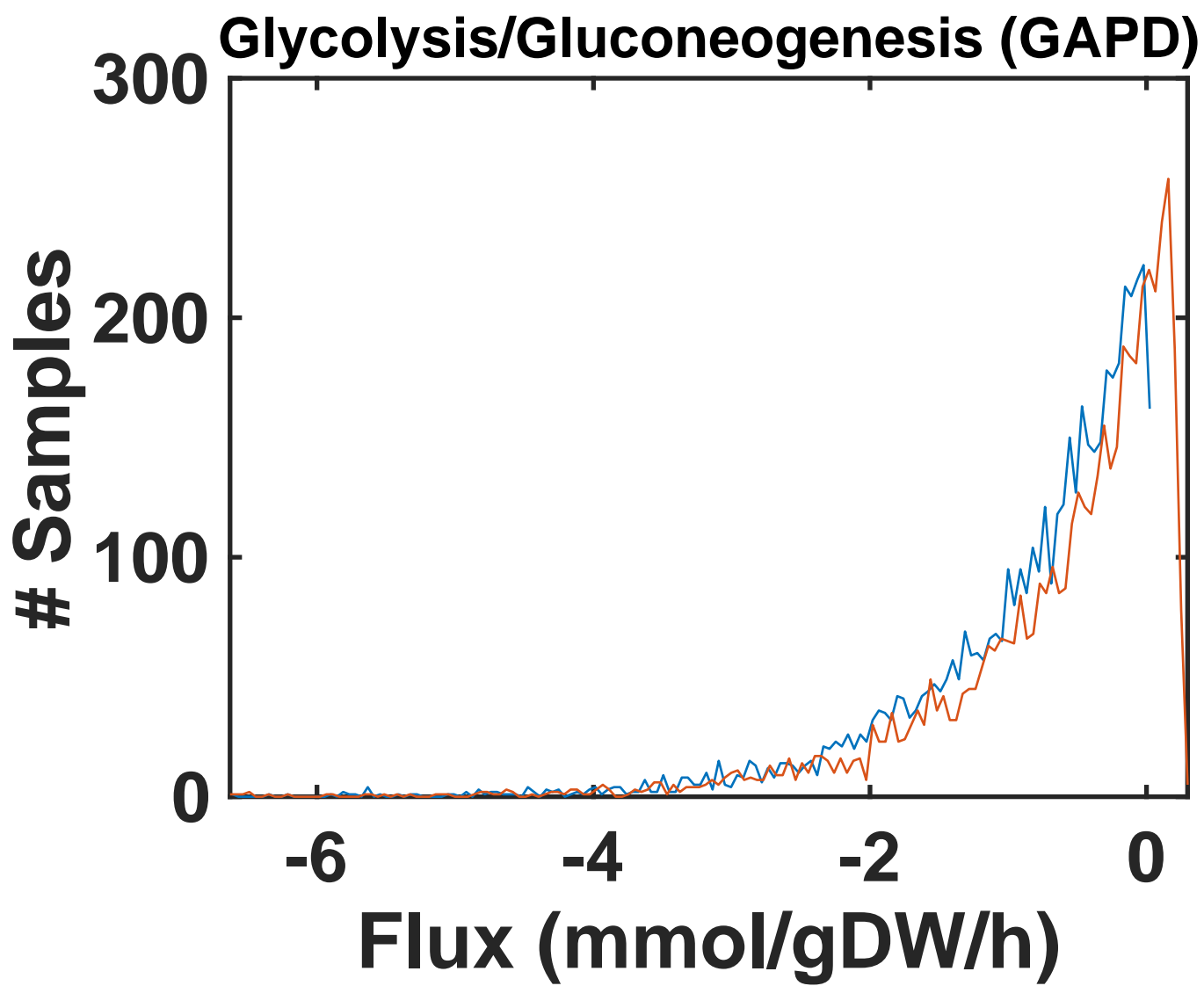

Supplement: Supplementary file 1 [file bioengineering-08-00103-s001.zip › FileS2/figure_sampling_noFVA-GAPD.pdf]

# Glutamate metabolism (GLUDxi)

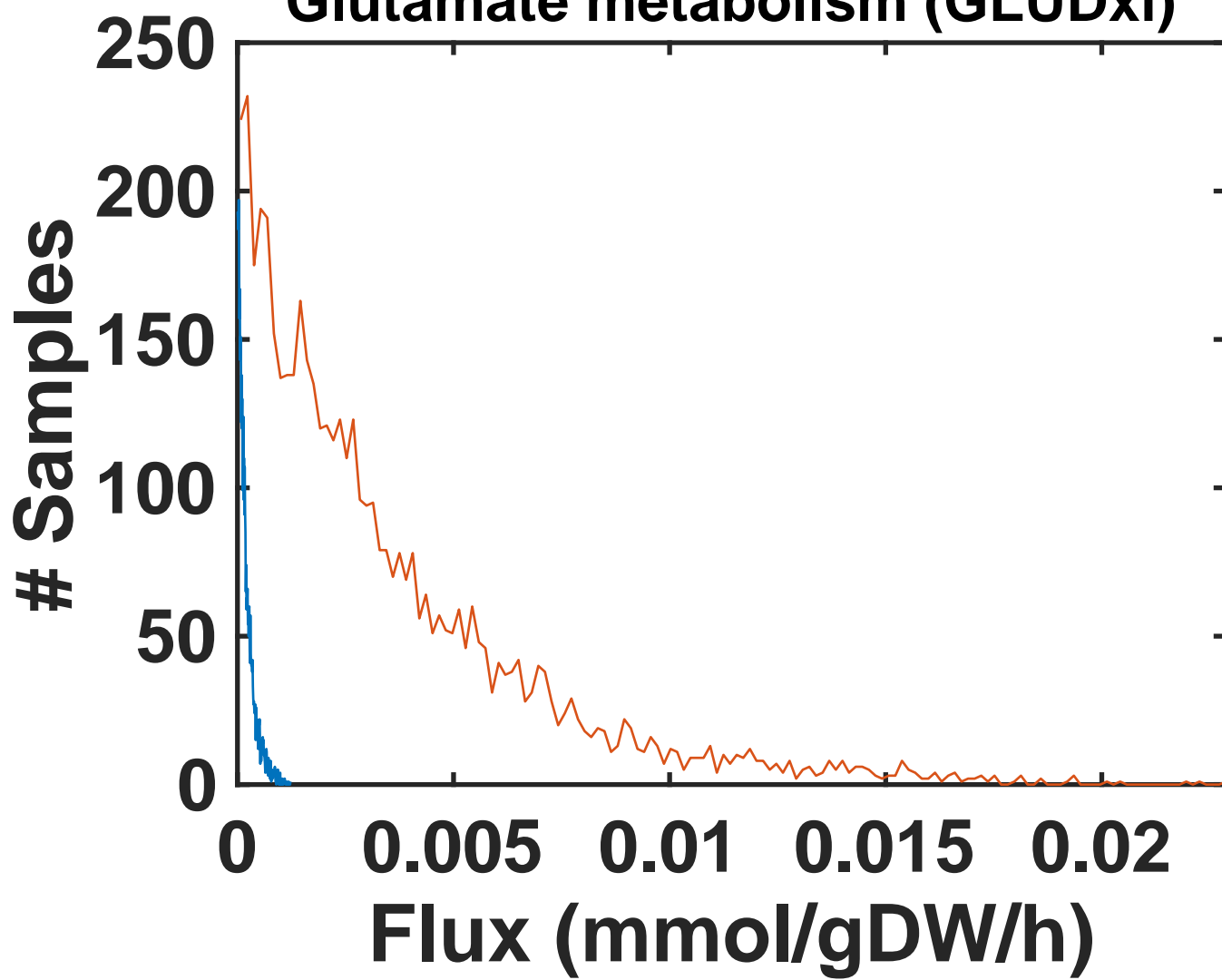

Supplement: Supplementary file 1 [file bioengineering-08-00103-s001.zip › FileS2/figure_sampling_noFVA-GLUDxi.pdf]

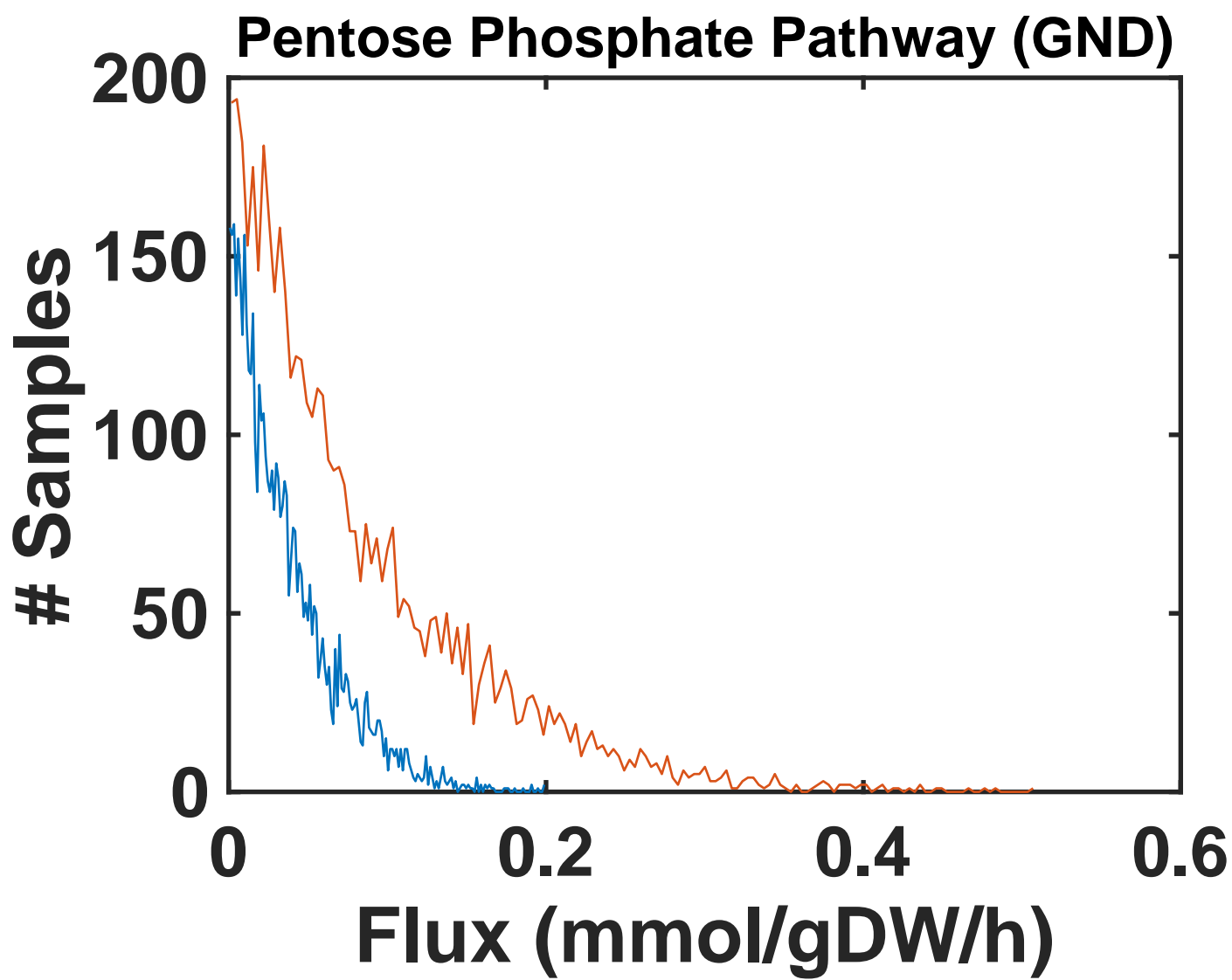

Supplement: Supplementary file 1 [file bioengineering-08-00103-s001.zip › FileS2/figure_sampling_noFVA-GND.pdf]

# Citric Acid Cycle (ICDHyr)

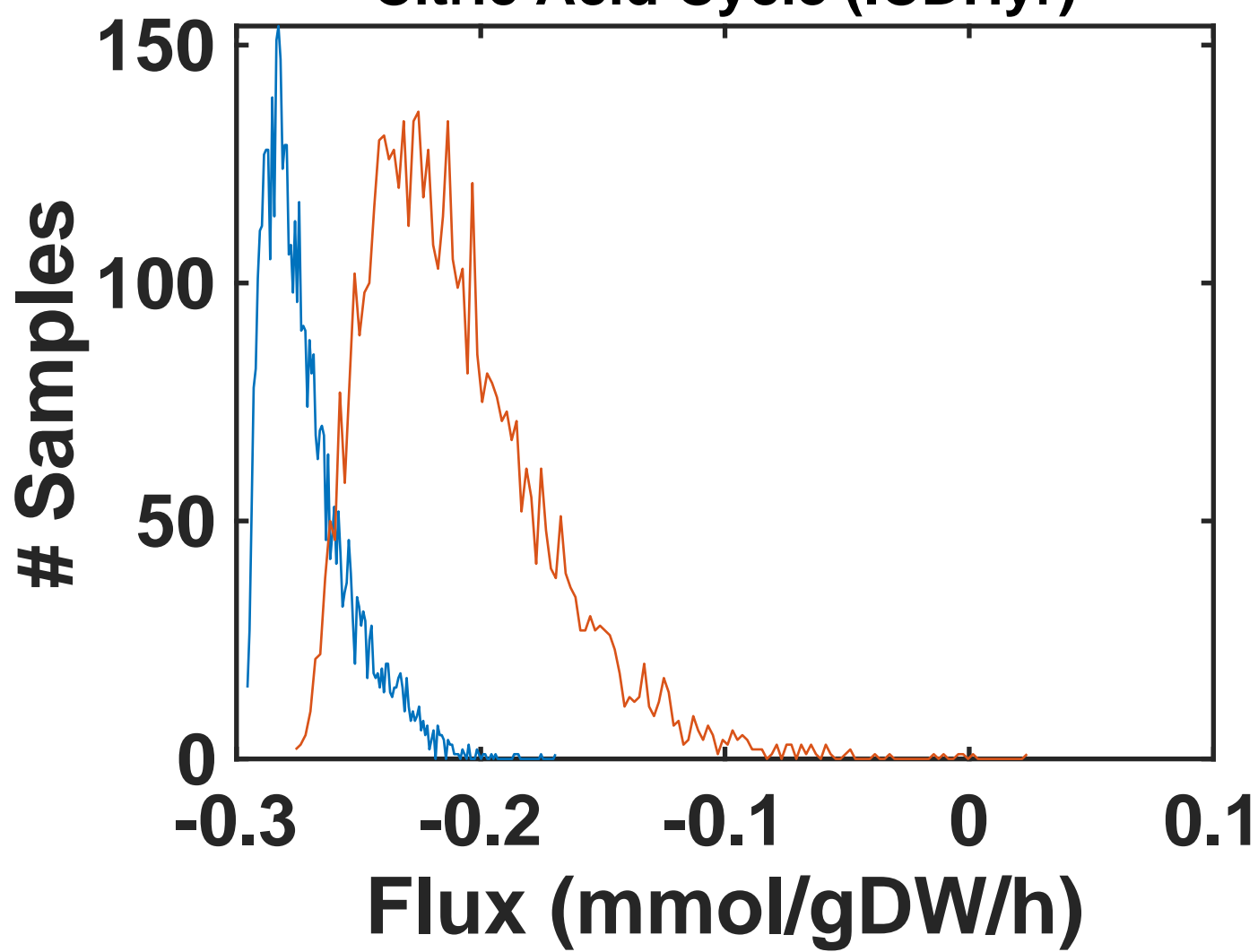

Supplement: Supplementary file 1 [file bioengineering-08-00103-s001.zip › FileS2/figure_sampling_noFVA-ICDHyr.pdf]

## Anaplerotic Reactions (ICL)

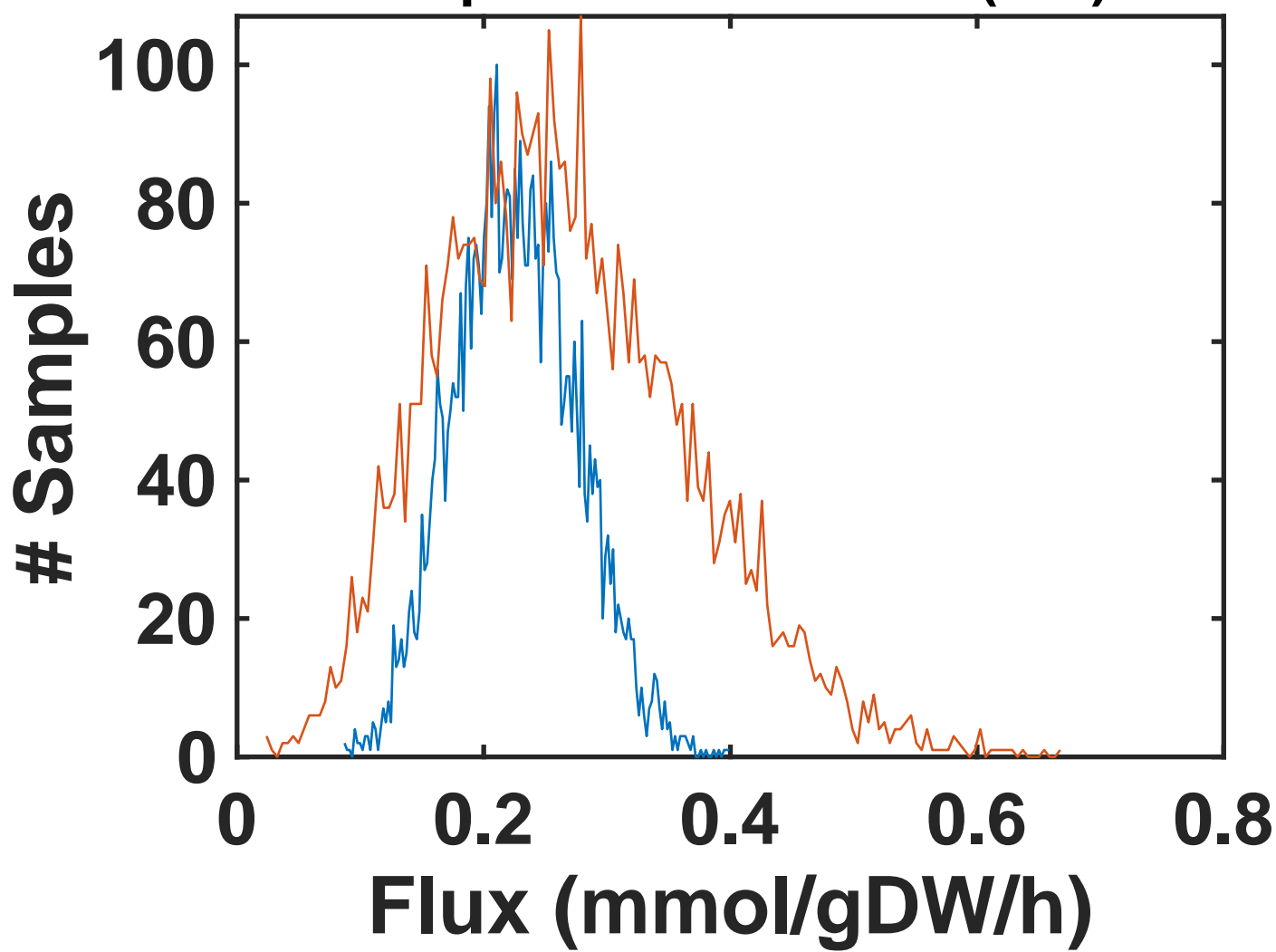

Supplement: Supplementary file 1 [file bioengineering-08-00103-s001.zip › FileS2/figure_sampling_noFVA-ICL.pdf]

## Pyruvate Metabolism (LDH<sub>D</sub>)

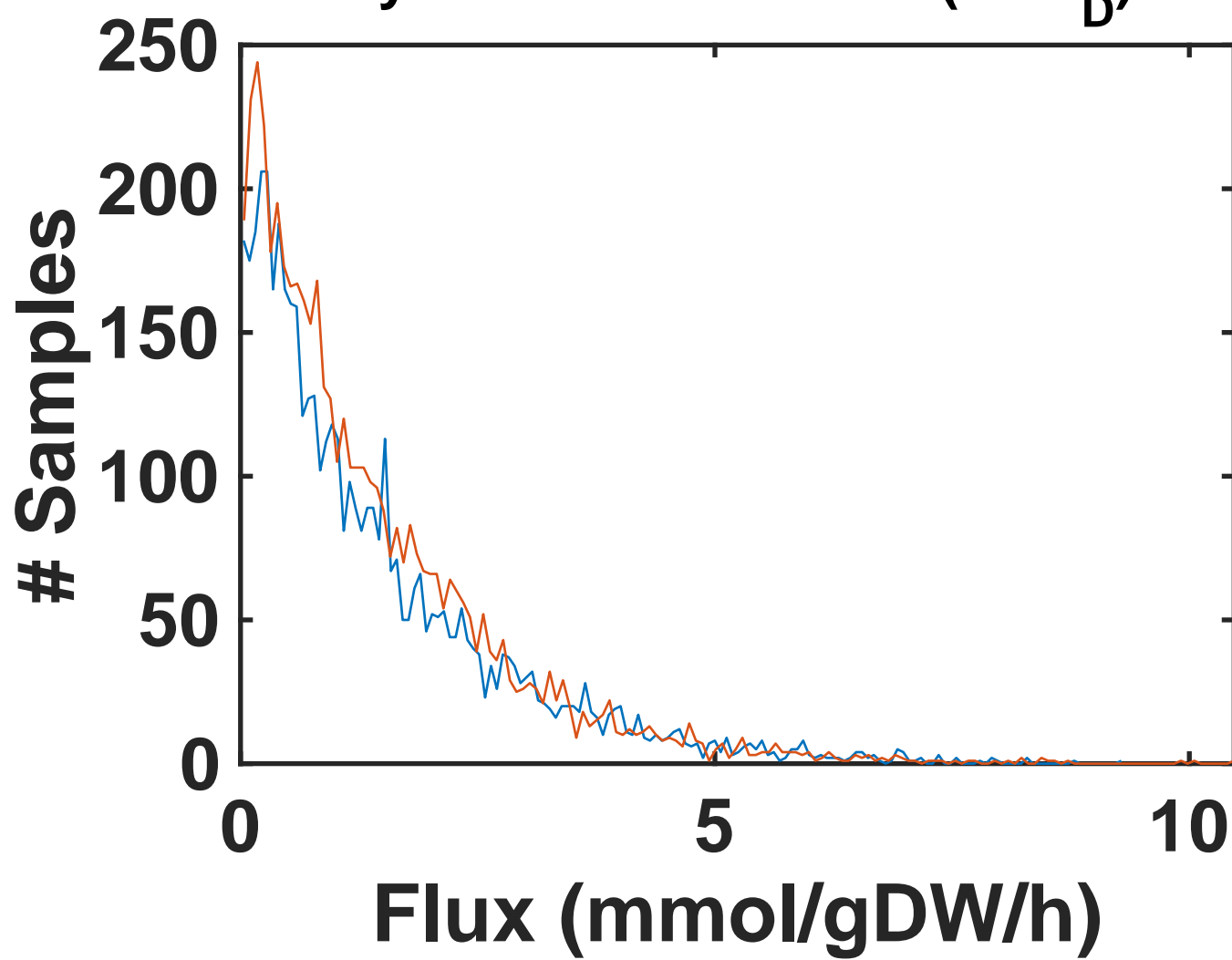

Supplement: Supplementary file 1 [file bioengineering-08-00103-s001.zip › FileS2/figure_sampling_noFVA-LDH_D.pdf]

(LMPD<sub>1</sub>16<sub>g</sub>In-L<sub>c</sub>)

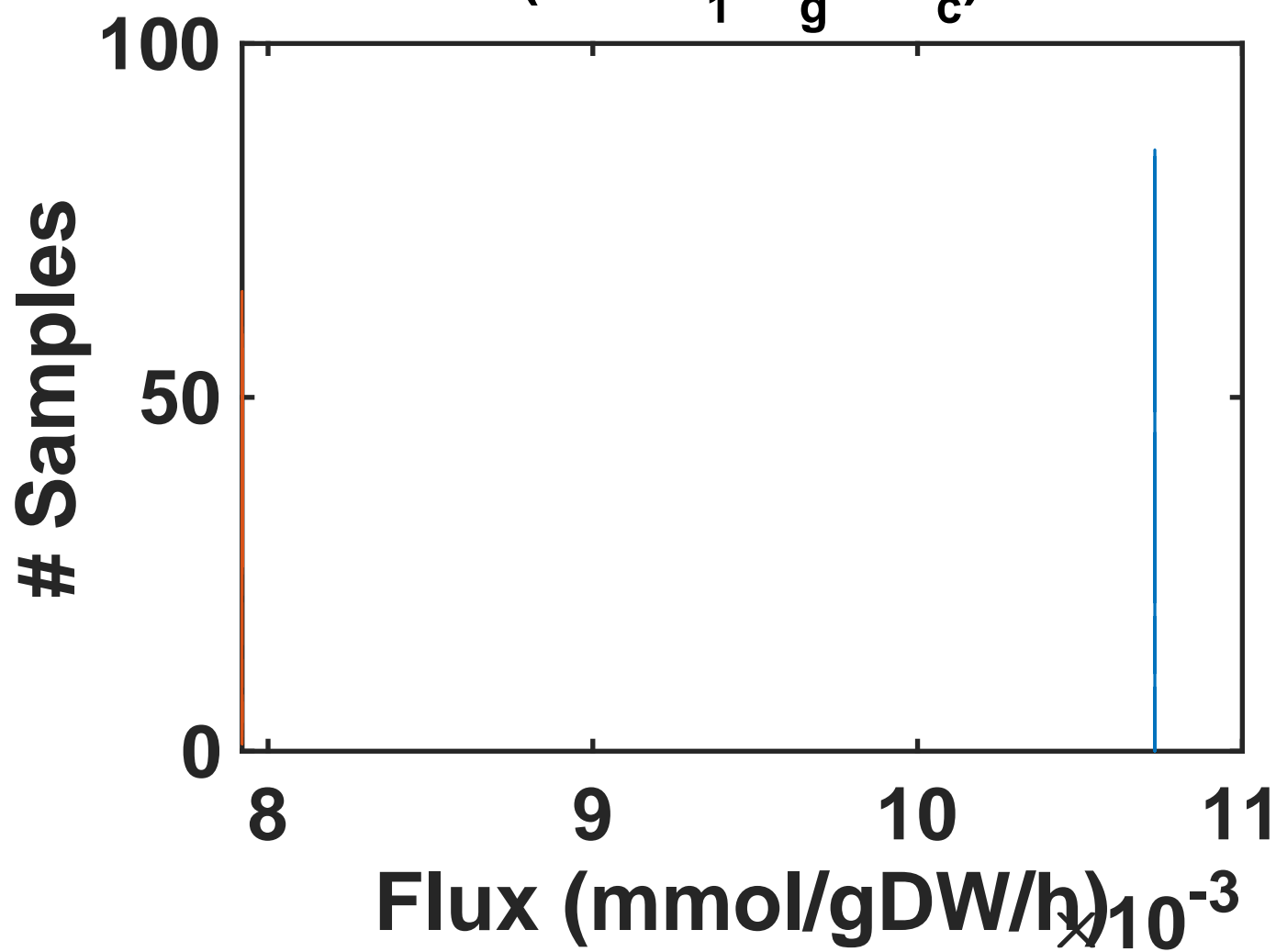

Supplement: Supplementary file 1 [file bioengineering-08-00103-s001.zip › FileS2/figure_sampling_noFVA-LMPD_116_gln-L_c.pdf]

(LMPD<sub>3</sub>arg-L<sub>c</sub>)

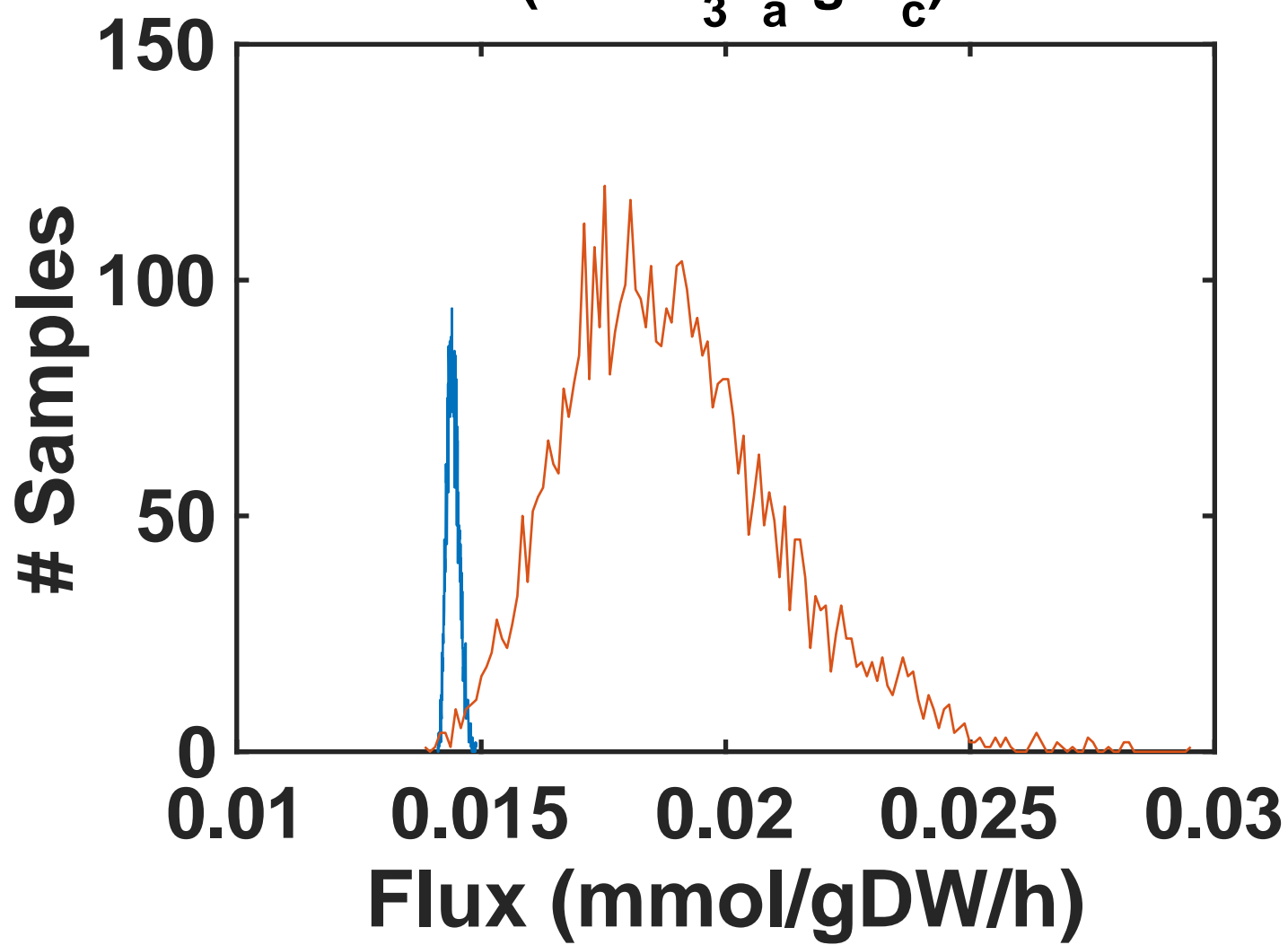

Supplement: Supplementary file 1 [file bioengineering-08-00103-s001.zip › FileS2/figure_sampling_noFVA-LMPD_33_arg-L_c.pdf]

## Oxidative Phosphorylation (L<sub>L</sub>ACD2)

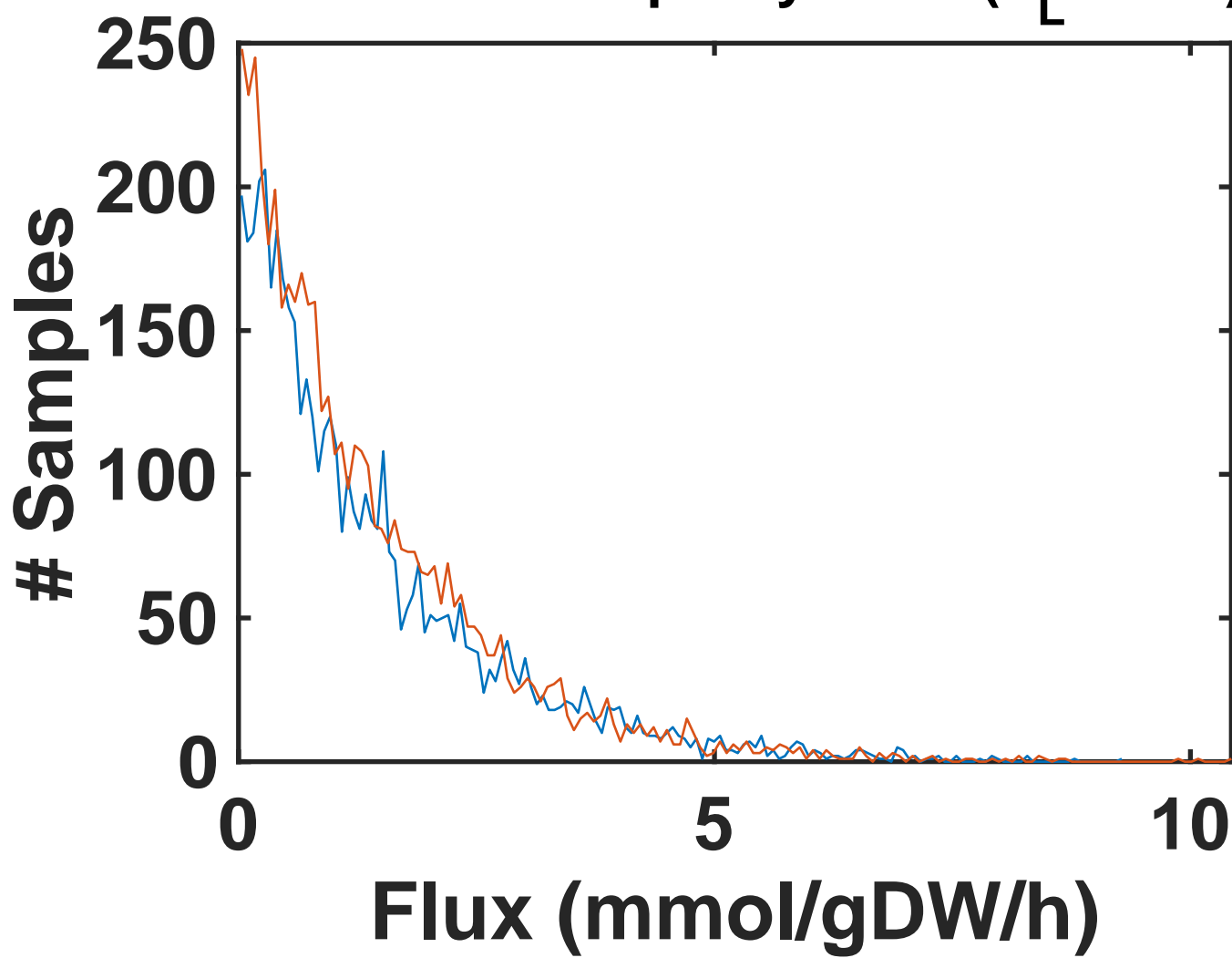

Supplement: Supplementary file 1 [file bioengineering-08-00103-s001.zip › FileS2/figure_sampling_noFVA-L_LACD2.pdf]

## Anaplerotic Reactions (MALS)

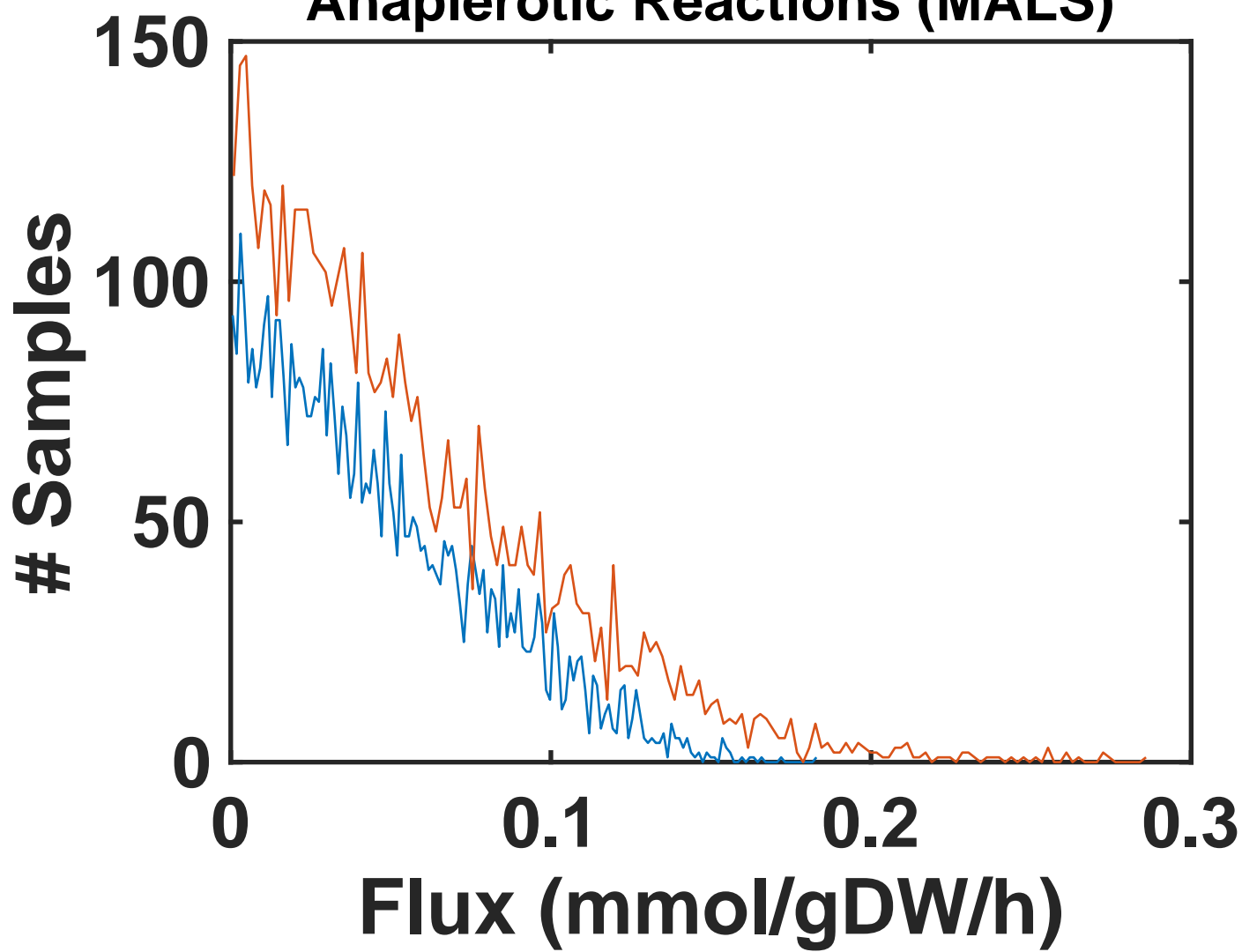

Supplement: Supplementary file 1 [file bioengineering-08-00103-s001.zip › FileS2/figure_sampling_noFVA-MALS.pdf]

# Citric Acid Cycle (MDH)

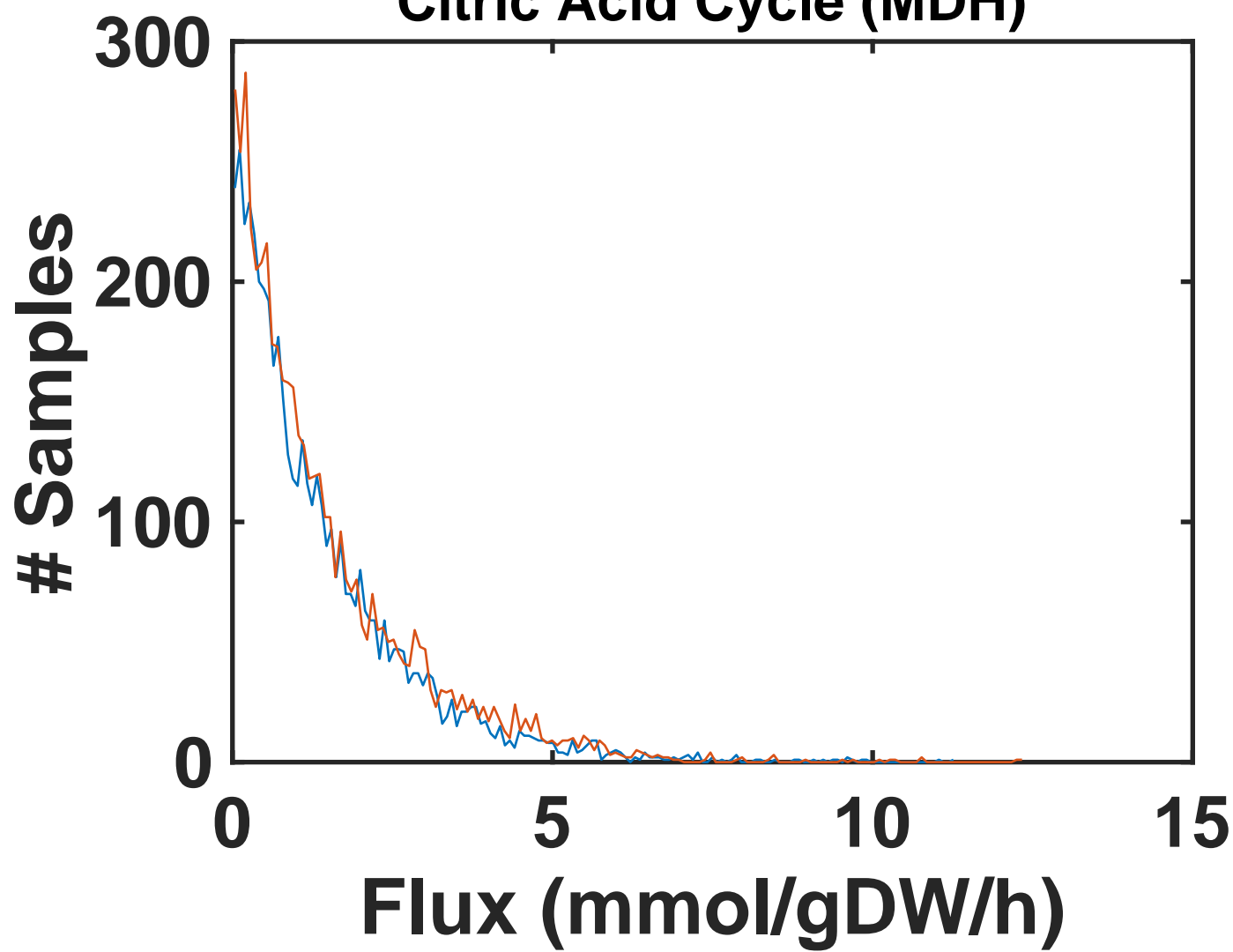

Supplement: Supplementary file 1 [file bioengineering-08-00103-s001.zip › FileS2/figure_sampling_noFVA-MDH.pdf]
